# Photothermal Conversion Promotes Challenging S_N_Ar for Facile C─N Bond Formation

**DOI:** 10.1002/anie.202522296

**Published:** 2026-01-18

**Authors:** Megan E. Matter, Rory C. Devin, Erin E. Stache

**Affiliations:** ^1^ Department of Chemistry Princeton University Princeton NJ 08544 USA

**Keywords:** C─N bond formation, Heteroarenes, Heterocycles, Nucleophilic aromatic substitution, Photothermal conversion

## Abstract

Nucleophilic aromatic substitution (S_N_Ar) is a widely used method for forming aromatic C─N bonds, which are of interest in both industry and academia. However, current approaches are often unable to access less activated electrophiles, due to negative charge buildup in the transition state, resulting in high activation energy barriers. Inspired by our work on the Newman Kwart Rearrangement (NKR), we sought to leverage photothermal conversion for challenging C─N bond‐forming S_N_Ar reactions. Here, we demonstrate that the incorporation of an inexpensive photothermal agent, carbon black, and irradiation with red light affords several poorly activated intermolecular substitution reactions. Application to less activated aryl halides resulted in unproductive reactivity, leading us to examine the reaction barriers. Computations revealed barriers within the range previously achieved during our photothermally mediated NKR. Electronically neutral intramolecular analogs were synthesized and underwent productive reactivity in short time frames (≤20 min), indicating that the inhomogeneous nature of photothermal heating was a challenge in terms of colocalizing reactants sufficiently close to the particle. This concept was leveraged into a sequential S_N_Ar, where an initial intermolecular reaction occurs which then primes the substrate for a more difficult intramolecular substitution. This approach afforded a diverse scope of fused heterocycles.

## Graphical abstract

Photothermal conversion enables facile aromatic C─N bond formation via red light irradiation. A variety of inter‐ and intramolecular S_N_Ar transformations are achieved under mild, schematically simple conditions. Additionally, a thermally promoted halogen exchange mechanism provides access to aryl bromides, chlorides, and fluorides – making this approach an excellent synthetic tool for forming aromatic C─N bonds.

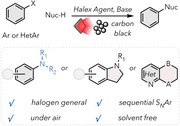

## Introduction

**Figure 1:**
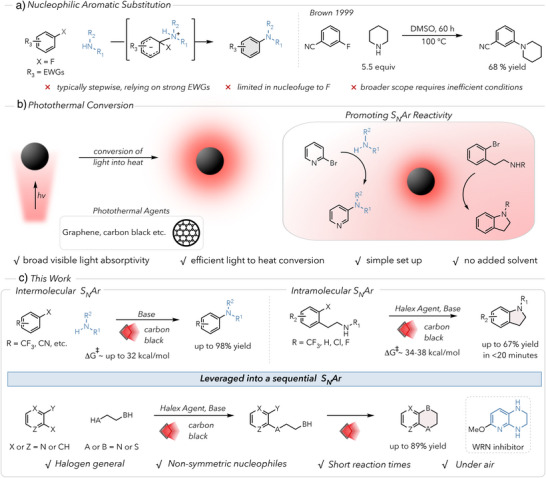
a) Challenges associated with S_N_Ar. b) Intense, localized heating is unique to photothermal conversion compared to traditional bulk heating. c) Demonstration of the lower barrier intermolecular S_N_Ar, the higher barrier intramolecular S_N_Ar, and the sequential approach. WRN inhibitor refers to a drug which inhibits the Werner Syndrome Helicase enzyme.

Aromatic C─N bond formation is widely studied and sought‐after, owing to the large number of C─N bonds in pharmaceuticals, agrochemicals, and other industrially relevant molecules ^[^ ^1^, ^2^ ^]^ Metal‐mediated cross couplings, such as the Ullman‐Goldberg and Buchwald‐Hartwig couplings,^[^ ^3^, ^4^, ^5^ ^]^ offer an effective route, providing the C─N coupled product from unactivated aryl chlorides and bromides. S_N_Ar (nucleophilic aromatic substitution), wherein a nucleophile directly attacks the aryl ring is another common route to generating aromatic C─N bonds (Figure 1a). These two methods have been identified as some of the most frequently used reactions in academia and industry, highlighting the ubiquity and importance of the aromatic C─N bond.^[^ ^1^ ^]^ While S_N_Ar does not necessitate the inclusion of a transition metal or designer ligands, their use is often limited to strongly activated aryl halides, typically requiring inclusion of strong electron‐withdrawing groups and the use of fluorine as the leaving group. This constraint arises from the buildup of negative charge during the reaction, resulting in exceptionally high activation energy barriers in less electron‐deficient substrates. These factors limit the application of S_N_Ar with other nucleofuges (X═Br, Cl, I) and more electron‐neutral/donating substituents.^[^ ^6^, ^7^, ^8^ ^]^

Photothermal conversion involves the transformation of light into heat, resulting from a nonradiative decay pathway that certain materials favor upon excitation with light.^[^ ^9^, ^10^, ^11^, ^12^, ^13^, ^14^, ^15^ ^]^ Amongst the vast array of materials that absorb light, numerous photothermal agents (PTAs) have exhibited efficient light‐to‐heat conversion. These species have been leveraged in photothermal therapeutics,^[^ ^16^, ^17^ ^]^ macromolecular syntheses,^[^ ^18^, ^19^, ^20^ ^]^ and degradation,^[^ ^21^, ^22^, ^23^ ^]^ alongside a few examples of challenging small molecule reactivity.^[^ ^10^, ^24^, ^25^, ^26^ ^]^ Our lab has been particularly interested in carbonaceous photothermal agents, due to their low cost and broad absorption in the visible region.^[^ ^10^, ^21^, ^23^ ^]^ Carbonaceous materials, such as carbon black (CB) are comprised of highly conjugated sp^2^ carbons with small HOMO–LUMO gaps, allowing for an incredibly short excitation lifetime (10^−12^ s) which precludes excited state reactivity.^[^ ^27^, ^28^, ^29^ ^]^ Relaxation to the ground state is instead achieved through the release of kinetic energy. The resulting vibrational movement generates intense heating localized to the surface of the particle, dissimilar to traditional bulk heating (Figure 1b). The inhomogeneous heating allows for a lower global reaction temperature, deterring some of the side reactivity typically associated with the use of high temperatures.^[^ ^30^, ^31^, ^32^ ^]^ Visible light irradiation thus provides access to high local temperatures to promote reactions with otherwise prohibitively high activation barriers. Photothermal conversion offers a photo‐mediated approach to thermally challenging reactivity without the need for microwave reactors or use of high dielectric constant solvents, offering an operationally simple protocol.^[^ ^33^, ^34^, ^35^ ^]^

We previously demonstrated a photothermally promoted Newman–Kwart Rearrangement (NKR), a challenging, intramolecular, concerted S_N_Ar (cS_N_Ar) with high activation barriers (up to 43 kcal/mol).^[^ ^10^ ^]^ Encouraged by this success, we hypothesized that photothermal conversion could broaden the scope of S_N_Ar reactions, providing access to otherwise energetically challenging C─N bond formation. Previous reports have demonstrated that cS_N_Ar reactions can provide access to less electron‐poor arenes which are inaccessible via the classical addition‐elimination mechanism.^[^ ^36^, ^37^, ^38^, ^39^, ^40^ ^]^ Moreover, aryl chlorides, bromides, and iodides (which are often more readily available than the fluorides traditionally used for S_N_Ar)^[^ ^41^ ^]^ have been reported to undergo the concerted mechanism preferentially.^[^ ^38^, ^39^ ^]^ While cS_N_Ar involves a transition state similar to the Meisenheimer intermediate making it more tolerant of electron‐donating substituents, its concerted nature favors substrates with better leaving groups (X═Br, I). Thus, we aimed to develop a photothermal system for aromatic C─N bond formation through the substitution of aryl halides with amine nucleophiles.

Here, we demonstrate that under visible light irradiation, using carbon black and a strong organic base, we can readily achieve several poorly activated intermolecular S_N_Ar reactions. In seeking to apply this method to highly challenging substrates, we found that while electron neutral/donating intermolecular aryl halides were unable to form the desired C─N bond, corresponding intramolecular substrates readily underwent cyclization. Taken together, these results indicated that lower barrier reactions readily occur at a greater distance from carbon black's surface. In contrast, higher barrier reactivity necessitates co‐localization of reactants at a very short distance, putting an entropic constraint on reactivity. This concept was further realized in a sequential S_N_Ar, where a more activated intermolecular reaction occurs first to prime the substrate for a second, more challenging intramolecular step. These reactions provided synthetically useful yields under schematically simple conditions free of added transition metal and under air. Our approach accesses a wide variety of unreported fused heterocycles, displaying unprecedented reactivity, and a pathway to rapidly build molecular complexity.^[^ ^42^ ^]^

## Results and Discussion

**Figure 2:**
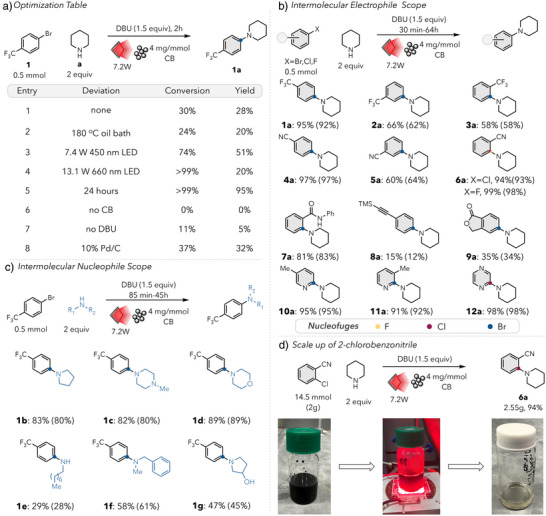
a) Optimization table for intermolecular S_N_Ar. Yields were calculated using ^1^H‐NMR using 1,3,5‐trimethoxybenzene as an internal standard. b) Electrophile scope for intermolecular S_N_Ar reacting with piperidine. Yields were calculated using ^1^H‐NMR using 1,3,5‐trimethoxybenzene as an internal standard. Isolated yields in parentheses. c) Nucleophile scope for intermolecular S_N_Ar reacting with 4‐trifluoromethyl bromobenzene. Yields were calculated using ^1^H‐NMR using 1,3,5‐trimethoxybenzene as an internal standard. Isolated yields in parentheses. d) Scale up of 2‐chlorobenzonitrile with piperidine.

There are relatively few reports regarding thermally promoted electron‐donating or electron‐neutral S_N_Ar reactions. Thus, we began our investigations with 4‐trifluoromethyl bromobenzene (**1**), a challenging electrophile reported to undergo cS_N_Ar.^[^ ^36^, ^38^ ^]^ Based on previous work, we determined that carbon black (CB) functioned as an optimal photothermal agent due to its low cost and broad absorption spectrum (Figure 2a). Using 1,8‐diazabicyclo [5.4.0] undec‐7‐ene (DBU) as a strong organic base, CB, and piperidine as a nucleophile, the desired product **1a** was formed in 30% yield after 2 h of red light irradiation (Figure 2a, entry 1). To achieve comparable reactivity thermally, we found that a temperature of at least 180 °C was required (Figure 2a, entry 2). Changing to higher energy blue light or higher intensity red light led to higher conversions, but low yields (Figure 2a, entries 3 and 4, respectively). We attribute the loss in mass balance to either photochemically mediated decomposition pathways (in the case of blue light) or to higher temperatures in the reaction mixture and at the PTA surface that accelerate thermal decomposition (in the case of higher intensity red light). Notably, irradiation with low‐intensity red light (660 nm) reduced rates of aryl halide and product decomposition, enabling near‐quantitative yield with longer reaction times of 24 h (Figure 2a, entry 5). In the absence of carbon black, no conversion due to background heating or photoreactivity occurred, emphasizing the necessity of photothermal heating (Figure 2a, entry 6). Exclusion of base still resulted in product formation, albeit in reduced yield (Figure 2a, entry 7). Other bases such as 1,5,7‐triazabicyclo [4.4.0] dec‐5‐ene (TBD) and 4‐dimethylaminopyridine (DMAP) were also able to promote the reaction effectively (Table S1), but we moved forward with DBU due to its low cost and greater compatibility with other substrates tested. We also found that replacing carbon black with 10% w/w palladium on carbon resulted in no significant increase in yield (Figure 2a, entry 8). Further, ICP‐MS analysis revealed low concentrations of Pd in the carbon black (<1 ppm), supporting that this process is not transition metal catalyzed (see Supporting Information for further details).

We next expanded our scope to substrates that possess moderately high computed activation energy barriers of up to 38.3 kcal/mol (Figure 2b). Shifting the trifluoromethyl group to the *meta* position provided product **2a**, albeit in lower yield due to the less activated arene and the resulting longer reaction time. Steric hindrance similarly reduced the yield of **3a**. Cyano substitution at the *para‐*, *meta‐*, and *ortho*‐positions afforded amination in good to excellent yields, including examples with nucleofuges Cl and F (**4a–6a**). Other resonant withdrawing groups, such as the amide in **7a** also proved sufficiently activating for this system. The alkyne and ester containing **8a** and **9a** performed more poorly due to conjugate addition to the alkyne and nucleophilic addition to the ester, respectively. Nitrogen‐containing heterocycles were well tolerated, producing arylamines (**10a–12a**) in near quantitative yields.

Our conditions also proved amenable to the arylation of several amines (Figure 2c). While largely limited to cyclic secondary amines with minimal steric hindrance, many pharmaceutically relevant saturated *N*‐heterocycles^[^ ^43^, ^44^ ^]^ could be arylated in good to excellent yields (**1b–d**). Primary and acyclic amines were less efficient at substitution, forming **1e** and **1f** in 29% and 51% yield, respectively, due to long reaction times necessitated by their lower nucleophilicity and increased steric hindrance. Additionally, free alcohols were reasonably tolerated to form **1g**. Excitingly, we were able to perform the reaction of 2‐chlorobenzonitrile with piperidine at a 2 g scale in excellent yield (94%) without modification except an extended reaction time (Figure 2d).

**Figure 3:**
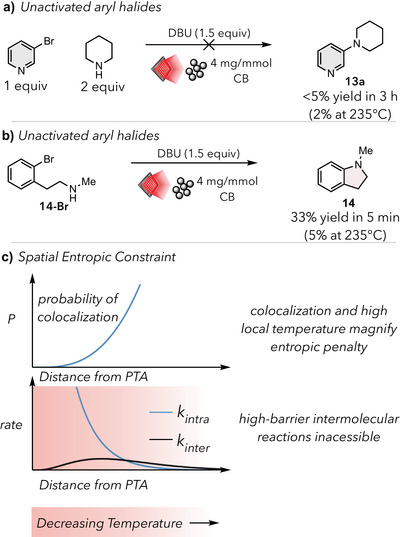
Challenges associated with intramolecular photothermally mediated reactivity, comparing a) the reaction of 3‐bromopyridine with piperidine and b) the intramolecular reaction of **14‐Br** under analogous conditions. c) temperature gradient as a function of distance from carbon black, impacting reaction rate.

Efforts to apply this method to a less activated electrophile, such as 3‐bromopyridine **13** offered little to no product formation (Figure 3a). Additional equivalents of base, more intense light, and extended reaction times resulted in competitive starting material decomposition over C─N bond formation to **13a** A key difference we identified was the intermolecular vs. intramolecular nature of these transformations. The concerted nature of these S_N_Ar reactions puts a significant entropic constraint on the transformation, with a highly ordered transition state.^[^ ^31^, ^32^, ^33^, ^39^ ^]^ As the entropic contribution to a reaction's energy barrier increases with temperature, we hypothesized that bimolecular reactions present a particular challenge to photothermal reactivity as colocalization of reacting molecules becomes increasingly unfavorable at high temperatures. Due to the highly localized heat generated by the carbon black, it would necessitate the presence of both coupling partners in proximity to the particle, generating an additional entropic constraint on the transformation and limiting the ability to perform productive chemistry.

**Figure 4:**
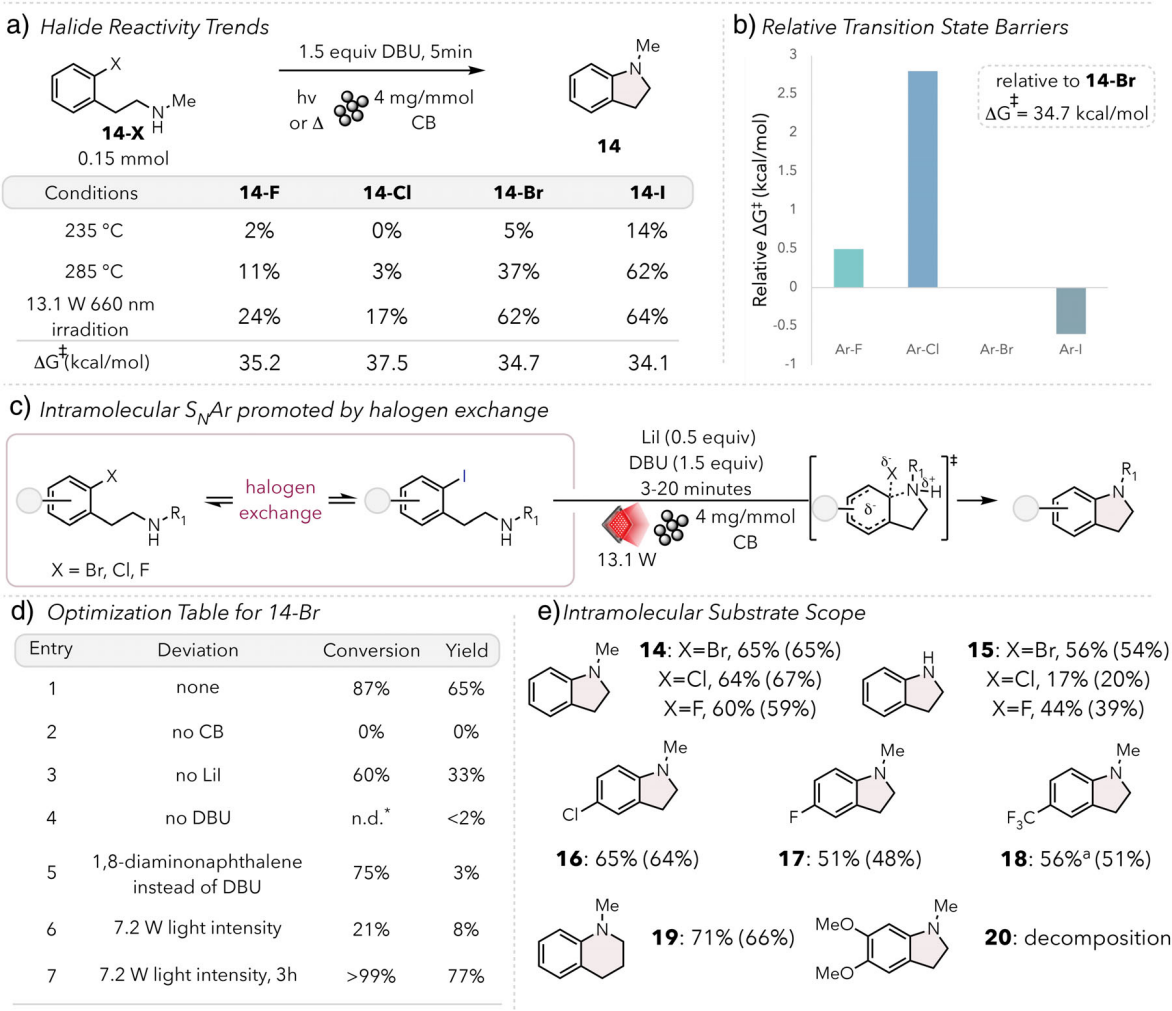
a) Comparison across halogens for intramolecular S_N_Ar under bulk heating and photothermal conditions b) Comparison of transition state barriers across halogens for intramolecular cyclization. c) Intramolecular S_N_Ar with halogen exchange mechanism d) Intramolecular S_N_Ar optimization table for 2‐(2‐bromophenyl)‐*N*‐methylethan‐1‐amine run for 5 min on a 13.1 W 660 nm LED unless otherwise specified. Yields were calculated using ^1^H‐NMR using 1,3,5‐trimethoxybenzene as an internal standard. e) Intramolecular substrate scope. Yields were calculated using ^1^H‐NMR using 1,3,5‐trimethoxybenzene as an internal standard. Isolated yields in parentheses. *^a^* Yield obtained from reactions run on a 7.5 W 660 nm LED for 3 min for substrate 18. n.d. – not detected due to significant overlapping peaks.

To test our hypothesis, we turned to an analogous electron‐neutral electrophile with a tethered amine nucleophile, **14‐Br** (Figure 3b). This substrate was calculated to be even more enthalpically challenging (*ΔH* ^‡^ = 29.9 kcal/mol) compared to the reaction of 3‐bromopyridine and piperidine (*ΔH* ^‡^ = 27.4 kcal/mol) but would be much less susceptible to the entropic penalty present in the intermolecular reaction. To our delight, initial studies revealed that the cyclization to form the corresponding *N*‐methyl indoline product **14** in 33% yield occurred in just 5 min (Figure 4b, entry 3). This contrasts starkly with the formation of **13a** which exhibited only trace yields even on much longer time scales. These results emphasize the entropically challenging nature of photothermal reactions. At lower temperatures (e.g., in the bulk reaction mixture), the two transformations present similar energetic challenges. At shorter distances from the PTA, however, extreme temperatures dictate that the entropic penalty for a bimolecular reaction increase even as the colocalization of both reagents at such a narrow radius becomes more unlikely (Figure 3c). This presents an entropic barrier above and beyond what would be encountered in homogeneous thermal reactions and effectively prohibits the application of photothermal conversion to very high barrier bimolecular reactions.^[^ ^36^, ^45^ ^]^

Excited by the intramolecular success, we further investigated the reaction parameters. During our photothermal screenings, we observed a general reactivity trend wherein Ar–I > Ar –Br > Ar– F > Ar–Cl, with Ar–F and Ar–Cl performing significantly worse (Figure 4a). To rule out any photochemical contributions, we further investigated this trend thermally and computationally. At 235 °C, the fluoro‐ and bromo‐ substrates exhibited <5%, whilst the chlorinated substrate did not form any of the desired product. By contrast, in just 5 min, the iodinated substrate resulted in 14% yield. A similar trend was observed at 285 °C, where Ar–I > Ar–Br > Ar–F > Ar–Cl. This trend was also consistent with our DFT calculations, which showed the lowest barrier for Ar–I (34.1 kcal/mol), followed by Ar–Br (34.7 kcal/mol), Ar–F (35.2 kcal/mol), and Ar–Cl (37.5 kcal/mol). Excitingly, we found that photothermal conversion was able to produce the highest yields across all substrates. With the exception of the iodinated substrate, the yield for the photothermal conditions is nearly doubled in comparison to the yields obtained via bulk heating at 285 °C. These results demonstrate the high barriers made easily accessible via photothermal conversion under mild, visible light irradiation.

We next questioned if the reactivity trend amongst halides could be leveraged to improve our system, as halogen generality is a current challenge for both S_N_Ar and metal‐mediated cross couplings.^[^ ^46^, ^47^, ^48^, ^49^, ^50^, ^51^, ^52^, ^53^ ^]^ The inclusion of LiI in the reaction of the bromoarene increased the yield of product from14% to 65% (Figure 4c, entry 1). Given previous reports and the lower calculated energy barrier for the aryl iodide substitution (34.1 kcal/mol), we surmised that this effect could result from a halogen exchange before the C─N bond‐forming step.^[^ ^54^ ^]^ To support the proposed halogen exchange pathway, we performed a series of experiments where various metal halide salts were added to 4‐bromobenzotrifluoride and subjected to our photothermal conditions in the absence of nucleophile (Figure S2). Using ^19^F‐NMR, we were able to track the formation of 4‐iodobenzotrifluoride (more details in Supporting Information), confirming its formation in cases where an iodide species (TBAI, LiI) was present. Inclusion of LiI in the intermolecular reactions did not provide any significant increase in yield (see Supporting Information for more details), and all transformations reported in Figure 2 were performed in the absence of LiI.

We found that exclusion of CB from the reaction shut down reactivity due to the lack of photothermal heating (Figure 4d, entry 2). The exclusion of DBU led to only trace yield and significant starting material degradation (Figure 4d, entry 4). Changing the base from DBU to another strong organic base,1,8‐diaminonaphthalene (Figure 4d, entry 5) produced only trace yield, likely resulting from significant starting material degradation. Additionally, a decrease in light intensity (13.1 W vs. 7.5 W) dramatically decreased yield from 65% to 8% (Figure 4d, entry 6). This finding is consistent with our previous work on the NKR, where light intensity can be used as a handle for modulating photothermal output (see Figure S2 for a thorough analysis).^[^ ^10^ ^]^

Gratifyingly, the halogen exchange provided improved yields for aryl fluorides and chlorides to greater than 60% yield (Figure 4c). When switching to the primary amine, lower yields were observed (15) despite complete conversion, likely due to competitive elimination of the amine to form styrene. These results were in line with the DFT‐calculated transition state barriers for the various halides, where Ar–Cl possessed a barrier ∼3 kcal/mol greater than the corresponding Ar–Br and Ar–F.

Next, we examined the effect of substitution on the aryl ring as well as the length of the tethered amine nucleophile. These transformations were carried out under ambient conditions in 3‐ 20 min using mild red‐light irradiation, underscoring the ability of photothermal conversion to perform challenging reactions in a facile and schematically simple manner. The inductive electron‐withdrawing effect present in substrates **16** and **17** lowered the reaction time from 5 to 3 min, demonstrating the incredibly facile nature of these transformations. The presence of a trifluoromethyl group at the *para*‐position (**18**), which possesses an even greater inductive electron‐withdrawing effect, enabled the use of a lower intensity light (7.5 W 660 nm LED). The *N*‐methyl‐1,2,3,4‐tetrahydroquinoline product (**19**) readily formed in 66% yield, providing access to the 6‐membered ring. Notably, the addition of methoxy groups at the 3‐ and 4‐ positions resulted in starting material decomposition and did not afford any cyclized product (**20**). These transformations were carried out under ambient conditions in 3–20 min using mild red‐light irradiation, underscoring the ability of photothermal conversion to perform challenging reactions in a facile and schematically simple manner.

**Figure 5:**
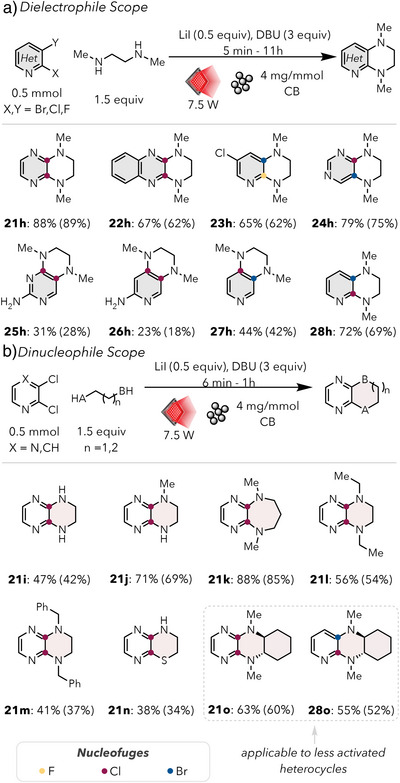
a) Electrophile scope for sequential S_N_Ar reaction with *N, N*‐dimethyl ethylene diamine. Yields were calculated using ^1^H‐NMR using 1,3,5‐trimethoxybenzene as an internal standard. Isolated yields in parentheses. b) Nucleophile scope for sequential S_N_Ar reaction with either 2,3‐dichloropyrazine or 3‐bromo‐2‐chloropyridine. Yields were calculated using ^1^H‐NMR using 1,3,5‐trimethoxybenzene as an internal standard. Isolated yields in parentheses.

Inspired by our success with activated intermolecular S_N_Ar and unactivated intramolecular S_N_Ar, we hypothesized that a sequential approach could overcome the entropic penalty associated with more challenging reactions. Using dielectrophilic arenes, an initial facile displacement would occur at the more activated position. Then, the entropic confinement allows for a secondary, more challenging S_N_Ar reaction closer to the PTA surface. To our delight, we found that this technique proved amenable to several dielectrophiles, including pyridines, pyrazines, and pyrimidines (Figure 5a). The dichloropyrazines, which possess a highly activated initial site of attack, formed the corresponding fused heterocycles in good yields (**21h** and **22h**). We found the tandem nature of the S_N_Ar to work favorably for multi‐halogenated heterocycles, where the Cl in the 5‐position on **23h** remained untouched while the fused heterocycle formed in 62% yield. This system also tolerated a moderate degree of electron density on the dielectrophile ring, with substrates **25h** and **26h** containing a free amine group para to the second substitution site, albeit in 31% and 23% yield respectively.

We additionally investigated several dinucleophiles and found that unhindered secondary diamines performed optimally (Figure 5b). The primary diamine in **21i**, for example, produced just over half the yield formed by the *N1*‐methylethane‐1,2‐diamine used for **21j**. This is easily attributed to the relative nucleophilicity of the amines. We were able to form the seven‐membered ring for substrate **21k** in 85% yield, demonstrating the ability to form less enthalpically favored ring sizes. Among the dinucleophiles tested, we found that bulky groups at the nitrogen resulted in no/trace product formation, with the *N,N*‐diethyl ethylene diamine used in substrate **21l** containing the most steric bulk. Additionally, the use of *N*,*N*‐dibenzylethane‐1,2‐diamine produced 41% yield of heterocycle **21m**. An asymmetric dinucleophile, 2‐aminoethane‐1‐thiol, was also used as a productive nucleophile for **21n**. The asymmetry in dinucleophiles present in **21j** and **21n** results in the first addition being performed by the more nucleophilic species, with the primary amine performing the cyclization in both cases. A fused cyclohexane ring on the dinucleophile was well tolerated for **21o**, where a slight diminishment in conformational flexibility produced only a small drop in yield. This same nucleophile performed well on the corresponding pyridine, but produced similar yields in **28o**, demonstrating applicability to less activated heterocycles. Reactions proved incredibly facile and were completed in 15–60 min, with the exception of **26h**, which ran for 11 h. This sequential S_N_Ar allows for complex fused heterocycle synthesis under operationally simple conditions using mild, visible light irradiation.

## Conclusion

Our initial interest in aromatic C─N bond formation led us to the discovery that photothermally mediated reactivity operates under an entropic constraint due to inhomogeneous heating. We observed that mildly activated aryl halides and heteroarenes readily undergo intermolecular S_N_Ar via photothermal promotion. Our conditions, which utilized visible light irradiation and a strong organic base, were not readily applicable to unactivated aryl halides. Computations revealed that the relative barriers for these transformations were well within the range achieved in previous work. Applying our conditions to analogous intramolecular substrates, we were able to see productive reactivity in incredibly short time frames (3–20 min), supporting our hypotheses. This perceived limitation was leveraged into a simple approach for forming complex, fused heterocycles. The inter‐, intra‐, and tandem S_N_Ar reactions achieved were performed under solvent‐free conditions, using mild red‐light irradiation. This work demonstrates that photothermal conversion can be used for a diverse array of challenging S_N_Ar reactions to form highly valuable aromatic C─N bonds under simple conditions.

## Supporting Information

The authors have cited additional references within the Supporting Information.^[^ ^55^, ^56^, ^57^, ^58^, ^59^ ^]^ The Supporting Information contains experimental methods and optimization for the reactions. It additionally contains computational parameters, starting material and product characterization data, and their corresponding spectra. (PDF)

## Conflict of Interests

The authors declare no conflict of interest.

## Supporting information



Supporting Information

## Data Availability

The data that support the findings of this study are available in the Supporting Information of this article.
