# Supplementary material for: Photothermal Conversion Promotes Challenging SNAr for Facile C─N Bond Formation
Source: Angew Chem Int Ed Engl. 2026 Jan 18;65(9):e22296. doi: 10.1002/anie.202522296 (PMC12826326; doi:10.1002/anie.202522296)
Supplement: Supplementary file 1 — Supporting Information [file ANIE-65-e22296-s001.pdf]

Supporting Information

Photothermal Conversion Promotes Challenging S_NAr for Facile C–N Bond Formation

Megan E. Matter[‡]*, Rory C. Devin*[‡]*, Erin E. Stache**

Department of Chemistry, Princeton University, Princeton, New Jersey 08544, United States

*Email: estache@princeton.edu

Table of Contents

General Information	S3
Synthesis of Starting Materials for Intermolecular S_NAr	S4
General Procedure A for Photothermal S_NAr of Intermolecular Substrates	S5
Base Screen for S_NAr of Intermolecular Substrates	S5
Effect of LiI on Substrates **1a** and **13a**	S6
Characterization for Intermolecular S_NAr Products	S7
Additional Substrates Tested in Intermolecular System	S14
Procedure for Experiments from Figure 3	S15
Procedure for Synthesis of Starting Materials for Intramolecular S_NAr	S16
Characterization of Starting Materials	S18
General Procedure B for Photothermal S_NAr of Intramolecular Substrates	S21
Characterization for Intramolecular S_NAr Products	S22
Procedure for Experiments from Figure 4a	S25
Halogen Exchange Experiments	S26
General Procedure C for Photothermal One Pot, Two Step S_NAr	S27
Characterization for One Pot, Two Step S_NAr Products	S28
Computational Data	S34
ICP-MS Characterization of Carbon Black, DBU, and LiI	S38
XPS Characterization of Carbon Black Pre and Post Reaction	S41
Isotherm Characterization of Carbon Black	S42
Inter- and intramolecular reactions screening with light intensity	S46
Carbon black loading studies	S48
Spectra	S49
References	S115

General Information

Methods and Materials

Amorphous carbon black was purchased from Alfa Aesar (carbon black, acetylene, 100% compressed) and used as received. Lithium Iodide, trace metals grade (99.9%) was purchased from Oakwood Chemical was analyzed using inductively coupled plasma-mass spectrometry, ICP-MS, and used without further purification. Nuclear magnetic resonance (NMR) spectra were recorded on a Bruker 400 MHz or 500 MHz instrument at room temperature using *d*-chloroform ($CDCl_3$), where solvent resonance functions as an internal standard. ^{1}H NMR, ^{13}C NMR, and ^{19}F NMR are reported in parts per million (ppm) relative to the solvent. Inductively Coupled Plasma-Mass spectrometry (ICP-MS) was performed by MSE Analytical Services using an Agilent 7800 ICP-MS, where experiments were run in triplicates and an average value for the ppm of Fe, Cu, and Pd was determined for the carbon black (CB), lithium iodide (LiI), and 1,8-Diazabicyclo(5.4.0)undec—7-ene (DBU). High resolution mass spectra were obtained with an Agilent 6230 TOF LC/MS. All photochemical reactions were irradiated using TX Aluminum Heatsink Cooling Fan equipped with Chanzon LED chips and wired with an LPC-60-1750 or HLG-120H-36A LED driver purchased from Mean Well. Each piece of the setup was purchased individually from either Amazon or Mouser Electronics and wired manually. Light intensity was measured using the PMD 100D compact power and energy meter equipped with the thermal power sensor head purchased from Thor Labs. Infrared (IR) spectra were acquired on an Agilent Cary 630 FTIR spectrometer. X-Ray photoelectric spectroscopy for pre- and post-reaction carbon black analysis was performed by Princeton's Imaging and Analysis Center using the Thermo Nexsa G2 X-ray Photoelectron Spectrometer. The specific surface area and pore size distribution of carbon black was measured using an ASAP 2020 Plus 2.00 adsorption analyzer under a Nitrogen atmosphere at 77K.

Synthesis of Starting Materials for Intermolecular S_NAr :2-bromo-*N*-phenylbenzamide (7)

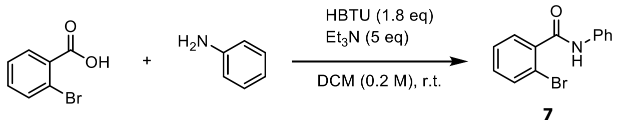

7

In a 100 mL round bottom flask under nitrogen, 2-bromobenzoic acid (500 mg, 2.49 mmol, 1 equiv) was dissolved in DCM (10 mL). Et_3N (1.73 mL, 12.44 mmol, 5 equiv) was added, followed by aniline (0.34 mL, 3.73 mmol, 1.5 equivalents) and *O*-(Benzotriazol-1-yl)-*N,N,N',N'*-tetramethyluronium hexafluorophosphate (HBTU, 1.7g, 4.48 mmol, 1.8 equiv). The mixture was stirred at room temperature overnight. The mixture was diluted with water and DCM, and the aqueous layer was extracted with DCM thrice. The combined organic layers were washed with brine, dried over MgSO4, and concentrated in vacuo. The crude yellow solid was purified on silica (10% EtOAc in hexanes) to afford the desired product as a white solid (620 mg, 85% yield). Analytical data agreed with previous reports.

^{1}H NMR (500 MHz, $CDCl_3$) δ 7.70 – 7.61 (m, 5H), 7.40 (ddd, J = 16.2, 8.5, 7.4 Hz, 3H), 7.33 (td, J = 7.7, 1.8 Hz, 1H), 7.21 – 7.15 (m, 1H).

^{13}C NMR (126 MHz, $CDCl_3$) δ 165.44, 137.80, 137.47, 133.55, 131.69, 129.88, 129.15, 127.78, 124.90, 120.05, 119.23

General Procedure A for Photothermal S_NAr of Intermolecular Substrates

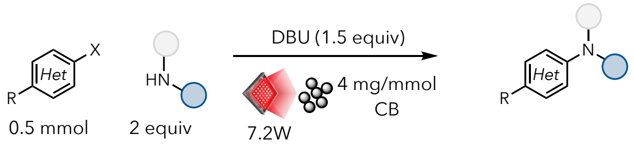

(hetero)Aryl halide (0.5 mmol), carbon black (2 mg, 4 mg/mmol), DBU (112 ul, 0.75 mmol, 1.5 equiv), and amine (1.0 mmol, 2 equiv) were added to a ½ dram vial before capping with a septum cap. This mixture was vortexed briefly and placed directly onto a 7.2W 660 nm LED and irradiated for the specified time. Following irradiation, the reaction is removed from the light, allowed to cool slightly, and the product was isolated via column chromatography or diluted with 1,3,5-trimethoxybenzene solution in $CDCl_3$ for quantitative NMR analysis.

For Scale up: In a 20 mL scintillation vial with a septum cap, 2-chlorobenzonitrile (2 g, 14.5 mmol, 1 equiv) was combined with carbon black (58 mg), DBU (3.32 g, 21.81 mmol, 1.5 equiv), and piperidine (2.48 g, 29.1 mmol, 2 equiv) and mixed with vortexing. The vial was placed directly onto a 7.2W 660 nm LED and irradiated for 9 hours. The crude mixture was filtered over a silica column to afford the pure product as a colorless oil (2.55 g, 94%).

Table S1: Base evaluation in S_NAr of Intermolecular Substrates

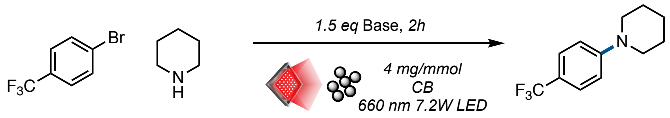

Entry	Base	Conversion[a]	Yield[a]
1	DBU	29%	30%
2	TBD	60%	55%
3	DIPEA	5%	2%
4	DMAP	48%	42%
5	ProtonSponge	10%	7%
6	1,8-diaminonaphthalene	11%	10%

[a]Determined by qNMR vs 1,3,5-trimethoxybenzene, average of 2 trials

Effect of LiI on Substrates 1a and 13a

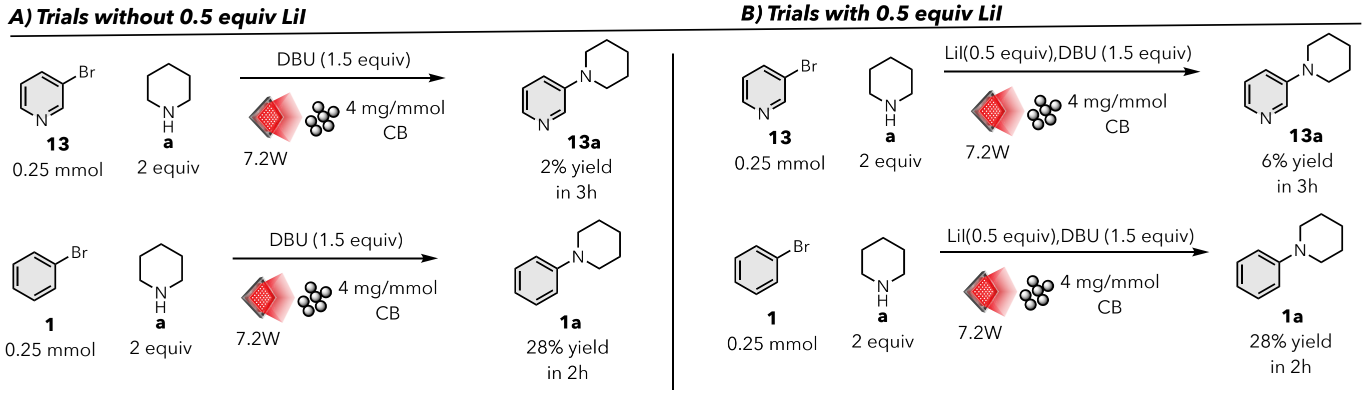

(hetero)Aryl halide (0.5 mmol), carbon black (2 mg, 4 mg/mmol), DBU (112 ul, 0.75 mmol, 1.5 equiv), and amine (1.0 mmol, 2 equiv) were added to a ½ dram vial before capping with a septum cap. In cases where presence of LiI was being determined, LiI (0.5 equiv) was added to the reaction mixture before irradiation. This mixture was vortexed briefly and placed directly onto a 7.2W 660 nm LED and irradiated for the specified time. Following irradiation, the reaction is removed from the light, allowed to cool slightly, and the product was isolated via column chromatography or diluted with 1,3,5-trimethoxybenzene solution in $CDCl_3$ for quantitative NMR analysis.

Characterization for S_NAr of Intermolecular Substrates

1-(4-(trifluoromethyl)phenyl)piperidine (1a)

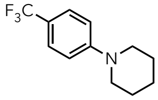

Substrate **1a** was synthesized according to General Procedure **A** using 1-bromo-4-(trifluoromethyl)benzene and piperidine for 24 hours. Following irradiation, the product was purified using column chromatography on silica gel with 5% ethyl acetate in hexanes to afford the pure product as a colorless oil (105 mg, 95%). Analytical data agreed with previous reports.
^{1}H NMR (500 MHz, $CDCl_3$) δ 7.45 (d, J = 8.6 Hz, 2H), 6.91 (d, J = 8.7 Hz, 2H), 3.30 – 3.22 (m, 4H), 1.72 – 1.66 (m, 4H), 1.63 (m, J = 5.3, 1.6 Hz, 2H).
^{13}C NMR (126 MHz, $CDCl_3$) δ 153.79, 126.31 (q, J = 3.8 Hz), 124.87 (q, J = 270.2 Hz), 119.52 (q, J = 32.7 Hz), 114.56, 49.29, 25.41, 24.26.
^{19}F NMR (471 MHz, $CDCl_3$) δ -61.22.

1-(3-(trifluoromethyl)phenyl)piperidine (2a)

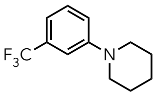

Substrate **2a** was synthesized according to General Procedure **A** using 1-bromo-3-(trifluoromethyl)benzene and piperidine for 64 hours. Following irradiation, the product was purified using column chromatography on silica gel with 5% ethyl acetate in hexanes to afford the pure product as a colorless oil (71 mg, 62%). Analytical data agreed with previous reports.
^{1}H NMR (500 MHz, $CDCl_3$) δ 7.32 (t, J = 8.0 Hz, 1H), 7.12 (t, J = 2.1 Hz, 1H), 7.06 (dd, J = 8.4, 2.5 Hz, 1H), 7.03 (d, J = 7.6 Hz, 1H), 3.23 – 3.18 (m, 4H), 1.71 (p, J = 5.7 Hz, 4H), 1.64 – 1.57 (m, 2H).
^{13}C NMR (126 MHz, $CDCl_3$) δ 152.14, 131.29 (q, J = 31.4 Hz), 129.39, 123.91 (q, J = 272.5 Hz), 119.09, 115.13, 112.45, 112.41, 50.10, 30.90, 25.60, 24.15.
^{19}F NMR (471 MHz, $CDCl_3$) δ -62.72.

1-(2-(trifluoromethylphenyl)piperidine (3a)

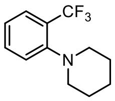

Substrate **3a** was synthesized according to General Procedure **A** using 1-bromo-2-(trifluoromethyl)benzene and piperidine for 24 hours. Following irradiation, the product was

purified using column chromatography on silica gel with 1% ethyl acetate in hexanes to afford the pure product as a colorless oil (66 mg, 58%).
^{1}H NMR (500 MHz, $CDCl_3$) δ 7.60 (dd, J = 7.9, 1.6 Hz, 1H), 7.48 (td, J = 7.7, 1.6 Hz, 1H), 7.33 (d, J = 8.0 Hz, 1H), 7.17 (t, J = 7.6 Hz, 1H), 2.88 – 2.82 (m, 4H), 1.74 – 1.66 (m, 4H), 1.60 – 1.51 (m, 2H).
^{13}C NMR (126 MHz, $CDCl_3$) δ 153.95, 132.52, 127.13 (q, *J* = 5.3 Hz), 127.0 (q, *J* = 28.5 Hz), 124.07, 123.76, 123.65 (q, *J* = 272.2 Hz), 55.00, 26.39, 24.23.
^{19}F NMR (471 MHz, $CDCl_3$) δ -60.49.
ESI: calculated 229.1073 (M^+), found 229.1030

4-(piperidin-1-yl)benzonitrile (4a)

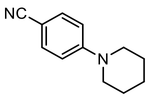

Substrate **4a** was synthesized according to General Procedure **A** using 4-bromobenzonitrile and piperidine for 5 minutes. Following irradiation, the product was purified using column chromatography on silica gel with 1% ethyl acetate in hexanes to afford the pure product as a colorless oil (90 mg, 97%). Analytical data agreed with previous reports.
^{1}H NMR (500 MHz, $CDCl_3$) δ 7.50 – 7.41 (m, 2H), 6.84 (d, *J* = 9.1 Hz, 2H), 3.33 (t, *J* = 4.8 Hz, 4H), 1.71 – 1.59 (m, 6H).
^{13}C NMR (126 MHz, $CDCl_3$) δ 153.55, 133.47, 120.34, 114.05, 98.97, 48.43, 25.22, 24.22.

3-(piperidin-1-yl)benzonitrile (5a)

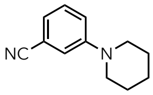

Substrate **5a** was synthesized according to General Procedure **A** using 3-bromobenzonitrile and piperidine for 6 hours. Following irradiation, the product was purified using column chromatography on silica gel with 10% ethyl acetate in hexanes to afford the product as a colorless oil (60 mg, 64%). Analytical data agreed with previous reports.
^{1}H NMR (500 MHz, $CDCl_3$) δ 7.28 (dd, *J* = 9.3, 7.5 Hz, 1H), 7.13 – 7.08 (m, 2H), 7.05 – 7.01 (m, 1H), 3.23 – 3.16 (m, 4H), 1.74 – 1.65 (m, 4H), 1.61 (m, *J* = 5.7, 1.6 Hz, 2H).
^{13}C NMR (126 MHz, $CDCl_3$) δ 151.91, 129.73, 121.73, 120.16, 119.56, 118.58, 112.86, 49.66, 25.42, 24.08.

2-(piperidin-1-yl)-benzonitrile (6a)

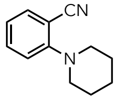

Substrate **6a** was synthesized according to General Procedure **A** using 2-fluorobenzonitrile and piperidine for 2 hours. Following irradiation, the product was purified using column

chromatography on silica gel with 10% ethyl acetate in hexanes to afford the pure product (93 mg, 99% yield) as a colorless oil.
X=Cl: Substrate **1e** was synthesized by irradiation of 2-chloro for 2.5 hours to afford the pure product (86 mg, 93% yield) as a colorless oil. Analytical data agreed with previous reports.
^{1}H NMR (500 MHz, $CDCl_3$) δ 7.54 (dd, *J* = 7.7, 1.6 Hz, 1H), 7.44 (ddd, *J* = 8.9, 7.4, 1.7 Hz, 1H), 6.99 (d, *J* = 8.4 Hz, 1H), 6.97 – 6.92 (m, 1H), 3.20 – 3.12 (m, 4H), 1.78 (m, *J* = 5.8 Hz, 4H), 1.60 (m, *J* = 6.1 Hz, 2H).
^{13}C NMR (126 MHz, $CDCl_3$) δ 156.88, 134.26, 133.55, 120.98, 118.67, 118.65, 105.85, 53.11, 26.08, 24.03.

_N_-phenyl-2-(piperidin-1-yl)benzamide (7a)

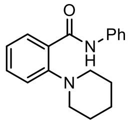

Substrate **7a** was synthesized according to General Procedure **A** using 2-bromo-*N*-phenylbenzamide and piperidine for 6 hours. Following irradiation, the product was purified using column chromatography on silica gel with 10% ethyl acetate in hexanes to afford the pure product (116 mg, 83% yield) as a white solid.
^{1}H NMR (500 MHz, $CDCl_3$) δ 12.55 (s, 1H), 8.30 (dd, *J* = 7.8, 1.7 Hz, 1H), 7.84 – 7.73 (m, 2H), 7.48 (ddd, *J* = 8.1, 7.3, 1.8 Hz, 1H), 7.37 (dd, *J* = 8.5, 7.3 Hz, 2H), 7.32 – 7.26 (m, 2H), 7.14 – 7.08 (m, 1H), 3.02 (t, *J* = 5.4 Hz, 4H), 1.84 (p, *J* = 5.7 Hz, 4H), 1.67 (d, *J* = 6.0 Hz, 2H).
^{13}C NMR (126 MHz, $CDCl_3$) δ 164.28, 152.25, 139.04, 132.32, 131.72, 129.11, 127.94, 125.18, 123.66, 120.95, 119.83, 55.07, 26.64, 23.70.
HRMS: calculated 281.1648 $(M+H)^+$; found 281.2246

1-(4-((trimethylsilyl)ethynyl)phenyl)piperidine (8a)

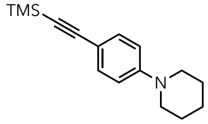

Substrate **8a** was synthesized according to General Procedure **A** using 4-trimethylsilylethynyl bromobenzene and piperidine for 2 hours. Following irradiation, the product was purified using column chromatography on silica gel with 5% ethyl acetate in hexanes to afford the pure product as a yellow solid (16 mg, 12%).
^{1}H NMR (500 MHz, $CDCl_3$) δ 7.34 (d, *J* = 8.8 Hz, 2H), 6.80 (d, *J* = 8.9 Hz, 2H), 3.24 – 3.15 (m, 4H), 1.73 – 1.63 (m, 4H), 1.59 (q, *J* = 6.5 Hz, 2H), 0.23 (s, 9H).
^{13}C NMR (126 MHz, $CDCl_3$) δ 151.50, 132.87, 114.90, 112.07, 105.97, 91.47, 49.41, 25.37, 24.13.
ESI: calculated $(M+H)^+$: 258.1673, found: 258.2050

5-(piperidin-1-yl)-2-benzofuran-1(3H)-one (9a)

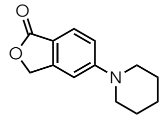

Substrate **9a** was synthesized according to General Procedure **A** using 5-bromoisobenzofuran-1(3*H*)-one and piperidine for 2.75 hours. Following irradiation, the product was purified using column chromatography on silica gel with 20% ethyl acetate in hexanes to afford the pure product as a white solid (37 mg, 34%). Analytical data agreed with previous reports.
^{1}H NMR (500 MHz, $CDCl_3$) δ 7.71 (d, *J* = 8.7 Hz, 1H), 6.97 (dd, *J* = 8.7, 2.2 Hz, 1H), 6.77 (d, *J* = 2.2 Hz, 1H), 5.18 (s, 2H), 3.38 (t, *J* = 5.1 Hz, 4H), 1.73 – 1.62 (m, 6H).
^{13}C NMR (126 MHz, $CDCl_3$) δ 155.62, 149.48, 126.74, 115.68, 113.96, 105.41, 69.17, 49.07, 25.33, 24.29.

5-methyl-2-(piperidin-1-yl)pyridine (10a)

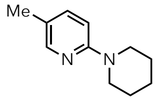

Substrate **10a** was synthesized according to General Procedure **A** using 2-bromo-5-methylpyridine and piperidine for 1 hour. Following irradiation, the product was purified using column chromatography on silica gel with 10% ethyl acetate in hexanes to afford the pure product as a colorless oil (84 mg, 95%). Analytical data agreed with previous reports.
^{1}H NMR (500 MHz, $CDCl_3$) δ 8.00 (d, *J* = 2.4 Hz, 1H), 7.31 – 7.22 (m, 1H), 6.59 (d, *J* = 8.6 Hz, 1H), 3.45 (dd, *J* = 5.6, 3.4 Hz, 4H), 2.17 (s, 3H), 1.69 – 1.57 (m, 6H).
^{13}C NMR (126 MHz, $CDCl_3$) δ 158.49, 147.65, 138.27, 121.42, 107.13, 46.85, 25.52, 24.74, 17.31.

3-methyl-2-(piperidin-1-yl)pyridine (11a)

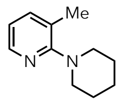

Substrate **11a** was synthesized according to General Procedure **A** using 2-bromo-3-methylpyridine and piperidine for 2 hours. Following irradiation, the product was purified using column chromatography on silica gel with 10% ethyl acetate in hexanes to afford the pure product as a colorless oil (81 mg, 92%)). Analytical data agreed with previous reports.
^{1}H NMR (500 MHz, $CDCl_3$) δ 8.17 – 8.12 (m, 1H), 7.39 – 7.35 (m, 1H), 6.81 (dd, *J* = 7.3, 4.9 Hz, 1H), 3.10 – 3.02 (m, 4H), 2.26 (s, 3H), 1.70 (p, *J* = 5.7 Hz, 4H), 1.64 – 1.56 (m, 2H).
^{13}C NMR (126 MHz, $CDCl_3$) δ 162.82, 145.20, 139.00, 125.11, 117.39, 50.96, 26.29, 24.60, 18.28.

3-(piperidine-1-yl)pyrazine (12a)

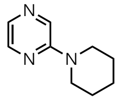

Substrate **12a** was synthesized according to General Procedure **A** using 2-chloropyrazine and piperidine for 0.5 hour. Following irradiation, the product was purified using column chromatography on silica gel with 20% ethyl acetate in hexanes to afford the pure product as a colorless oil (79 mg, 97%). Analytical data agreed with previous reports.
^{1}H NMR (500 MHz, $CDCl_3$) δ 8.12 (d, *J* = 1.5 Hz, 1H), 8.02 (d, *J* = 1.1 Hz, 1H), 7.76 (d, *J* = 2.7 Hz, 1H), 3.57 (m, *J* = 6.2, 4.1 Hz, 4H), 1.65 (m, *J* = 8.3, 5.4, 3.0 Hz, 6H).
^{13}C NMR (126 MHz, $CDCl_3$) δ 155.11, 141.67, 131.94, 131.13, 45.55, 25.32, 24.50.

1-(4-trifluoromethylphenyl)pyrrolidine (1b)

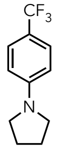

Substrate **1b** was synthesized according to General Procedure **A** using 4-trifluoromethyl bromobenzene and pyrrolidine for 1.25 hours. Following irradiation, the product was purified using column chromatography on silica gel with 10% ethyl acetate in hexanes to afford the pure product as a white solid (86 mg, 80%). Analytical data agreed with previous reports.
^{1}H NMR (500 MHz, $CDCl_3$) δ 7.48 – 7.38 (m, 2H), 6.55 (d, *J* = 8.7 Hz, 2H), 3.37 – 3.26 (m, 4H), 2.09 – 1.98 (m, 4H).
^{13}C NMR (126 MHz, $CDCl_3$) δ 149.73, 126.40, 126.37 (q, *J* = 3.8 Hz), 125.33 (q, *J* = 270 Hz), 116.66 (q, *J* = 32.3 Hz), 110.82, 47.51, 25.45.
^{19}F NMR (471 MHz, $CDCl_3$) δ -60.62.

1-methyl-4-(4-(trifluoromethyl)phenyl)piperazine (1c)

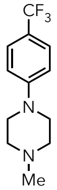

Substrate **1c** was synthesized according to General Procedure **A** using 4-trifluoromethylbromobenzene and 1-methylpiperazine for 16 hours. Following irradiation, the product was purified using column chromatography on silica gel with 10% ethyl acetate in hexanes to afford the pure product as a white solid (98 mg, 80%). Analytical data agreed with previous reports.
^{1}H NMR (500 MHz, $CDCl_3$) δ 7.52 – 7.41 (m, 2H), 6.92 (d, *J* = 8.7 Hz, 2H), 3.35 – 3.23 (m, 4H), 2.61 – 2.50 (m, 4H), 2.36 (s, 3H).
^{13}C NMR (126 MHz, $CDCl_3$) δ 153.22, 126.38 (q, *J* = 3.72 Hz), 124.72 (q, *J* = 270.7 Hz), 120.52 (q, *J* = 32.8 Hz), 114.53, 54.78, 47.88, 46.07.
^{19}F NMR (471 MHz, $CDCl_3$) δ -61.37.

4-(4-(trifluoromethyl)phenyl)morpholine

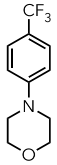

Substrate **1d** was synthesized according to General Procedure **A** using 4-trifluoromethylbromobenzene and morpholine for 7 hours. Following irradiation, the product was purified using column chromatography on silica gel with 10% ethyl acetate in hexanes to afford the pure product as a white solid (103 mg, 89%). Analytical data agreed with previous reports.
^{1}H NMR (500 MHz, $CDCl_3$) δ 7.50 (d, *J* = 8.4 Hz, 2H), 6.97 – 6.87 (m, 2H), 3.90 – 3.81 (m, 4H), 3.30 – 3.19 (m, 4H).
^{13}C NMR (126 MHz, $CDCl_3$) δ 153.33, 126.44 (q, *J* = 3.8 Hz), 271.3 (q, *J* = 271.3 Hz), 121.02 (q, *J* = 32.8 Hz), 114.31, 66.63, 48.17.
^{19}F NMR (471 MHz, $CDCl_3$) δ -61.44.

***N*-octyl-4-(trifluoromethyl)aniline (1e)**

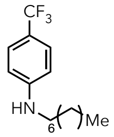

Substrate **1e** was synthesized according to General Procedure **A** using 4-trifluoromethylbromobenzene and octylamine for 45 hours. Following irradiation, the product was purified using column chromatography on silica gel with 5% ethyl acetate in hexanes to afford the pure product as a colorless oil (38 mg, 28%). Analytical data agreed with previous reports.
^{1}H NMR (400 MHz, $CDCl_3$) δ 7.39 (d, *J* = 8.4 Hz, 2H), 6.63 – 6.52 (m, 2H), 3.94 (s, 1H), 3.13 (m, *J* = 7.1, 3.9 Hz, 2H), 1.69 – 1.55 (m, 2H), 1.46 – 1.18 (m, 10H), 0.94 – 0.82 (m, 3H).
^{13}C NMR (101 MHz, $CDCl_3$) δ 126.58, 126.54, 111.63, 43.52, 31.79, 29.34, 29.31, 29.22, 27.07, 22.64, 14.07.
^{19}F NMR (376 MHz, $CDCl_3$) δ -60.95.

N-benzyl-N-methyl-4-(trifluoromethyl)aniline (1f)

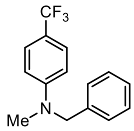

Substrate **1f** was synthesized according to General Procedure **A** using 4-trifluoromethylbromobenzene and N-benzylmethylamine for 16 hours. Following irradiation, the product was purified using column chromatography on silica gel with 5% ethyl acetate in hexanes to afford the pure product as a colorless oil (82 mg, 62%). Analytical data agreed with previous reports.

^{1}H NMR (400 MHz, $CDCl_3$) δ 7.43 (d, *J* = 8.9 Hz, 2H), 7.37 – 7.30 (m, 2H), 7.30 – 7.23 (m, 1H), 7.22 – 7.16 (m, 2H), 6.73 (d, *J* = 8.6 Hz, 2H), 4.60 (s, 2H), 3.11 (s, 3H).
^{13}C NMR (101 MHz, $CDCl_3$) δ 151.58, 137.89, 128.74, 127.15, 126.47 (q, *J* = 3.85 Hz), 125.10 (q, *J* = 270.1 Hz), 117.81 (q, *J* = 32.7 Hz), 111.20, 56.10, 38.69.
^{19}F NMR (376 MHz, $CDCl_3$) δ -60.88.

1-(4-(trifluoromethyl)phenyl)pyrrolidin-3-ol (1g)

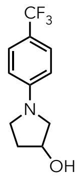

Substrate **1g** was synthesized according to General Procedure **A** using 4-trifluoromethylbromobenzene and pyrrolidin-3-ol for 2 hours. Following irradiation, the product was purified using column chromatography on silica gel with 5% ethyl acetate in hexanes to afford the pure product as a white solid (52 mg, 25%). Analytical data agreed with previous reports.
^{1}H NMR (400 MHz, $CDCl_3$) δ 7.49 – 7.41 (m, 2H), 6.56 (d, *J* = 8.7 Hz, 2H), 4.64 (s, 1H), 3.61 – 3.48 (m, 2H), 3.41 (td, *J* = 8.9, 3.4 Hz, 1H), 3.31 (dt, *J* = 10.6, 1.7 Hz, 1H), 2.19 (dtd, *J* = 13.4, 8.8, 4.7 Hz, 1H), 2.10 (dddd, *J* = 13.1, 6.9, 3.6, 1.4 Hz, 1H), 1.65 (d, *J* = 4.3 Hz, 1H), 1.56 (s, 1H).
^{13}C NMR (101 MHz, $CDCl_3$) δ 149.63, 126.44 (q, *J* = 3.74 Hz), 125.21(q, *J* = 270.15), 117.38 (q, *J* = 32.4), 111.01, 71.11, 56.09, 45.45, 34.15.
^{19}F NMR (376 MHz, $CDCl_3$) δ -60.74.

Additional Substrates

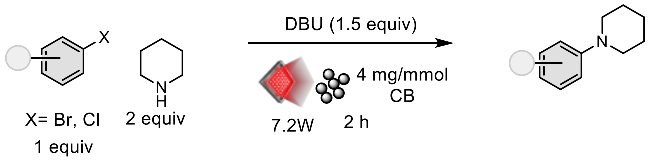

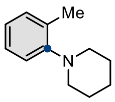

29a: 0%

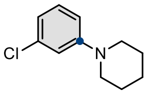

30a: 5%

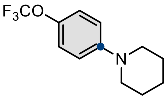

31a: <1%
$\Delta G^{\ddagger}$=50.0 kcal/mol

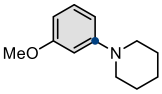

32a: 0%

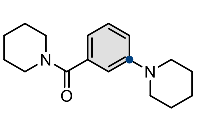

33a: <1%
$\Delta G^{\ddagger}$=50.7 kcal/mol

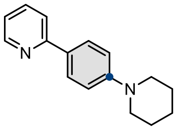

34a: 4%

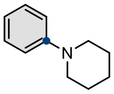

35a: 0%

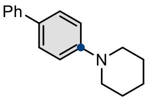

36a: 0%

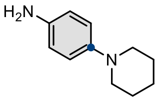

37a: <1%

Figure S1. Additional Substrates tested in the intermolecular system

Procedure for Experiments from Figure 3

Procedure for Figure **3a**

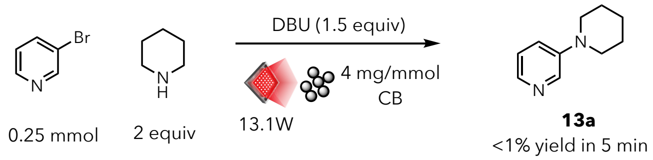

3-Bromopyridine (0.25 mmol), carbon black (1 mg, 4 mg/mmol), DBU (56 ul, 0.375mmol, 1.5 equiv), and piperidine (0.5 mmol, 2 equiv) were added to a ½ dram vial before capping with a septum cap. This mixture was vortexed briefly and placed directly onto a 13.1W 660 nm LED and irradiated for 5 minutes. Following irradiation, the reaction is removed from the light, allowed to cool slightly, and the product is diluted with 1,3,5-trimethoxybenzene solution in $CDCl_3$ for quantitative NMR analysis.

Procedure for Figure **3b**

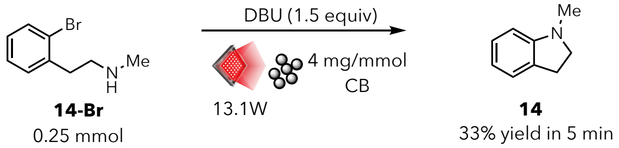

2-(2-bromophenyl)-*N*-methylethan-1-amine (0.25 mmol), carbon black (1 mg, 4 mg/mmol), and DBU (56 ul, 0.375mmol, 1.5 equiv) were added to a ½ dram vial before capping with a septum cap. This mixture was vortexed briefly and placed directly onto a 13.1W 660 nm LED and irradiated for 5 minutes. Following irradiation, the reaction is removed from the light, allowed to cool slightly, and the product is diluted with 1,3,5-trimethoxybenzene solution in $CDCl_3$ for quantitative NMR analysis.

Procedures for Synthesis of Starting Materials

Procedure 1

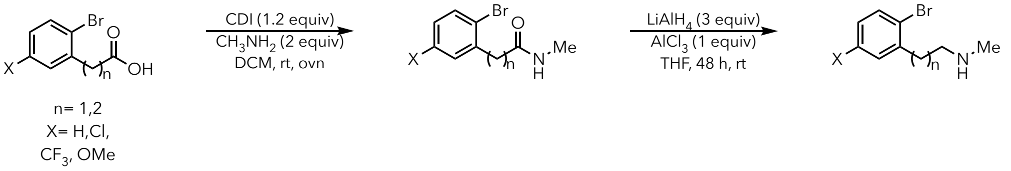

Step 1: Adapted from the procedure provided by Doyle et al.[1] , 2 g (8.73 mmol, 1.0 equiv) of 3-(2-bromophenyl) propionic acid was dissolved in dry DCM (30 mL). 1,1'-carbonyldiimidazole (1.7g, 10.48 mmol, 1.2 equiv) was then added portion wise, after which the reaction was allowed to stir for 1h at room temperature. Following this, the reaction was cooled to 0 °C, and methylamine (2.0 M in THF, 8.7 mL, 17.46 mmol, 2.0 equiv) was added. The reaction was allowed to stir for 16 h at room temperature. Water was added to the reaction mixture and an extraction was performed on the aqueous layer using DCM. The combined organic layers were washed with 1 M HCl and sat. aqueous sodium bicarbonate, dried with magnesium sulfate, before being filtered and concentrated in vacuo. This afforded the corresponding *N*-methylamide in high purity, and the material was used in the next step without additional purification.

Step 2: $LiAlH_4$ (2.0 M solution in THF, 14.7 mL, 26.2 mmol, 3 equiv) was added to an ice cooled round bottom flask containing 20 mL dry THF. Following that, a solution of $AlCl_3$ (1.16 g, 8.73 mmol, 1 equiv) in dry THF (15mL) was slowly added to the flask. The ice bath was removed, and the reaction was allowed to stir at room temperature for 1h before being cooled again to 0 °C, and the *N*-methylamide (2.11g, 8.73 mmol, 1 equiv) in 20mL dry THF was added dropwise to the solution. The reaction was warmed to rt and allowed to stir for 2 days, after which a Fieser workup was performed, and the filter cake was washed extensively with ethyl acetate. This was then concentrated in vacuo before being purified using column chromatography on neutral alumina (0-5% methanol: DCM). This afforded the corresponding amine products in good to moderate yields (45-88% yield).

Cautionary Note: *N*-methylamide product tends to be poorly soluble in THF and diethyl ether, extensive sonication was used to solubilize it before addition to the $LiAlH_4$ solution, however, needle clogs can occur during addition. A slightly larger gauge needle and an excess of solvent is recommended for addition of both the $AlCl_3$ and amide due to these challenges.

Procedure 2

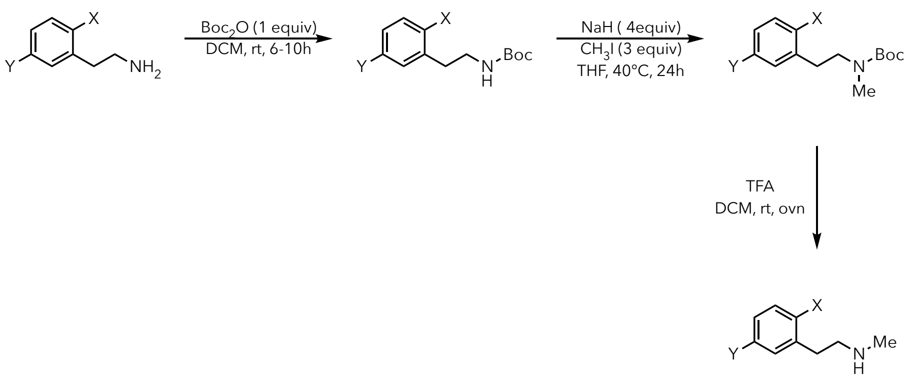

Step 1: Adapted from the procedure provided by Beak et al.[2] ,to a solution of the corresponding amine (9.0 mmol, 1 equiv) in dry CH_2Cl_2 (20 mL) was added di-*t*-butyldicarbonate (2.0 mL, 9.0 mmol, 1 equiv). Addition of the Boc_2O will result in gas evolution, so slow addition and proper venting is recommended to avoid over pressurization of the reaction vessel. The reaction was allowed to stir 6-10 h before the CH_2Cl_2 was removed in vacuo. Purification was performed using column chromatography with silica gel as the stationary phase and a gradient of 0-20% (ethyl acetate: hexane) to afford the product.

Step 2: To a solution of the corresponding *N-Boc-N*-2-(2-bromophenyl)-ethylamine (2.77 g, 8.7 mmol, 1 equivalent) in dry THF was added NaH (60% in mineral oil, 34.8 mmol, 4 equiv). The solution was then allowed to stir for 10-30minutes before CH_3I (1.62 mL, 26.1 mmol, 3 equiv) was added to the reaction mixture. The solution was then heated to 40 °C and allowed to stir for 24 h. When the allotted reaction time had occurred, the reaction was cooled to room temperature, and the excess NaH was quenched via slow addition of water until hydrogen gas evolution had subsided. The reaction mixture was subsequently diluted with brine and extracted 3x with diethyl ether. The combined organics were dried with $MgSO_4$, filtered, and concentrated in vacuo to afford the desired product. No further purification was performed, and the product was used in the next step.

Step 3: To a solution of the corresponding *N-Boc-N*-methyl-*N*-2-(2-bromophenyl) ethylamine (2.5 g, 7.53 mmol, 1 equiv) in 50 mL CH_2Cl_2 was added 5 mL of trifluoroacetic acid. This was allowed to stir at rt overnight, after which the solution was basified with an aqueous 2.0 M NaOH solution. The mixture was repeatedly extracted with CH_2Cl_2, and the combined organics were dried over $MgSO_4$ before being filtered and concentrated in vacuo. In cases where further purification was required, they product was subject to column chromatography on neutral alumina using 0-5% methanol: DCM gradient.

Starting Material Characterization for Intramolecular S_NAr

2-(2-bromophenyl)-*N*-methylethan-1-amine

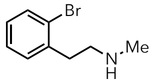

Prepared using Procedure 2 from 2-(2-bromophenyl)acetic acid and purified on neutral alumina using a gradient of 0-5% methanol : DCM to afford 71% product as a yellow oil. Analytical data agreed with previous reports.
^{1}H NMR (500 MHz, $CDCl_3$) δ 7.56 (d, *J* = 8.7 Hz, 1H), 7.31 – 7.25 (m, 2H), 7.15 – 7.08 (m, 1H), 3.21 (s, 1H), 3.06 – 3.00 (m, 2H), 2.98 – 2.89 (m, 2H), 2.55 (s, 3H).
^{13}C NMR (126 MHz, $CDCl_3$) δ 138.65, 132.99, 130.84, 128.16, 127.59, 124.54, 51.00, 35.72, 35.68.

2-(2-chlorophenyl)-*N*-methylethan-1-amine

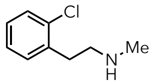

Prepared using Procedure 2 from 2-(2-chlorophenyl)acetic acid and purified on neutral alumina using a gradient of 0-5% methanol : DCM to afford 82% product as a yellow oil. Analytical data agreed with previous reports.
^{1}H NMR (500 MHz, $CDCl_3$) δ 7.42 – 7.34 (m, 1H), 7.27 (d, *J* = 1.9 Hz, 1H), 7.24 – 7.14 (m, 2H), 2.98 (d, *J* = 6.4 Hz, 2H), 2.88 (d, *J* = 14.1 Hz, 2H), 2.49 (s, 3H).
^{13}C NMR (126 MHz, $CDCl_3$) δ 137.63, 134.12, 130.82, 129.60, 127.67, 126.82, 51.41, 36.27, 33.89.

2-(2-fluorophenyl)-*N*-methylethan-1-amine

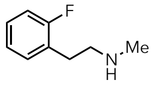

Prepared using Procedure 2 from 2-(2-fluorophenyl)acetic acid and purified on neutral alumina using a gradient of 0-5% methanol : DCM to afford 55% product as an orange oil. Analytical data agreed with previous reports.
^{1}H NMR (500 MHz, $CDCl_3$) δ 7.26 – 7.16 (m, 2H), 7.09 (td, *J* = 7.5, 1.3 Hz, 1H), 7.07 – 7.00 (m, 1H), 2.89 (s, 4H), 2.49 (s, 3H).
^{13}C NMR (126 MHz, $CDCl_3$) δ 162.25, 160.30, 131.01 (d, *J* = 5.0 Hz), 127.98 (d, *J* = 8.1 Hz), 126.66 (dd, *J* = 16.0, 1.5 Hz), 124.05 (d, *J* = 3.6 Hz), 115.35 (d, *J* = 22.1 Hz), 36.06, 29.31 (d, *J* = 1.8 Hz).
^{19}F NMR (471 MHz, $CDCl_3$) δ -75.76, -118.67.

2-(2-iodophenyl)-*N*-methylethan-1-amine

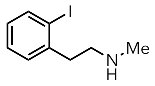

Prepared using Procedure 1 from 2-(2-iodophenyl)acetonitrile and purified on neutral alumina using a gradient of 0-5% methanol : DCM to afford 28% product as a pale yellow oil. Analytical data agreed with previous reports.

^{1}H NMR (500 MHz, $CDCl_3$) δ 7.84 (dd, *J* = 7.9, 1.4 Hz, 1H), 7.35 – 7.20 (m, 2H), 6.98 – 6.84 (m, 1H), 3.01 – 2.91 (m, 2H), 2.85 (d, *J* = 14.6 Hz, 2H), 2.50 (s, 3H).
^{13}C NMR (126 MHz, $CDCl_3$) δ 142.68, 139.63, 129.83, 128.37, 128.05, 100.68, 51.82, 40.95, 36.40.

2-(2-bromo-5-chlorophenyl)-*N*-methylethan-1-amine

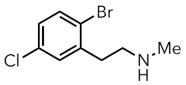

Prepared using Procedure 1 from 2-(2-bromo-5-chlorophenyl)acetic acid and purified on neutral alumina using a gradient of 0-5% methanol : DCM to afford 48% product as a light brown oil. Analytical data agreed with previous reports.

^{1}H NMR (500 MHz, $CDCl_3$) δ 7.56 (s, 1H), 7.27 (d, *J* = 4.5 Hz, 1H), 7.14 – 7.07 (m, 1H), 3.02 – 2.97 (m, 2H), 2.89 (t, *J* = 7.8 Hz, 2H), 2.51 (s, 3H).
13C NMR (126 MHz, $CDCl_3$) δ 139.28, 132.94, 130.80, 127.95, 127.49, 124.59, 51.47, 36.32, 36.23.

2-(2-bromo-5-fluorophenyl)-*N*-methylethan-1-amine

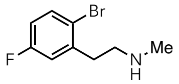

Prepared using Procedure 1 from 2-(2-bromo-5-fluorophenyl)acetic acid and purified on neutral alumina using a gradient of 0-5% methanol : DCM to afford 63% product as an orange oil. Analytical data agreed with previous reports.
^{1}H NMR (500 MHz, $CDCl_3$) δ 7.50 (dd, *J* = 8.7, 5.4 Hz, 1H), 7.00 (dd, *J* = 9.3, 3.1 Hz, 1H), 6.83 (td, *J* = 8.3, 3.1 Hz, 1H), 2.98 – 2.91 (m, 2H), 2.91 – 2.84 (m, 2H), 2.50 (s, 3H).
^{13}C NMR (126 MHz, $CDCl_3$) δ 162.90, 160.94, 141.55 (d, *J* = 7.4 Hz), 133.94 (d, *J* = 8.1 Hz), 118.60 (d, *J* = 3.1 Hz), 117.53 (d, *J* = 22.4 Hz), 115.03 (d, *J* = 22.3 Hz), 51.24, 36.70 – 36.11 (m).
^{19}F NMR (471 MHz, $CDCl_3$) δ -115.11.

2-(2-bromo-5-(trifluoromethyl)phenyl)-*N*-methylethan-1-amine

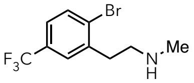

Prepared using general Procedure 1 from 2-(2-bromo-5(trifluoromethyl)phenyl)acetic acid and purified on neutral alumina using a gradient of 0-5% methanol : DCM to afford 33% product as a light brown oil. Analytical data agreed with previous reports.
^{1}H NMR (500 MHz, $CDCl_3$) δ 7.69 (d, *J* = 9.1 Hz, 1H), 7.53 (d, *J* = 2.5 Hz, 1H), 7.36 (dd, *J* = 8.3, 2.7 Hz, 1H), 3.11 – 3.05 (m, 2H), 2.94 (t, *J* = 7.9 Hz, 2H), 2.67 (s, 1H), 2.54 (s, 3H).
^{13}C NMR (126 MHz, $CDCl_3$) δ 140.10, 133.49, 130.17, 128.43 (d, *J* = 1.8 Hz), 127.35 (q, *J* = 3.8 Hz), 124.74 (q, *J* = 3.8 Hz), 122.69, 120.53, 50.90, 35.90.
^{19}F NMR (471 MHz, $CDCl_3$) δ -62.65.

3-(2-bromophenyl)-*N*-methylpropan-1-amine

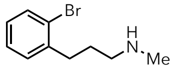

Prepared using general Procedure 1 from 3-(2-bromophenyl)propanoic acid and purified on neutral alumina using a gradient of 0-5% methanol : DCM to afford 88% product as a pale yellow oil. Analytical data agreed with previous reports.

^{1}H NMR (500 MHz, $CDCl_3$) δ 7.55 (d, *J* = 8.3 Hz, 1H), 7.27 – 7.23 (m, 2H), 7.13 – 7.05 (m, 1H), 2.85 – 2.80 (m, 2H), 2.77 (t, *J* = 7.4 Hz, 2H), 2.54 (s, 3H), 1.95 (p, *J* = 7.5 Hz, 2H).
^{13}C NMR (126 MHz, $CDCl_3$) δ 140.80, 132.87, 130.37, 127.77, 127.53, 124.41, 50.71, 35.41, 33.64, 28.85.

General Procedure B for Photothermal S_NAr of Intramolecular Substrates

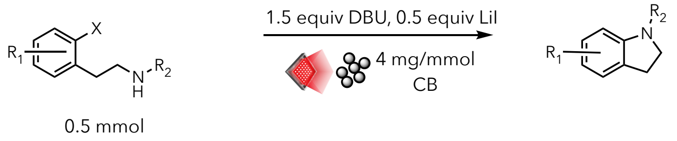

Substrate (0.5 mmol, 1 equiv), carbon black (2 mg, 4 mg/mmol), LiI (33 mg, 0.5 equiv), and DBU (112 ul, 0.75 mmol, 1.5 equiv) are added to a 1 dram vial before being capped with a septum cap and vortexed till mixture appears homogenous. The reaction mixture is then placed directly onto a 13.1 W 660 nm LED and irradiated for the specified time. Following irradiation, the reaction is removed from the light, allowed to cool slightly, and the product is isolated via column chromatography or diluted with 1,3,5-trimethoxybenzene solution in $CDCl_3$ for quantitative NMR analysis.

Characterization Data for Photothermal S_NAr of Intramolecular Substrates

1-methylindoline (14)

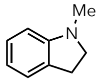

Substrate **14** was synthesized according to General Procedure B using 2-(2-bromophenyl)-*N*-methylethan-1-amine for 5 minutes. Following irradiation, the substrate was purified using column chromatography on silica gel where it was eluted using a gradient of 0-50% DCM: hexanes to afford 43 mg (65%) isolated yield as a brown oil. Analytical data agreed with previous reports.
Note : Substrate **14** was additionally synthesized by irradiation of 2-(2-bromophenyl)-*N*-methylethan-1-amine with 2 mg of CB and 1.5 equivalents of DBU for 3 hours on a 7.2 W 660 nm LED. This produced 77% yield. (See entry 7 from Figure 4D)

X=Cl Substrate **14** was synthesized by irradiation of 2-(2-chlorophenyl)-*N*-methylethan-1-amine for 20 minutes to afford 44.5 mg (67%) isolated yield.

X=F*:* Substrate **14** was synthesized by irradiation of 2-(2-fluorophenyl)-*N*-methylethan-1-amine for 10 minutes to afford 39.7 mg (60%) isolated yield.

^{1}H NMR (500 MHz, $CDCl_3$) δ 7.15 – 7.08 (m, 2H), 6.74 – 6.66 (m, 1H), 6.52 (d, *J* = 8.3 Hz, 1H), 3.32 (t, *J* = 8.2 Hz, 2H), 3.02 – 2.94 (m, 2H), 2.79 (s, 3H).
^{13}C NMR (126 MHz, $CDCl_3$) δ 153.40, 130.32, 127.32, 124.27, 117.79, 107.25, 56.17, 36.30, 28.75.

Indoline (15)

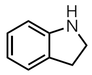

Substrate **15** was synthesized according to General Procedure B using 2-(2-bromophenyl)ethan-1-amine for 10 minutes. Following irradiation, the substrate was purified using column chromatography on silica gel where it was eluted using a gradient of 0-3% acetone: hexanes to afford 32.2 mg (54%) isolated yield as a brown oil. Analytical data agreed with previous reports.

X=Cl: Substrate **15a** was synthesized by irradiation of 2-(2-chlorophenyl)ethan-1-amine for 20 minutes to afford 12.2 mg (20%) isolated yield.

X=F: Substrate **15a** was synthesized by irradiation of 2-(2-fluorophenyl)ethan-1-amine for 15 minutes to afford 23.3 mg (39%) isolated yield.

^{1}H NMR (500 MHz, $CDCl_3$) δ 7.15 (d, *J* = 6.6 Hz, 1H), 7.04 (t, *J* = 7.0 Hz, 1H), 6.77 – 6.70 (m, 1H), 6.68 (d, *J* = 7.9 Hz, 1H), 3.58 (t, *J* = 8.4 Hz, 2H), 3.06 (t, *J* = 8.4 Hz, 2H).
^{13}C NMR (126 MHz, $CDCl_3$) δ 151.60, 129.32, 127.22, 124.65, 118.67, 109.45, 47.34, 29.86.

5-chloro-1-methylindoline (16)

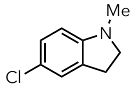

Substrate **16** was synthesized according to General Procedure B using 2-(2-bromo-5-chlorophenyl)-*N*-methylethan-1-amine for 3 minutes. Following irradiation, the substrate was purified using column chromatography on silica gel where it was eluted using a gradient of 0-80% DCM: hexanes to afford 53.5 mg (64%) isolated yield as a pale yellow oil. Analytical data agreed with previous reports.

^{1}H NMR (500 MHz, $CDCl_3$) δ 7.05 (d, *J* = 2.0 Hz, 2H), 6.40 (d, *J* = 8.7 Hz, 1H), 3.35 (t, *J* = 8.2 Hz, 2H), 2.95 (t, *J* = 8.2 Hz, 2H), 2.77 (s, 3H).
^{13}C NMR (126 MHz, $CDCl_3$) δ 151.89, 132.21, 127.01, 124.51, 122.54, 107.88, 56.23, 36.32, 28.52.

5-fluoro-1-methylindoline (17)

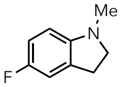

Substrate **17** was synthesized according to General Procedure B using 2-(2-bromo-5-fluorophenyl)-*N*-methylethan-1-amine for 3 minutes. Following irradiation, the substrate was purified using column chromatography on silica gel where it was eluted using a gradient of 0-80% DCM: hexanes to afford 36 mg (48%) isolated yield as a pale yellow oil. Analytical data agreed with previous reports.

^{1}H NMR (500 MHz, $CDCl_3$) δ 6.85 (d, *J* = 9.8 Hz, 1H), 6.80 (td, *J* = 8.8, 2.7 Hz, 1H), 6.42 (dd, *J* = 8.5, 4.3 Hz, 1H), 3.32 (t, *J* = 8.1 Hz, 2H), 2.95 (t, *J* = 8.1 Hz, 2H), 2.76 (s, 3H).
^{13}C NMR (126 MHz, $CDCl_3$) δ 132.13, 113.12, 112.93, 112.12, 111.92, 107.65, 56.78, 37.14, 28.77.

1-methyl-5-(trifluoromethyl)indoline (18)

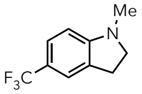

Substrate **18** was synthesized according to General Procedure B using 2-(2-bromo-5-(trifluoromethyl)phenyl)-*N*-methylethan-1-amine for 3 minutes on a 7.4 W 660 nm LED. Following irradiation, the substrate was purified using column chromatography on silica gel where it was eluted using a gradient of 0-5% acetone: hexanes to afford 51.2 mg (51%) isolated yield as a pale pink oil. Analytical data agreed with previous reports.

^{1}H NMR (500 MHz, $CDCl_3$) δ 7.35 (d, *J* = 8.3 Hz, 1H), 7.28 (s, 1H), 6.43 (d, *J* = 8.3 Hz, 1H), 3.44 (t, *J* = 8.4 Hz, 2H), 3.01 (t, *J* = 8.3 Hz, 2H), 2.83 (s, 3H).
^{13}C NMR (126 MHz, $CDCl_3$) δ 155.72, 130.34, 125.45 (q, *J* = 4.2 Hz), 124.13, 121.13 (q, *J* = 3.6 Hz), 119.09, 105.37, 55.57, 35.13, 28.15.
^{19}F NMR (471 MHz, $CDCl_3$) δ -60.61.

1-methyl-1,2,3,4-tetrahydroquinoline (19)

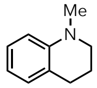

Substrate **19** was synthesized according to General Procedure B using 3-(2-bromophenyl)-*N*-methylpropan-1-amine for 10 minutes. Following irradiation, the substrate was purified using column chromatography on silica gel where it was eluted using a gradient of 0-50% DCM: hexanes to afford 48.6 mg (66%) isolated yield as a colorless oil. Analytical data agreed with previous reports.

^{1}H NMR (500 MHz, $CDCl_3$) δ 7.21 – 7.07 (m, 1H), 6.99 (d, *J* = 8.1 Hz, 1H), 6.64 (t, *J* = 7.7 Hz, 2H), 3.31 – 3.20 (m, 2H), 2.92 (s, 3H), 2.80 (t, *J* = 6.5 Hz, 2H), 2.02 (p, *J* = 6.3 Hz, 2H).
^{13}C NMR (126 MHz, $CDCl_3$) δ 146.78, 128.83, 127.06, 122.90, 116.22, 110.99, 51.31, 39.14, 27.81, 22.49.

Procedure for Figure 4a

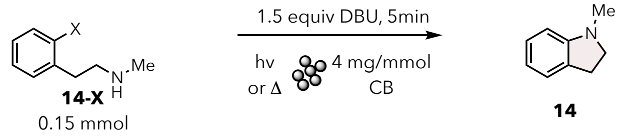

0.15 mmol of **14-X** was added to a ½ dram vial with 1.5 equiv of DBU (33.6 ul,1.5 equiv, 0.225 mmol) and carbon black (0.6 mg, 4 mg/mmol). It was then capped with a septa cap. For thermal trials, the vial was then placed into a sand bath at the denoted temperature (either 235 °C or 285 °C) for 5 minutes. For photothermal trials, the vial was placed directly onto a 13.1 W 660 nm LED and irradiated for 5 minutes. Following bulk heating or irradiation, the reaction is allowed to cool slightly, and then diluted with 1,3,5-trimethoxybenzene solution in $CDCl_3$ for quantitative NMR analysis.

Halogen Exchange Experiments

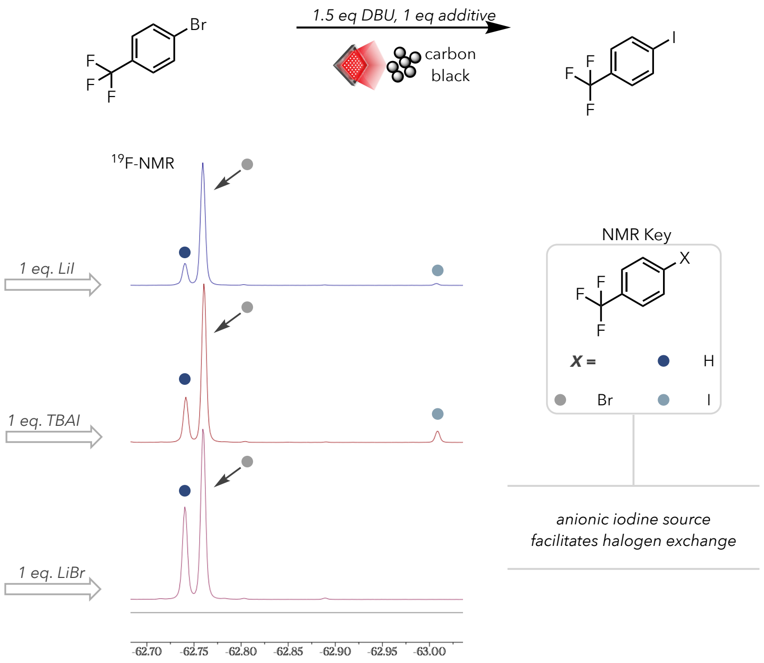

Figure S2. ^{19}F-NMR spectra, demonstrating the presence or absence of the iodinated species.

Procedure for halogen exchange screenings

0.25 mmol of 1-bromo-4-(trifluoromethyl)benzene(1 equiv), carbon black (1 mg, 4 mg/mmol), 1.5 equiv of DBU (56 ul,1.5 equiv, 0.375 mmol), and 1 equiv of additive (LiI, LiBr, TBAI) were added to a ½ dram vial before capping with a septum cap. This mixture was vortexed briefly and placed directly onto a 7.4 W 660 nm LED and irradiated for 15 minutes. Following irradiation, the reaction is removed from the light, allowed to cool slightly, and the sample was diluted with 1,3,5-trimethoxybenzene solution in $CDCl_3$ for quantitative NMR analysis. The formation of each observed species was further confirmed by spiking the NMR with authentic product.

General Procedure C for Photothermal S_NAr of One Pot, Two Step Substrates

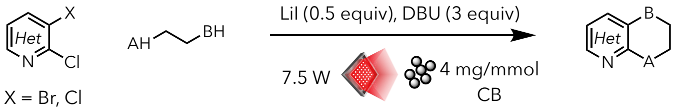

0.5 mmol of dielectrophile (1 equiv), carbon black (2 mg, 4 mg/mmol), dinucleophile (1.5 equiv), LiI (33 mg, 0.5 equiv), and DBU (224 ul, 1.5 mmol, 3 equiv) are added to a 1 dram vial before being capped with a septum cap and vortexed till mixture appears homogenous. The reaction mixture is then placed directly onto a 7.2 W 660 nm LED and irradiated for the specified time. Following irradiation, the reaction is removed from the light, allowed to cool slightly, and the product is isolated via column chromatography or diluted with 1,3,5-trimethoxybenzene solution in $CDCl_3$ for quantitative NMR analysis.

Characterization Data for One Pot, Two Step Products

1,4-dimethyl-1,2,3,4-tetrahydropyrazino[2,3-*b*]pyrazine (21h)

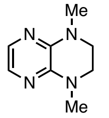

Substrate **21h** was synthesized according to General Procedure C using 2,3-dichloropyrazine with and *N*1,*N*2-dimethylethane-1,2-diamine for 10 minutes. Following irradiation, the substrate was subject to purification using column chromatography where it was eluted on silica gel using 1% TEA and 50-100% ethyl acetate: hexanes to afford 73 mg (89%) yield as a pale brown solid.

^{1}H NMR (500 MHz, $CDCl_3$) δ 7.34 (s, 2H), 3.49 (s, 4H), 3.08 (s, 6H).
^{13}C NMR (126 MHz, $CDCl_3$) δ 144.17, 129.27, 47.10, 36.00.
ESI predicted M+H (165.1134) found 165.1128

1,4-dimethyl-1,2,3,4-tetrahydropyrazino[2,3-b]quinoxaline (22h)

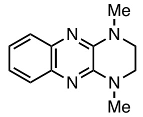

Substrate **22h** was synthesized according to General Procedure C using 2,3-dichloroquinoxaline *N*1, *N*2-dimethylethane-1,2-diamine for 20 minutes. Following irradiation, the substrate was subject to purification using column chromatography where it was eluted on silica gel using 1% TEA and 0-60% ethyl acetate: hexanes to afford 66.7 mg (62%) yield as a yellow, crystalline solid.

^{1}H NMR (500 MHz, $CDCl_3$) δ 7.57 (dd, *J* = 6.1, 3.5 Hz, 2H), 7.26 (dd, *J* = 6.1, 3.4 Hz, 2H), 3.57 (s, 4H), 3.25 (s, 6H).
^{13}C NMR (126 MHz, $CDCl_3$) δ 144.05, 137.32, 125.12, 124.21, 47.01, 36.34.
ESI predicted M+H (215.1291) found 215.1296

7-chloro-1,4-dimethyl-1,2,3,4-tetrahydropyrido[2,3-*b*]pyrazine (23h)

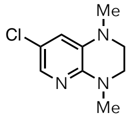

Substrate **23h** was synthesized according to General Procedure C using 3-bromo-5-chloro-2-fluoropyridine and *N*1,*N*2-dimethylethane-1,2-diamine for 60 minutes. Following irradiation, the substrate was purified using column chromatography on silica gel where it was eluted using a gradient of 0-2% acetone: hexanes to afford 61.1 mg (62%) yield as a brown oil.

^{1}H NMR (500 MHz, $CDCl_3$) δ 7.50 (d, *J* = 3.4 Hz, 1H), 6.50 (d, *J* = 2.1 Hz, 1H), 3.52 – 3.45 (m, 2H), 3.29 (d, *J* = 4.9 Hz, 2H), 3.09 (s, 3H), 2.84 (s, 3H).
^{13}C NMR (126 MHz, $CDCl_3$) δ 146.31, 133.17, 132.87, 119.93, 114.12, 48.23, 48.07, 38.44, 36.66.
ESI predicted M+ (197.0719) found 197.0715

5,8-dimethyl-5,6,7,8-tetrahydropteridine (24h)

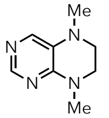

Substrate **24h** was synthesized according to General Procedure C using 5-bromo-4-chloropyrimidine and *N*1,*N*2-dimethylethane-1,2-diamine for 5 minutes. Following irradiation, the substrate was purified using column chromatography on silica gel where it was eluted on silica gel using a 1% TEA and a gradient of 10-30% ethyl acetate: hexanes to afford 62 mg (75%) yield as a pale brown oil.

^{1}H NMR (500 MHz, $CDCl_3$) δ 8.15 (s, 1H), 7.45 (s, 1H), 3.64 – 3.56 (m, 2H), 3.23 – 3.18 (m, 2H), 3.17 (s, 3H), 2.84 (s, 3H).
^{13}C NMR (126 MHz, $CDCl_3$) δ 151.75, 149.09, 132.03, 129.39, 48.23, 47.42, 38.04, 35.55.
ESI predicted (M+H) 165.1135 found (M+H) 165.1105

5,8-dimethyl-5,6,7,8-tetrahydropteridin-2-amine (25h)

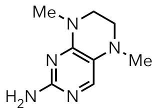

Substrate **25h** was synthesized according to General Procedure C using 4,5-dichloropyrimidin-2-amine and *N*1,*N*2-dimethylethane-1,2-diamine for 120 minutes. Following irradiation, the substrate was purified using column chromatography on silica gel where it was eluted on silica gel using a gradient of 0-10% methanol: DCM to afford 25.5 mg (28%) yield as a pale brown solid.

^{1}H NMR (500 MHz, $CDCl_3$) δ 6.67 (d, J = 5.6 Hz, 1H), 3.67 (t, J = 5.2 Hz, 2H), 3.24 (s, 3H), 3.21 – 3.12 (m, 2H), 2.74 (s, 3H).
^{13}C NMR (126 MHz, $CDCl_3$) δ 154.48, 151.85, 122.97, 116.80, 48.80, 46.93, 38.57, 36.54.
ESI predicted (M+H) 180.1244 found 180.1255

1,4-dimethyl-1,2,3,4-tetrahydropyrido[3,4-*b*]pyrazin-7-amine (26h)

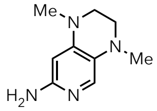

Substrate **26h** was synthesized according to General Procedure C using 4,5-dichloropyridin-2-amine and *N*1,*N*2-dimethylethane-1,2-diamine for 11 hours. Following irradiation, the substrate was purified using column chromatography on silica gel where it was eluted on silica gel using a gradient of 0-10% methanol: DCM to afford 15.8 mg (18%) yield as a brown oil.

^{1}H NMR (500 MHz, $CDCl_3$) δ 6.95 (s, 1H), 5.61 (s, 1H), 4.89 (s, 2H), 3.62 – 3.47 (m, 2H), 3.15 – 3.05 (m, 2H), 2.97 (s, 3H), 2.77 (s, 3H).
^{13}C NMR (126 MHz, $CDCl_3$) δ 151.53, 146.57, 126.91, 87.76, 49.94, 48.53, 39.47, 38.20, 29.72.
ESI predicted (M+H) 179.1291found 179.1273

1,4-dimethyl-1,2,3,4-tetrahydropyrido[3,4-*b*]pyrazine (27h)

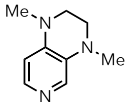

Substrate **27h** was synthesized according to General Procedure C using 3-bromo-4-chloropyridine and *N*1, *N*2-dimethylethane-1,2-diamine for 30 minutes. Following irradiation, the substrate was purified using column chromatography on silica gel where it was eluted on silica gel using a gradient of 0-10% methanol: DCM to afford 34.6 mg (42%) product. Analytical data agreed with previous reports.

^{1}H NMR (500 MHz, $CDCl_3$) δ 7.81 (d, J = 6.7 Hz, 1H), 7.43 (s, 1H), 6.52 (d, J = 6.7 Hz, 1H), 3.72 (t, J = 4.9 Hz, 2H), 3.38 – 3.30 (m, 2H), 3.19 (s, 3H), 2.93 (s, 3H).
^{13}C NMR (126 MHz, $CDCl_3$) δ 146.74, 132.51, 131.59, 117.08, 102.22, 49.69, 46.35, 38.98, 38.92.

1,4-dimethyl-1,2,3,4-tetrahydropyrido[2,3-b]pyrazine (28h)

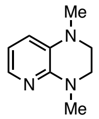

Substrate **28h** was synthesized according to General Procedure C using 3-bromo-2-chloropyridine and *N*1, *N*2-dimethylethane-1,2-diamine for 60 minutes. Following irradiation, the substrate was subject to purification using column chromatography where it was eluted on silica gel using 1% TEA and 0-8% ethyl acetate: hexanes to afford 56.2 mg (69%) yield as a brown solid. Analytical data agreed with previous reports

^{1}H NMR (500 MHz, $CDCl_3$) δ 7.58 (d, J = 5.1 Hz, 1H), 6.56 (d, J = 7.6 Hz, 1H), 6.50 (dd, J = 7.6, 4.9 Hz, 1H), 3.55 – 3.45 (m, 2H), 3.29 – 3.20 (m, 2H), 3.11 (s, 3H), 2.82 (s, 3H).
^{13}C NMR (126 MHz, CDCl3) δ 148.08, 136.07, 132.24, 114.52, 112.77, 53.43, 48.53, 38.56, 36.58.

1,2,3,4-tetrahydropyrazino[2,3-*b*]pyrazine (21i)

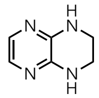

Substrate **21i** was synthesized according to General Procedure C using 2,3-dichloropyrazine and ethylene diamine for 15 minutes. Following irradiation, the substrate was purified using column chromatography on silica gel where it was eluted using a gradient of 50-100% ethyl acetate: hexanes to afford 28.7 mg (42%) pure product.

^{1}H NMR (500 MHz, $CDCl_3$) δ 7.23 (s, 2H), 4.81 (s, 2H), 3.47 (s, 4H).
^{13}C NMR (126 MHz, $CDCl_3$) δ 143.11, 130.70, 77.23, 39.86.
ESI predicted (M+H) 137.0821 found (M+H) 137.0819

1-methyl-1,2,3,4-tetrahydropyrazino[2,3-*b*]pyrazine (21j)

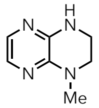

Substrate **21j** was synthesized according to General Procedure C using 2,3-dichloropyrazine and N1-methylethane-1,2-diamine for 20 minutes. Following irradiation, the substrate was purified using column chromatography on silica gel where it was eluted using a gradient of 50-100% ethyl acetate: hexanes to afford 51.8 mg (69%) yield as a pale brown solid.

^{1}H NMR (500 MHz, $CDCl_3$) δ 7.41 (d, *J* = 3.2 Hz, 1H), 7.23 (d, *J* = 3.2 Hz, 1H), 4.93 (s, 1H), 3.57 (t, *J* = 4.2 Hz, 2H), 3.50 – 3.44 (m, 2H), 3.10 (s, 3H).
^{13}C NMR (126 MHz, $CDCl_3$) δ 143.93, 143.39, 130.92, 129.11, 47.72, 39.31, 35.76.
ESI predicted (M+H) 151.0978 found (M+H) 151.0980

5,9-dimethyl-6,7,8,9-tetrahydro-5*H*-pyrazino[2,3-*b*]azepine (21k)

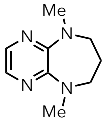

Substrate **21k** was synthesized according to General Procedure C using 2,3-dichloropyrazine and *N^1,N^3*-dimethylpropane-1,3-diamine for 30 minutes. Following irradiation, the substrate was purified using column chromatography on silica gel where it was eluted using a gradient of 0-50% ethyl acetate: hexanes to afford 76 mg (85%) yield as a pale brown solid.

^{1}H NMR (500 MHz, $CDCl_3$) δ 7.48 (s, 2H), 3.55 – 3.46 (m, 4H), 3.00 (s, 6H), 2.00 – 1.89 (m, 2H).
^{13}C NMR (126 MHz, $CDCl_3$) δ 149.13, 130.61, 50.72, 39.05, 25.31.
ESI predicted (M+H) 179.1291 found (M+H) 179.1214

1,4-diethyl-1,2,3,4-tetrahydropyrazino[2,3-*b*]pyrazine (21l)

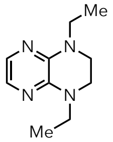

Substrate **21l** was synthesized according to General Procedure C using 2,3-dichloropyrazine and *N*1, *N*2-diethylethane-1,2-diamine for 15 minutes. Following irradiation, the substrate was purified using column chromatography on silica gel where it was eluted using a gradient of 50-100% ethyl acetate: hexanes to afford 51.7mg (54%) yield as a pale brown solid.

^{1}H NMR (500 MHz, $CDCl_3$) δ 7.31 (s, 2H), 3.61 (q, *J* = 7.2 Hz, 4H), 3.48 (s, 4H), 1.19 (t, *J* = 7.2 Hz, 6H).
^{13}C NMR (126 MHz, $CDCl_3$) δ 143.14, 128.74, 44.16, 42.53, 11.31.
ESI predicted (M+H) 151.0978 found (M+H) 151.0980

1,4-dibenzyl-1,2,3,4-tetrahydropyrazino[2,3-*b*]pyrazine (21m)

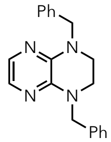

Substrate **21m** according to General Procedure C using 2,3-dichloropyrazine and N^1,N^2-dibenzylethane-1,2-diamine for 20 minutes. Following irradiation, the substrate was purified using column chromatography on silica gel where it was eluted using a gradient of 50-100% ethyl acetate: hexanes to afford 59.1 mg (37%) yield as a yellow oil.

^{1}H NMR (500 MHz, $CDCl_3$) δ 7.41 (s, 2H), 7.37 – 7.24 (m, 10H), 4.84 (s, 4H), 3.35 (s, 4H).
^{13}C NMR (126 MHz, $CDCl_3$) δ 143.20, 137.65, 129.37, 128.55, 128.06, 127.22, 50.70, 44.00.
ESI predicted (M+H) 317.1761 found 317.1789

3,4-dihydro-2*H*-pyrazino[2,3-*b*][1,4]thiazine (21n)

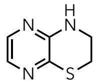

Substrate **21n** was synthesized by irradiation of 0.5 mmol of 2,3-dichloropyrazine with 1.5 equivalents of 2-Mercaptoethylamine•HCl, 4.5 equivalents of DBU, and 2 mg of CB for 6 minutes. Following irradiation, the substrate was purified using column chromatography on silica gel where it was eluted using a gradient of 50-100% ethyl acetate: hexanes to afford 26.3 mg (34%) yield as a pale yellow solid.

^{1}H NMR (500 MHz, $CDCl_3$) δ 7.74 (d, J = 2.9 Hz, 1H), 7.69 (d, J = 2.7 Hz, 1H), 5.12 (s, 1H), 3.85 – 3.77 (m, 2H), 3.24 – 3.17 (m, 2H).
^{13}C NMR (126 MHz, $CDCl_3$) δ 149.64, 139.16, 137.91, 133.61, 42.09, 26.22.
ESI predicted (M+H) 154.0433 found (M+H) 154.0304

(5a*S*,9a*S*)-5,10-dimethyl-5,5a,6,7,8,9,9a,10-octahydropyrazino[2,3-*b*]quinoxaline (21o)

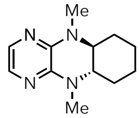

Substrate **21o** was synthesized according to General Procedure C using 0.5 mmol of 2,3-dichloropyrazine with (1*S*,2*S*)-*N*1,*N*2-dimethylcyclohexane-1,2-diamine for 10 minutes. Following irradiation, the substrate was purified using column chromatography on silica gel where it was eluted using a gradient of 0-30% ethyl acetate: hexanes to afford 49.2 mg (60%) yield as a pale brown solid.

^{1}H NMR (500 MHz, $CDCl_3$) δ 7.37 (s, 2H), 3.07 (s, 6H), 3.06 – 2.99 (m, 2H), 2.40 (dd, J = 10.5, 3.0 Hz, 2H), 1.97 – 1.82 (m, 2H), 1.45 – 1.21 (m, 4H).
^{13}C NMR (126 MHz, $CDCl_3$) δ 145.30, 129.19, 59.57, 30.26, 29.69, 24.20.
ESI predicted (M+H) 219.1604 found (M+H) 219.1608

5,10-dimethyl-5,5a,6,7,8,9,9a,10-octahydropyrido[2,3-*b*]quinoxaline (28o)

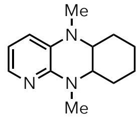

Substrate **28o** was synthesized according to General Procedure C using 3-bromo-2-chloropyridine and N^1,N^2-dimethylcyclohexane-1,2-diamine for 60 minutes. Following irradiation, the substrate was purified using column chromatography on silica gel where it was eluted using a gradient of 0-10% ethyl acetate: hexanes to afford 55.9 mg (52%) yield as a brown oil. Analytical data agreed with previous reports.

^{1}H NMR (500 MHz, $CDCl_3$) δ 7.63 (d, *J* = 5.0 Hz, 1H), 6.69 (d, *J* = 7.6 Hz, 1H), 6.54 (t, *J* = 7.3 Hz, 1H), 3.13 (s, 3H), 3.11 – 3.03 (m, 1H), 2.77 (s, 3H), 2.73 (d, *J* = 11.6 Hz, 1H), 2.35 (dd, *J* = 33.8, 13.5 Hz, 2H), 1.93 – 1.80 (m, 2H), 1.32 (dt, *J* = 52.0, 11.9 Hz, 4H).
^{13}C NMR (126 MHz, $CDCl_3$) δ 136.30, 133.84, 115.78, 112.91, 61.46, 60.33, 33.08, 31.55, 30.30, 29.88, 24.51, 24.20.

Computational data

All computations were performed in Gaussian 16 at the M11/def2-TZVP level of theory, with TZVPD basis for the halogen atoms Cl, Br, and I (including the corresponding pseudopotential for I). All calculations utilized the self-consistent reaction field in the conductor-like polarizable continuum model (CPCM) with 2-methylpyridine as the solvent, except for the 7th entry, which was benchmarked experimentally (see below). Temperature was specified as noted for all computations for comparison with thermal experiments.

Table S1

Substrate	Substrate complex	Transition State	Imaginary Frequency	ΔG‡ (kcal/mol)	ΔH‡ (kcal/mol)	ΔS‡ (cal/mol K)
14-F	-316637.50	-316602.30	-296.99	35.2 (570K)	29.6	9.9
14-Cl	-542752.74	-542715.20	-460.09	37.5 (570K)	32.8	8.2
14-Br	-1869094.31	-1869059.61	-444.88	34.7 (570K)	29.9	8.4
14-I	-440712.12	-440677.93	-444.67	34.2 (570K)	29.4	8.4
13a	-1928481.17	-1928430.07	-374.75	51.1 (570K)	27.7	41.8
1a (DMAc)	-2129937.91	-2129899.63	-317.39	38.3 (438K)	24.1	24.8
1a	-2129964.34	-2129920.95	-318.18	43.4 (570K)	25.0	32.9
31a	-2177176.28	-2177126.25	-362.31	50.0 (570K)	27.9	40.6
33a	-2146752.88	-2146702.17	-368.32	50.7 (570K)	27.0	41.1

Experimental Kinetics Benchmarking Procedure:

In 1-dram vials, 0.5 mmol of 4-trifluoromethyl bromobenzene **1** was combined with 1 mmol piperidine **a** and 0.75 mmol DBU and diluted with dimethylacetamide to a total volume of 1 mL. The reactions were heated to 165 °C and sampled every hour for 5 hours. At each time point, the concentrations of **1** and **a** were monitored for 3 trials, and the rate constant was thus determined to be 1.62×10^{-5}. From the Eyring equation, we calculated that this would be expected from a reaction with activation energy of approximately 35.6 kcal/mol, a 2.7 kcal/mol difference from our calculated energy that thus validated the acceptability of our chosen methodology.

Transition State 1 (intra-F)

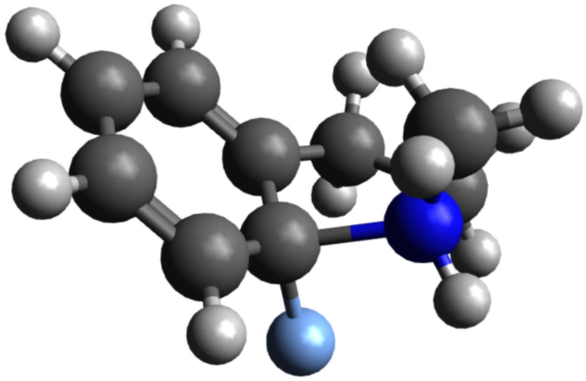

Transition State 2 (intra-Cl)

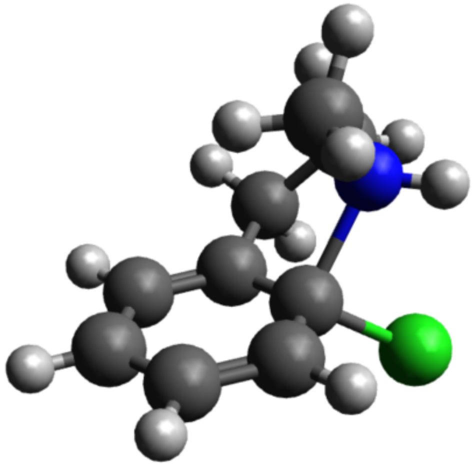

Transition State 3 (intra-Br)

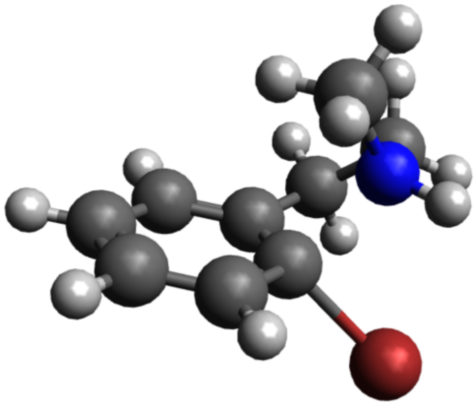

Transition State 4 (intra-I)

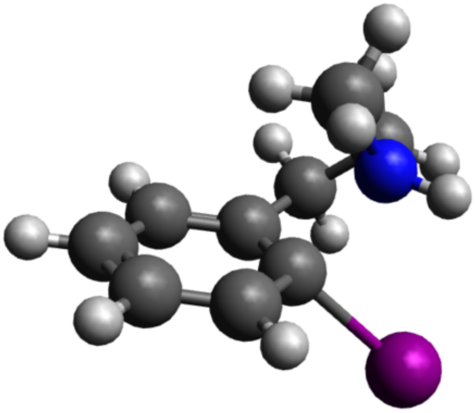

Transition State 5 (Piperidine attack on 3-bromopyridine)

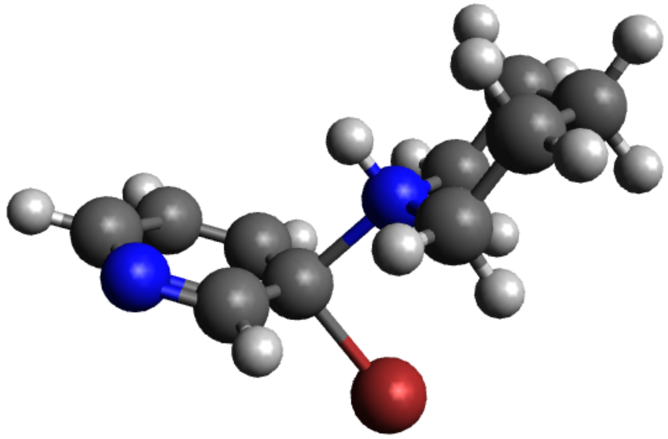

Transition State 6 (Piperidine attack on 4-trifluoromethylbromobenzene)

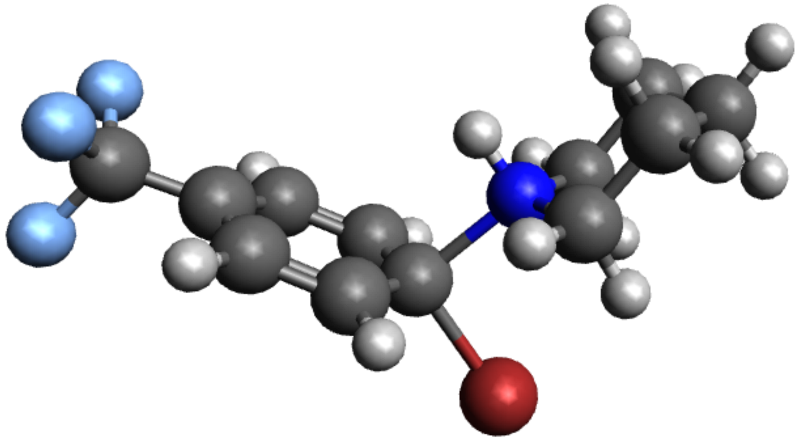

Transition State 7 (Piperidine attack on 4-Trifluoromethoxybromobenzene)

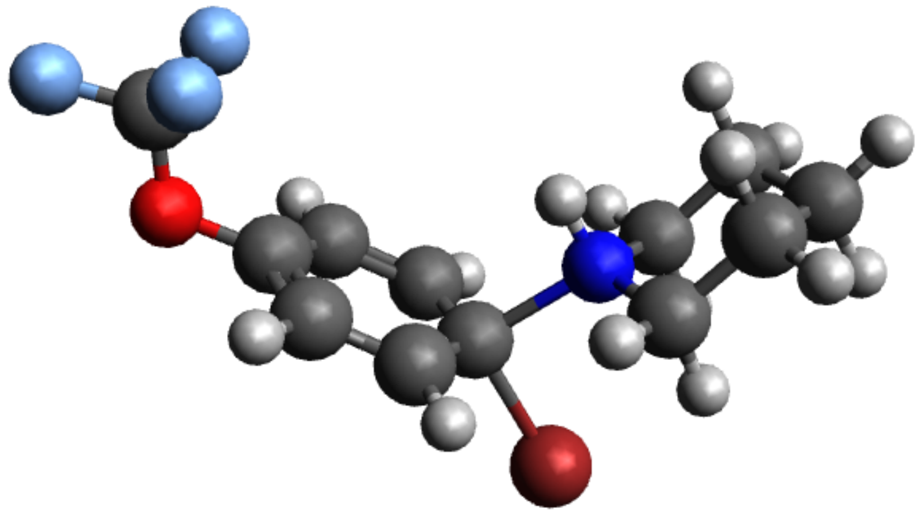

Transition State 8 (Piperidine attack on (3-bromophenyl)(piperidin-1-yl)methanone)

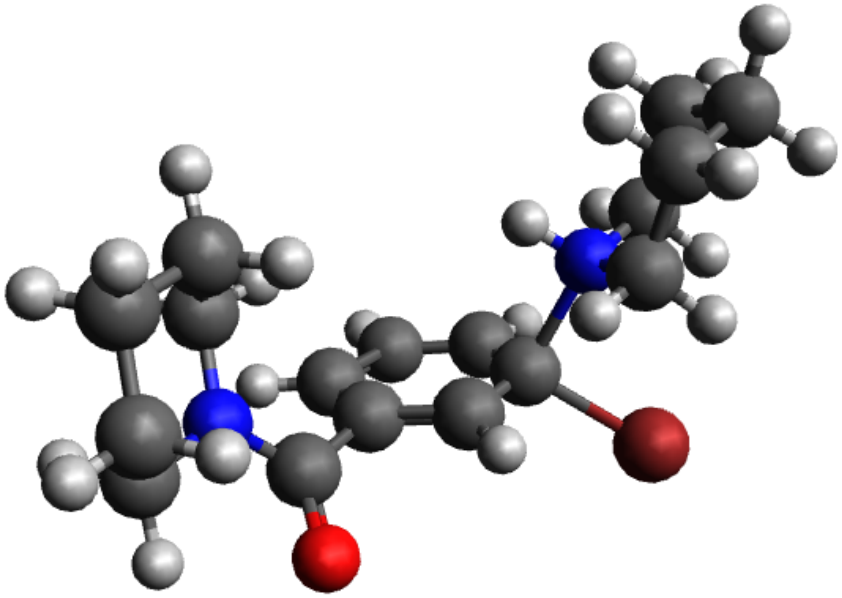

ICP-MS Characterization of Carbon Black, DBU, and LiI

Inductively Coupled Plasma-Mass spectrometry (ICP-MS) was performed by MSE Analytical Services using an Agilent 7800 ICP-MS, where experiments were run in triplicates and an average value for the ppm of Fe, Cu, and Pd was determined for the carbon black (CB), lithium iodide (LiI), and 1,8-Diazabicyclo(5.4.0)undec—7-ene (DBU). Analytical methods are detailed below.

RF Power：1.55KW
RF Matching：1.80V
Auxiliary flow：1.50L/min
Carrier Gas：1.0L/min
Makeup Gas:0.48L/min
Uptake Time:45sec
Stabilization Time:30sec
Integ Time per mass:0.90se

$$Cx(ug/kg) = \frac{C_0(ug/L) * f * V_0(mL) * 10^{-3}}{m(g) * 10^{-3}} = \frac{C_1(ug/L) * V_0(mL) * 10^{-3}}{m(g) * 10^{-3}}$$

$$W(\%) = \frac{Cx(ug/kg)}{10^9} * 100\%$$

$$C_1(mg/L) = C_0(mg/L) * f$$

m0: The mass of the sample taken when analyzing the sample, in grams (g), as recorded by the analytical balance, corresponding to the data in column B in the table below.

V0: The volume of the sample after digestion, in milliliters (mL), corresponding to the data in column C in the table below.

f: Dilution factor, corresponding to the data in column F in the table below.

Co: The concentration of the elements in the test solution in milligrams per liter (ug/L), this data is obtained from the instrument test, corresponding to the data in column E of the following table.

C1: Elemental concentration of the sample digest stock solution, in milligrams per liter (ug/L), this data is calculated from equation (3), if the sample is a liquid that can be directly tested, C1 is the final test result, corresponding to the data in column G of the table below.

W (%): The final test result of the measured element, expressed as a percentage, calculated from equation (2) above, corresponding to the data in column I in the table below

Note: The final results of powdered solid samples or other samples that need to be treated before digestion are calculated by equation (1) or (2), and the final results of liquid samples that can be directly tested are calculated by equation (3).

Sample ID	Sample quality (g)	Act Vol (mL)	Element	Soln Conc(ug/L)	Dilution factor	Corr Con (ug/L)	Sample con (ug/kg)	Sample con(%)
Lithium iodide	0.1191	25	Fe	51.414	1	51.414	10792.233	0.00%
Lithium iodide	0.1191	25	Fe	52.121	1	52.121	10940.653	0.00%
Lithium iodide	0.1191	25	Fe	51.969	1	51.969	10908.644	0.00%
carbon black	0.0364	25	Fe	222.073	1	222.073	152522.424	0.02%
carbon black	0.0364	25	Fe	222.342	1	222.342	152707.672	0.02%
carbon black	0.0364	25	Fe	222.298	1	222.298	152677.500	0.02%
Lithium iodide	0.1191	25	Cu	12.123	1	12.123	2544.794	0.00%
Lithium iodide	0.1191	25	Cu	13.051	1	13.051	2739.484	0.00%
Lithium iodide	0.1191	25	Cu	13.145	1	13.145	2759.215	0.00%
carbon black	0.0364	25	Cu	13.580	1	13.580	9326.854	0.00%
carbon black	0.0364	25	Cu	11.551	1	11.551	7933.310	0.00%
carbon black	0.0364	25	Cu	11.498	1	11.498	7897.184	0.00%
Lithium iodide	0.1191	25	Pd	0.785	1	0.785	164.840	0.00%
Lithium iodide	0.1191	25	Pd	1.114	1	1.114	233.900	0.00%
Lithium iodide	0.1191	25	Pd	0.635	1	0.635	133.354	0.00%
carbon black	0.0364	25	Pd	1.126	1	1.126	773.146	0.00%
carbon black	0.0364	25	Pd	0.593	1	0.593	406.937	0.00%
carbon black	0.0364	25	Pd	0.569	1	0.569	390.453	0.00%

Sample ID	Sample Vol（mL）	Act Vol（mL)	Elment	Soln Conc(ug/L)	Dilution factor	Corr Con（ug/L)	Sample con（ug/L)
DBU	1	25	Fe	54.733	1	54.733	1368.333
DBU	1	25	Fe	54.609	1	54.609	1365.229
DBU	1	25	Fe	53.896	1	53.896	1347.409
DBU	1	25	Cu	8.148	1	8.148	203.688
DBU	1	25	Cu	7.648	1	7.648	191.210
DBU	1	25	Cu	7.459	1	7.459	186.478
DBU	1	25	Pd	0.552	1	0.552	13.805
DBU	1	25	Pd	0.276	1	0.276	6.903
DBU	1	25	Pd	0.386	1	0.386	9.655

XPS Characterization of Carbon Black Pre and Post Reaction

Procedure for XPS Studies

0.5 mmol of 1-bromo-4-(trifluoromethyl)benzene was weighed into a 1-dram vial, along with 1.5 equiv DBU, and 2 equiv piperidine. 4 mg of carbon black was then added to achieve a concentration of 4 mg/mmol. The reaction was then capped with a septa cap and placed atop a 660 nm LED and irradiated for 180 minutes. 1 M HCl (5 mL) was then added to the reaction mixture, which was then sonicated to ensure all solids were fully suspended before this was transferred to a centrifuge tube. The reaction was then centrifuged at 4400 rpm for 30 minutes, and then the solvent was decanted off. This washing cycle was performed 3x before an identical washing cycle using 1 M NaOH (5 mL) was performed an additional 3x. Following this, an identical washing cycle using water (5 mL) was performed an additional 3x. Finally, an identical washing cycle using acetone (5 mL) was performed an additional 3x. Following this, the carbon black was transferred to a vacuum oven to dry, and then XPS was performed.

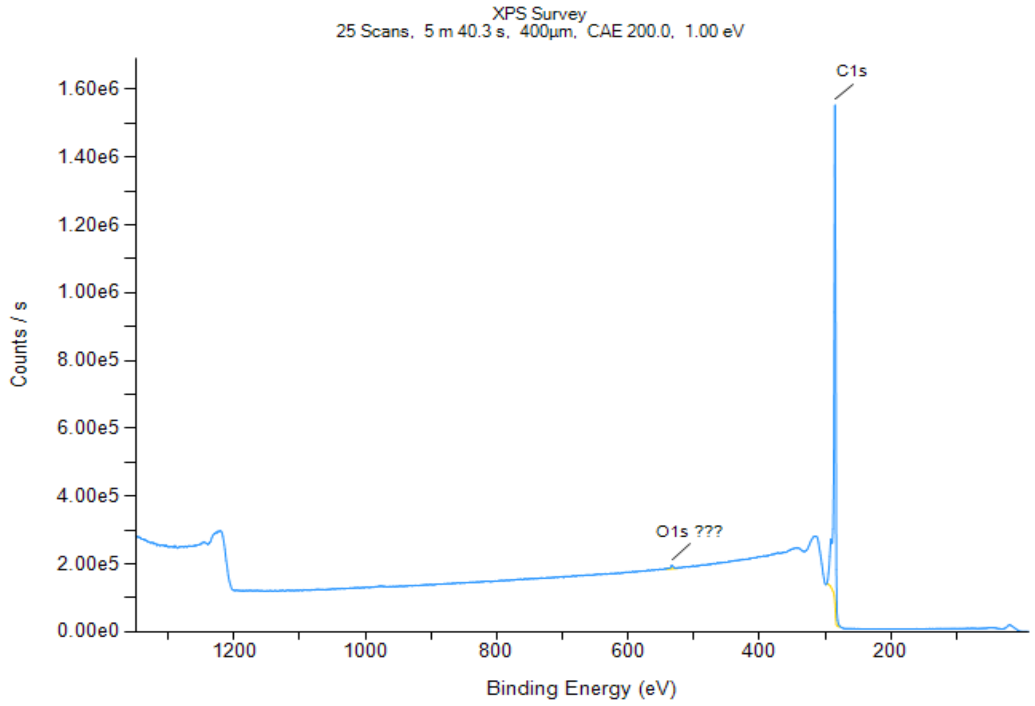

Figure S3. XPS spectral scan of virgin carbon black

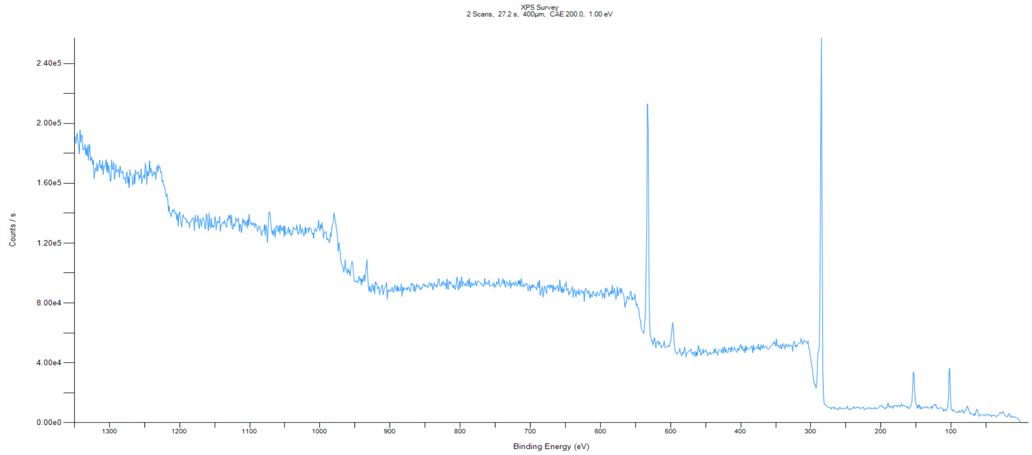

Figure S4. XPS spectral scan of carbon black following reaction

Isotherm Characterization of Carbon Black

Procedure for Isotherm Characterization of Carbon Black

To prepare carbon black for isotherm characterization, 0.5 g of carbon black was dried under vacuum using Schlenk technique at elevated temperatures (110 °C) for 6h. It was then allowed to cool under Nitrogen before being transferred to the instrument, where it was subject to vacuum at elevated temperatures (120 °C) overnight in order to activate the sample for analysis . The sample was then run on an ASAP 2020 Plus Version 2.00, performing Nitrogen physisorption at 77K to determine the specific surface area and porosity of the carbon black photothermal agent. The specific surface area was calculated to be 74.9313 m^2/g and a plot of pore size distribution is included below.

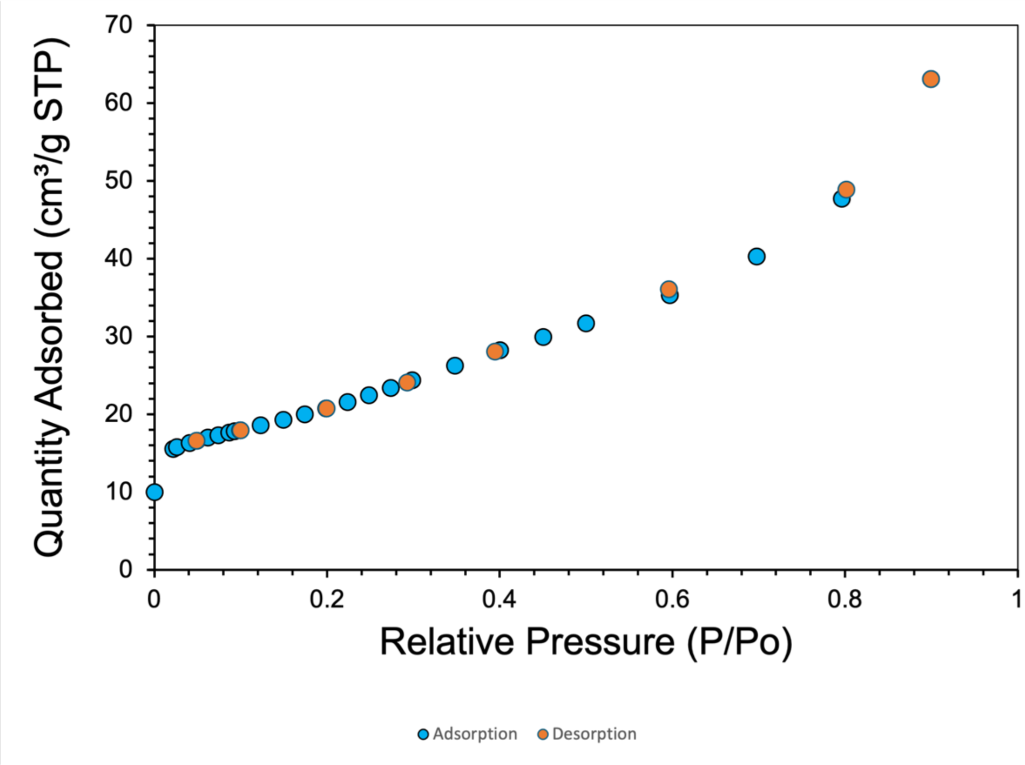

Figure S5. Isotherm of Carbon Black under N_2 at 77K

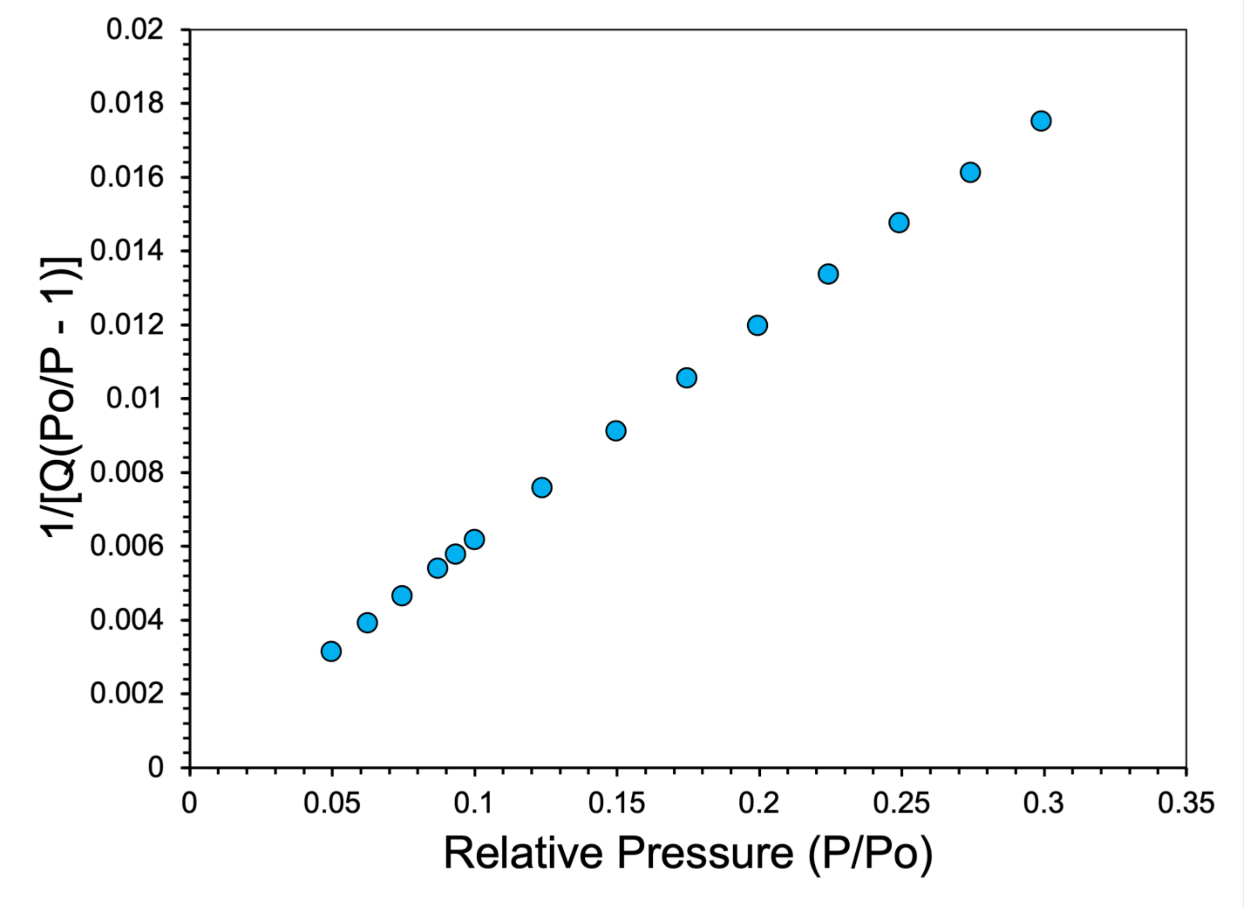

Figure S6. BET Plot

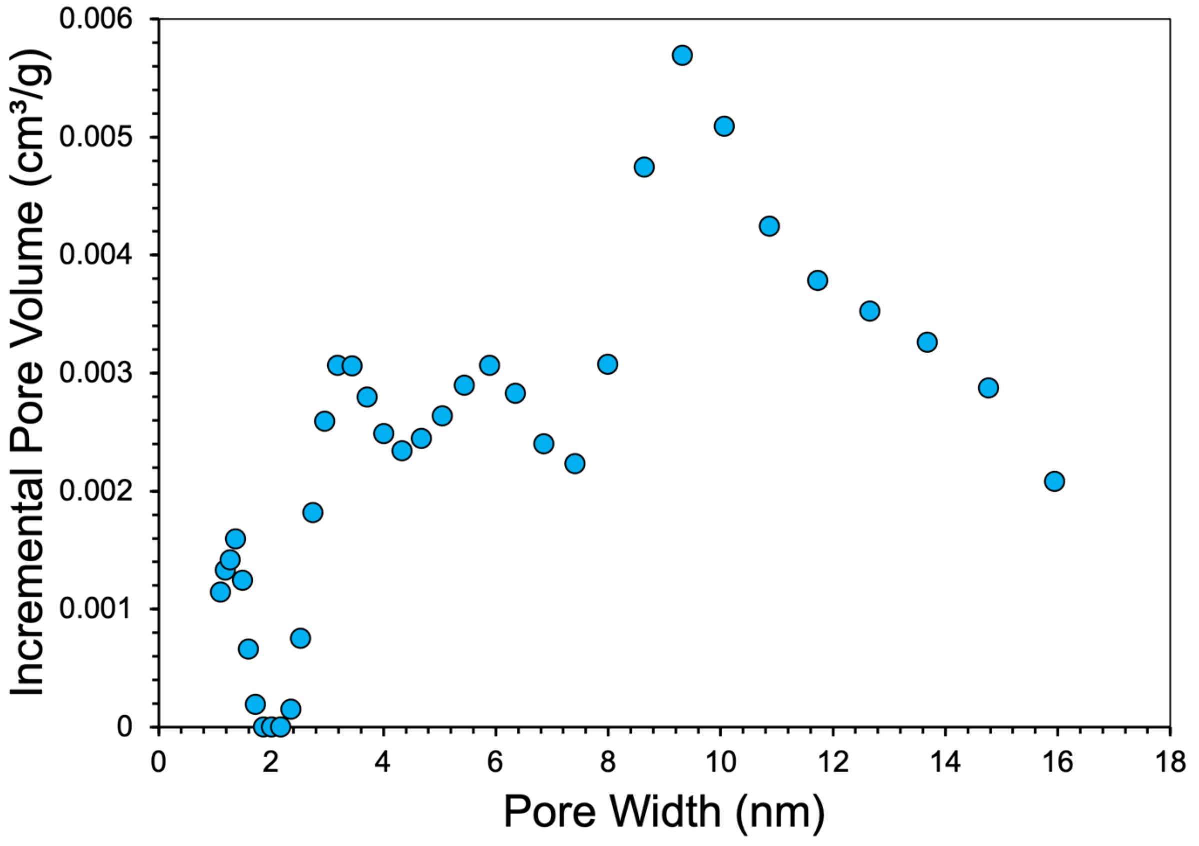

Figure S7. Pore Width distribution for Carbon Black Photothermal Agent

Insight into Light Intensity's Effect on Photothermal Conversion

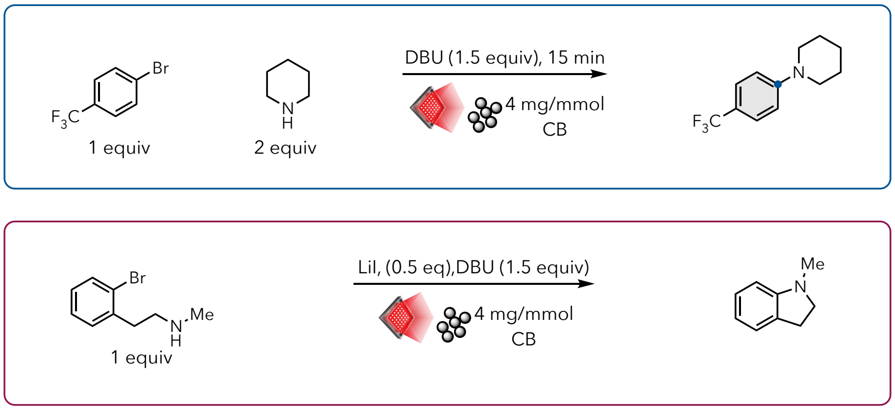

Figure S8. Inter- and intramolecular reactions screening with light intensity

Procedure for Intermolecular intensity screenings:
0.25 mmol of 1-bromo-4-(trifluoromethyl)benzene (1 equiv), carbon black (2 mg,4 mg/mmol), DBU (56 ul, 0.375mmol, 1.5 equiv) and piperidine (49.9 ul,0.5 mmol, 2 equiv) were added to a ½ dram vial before capping with a septum cap. This mixture was vortexed briefly and placed directly onto a 660 nm LED whose intensity was adjusted throughout the trials from 7-12W and irradiated for 15 minutes. Following irradiation, the reaction is removed from the light, allowed to cool slightly, and the sample was diluted with 1,3,5-trimethoxybenzene solution in $CDCl_3$ for quantitative NMR analysis.

Procedure for Intermolecular intensity screenings:
0.25 mmol of 2-(2-bromophenyl)-*N*-methylethan-1-amine (1 equiv), 2 mg of carbon black (2 mg,4 mg/mmol), DBU (56 ul,0.375mmol,1.5 equiv), and LiI (33 mg, 0.5 equiv) were added to a ½ dram vial before capping with a septum cap. This mixture was vortexed briefly and placed directly onto a 660 nm LED whose intensity was adjusted throughout the trials from 7-12W and irradiated for 15 minutes. Following irradiation, the reaction is removed from the light, allowed to cool slightly, and the sample was diluted with 1,3,5-trimethoxybenzene solution in $CDCl_3$ for quantitative NMR analysis.

Note: For photothermally promoted systems, the number of incident photons absorbed by the system correlates to the number of excitation-relaxation events which produce heat.[3–6] This unique facet of photothermal conversion can be observed in the near linear trend between conversion/yield and photon equivalence for the photothermal coupling of 4-trifluoromethyl bromobenzene and piperidine The intramolecular S_NAr of 2-(2-bromophenyl)-N-methylethan-1-amine produces a similar trend, however, at greater photon equivalency, quantitative conversion is achieved whilst yield drops due to competitive side reactivity.

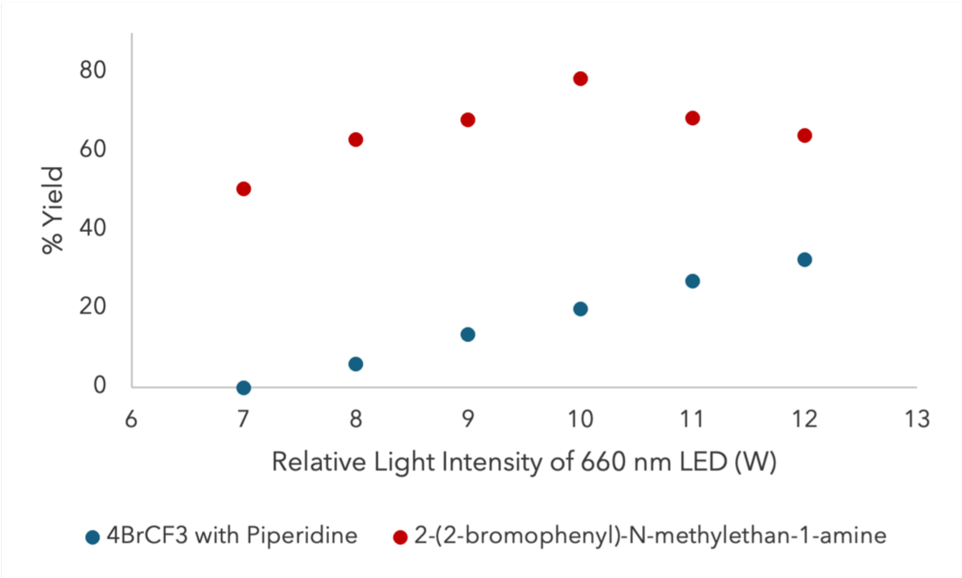

Figure S9: Reaction performance across various light intensity

Carbon black loading studies

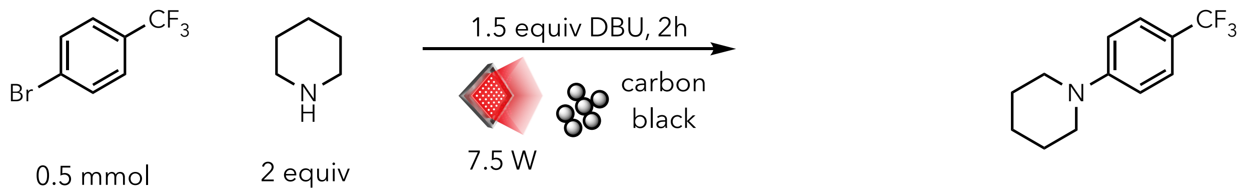

Entry	CB loading	Conversion %	Yield %
1	4 mg/mmol	30%	28%
2	8 mg/mmol	35%	34%
3	16 mg/mmol	46%	46%
4	32 mg/mmol	60%	54%
5	64 mg/mmol	37%	33%
6	128 mg/mmol	11%	9%

Figure S10. Reaction performance with different CB loadings

Spectra

2-bromo-*N*-phenylbenzamide (7)

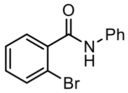

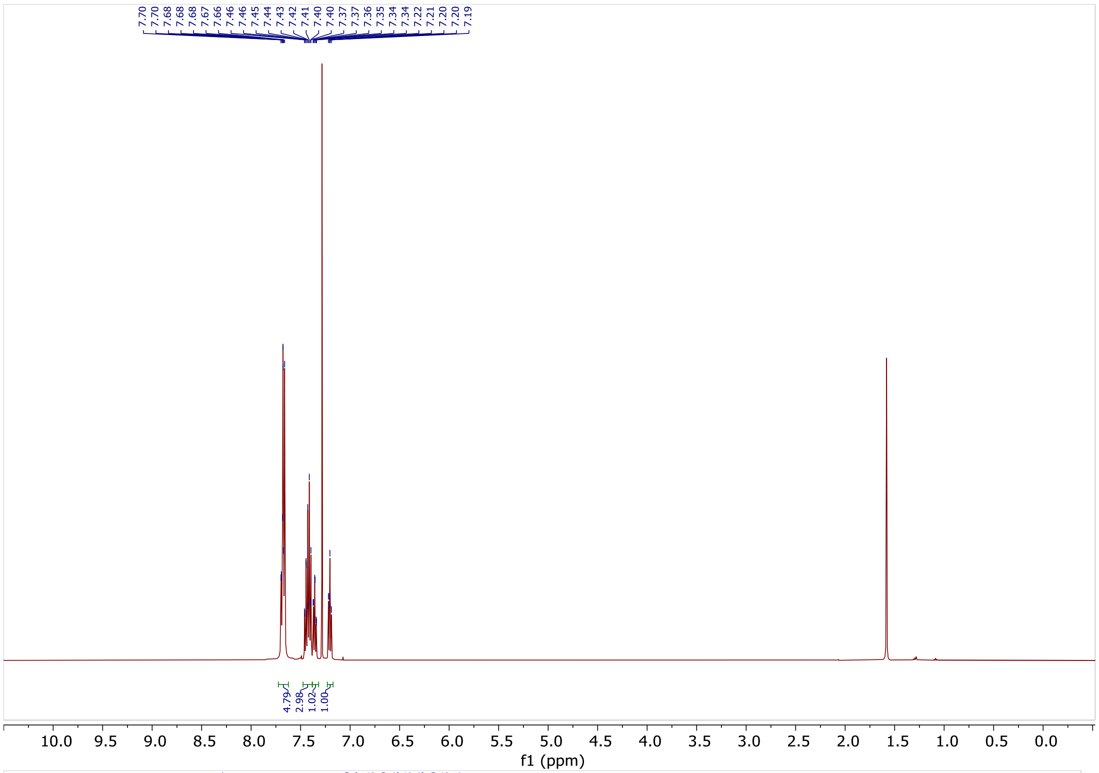

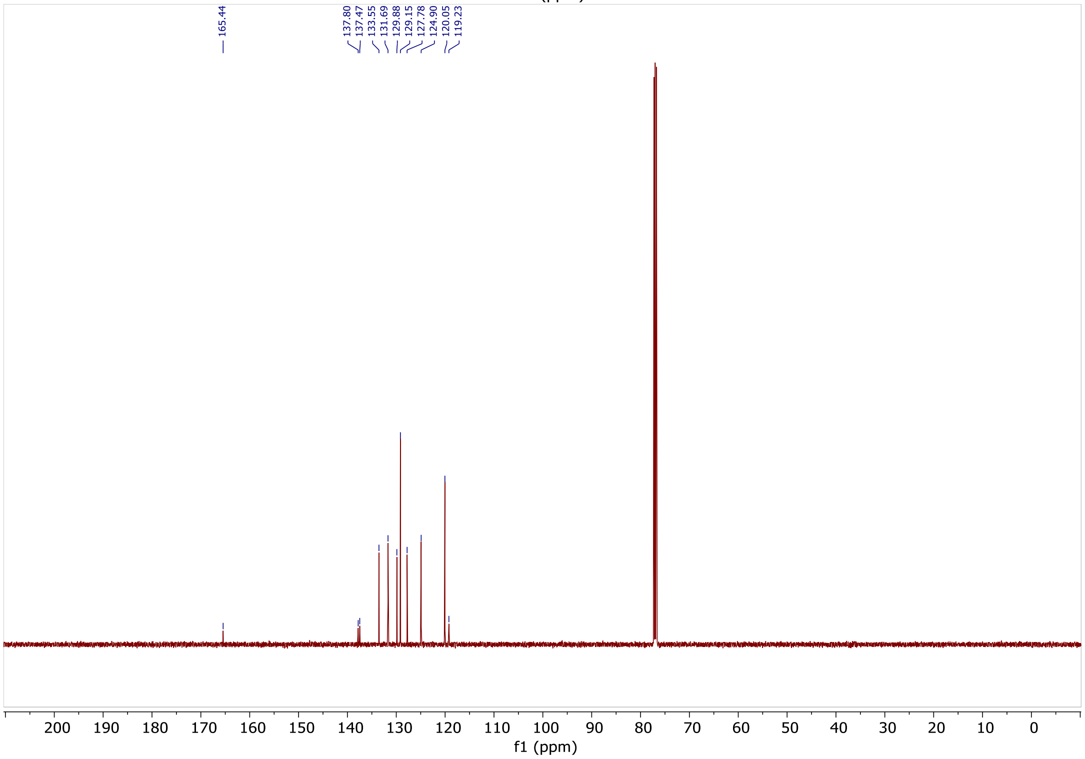

1-(4-trifluromethyl)phenylpiperidine (1a)

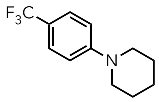

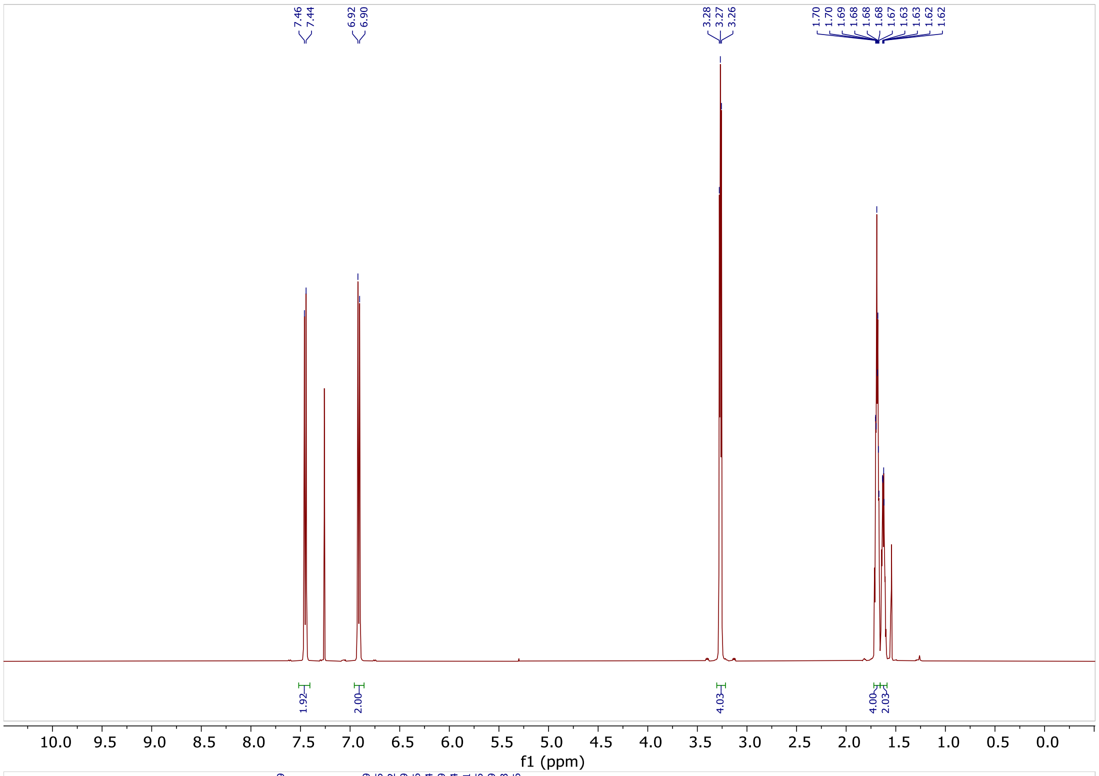

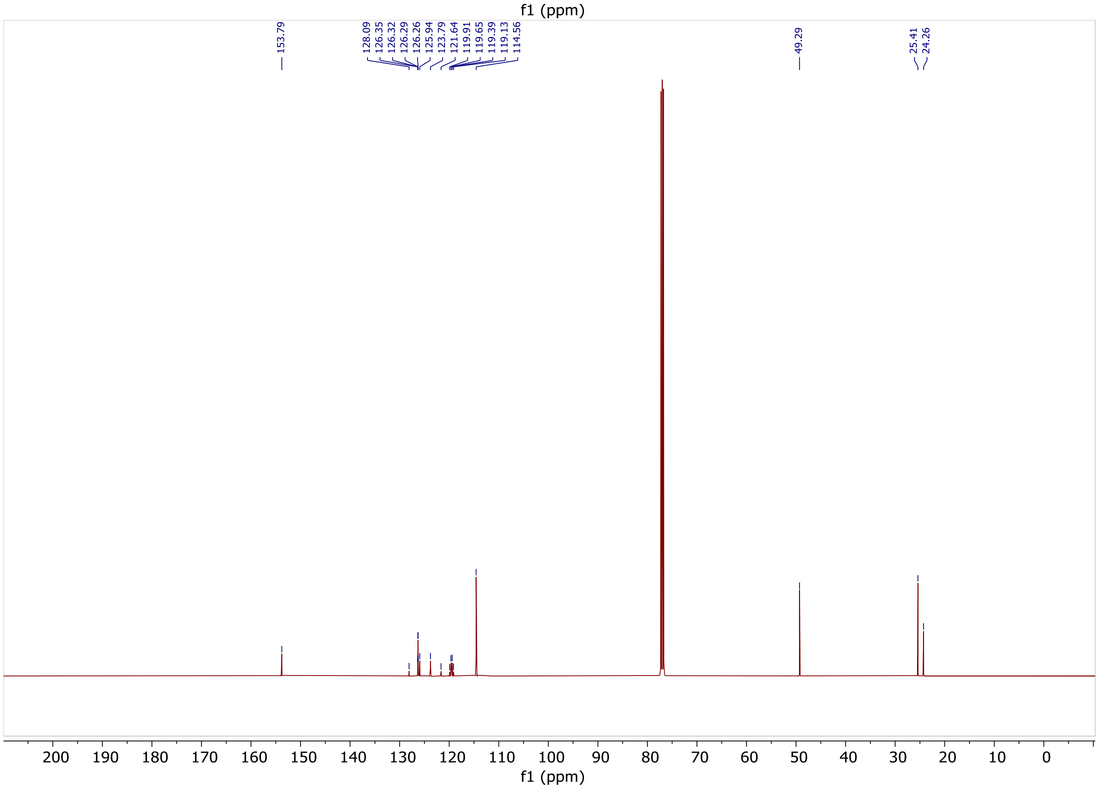

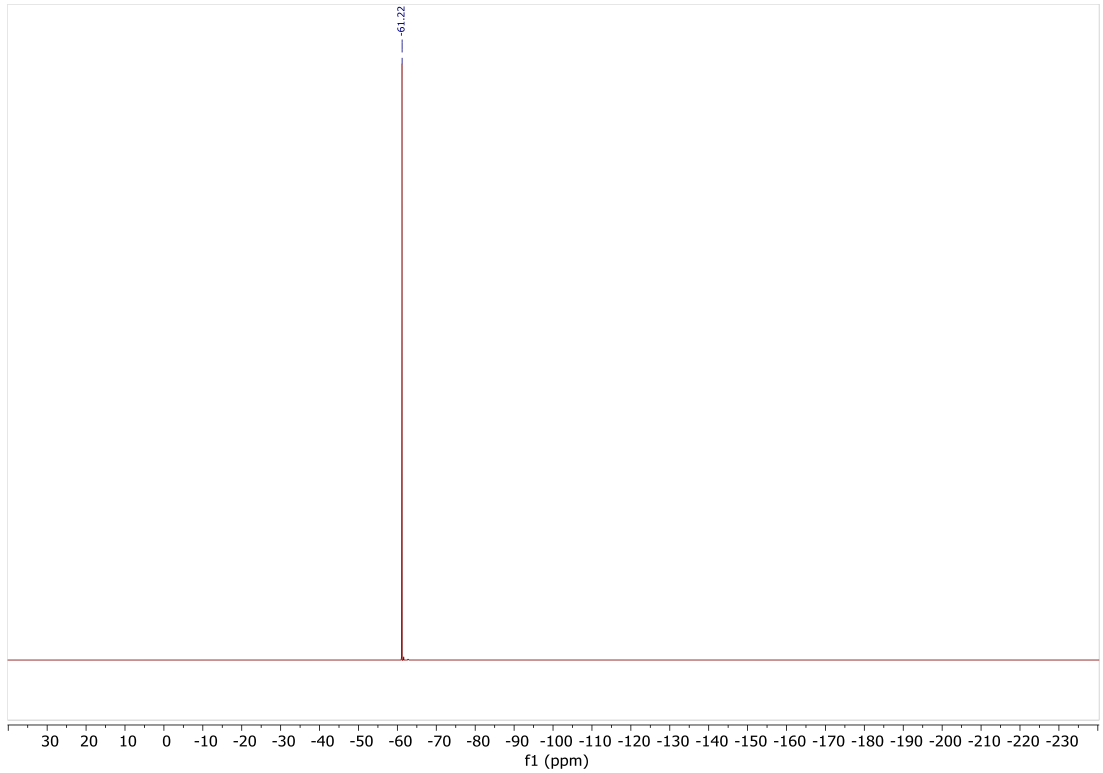
-61.22
30 20 10 0 -10 -20 -30 -40 -50 -60 -70 -80 -90 -100 -110 -120 -130 -140 -150 -160 -170 -180 -190 -200 -210 -220 -230
f1 (ppm)

1-(3-trifluoromethyl)phenyl piperidine (2a)

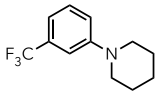

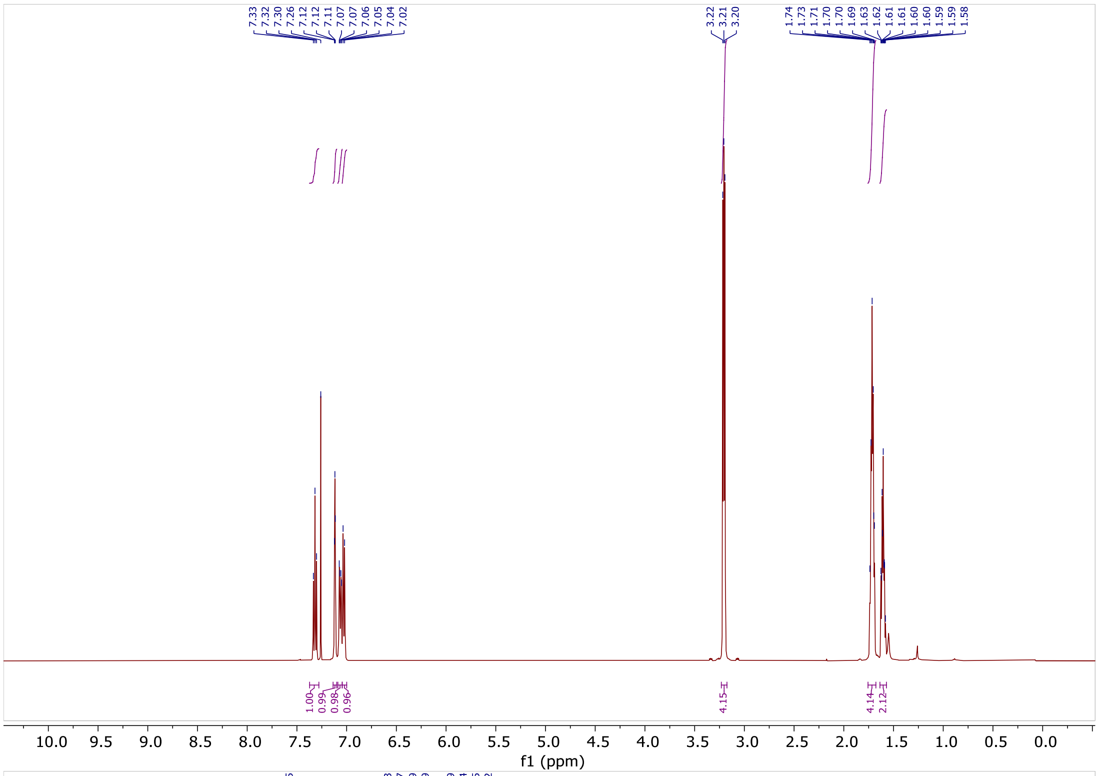

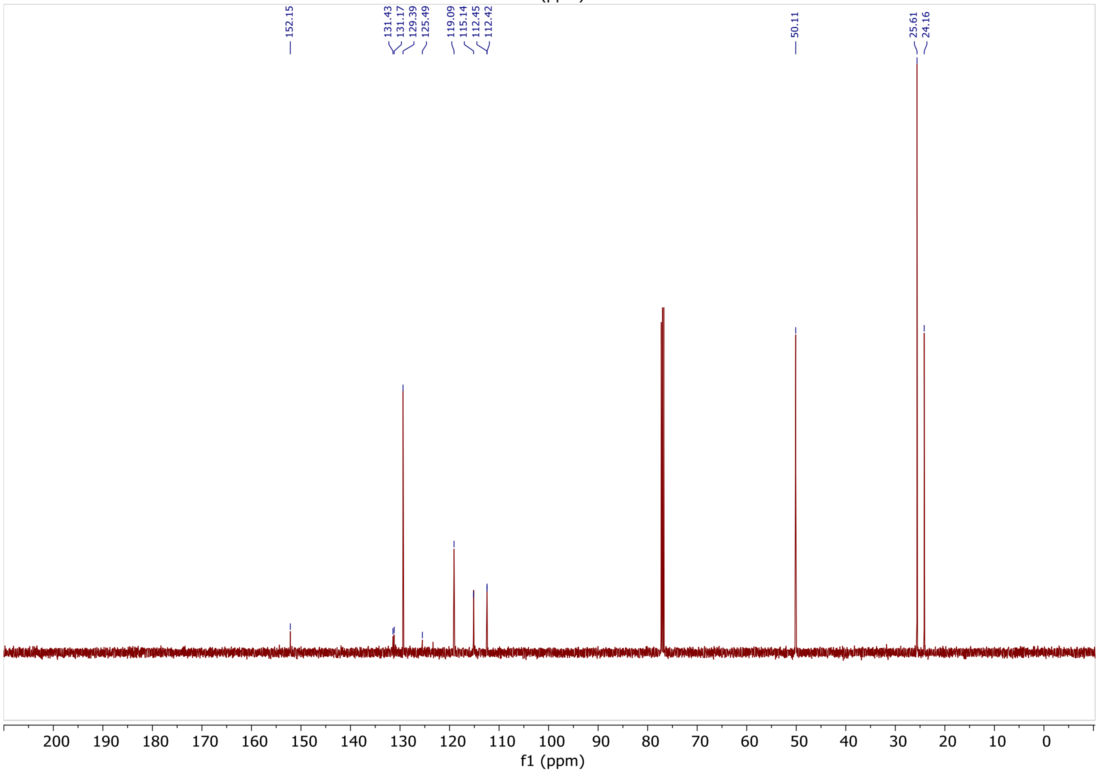

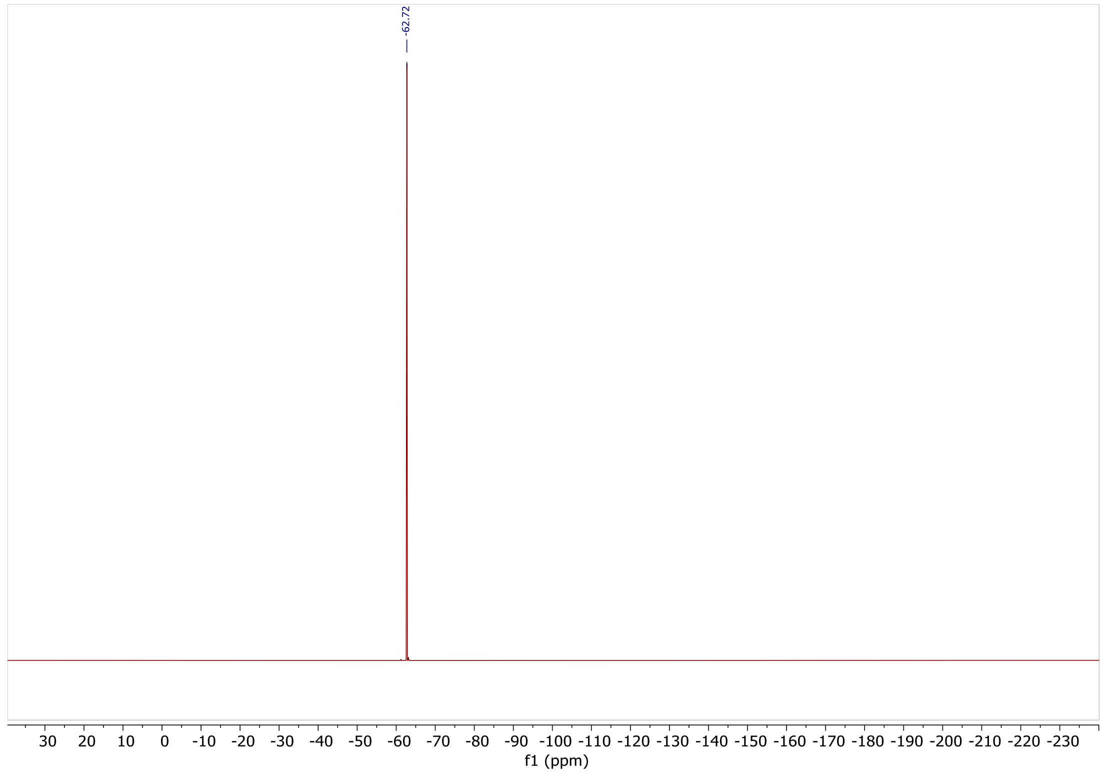
-62.72
30 20 10 0 -10 -20 -30 -40 -50 -60 -70 -80 -90 -100 -110 -120 -130 -140 -150 -160 -170 -180 -190 -200 -210 -220 -230
f1 (ppm)

1-(2-trifluoromethyl)phenyl piperidine (3a)

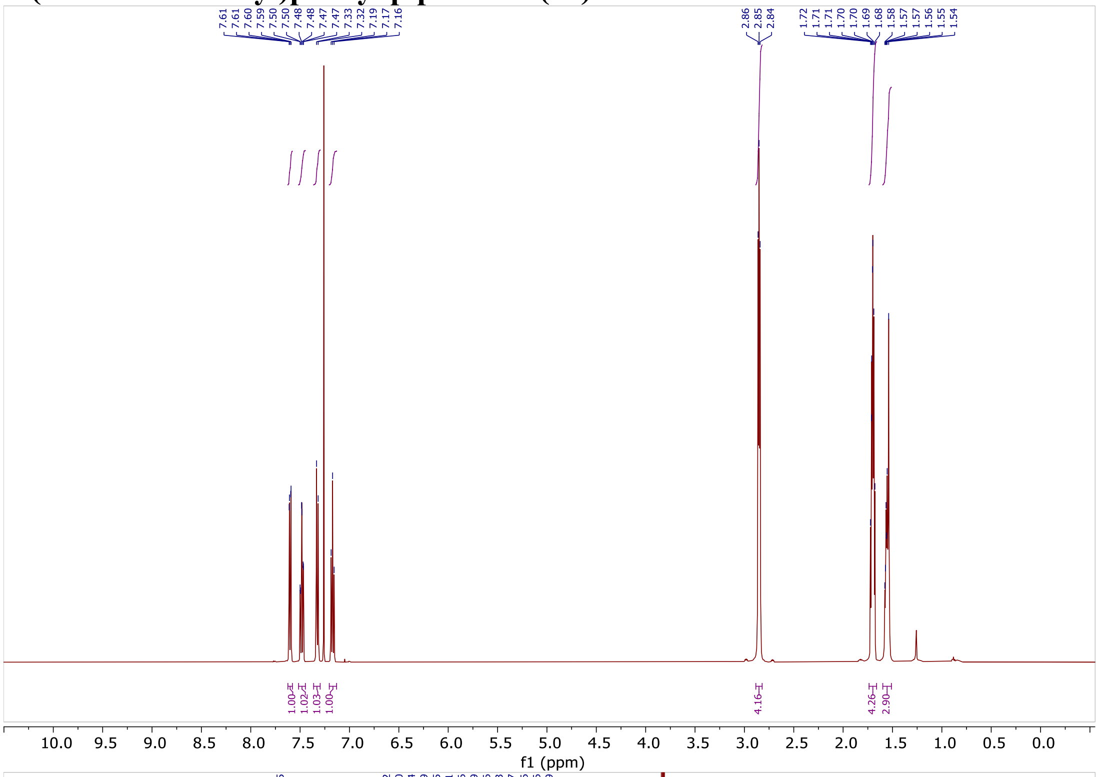

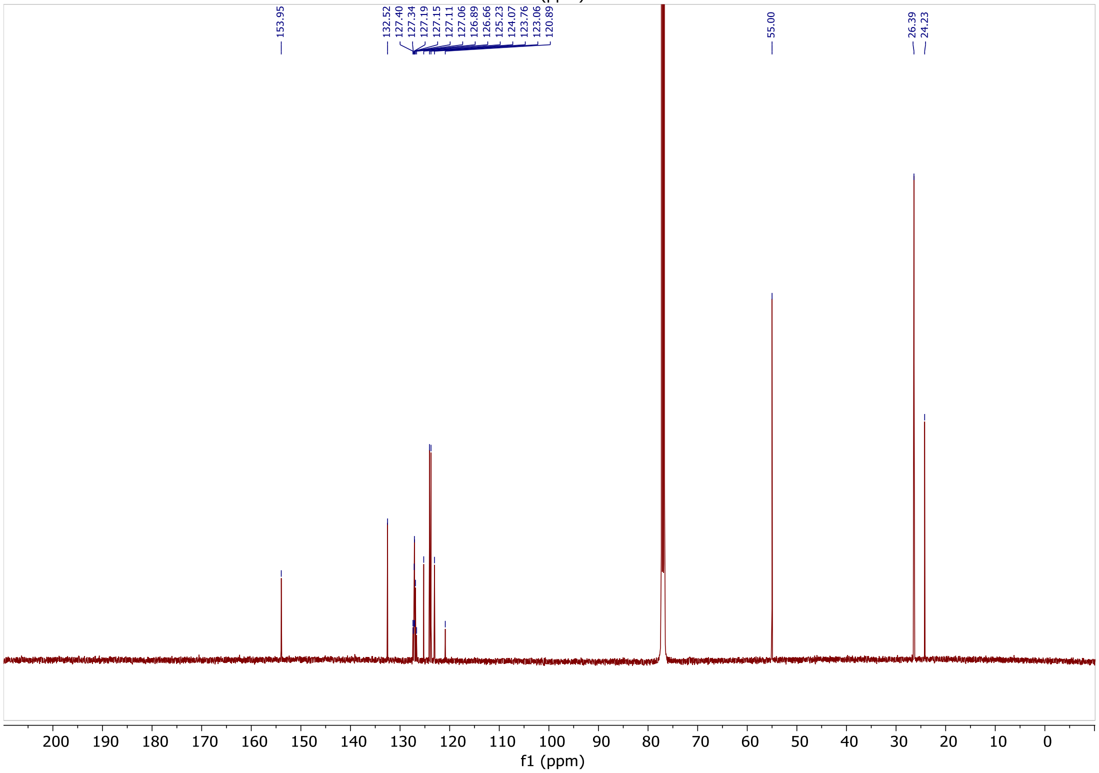

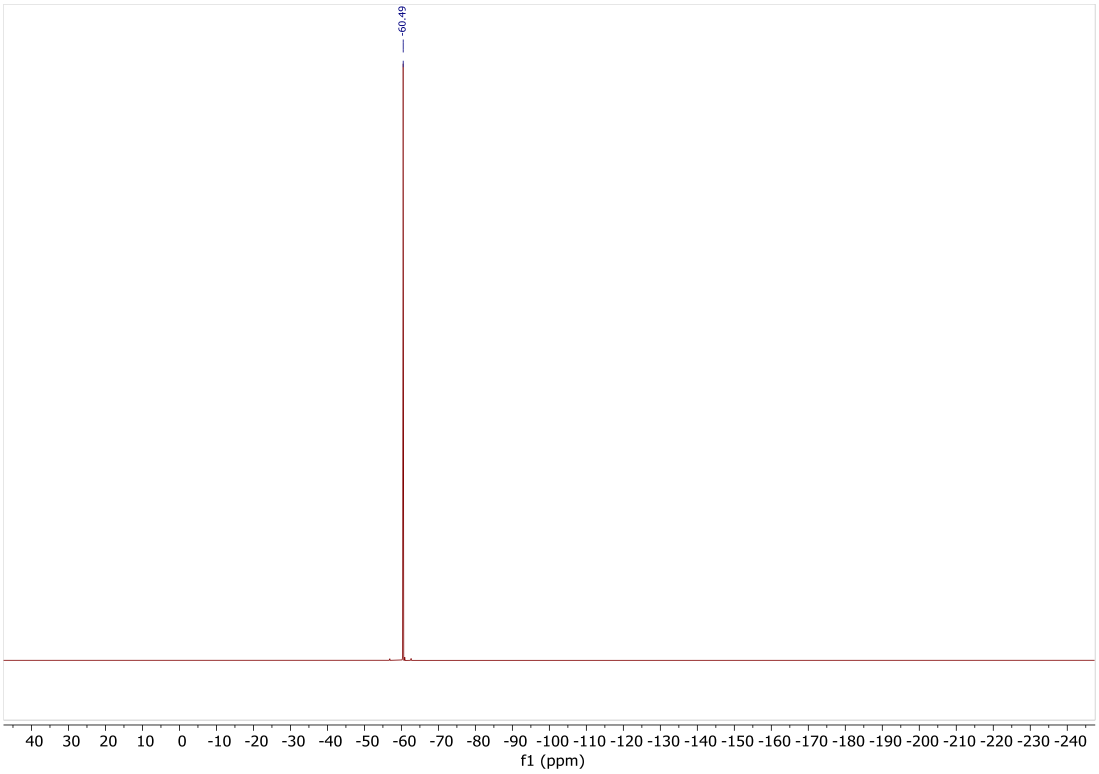
-60.49
40 30 20 10 0 -10 -20 -30 -40 -50 -60 -70 -80 -90 -100 -110 -120 -130 -140 -150 -160 -170 -180 -190 -200 -210 -220 -230 -240
f1 (ppm)

4-(piperidin-1-yl)benzonitrile (4a)

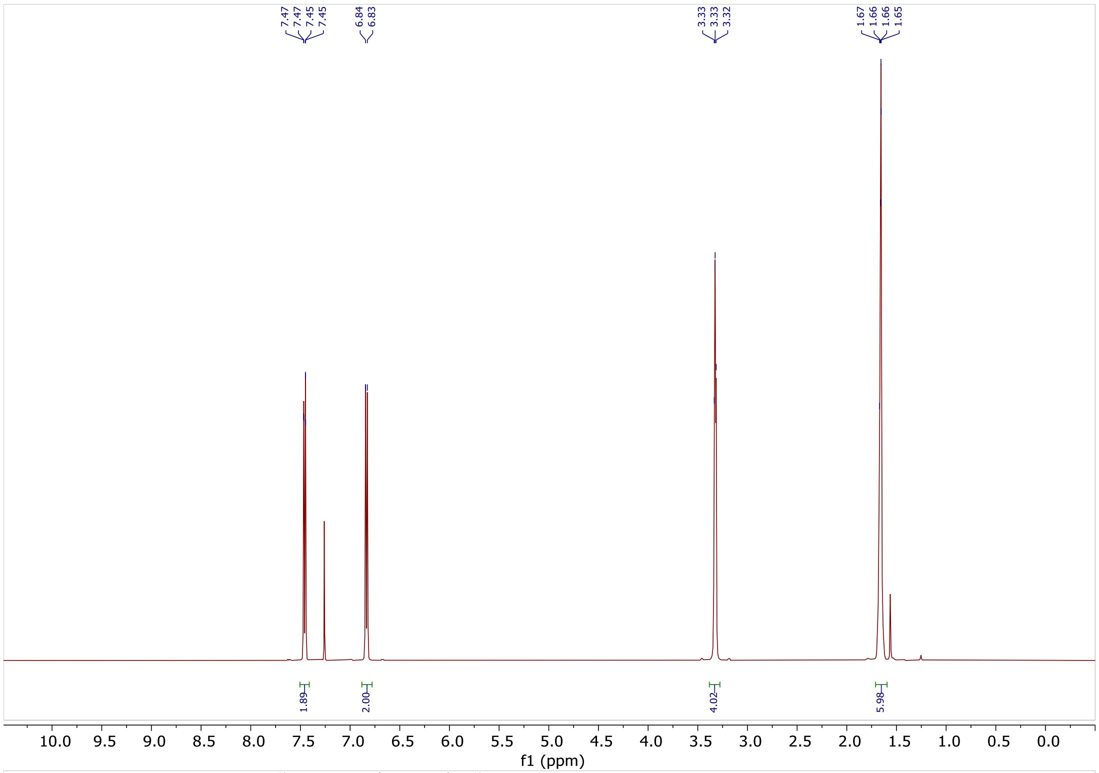

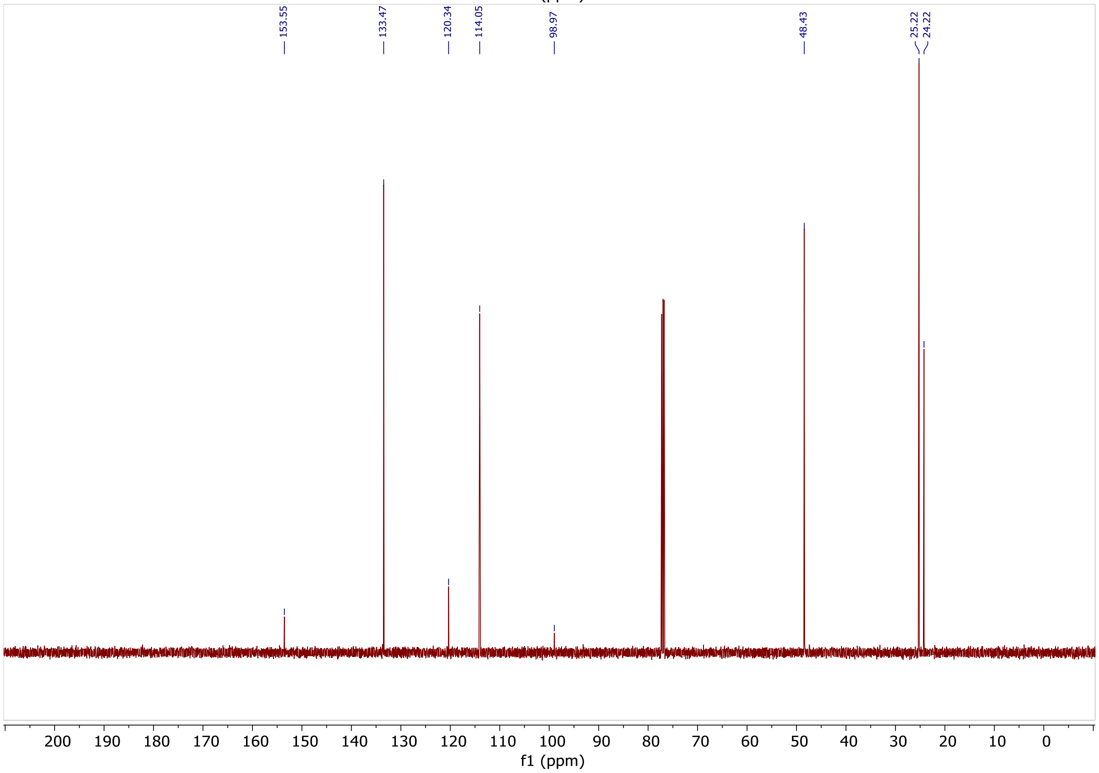

3-(piperidin-1-yl)benzonitrile (5a)

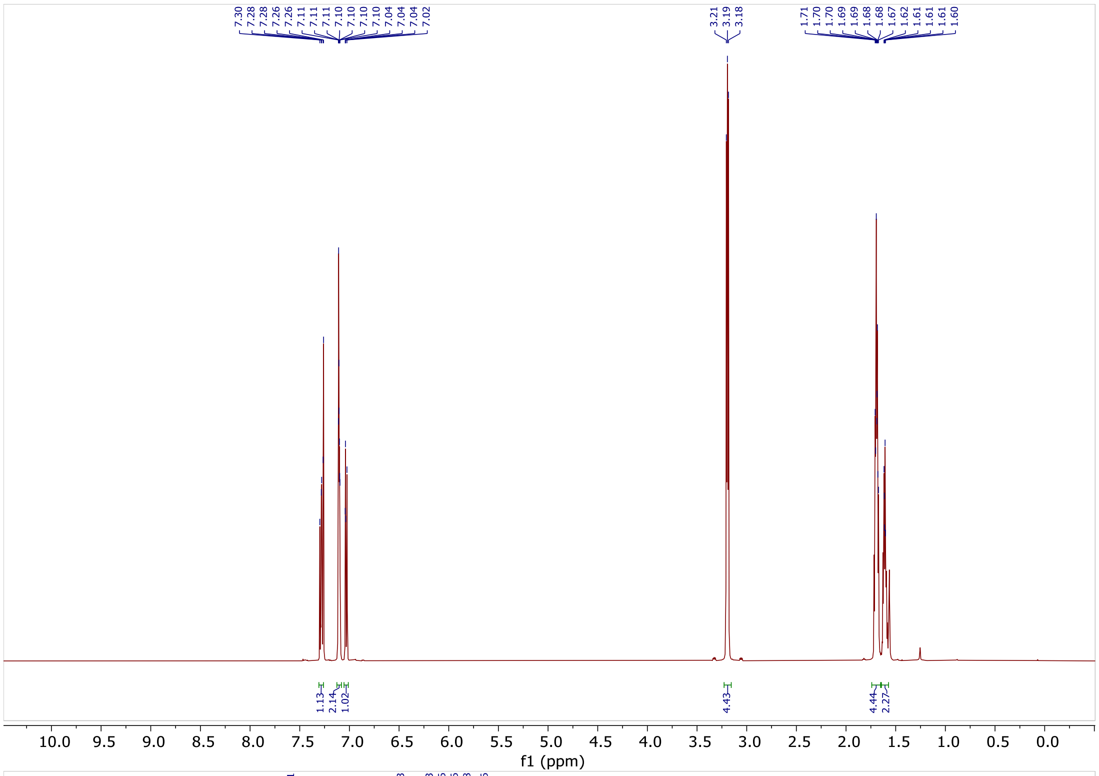

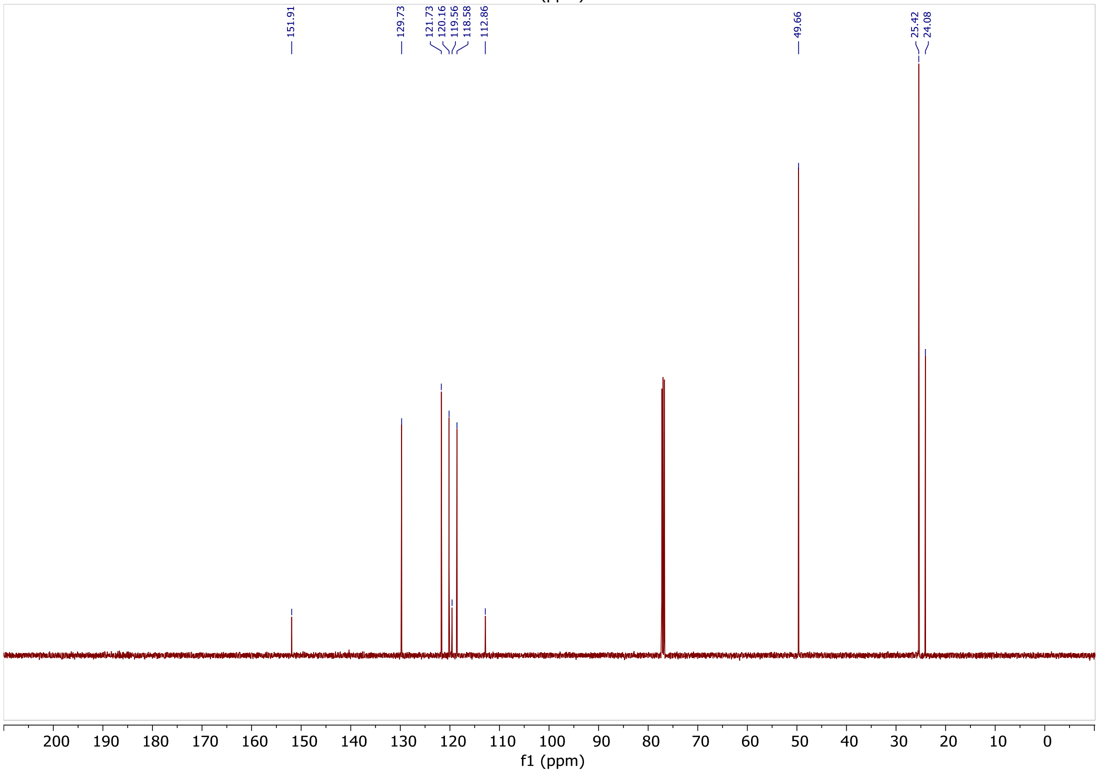

2-(piperidin-1-yl)benzonitrile (6a)

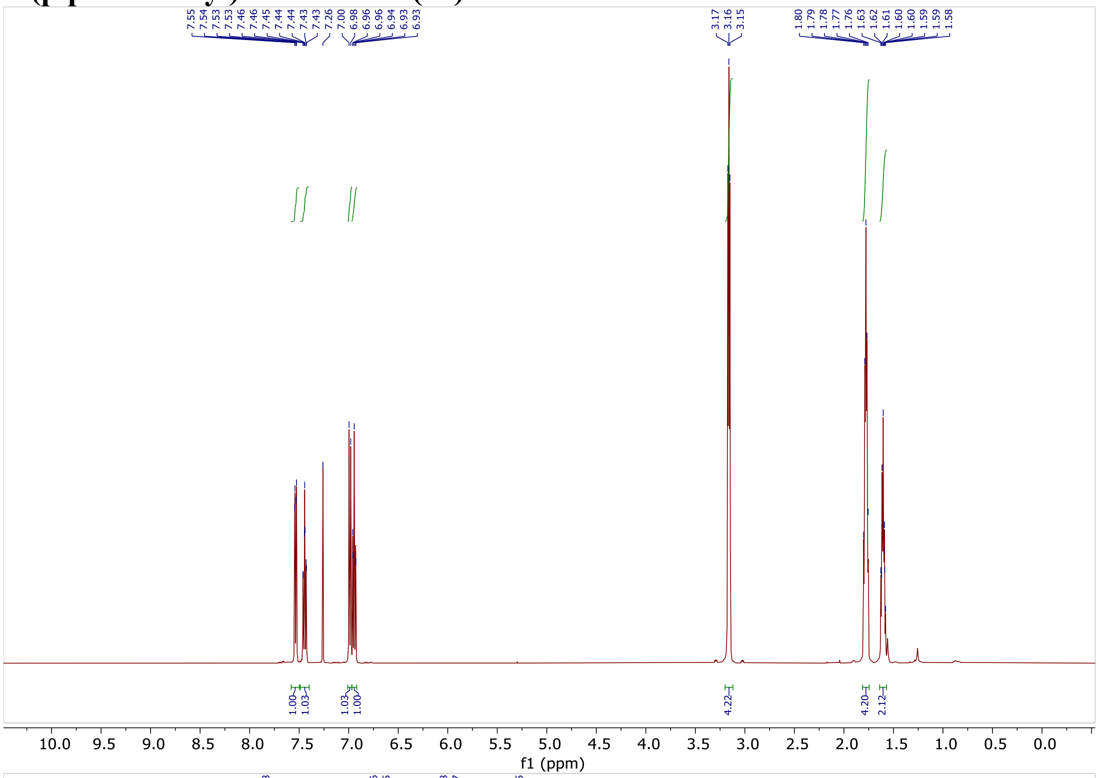

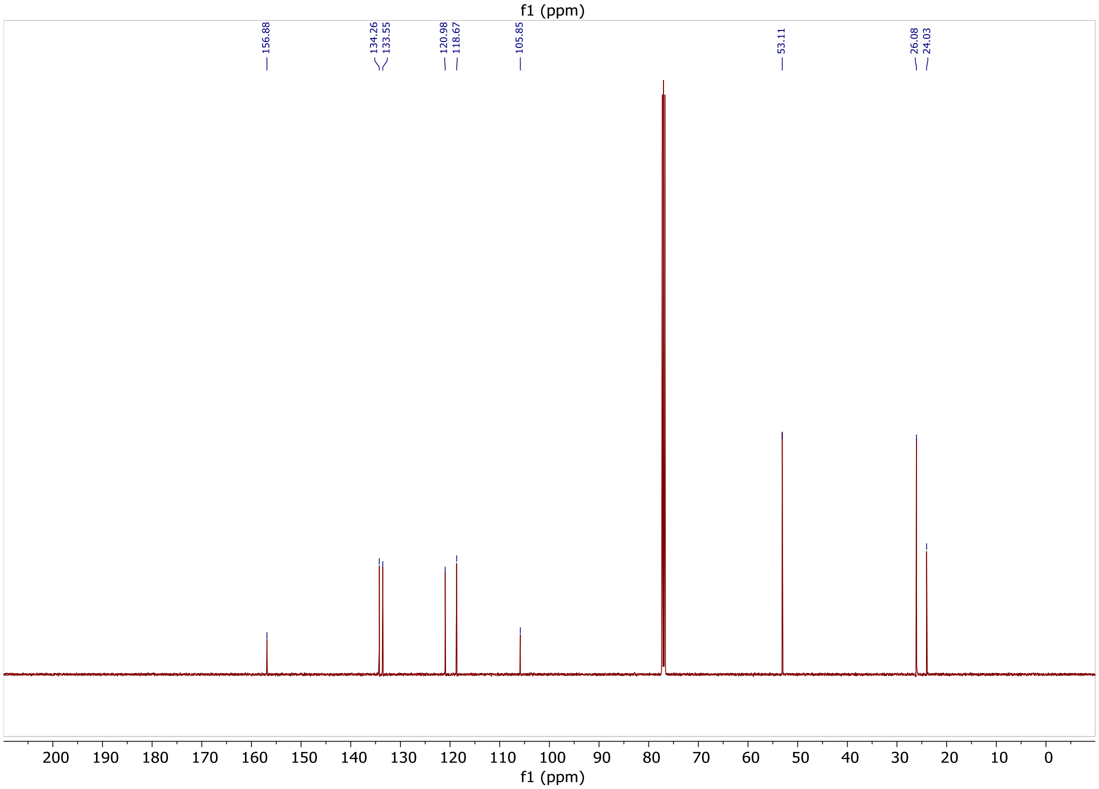

N-phenyl-2-(piperidin-1-yl)benzamide (7a)

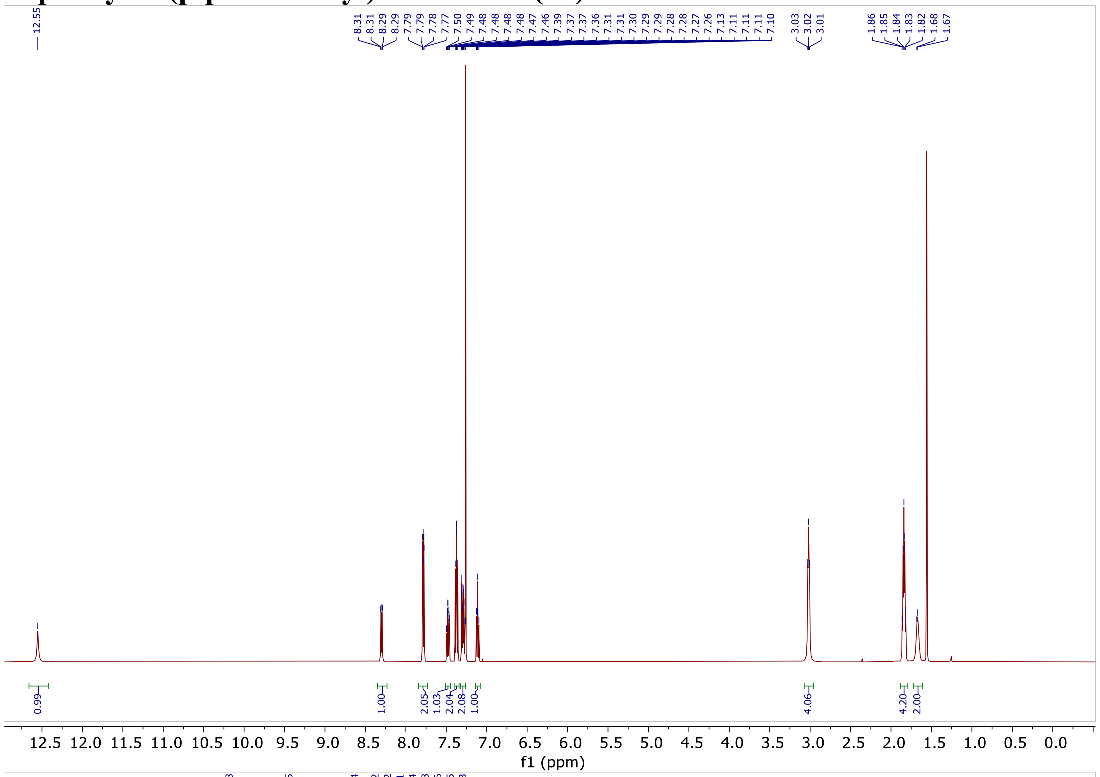

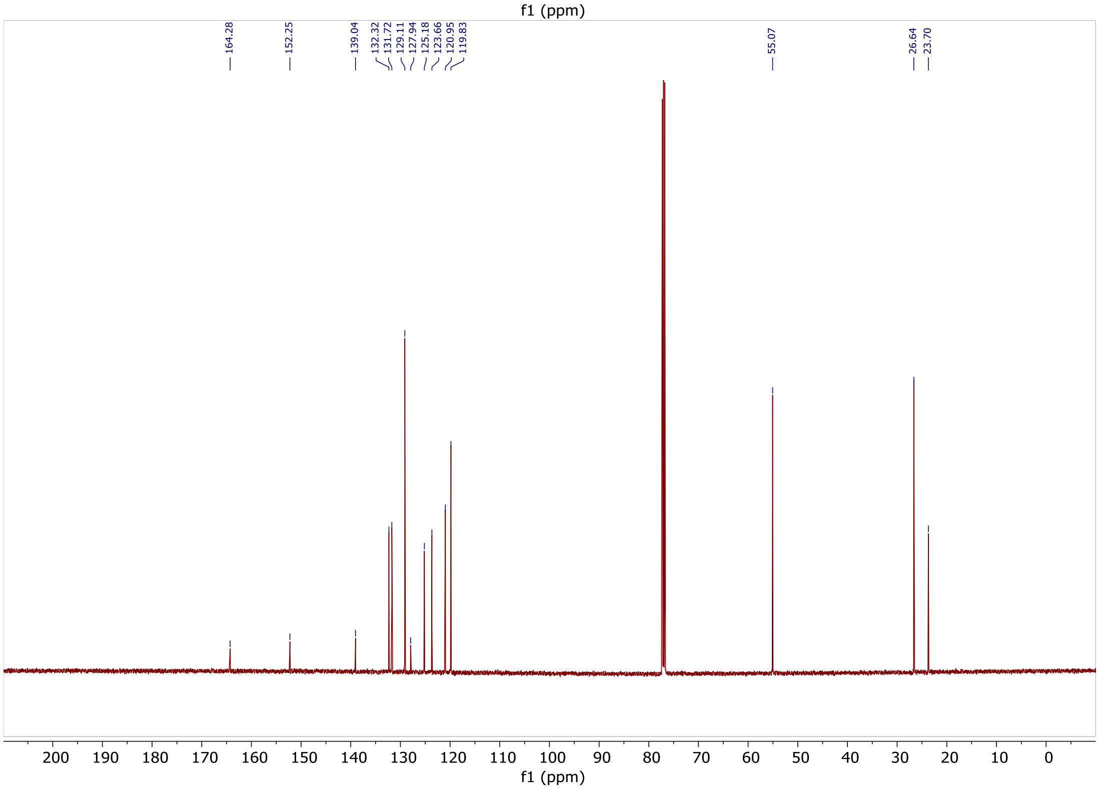

1-(4-Trimethylsilanylethynyl-phenyl)-piperidine (8a)

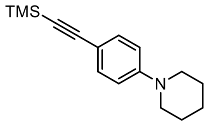

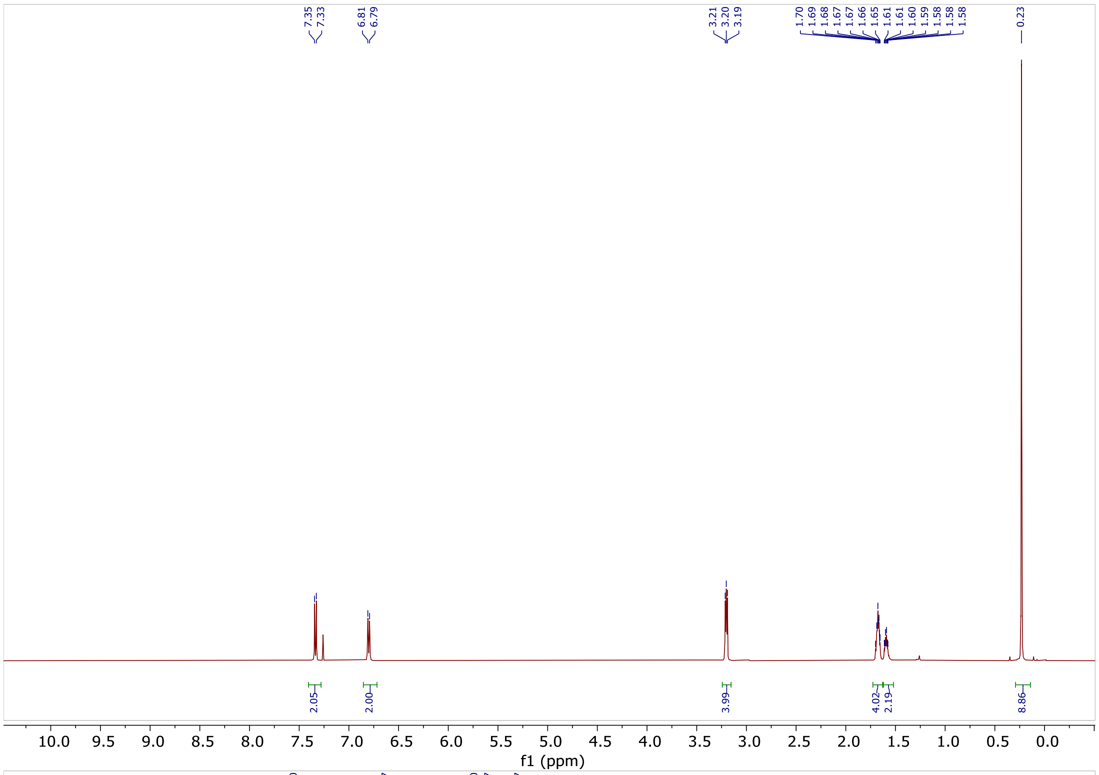

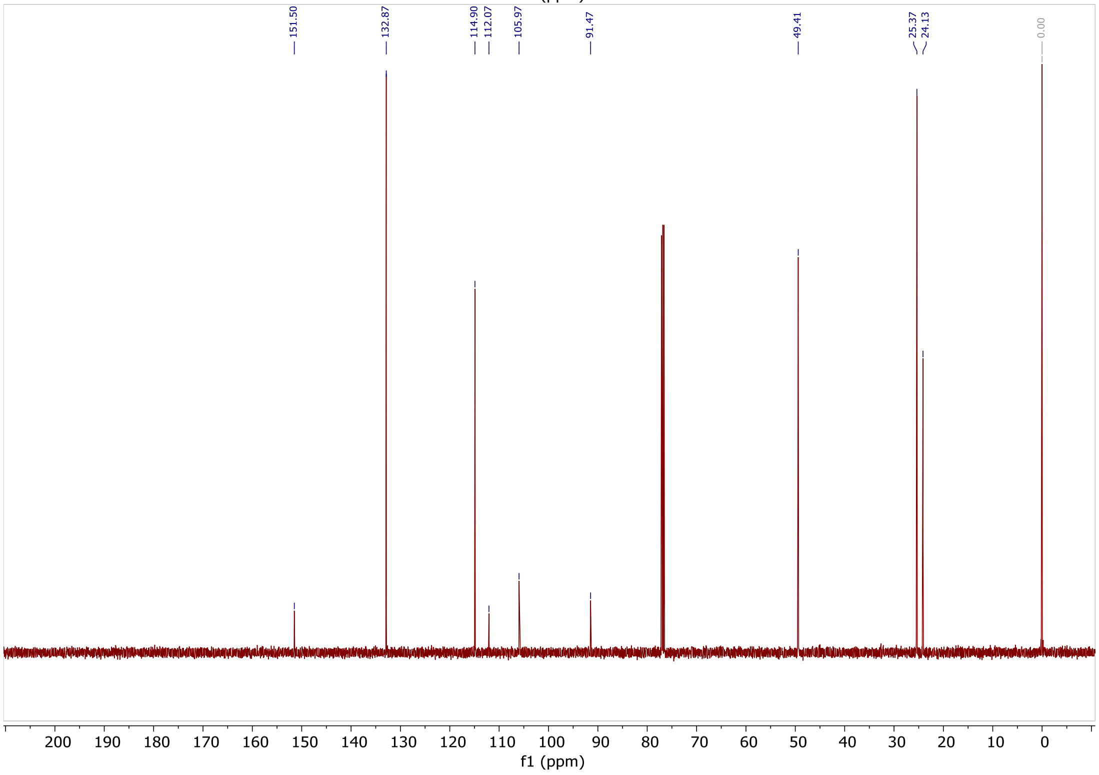

5-(piperidin-1-yl)-2-benzofuran-1(3H)-one (9a)

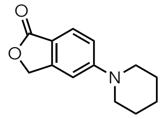

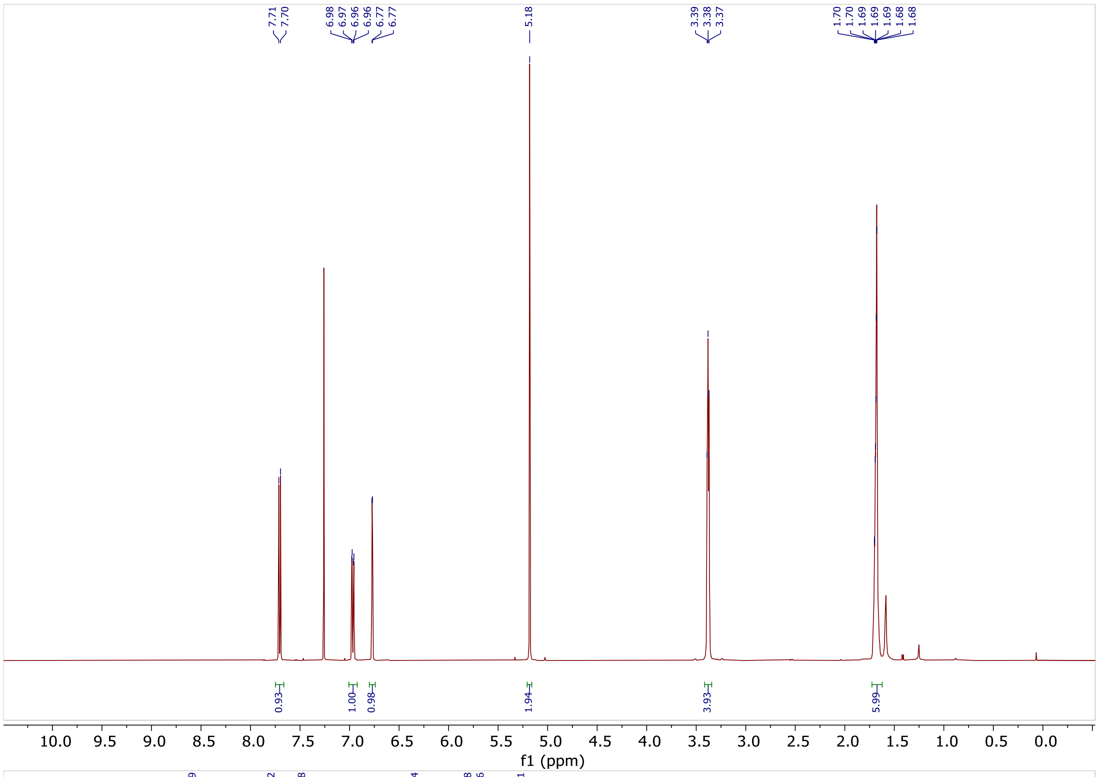

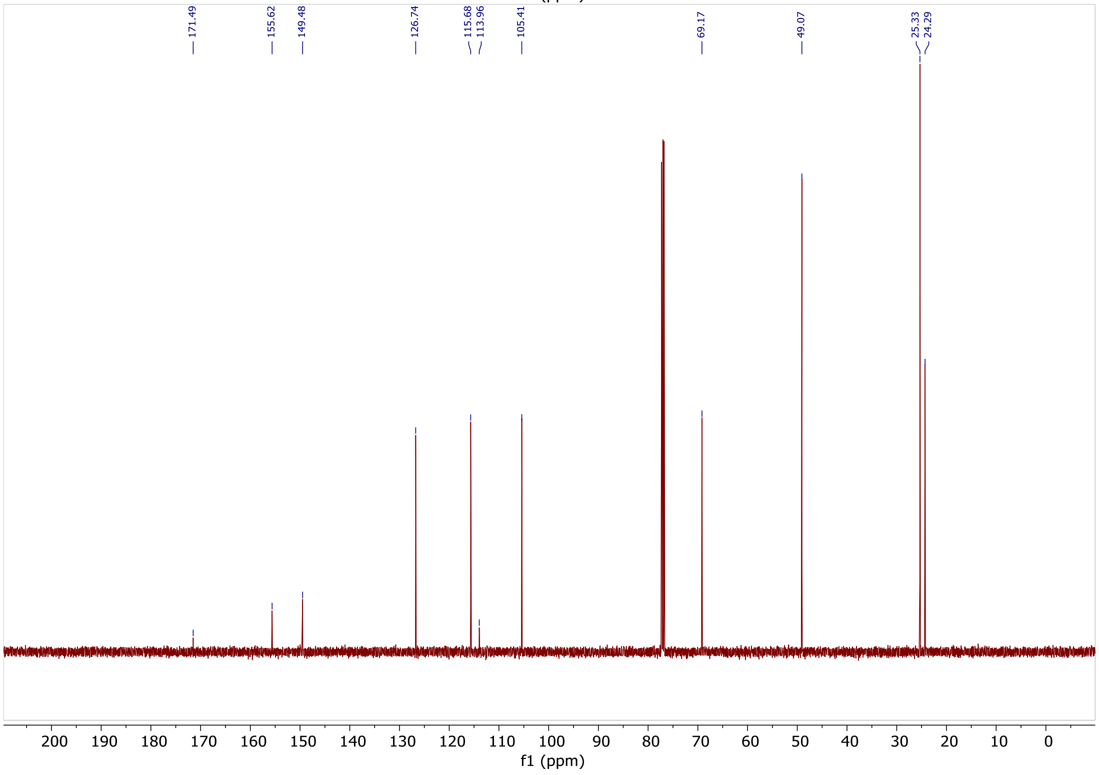

5-methyl-2-(piperidin-1-yl)pyridine (10a)

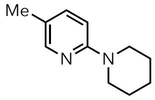

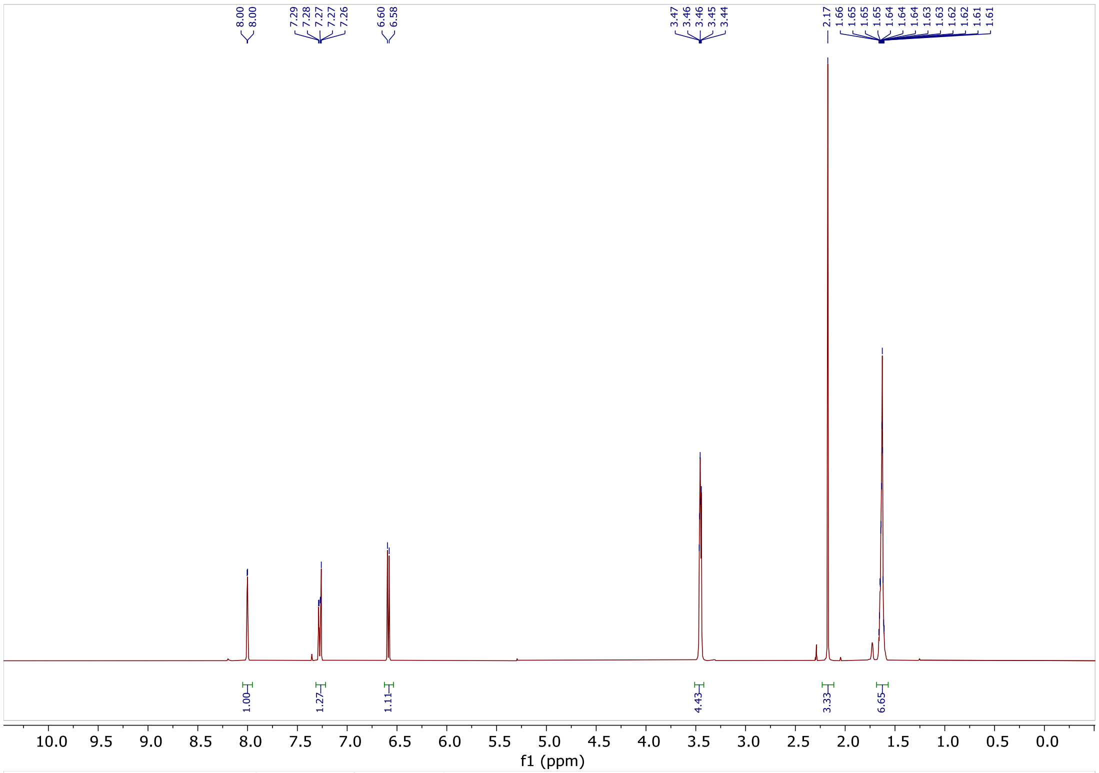

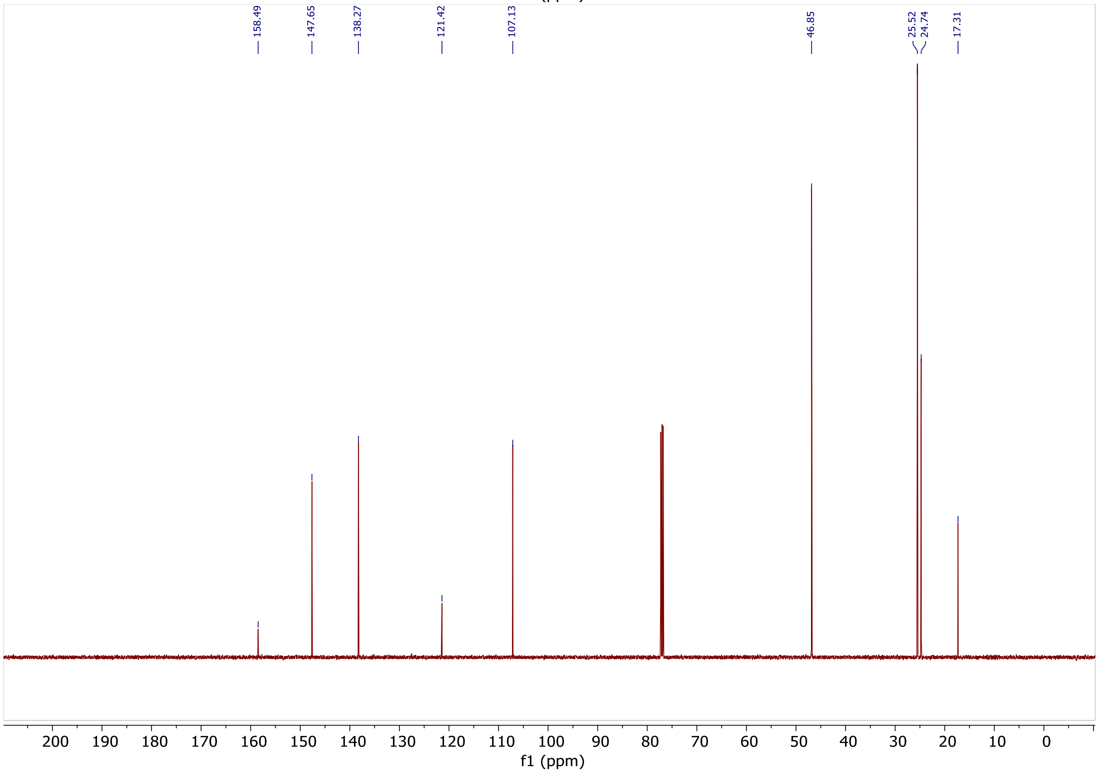

3-methyl-2-(piperidin-1-yl)pyridine (11a)

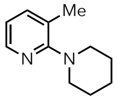

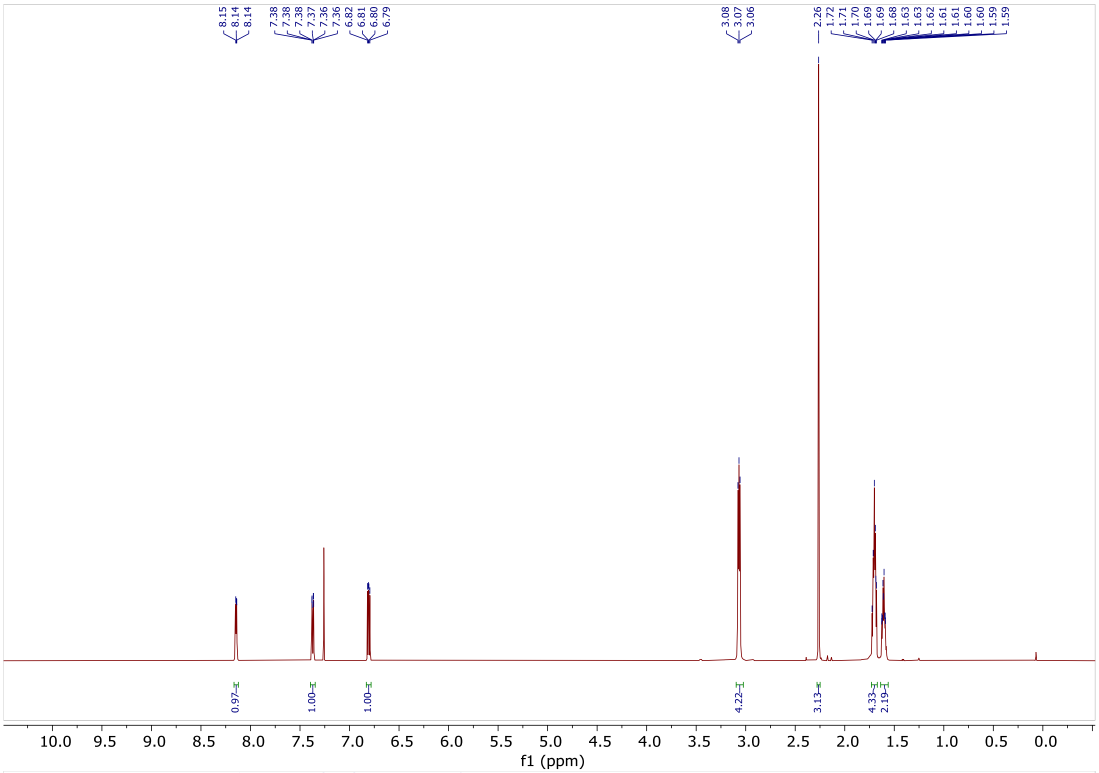

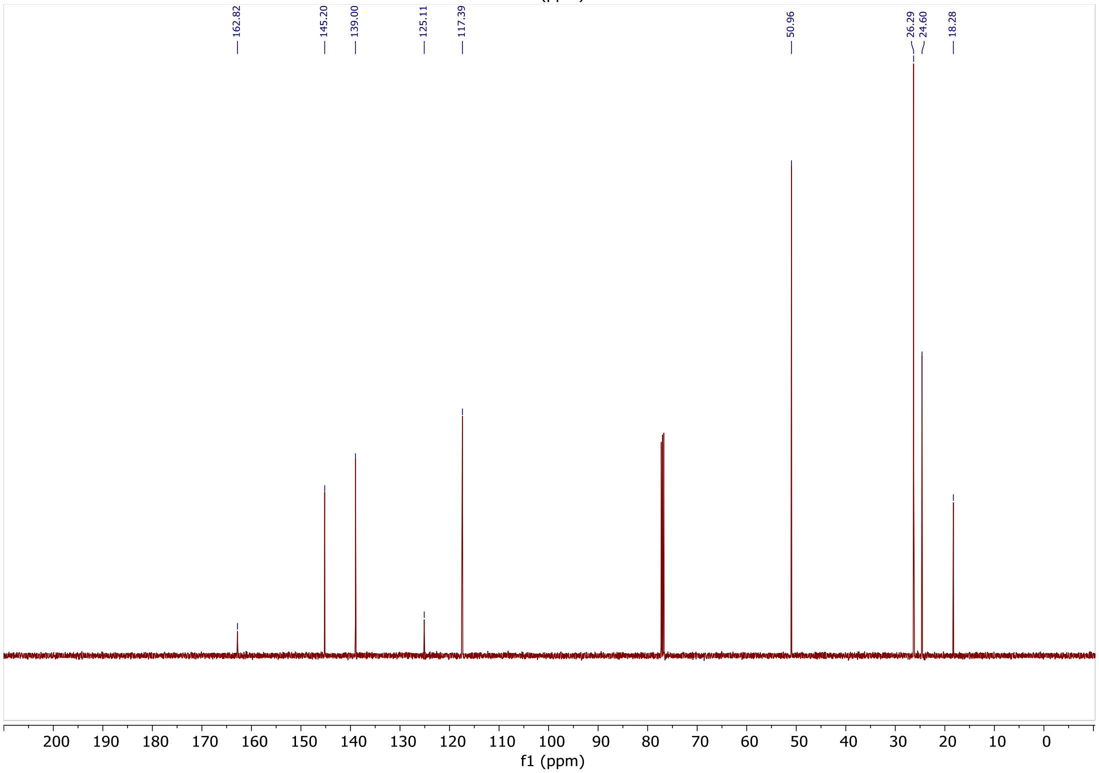

3-(piperidine-1-yl)pyrazine (12a)

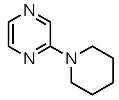

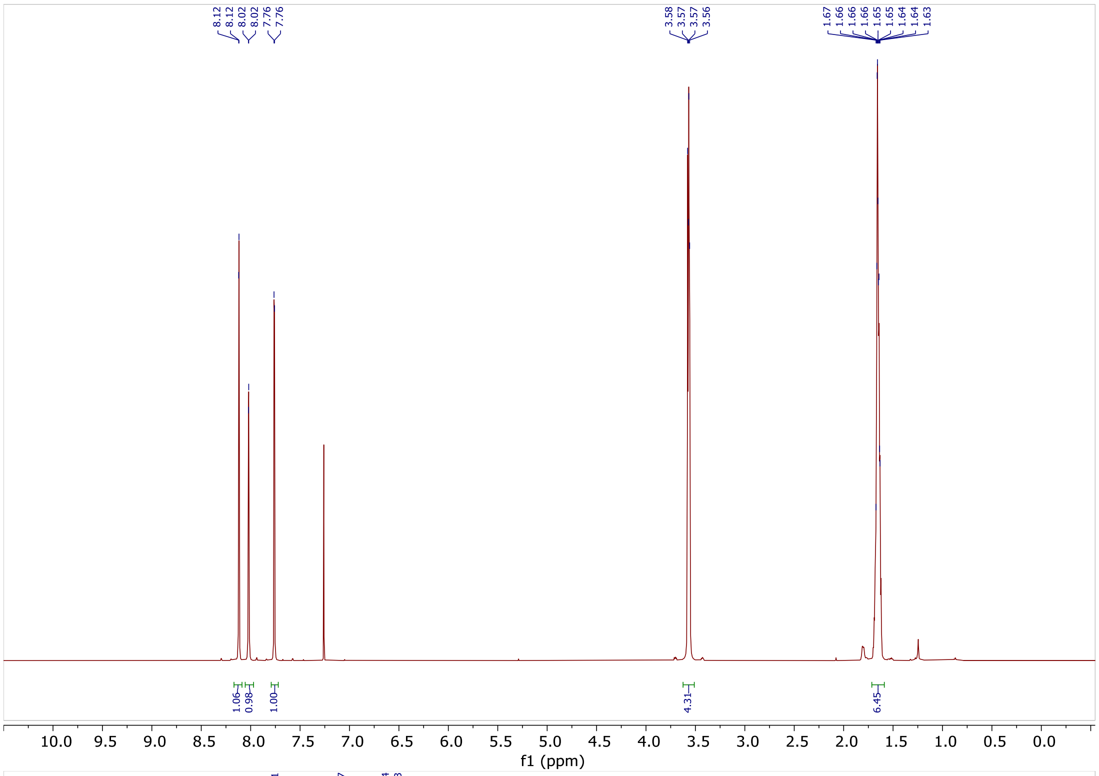

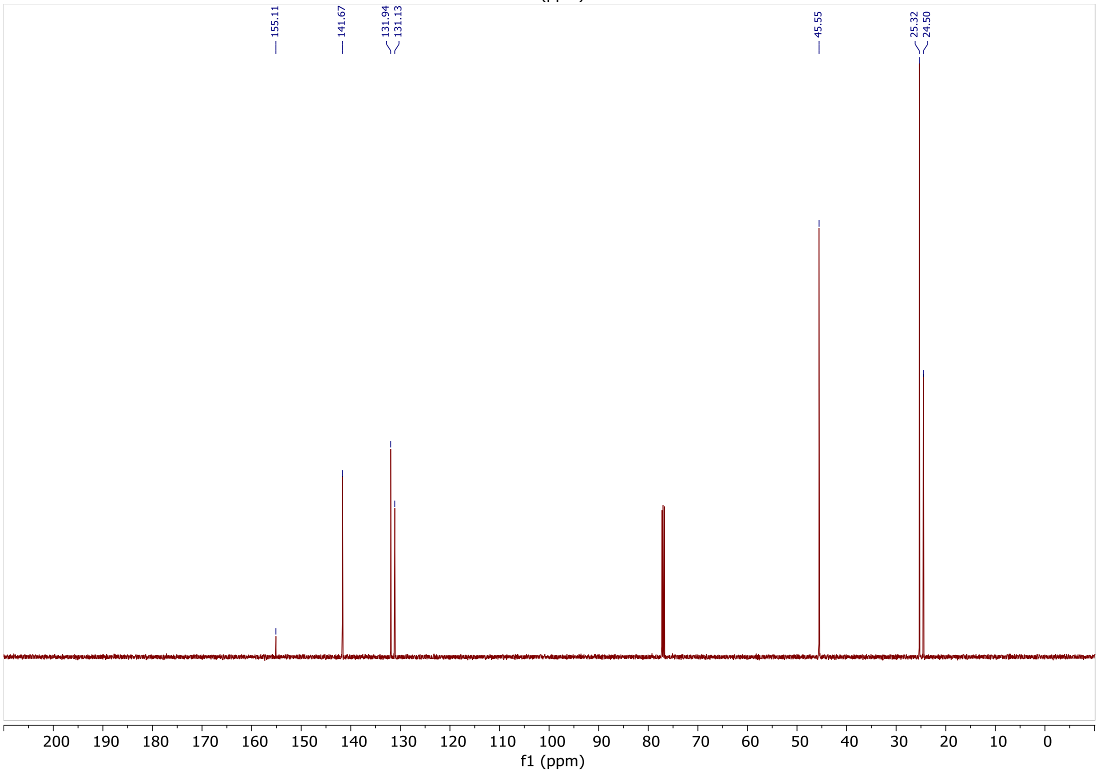

1-(4-trifluoromethylphenyl)pyrrolidine (1b)

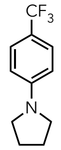

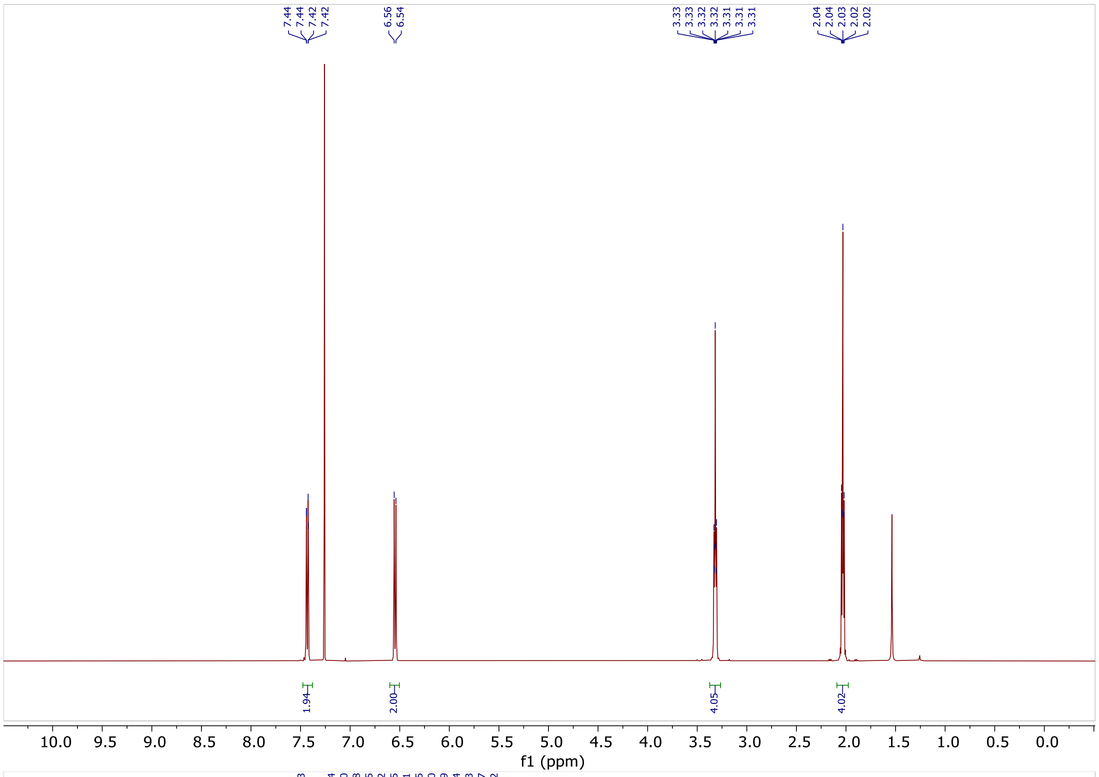

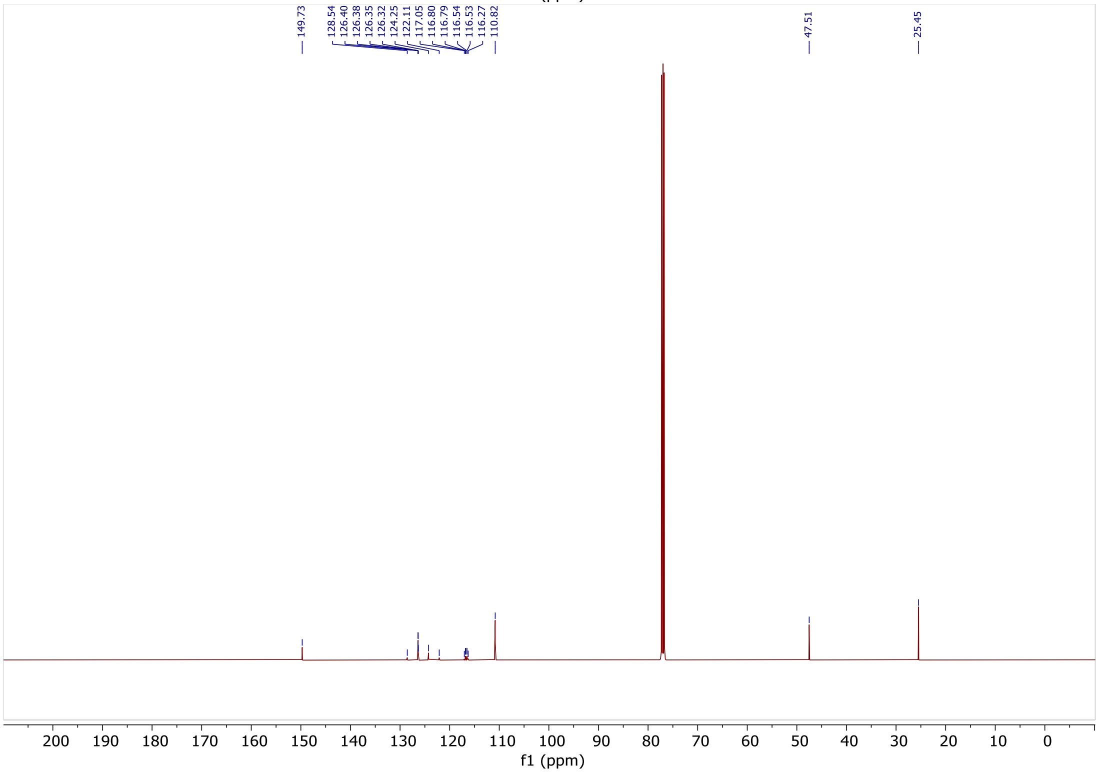

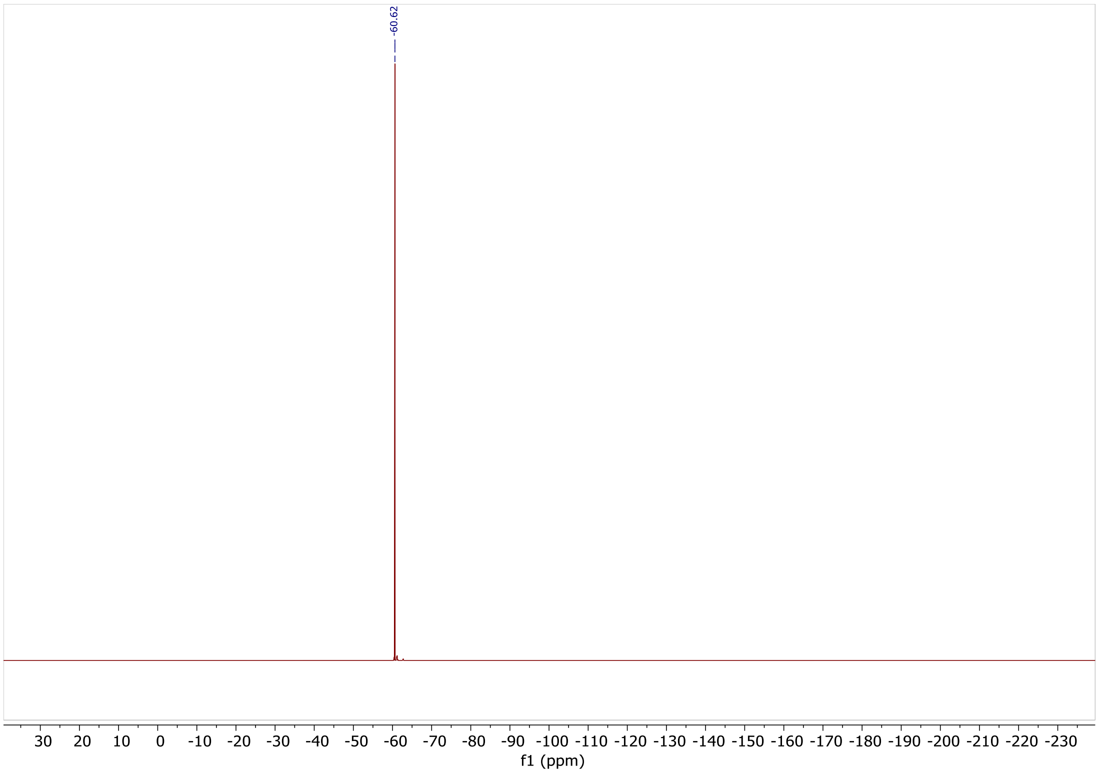
-60.62
30 20 10 0 -10 -20 -30 -40 -50 -60 -70 -80 -90 -100 -110 -120 -130 -140 -150 -160 -170 -180 -190 -200 -210 -220 -230
f1 (ppm)

1-methyl-4-(4-(trifluoromethyl)phenyl)piperazine (1c)

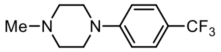

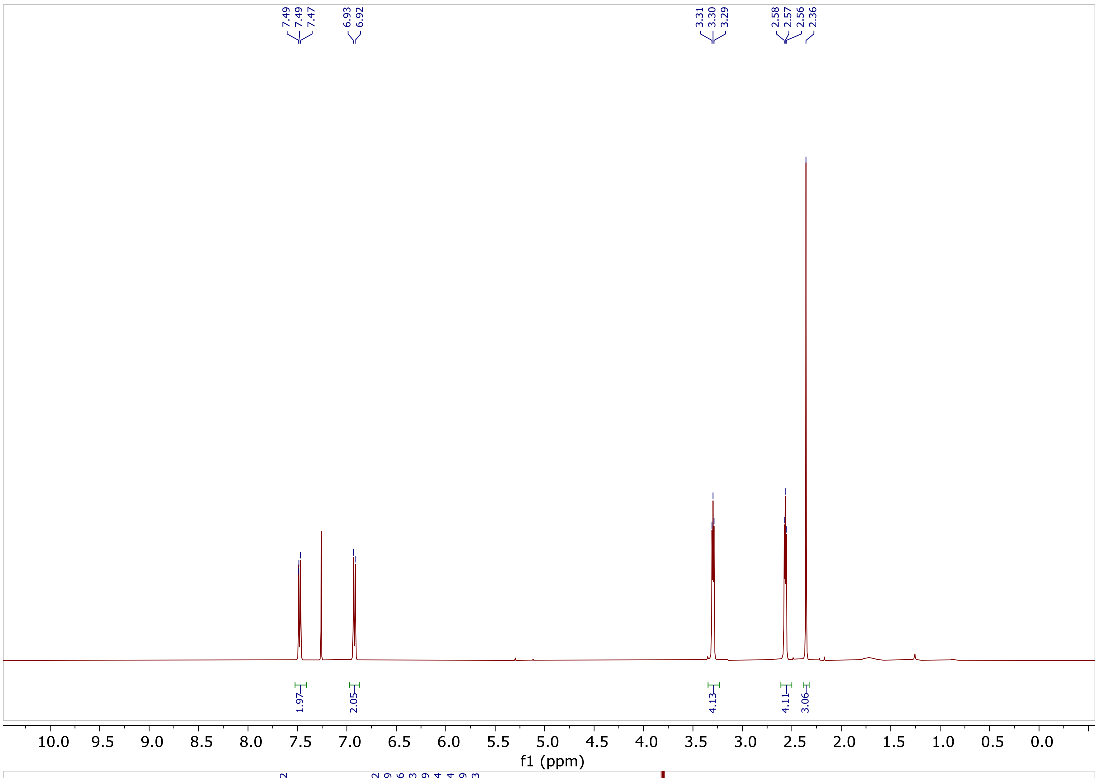

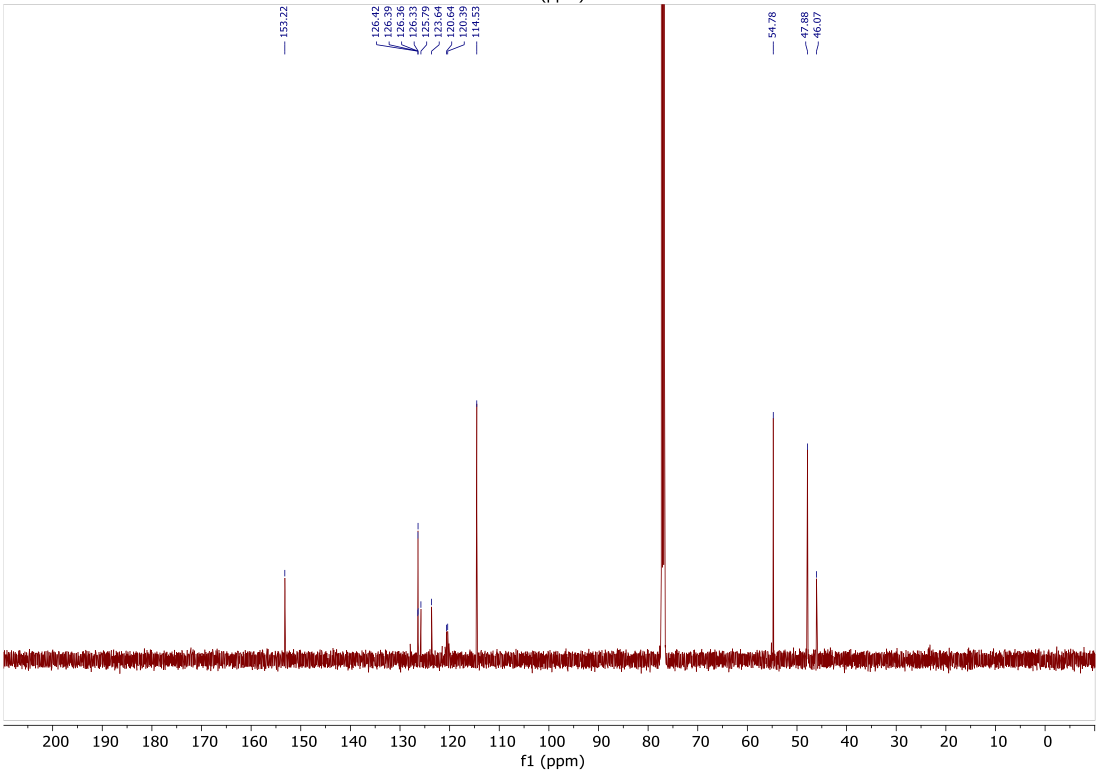

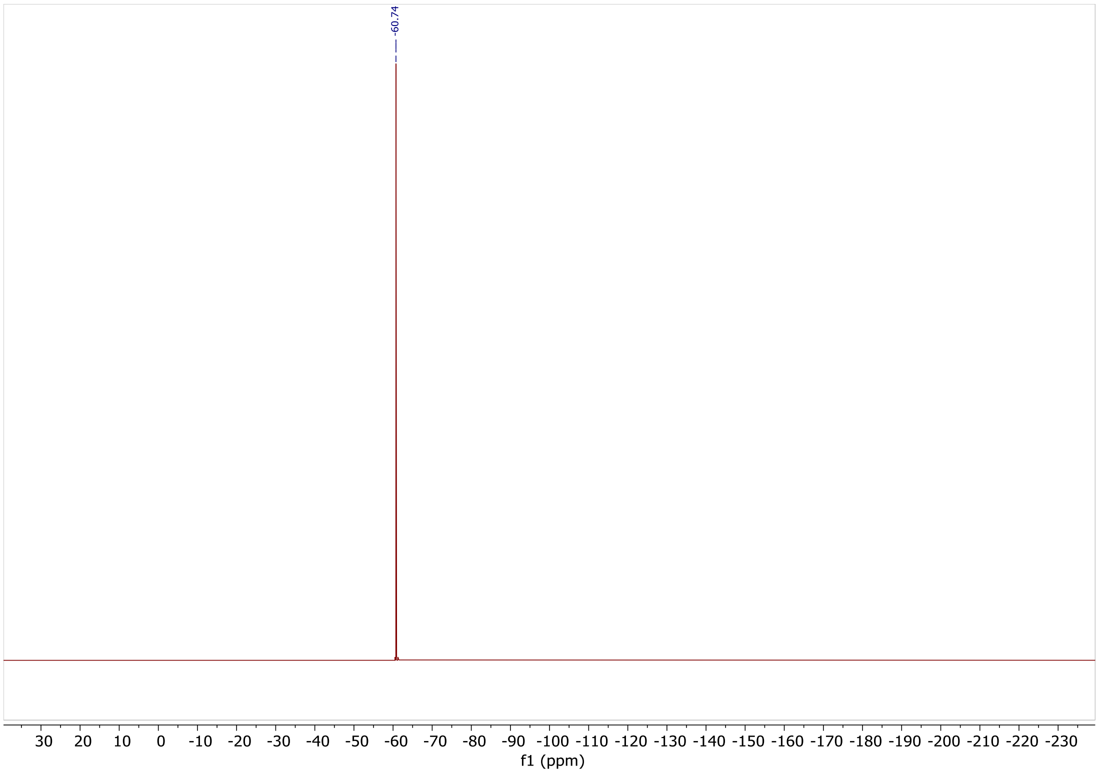

-60.74
30
20
10
0
-10
-20
-30
-40
-50
-60
-70
-80
-90
-100
-110
-120
-130
-140
-150
-160
-170
-180
-190
-200
-210
-220
-230
f1 (ppm)

4-(4-trifluoromethyl)phenyl)morpholine (1d)

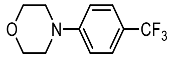

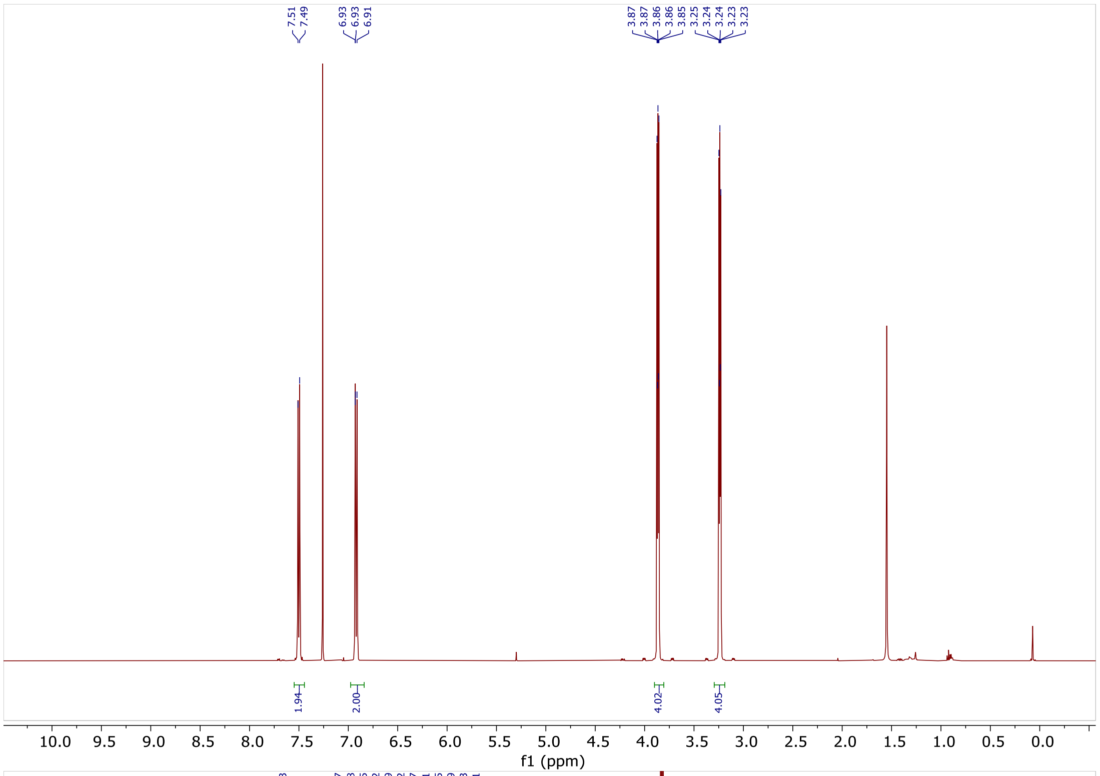

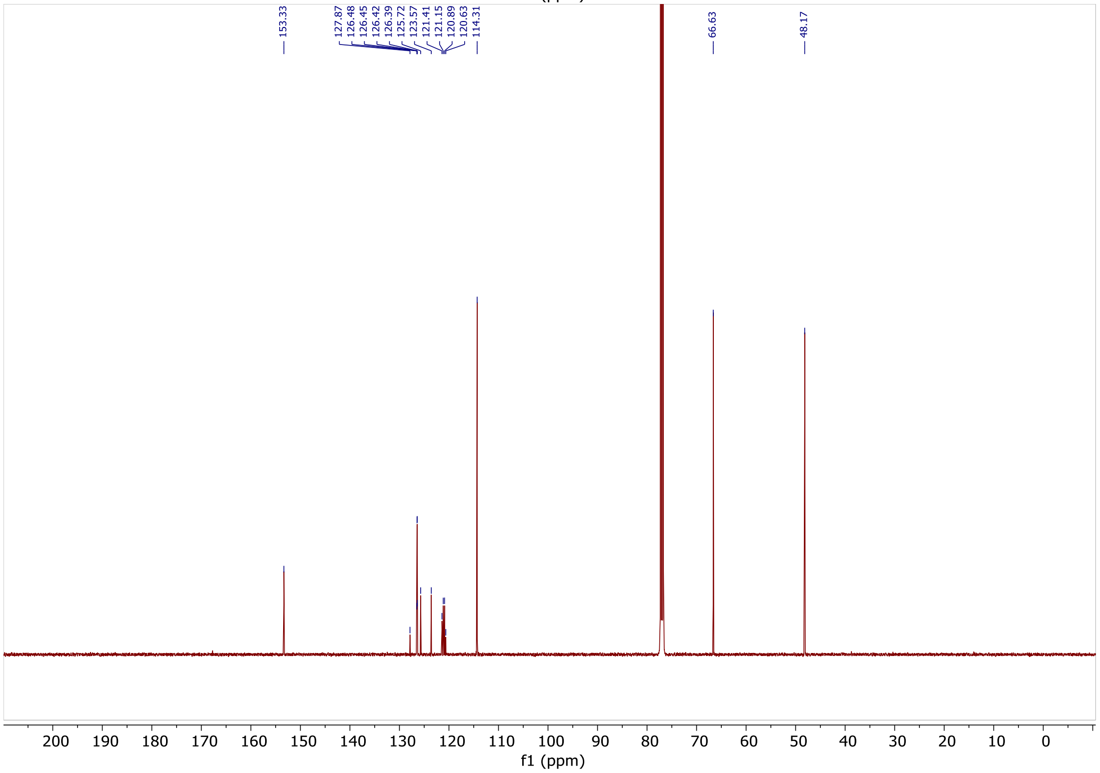

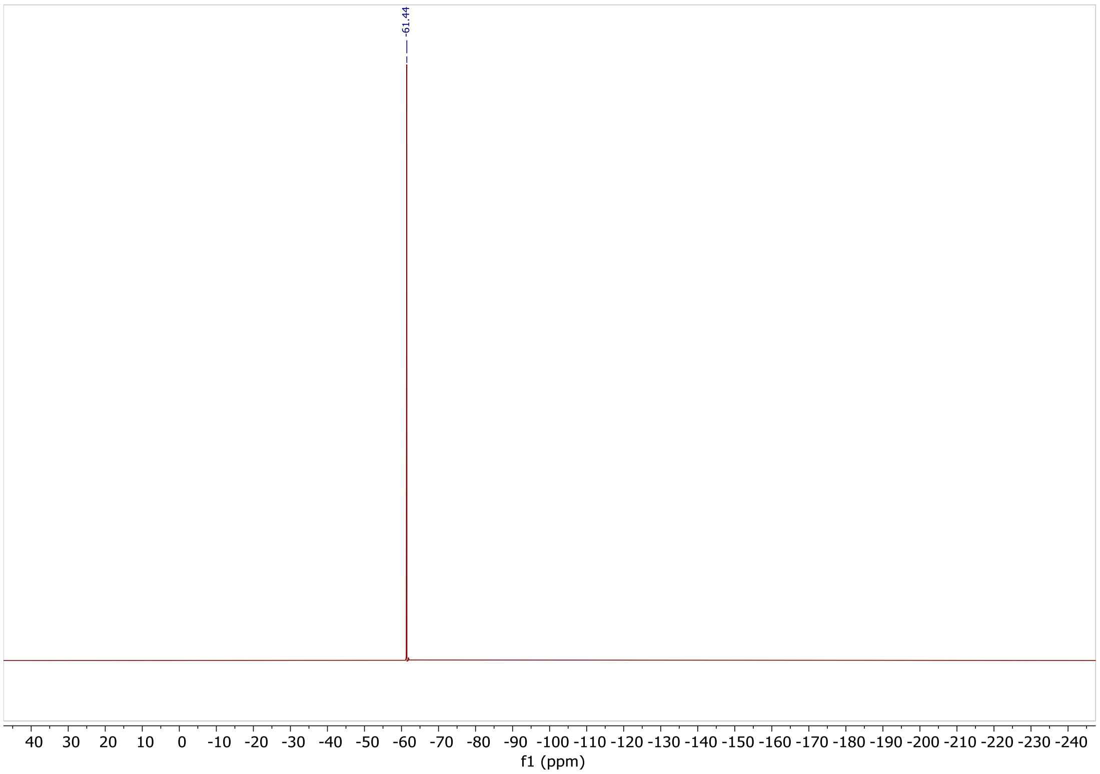

-61.44
40 30 20 10 0 -10 -20 -30 -40 -50 -60 -70 -80 -90 -100 -110 -120 -130 -140 -150 -160 -170 -180 -190 -200 -210 -220 -230 -240
f1 (ppm)

N-octyl-4-(trifluoromethyl)aniline (1e)

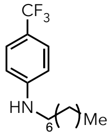

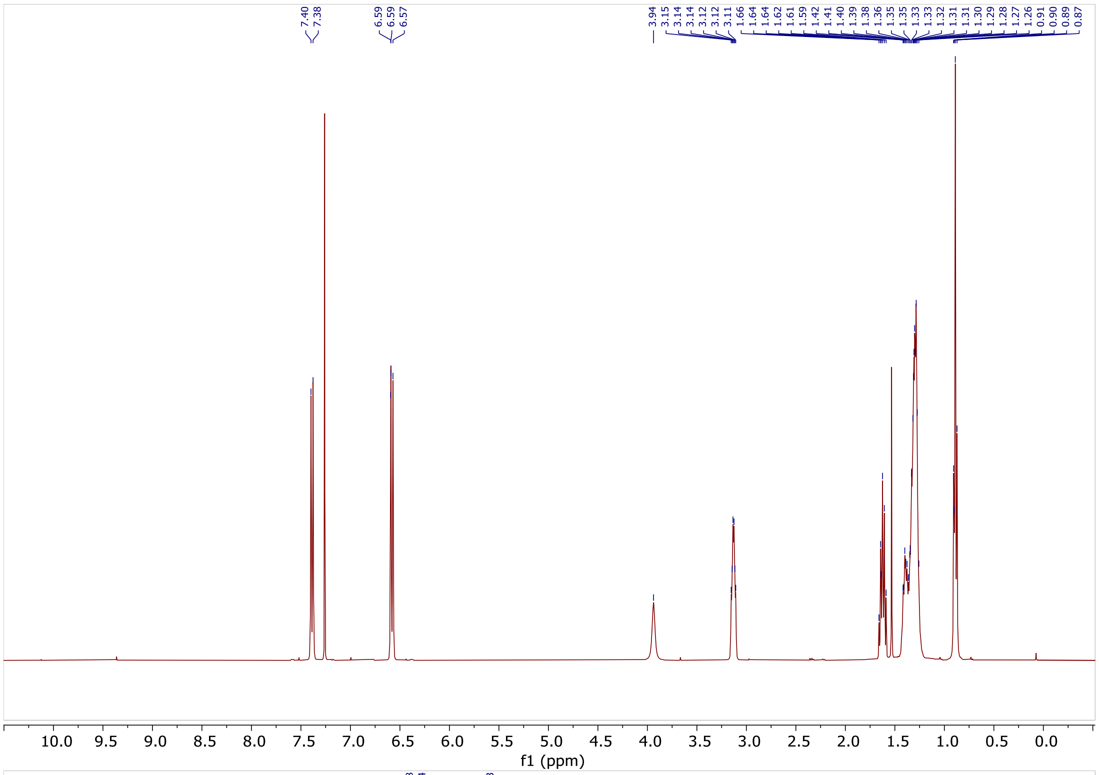

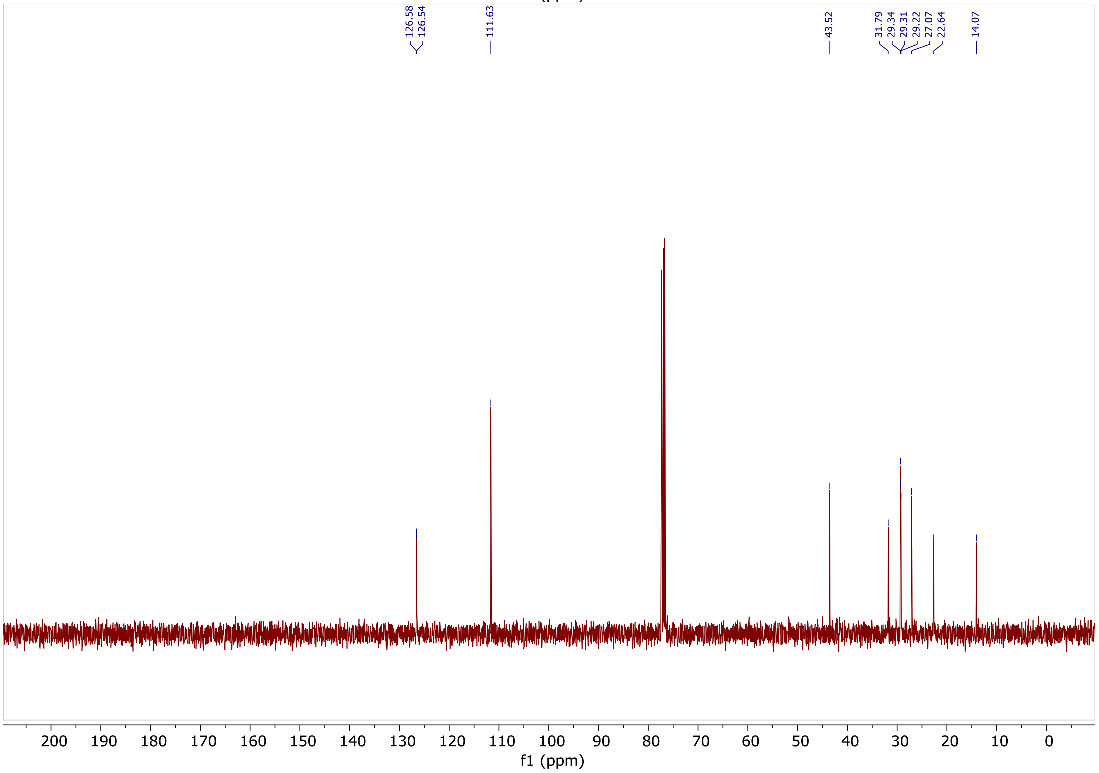

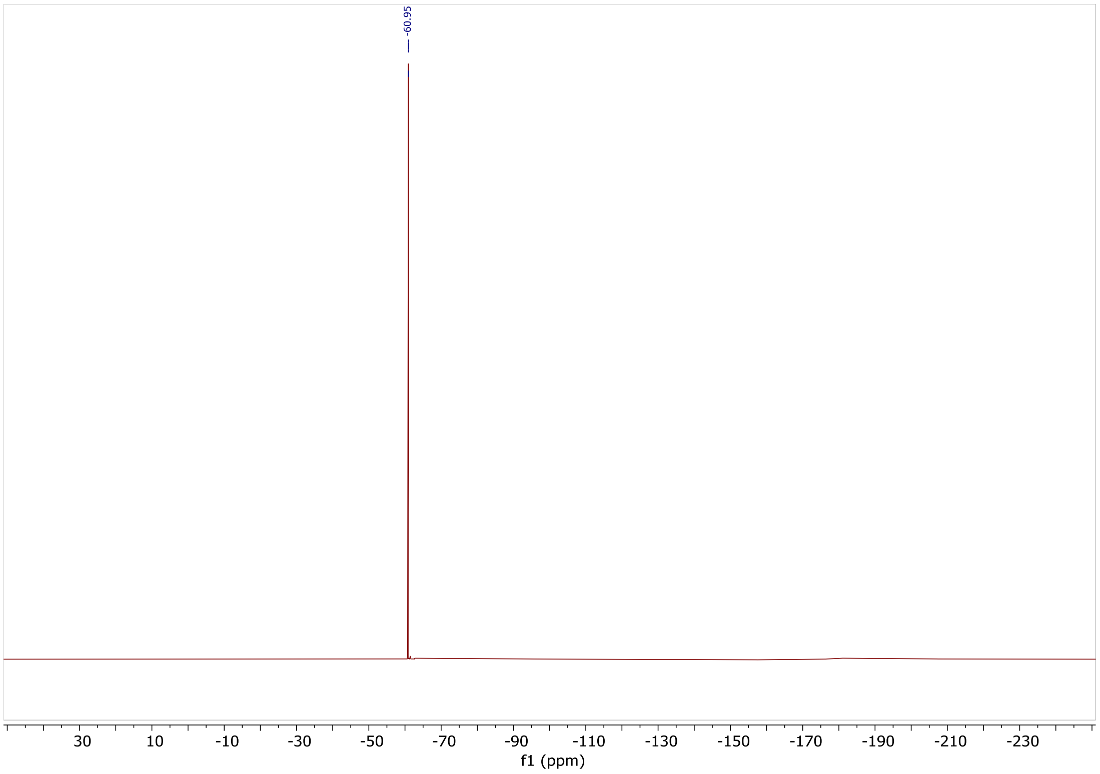

-60.95
30
10
-10
-30
-50
-70
-90
-110
-130
-150
-170
-190
-210
-230
f1 (ppm)

N-benzyl-N-methyl-4-(trifluoromethyl)aniline (1f)

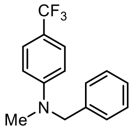

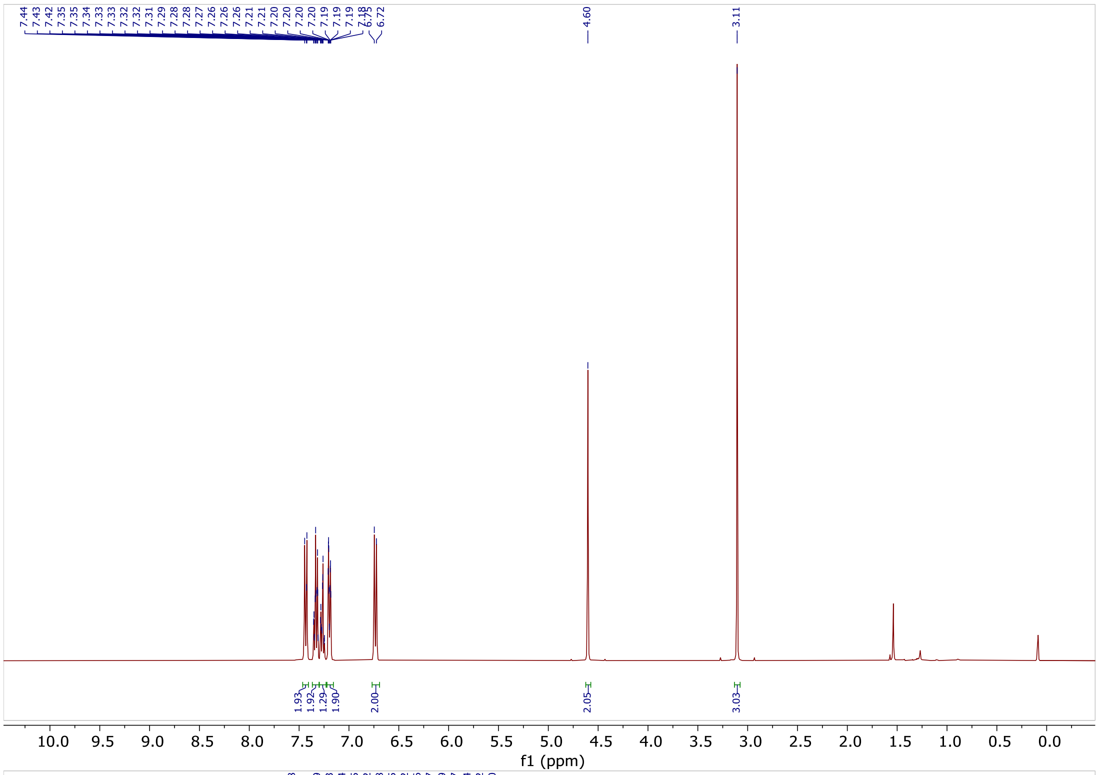

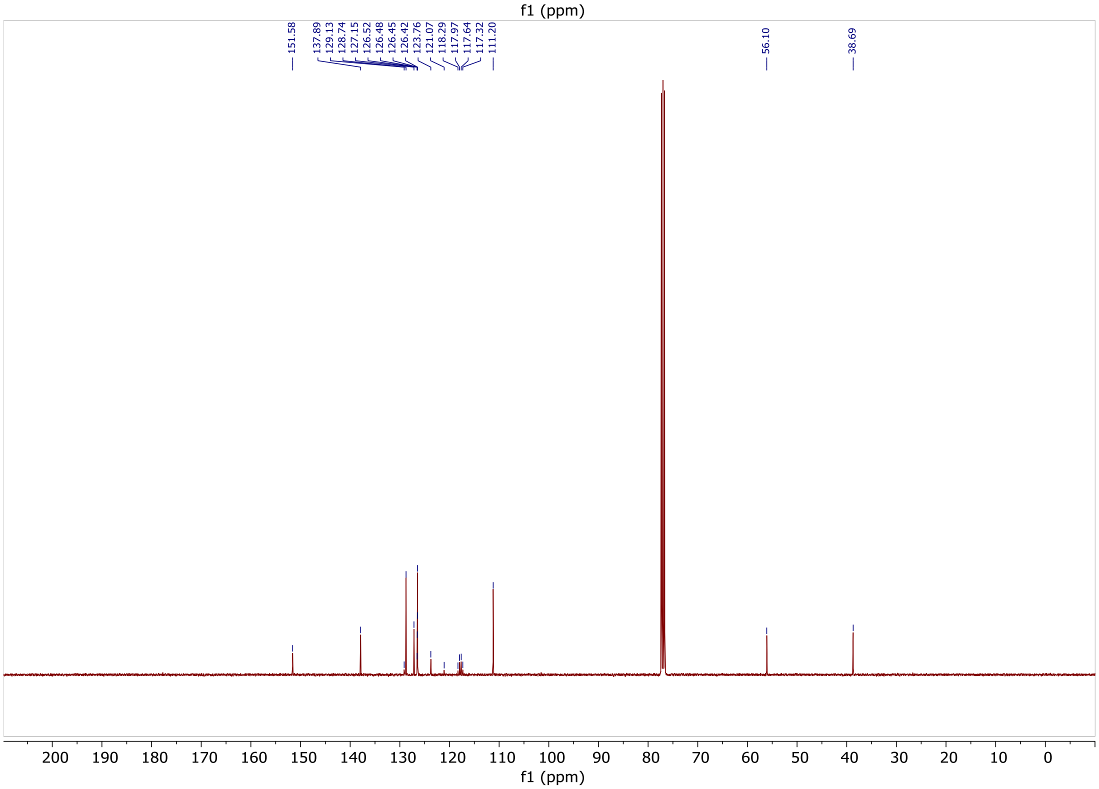

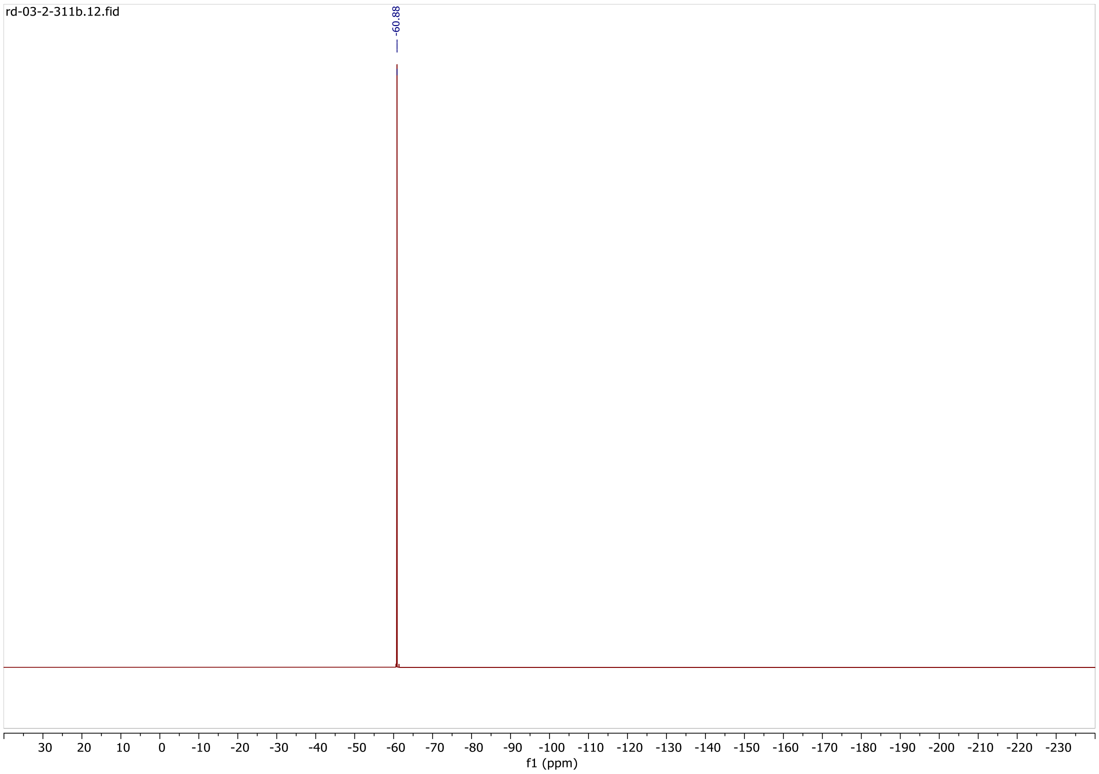
rd-03-2-311b.12.fid
-60.88
30
20
10
0
-10
-20
-30
-40
-50
-60
-70
-80
-90
-100
-110
-120
-130
-140
-150
-160
-170
-180
-190
-200
-210
-220
-230
f1 (ppm)

1-(4-(trifluoromethyl)phenyl)pyrrolidin-3-ol (1g)

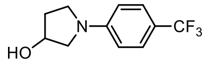

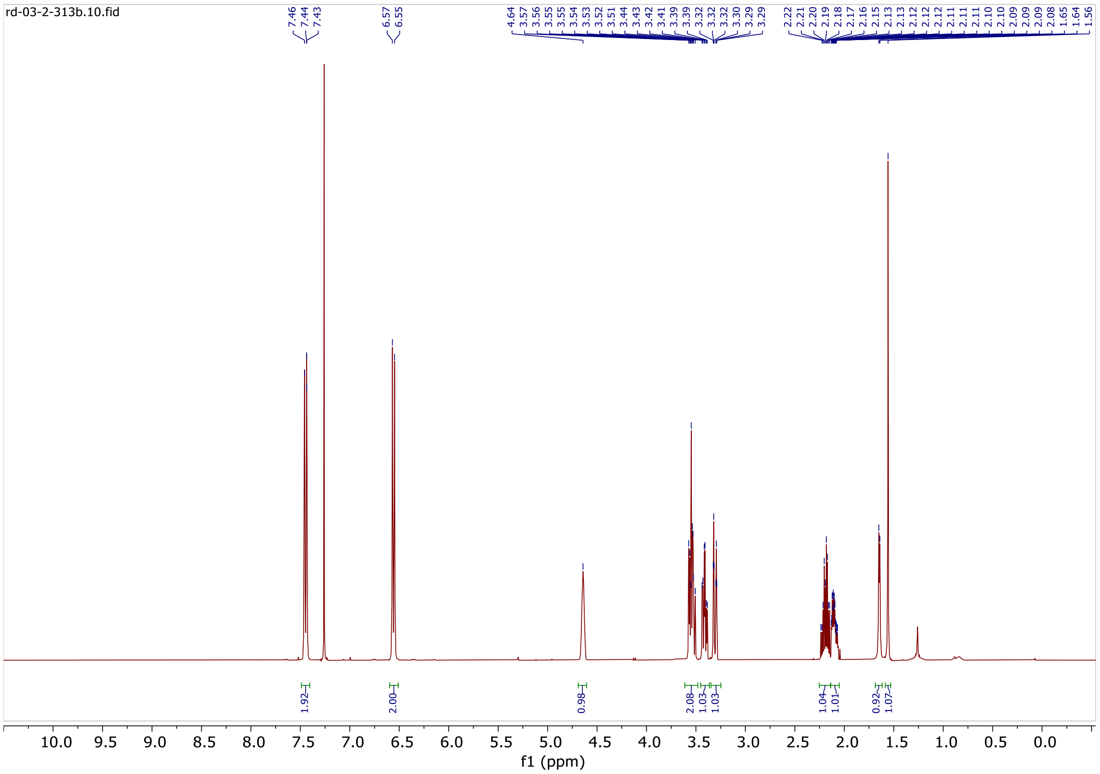

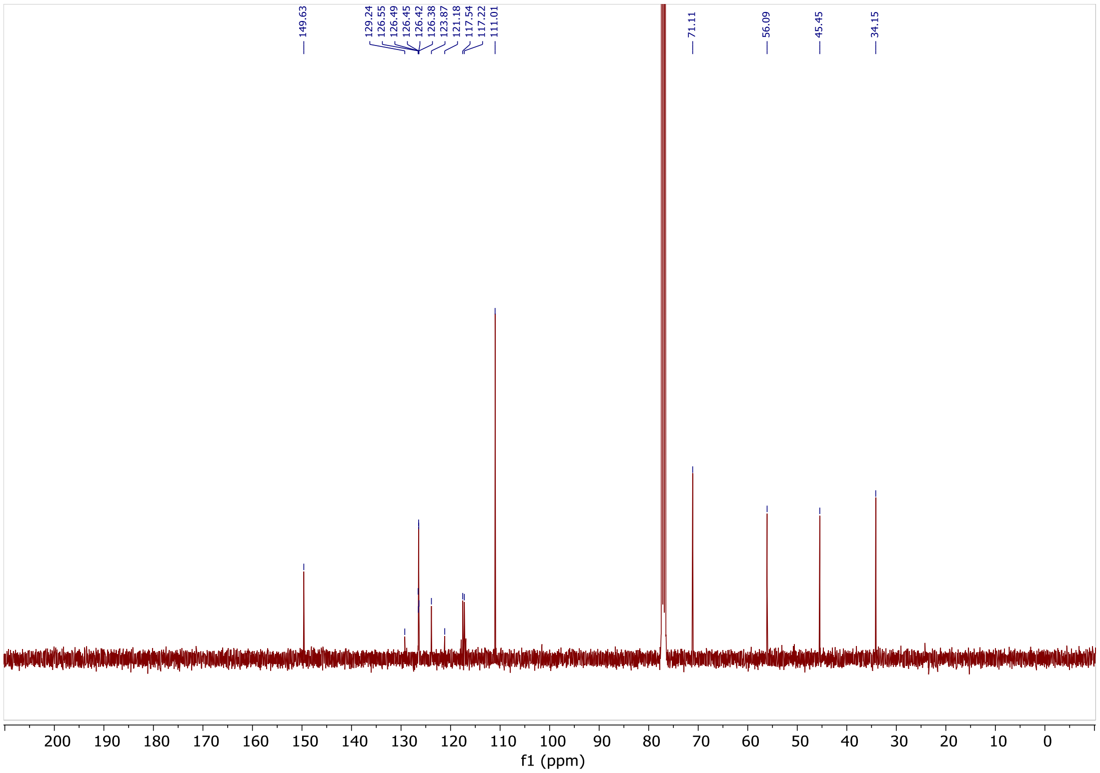

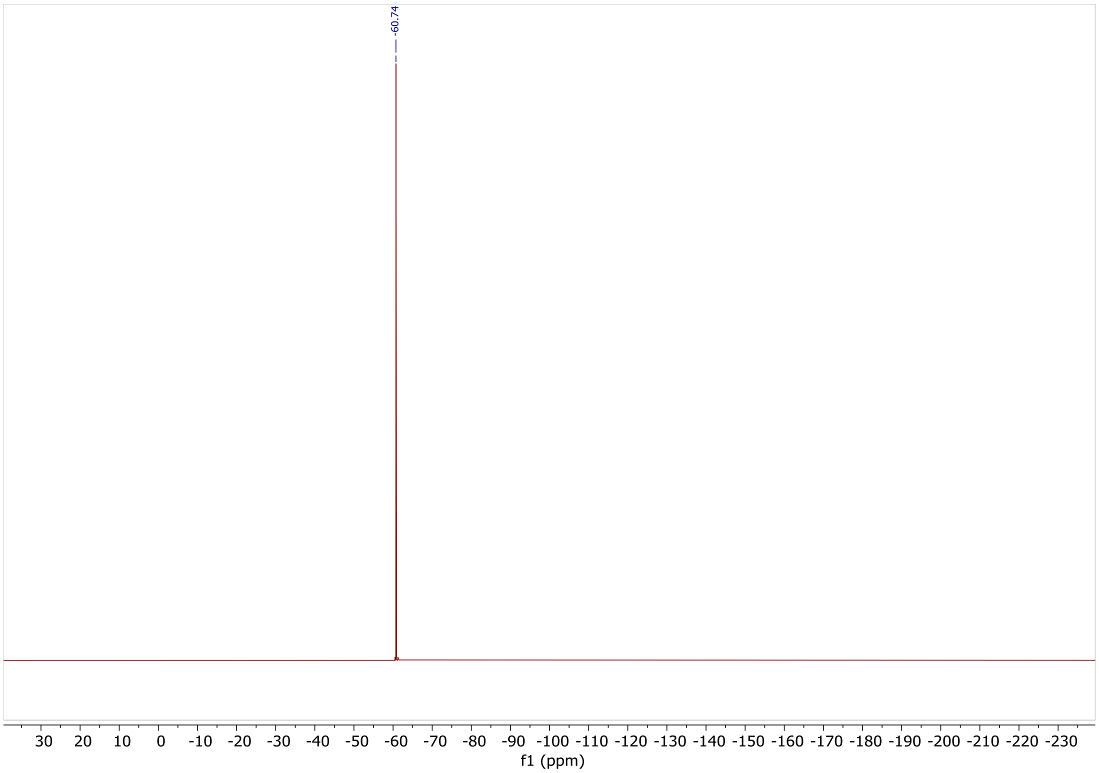
-60.74
30 20 10 0 -10 -20 -30 -40 -50 -60 -70 -80 -90 -100 -110 -120 -130 -140 -150 -160 -170 -180 -190 -200 -210 -220 -230
f1 (ppm)

2-(2-bromophenyl)-*N*-methylethan-1-amine

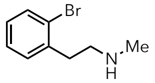

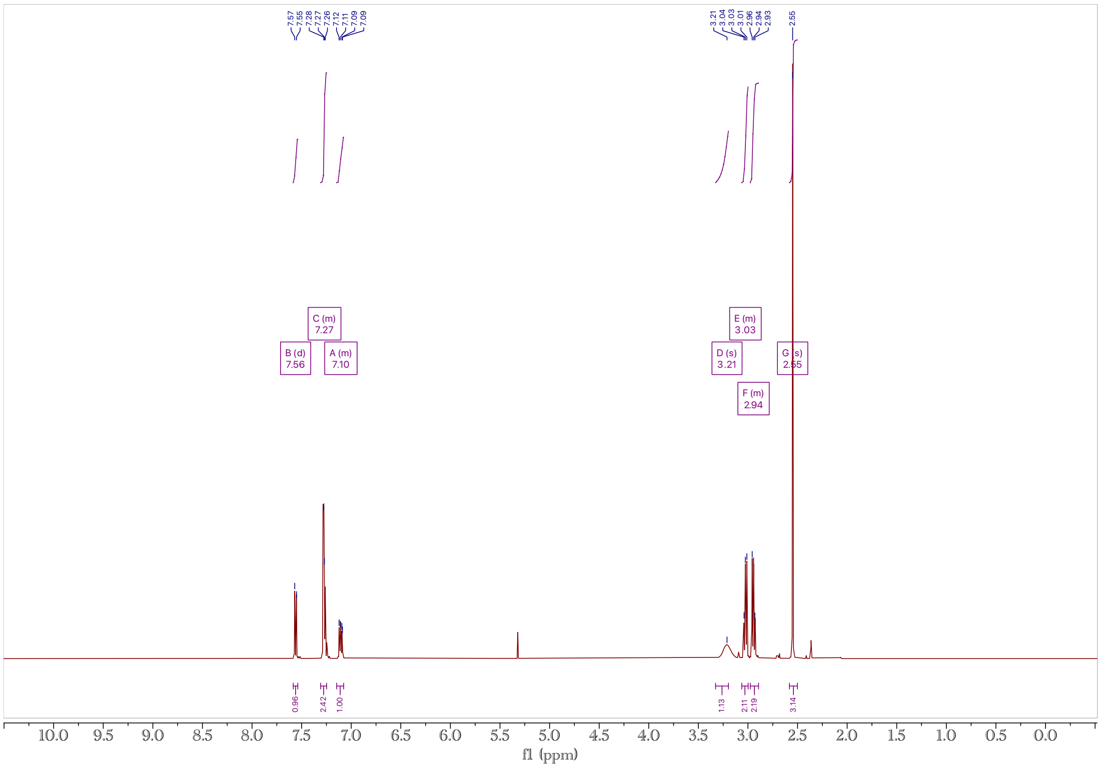

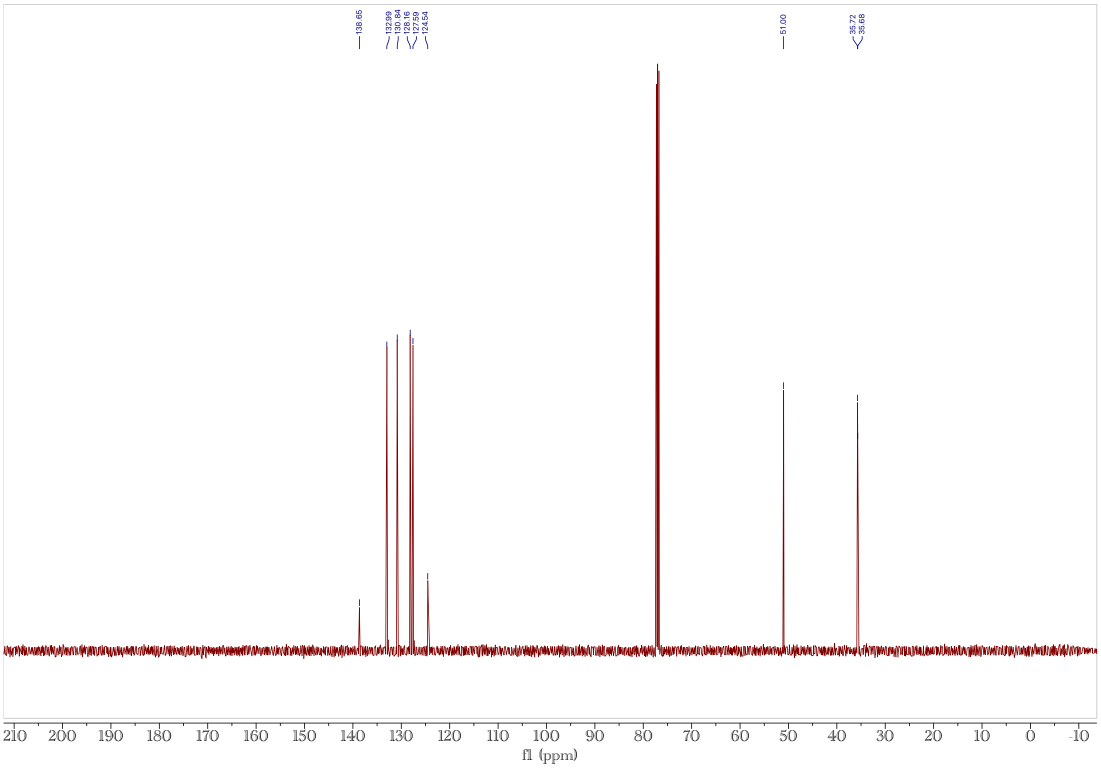

2-(2-chlorophenyl)-*N*-methylethan-1-amine

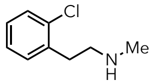

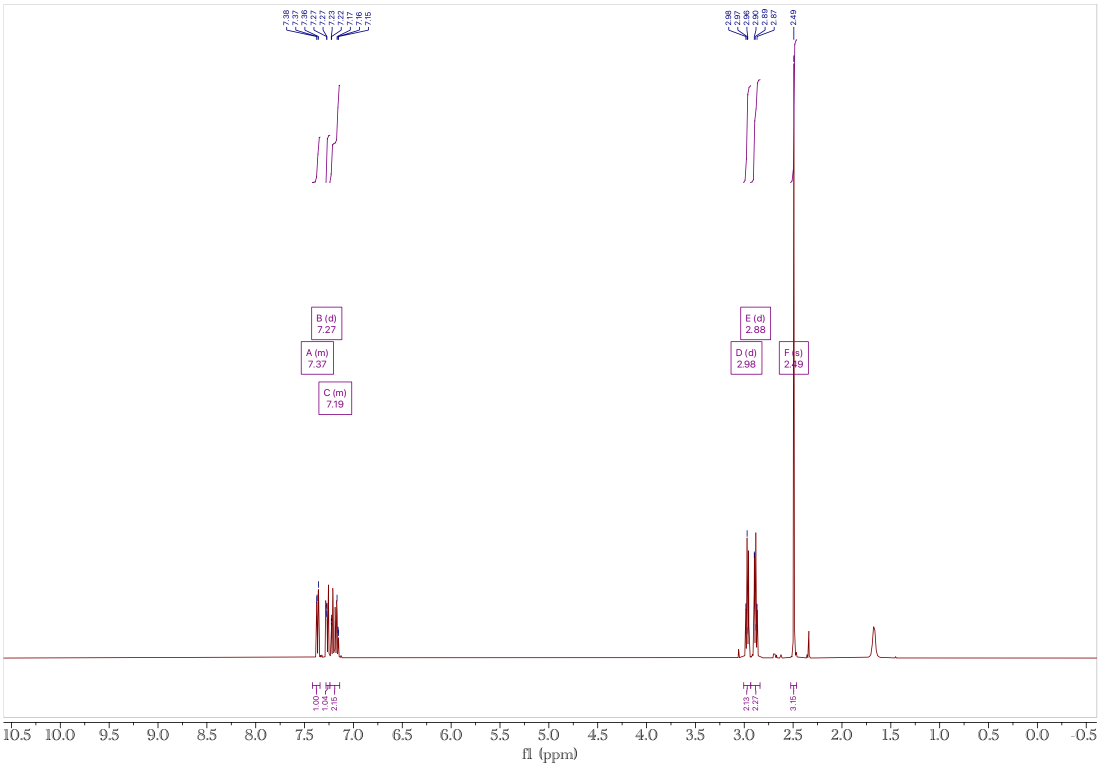

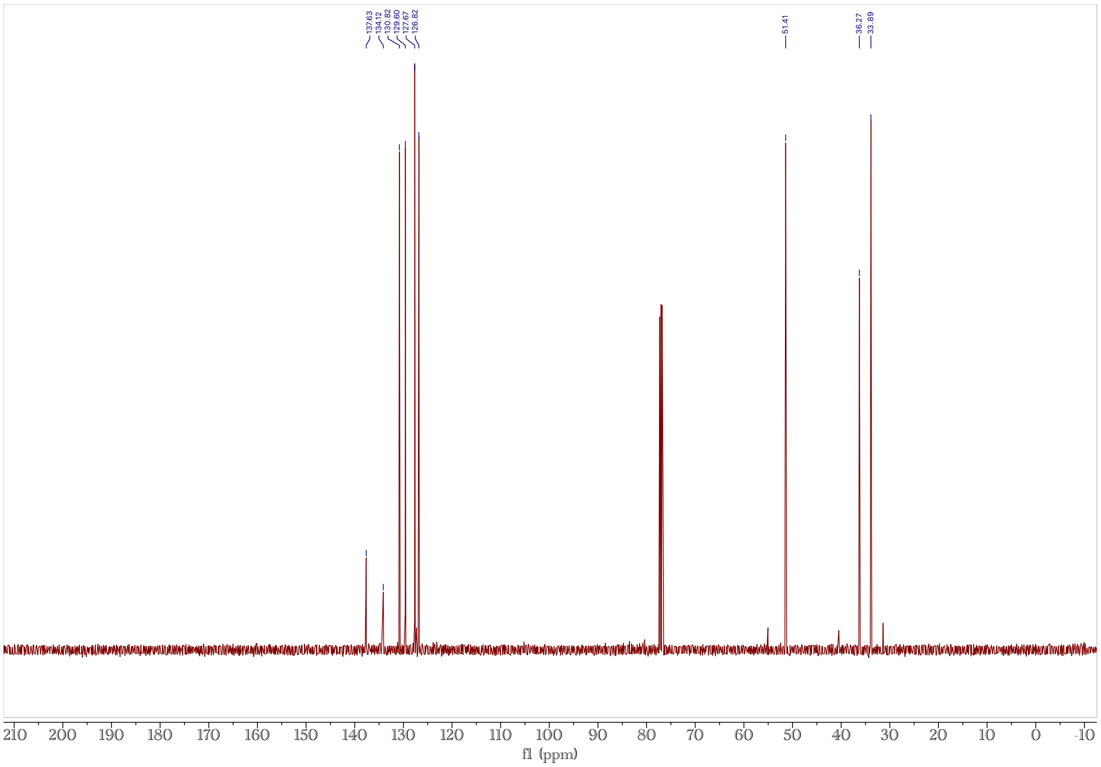

2-(2-fluorophenyl)-*N*-methylethan-1-amine

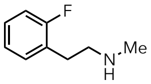

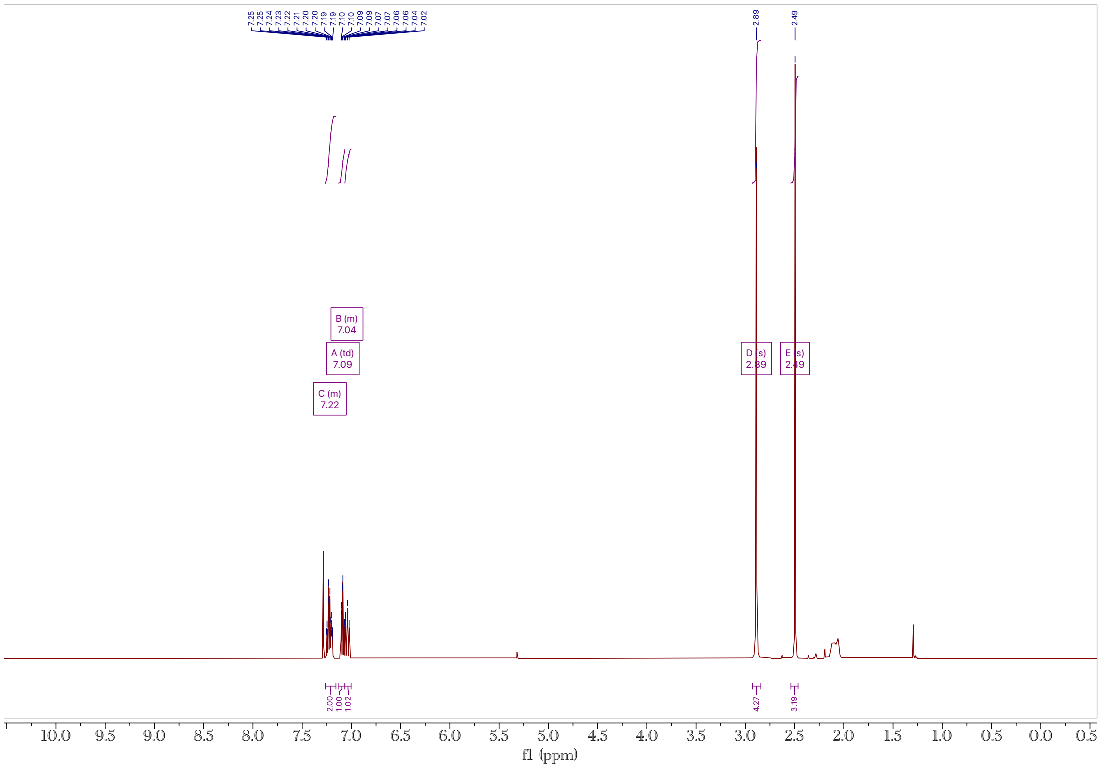

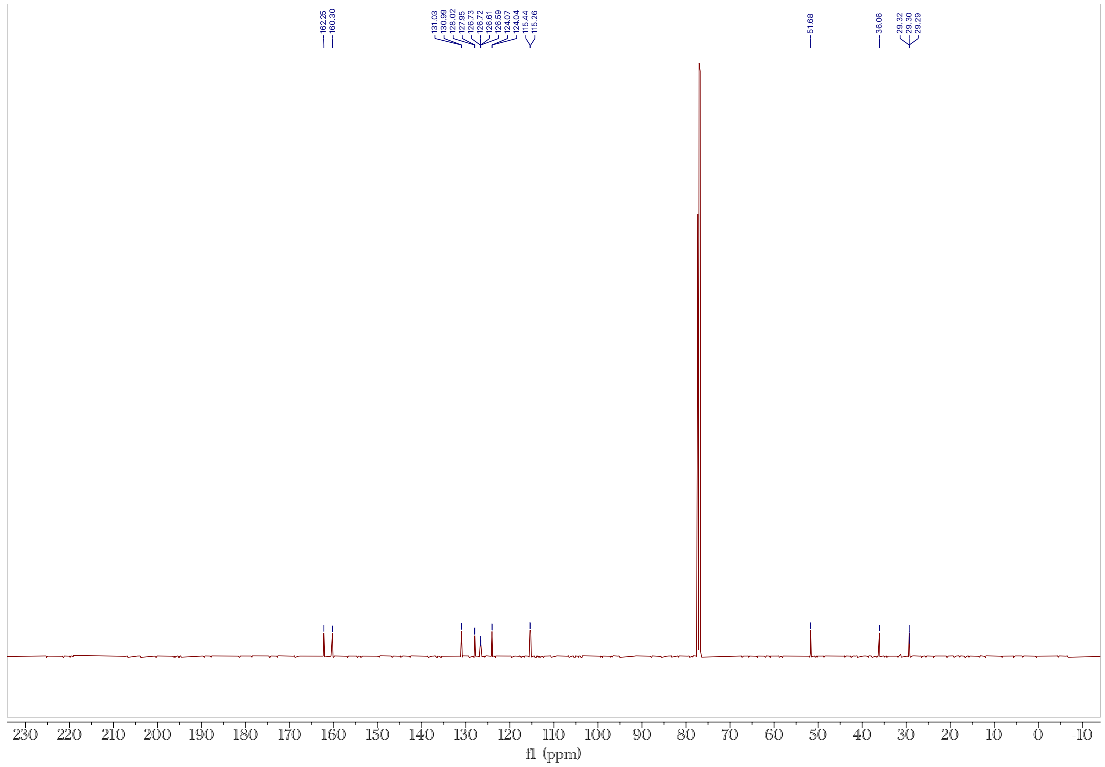
162.25
160.30
131.03
130.99
128.02
127.95
126.73
126.72
126.61
126.59
124.07
124.04
115.44
115.26
51.68
36.06
29.32
29.30
29.29
f1 (ppm)

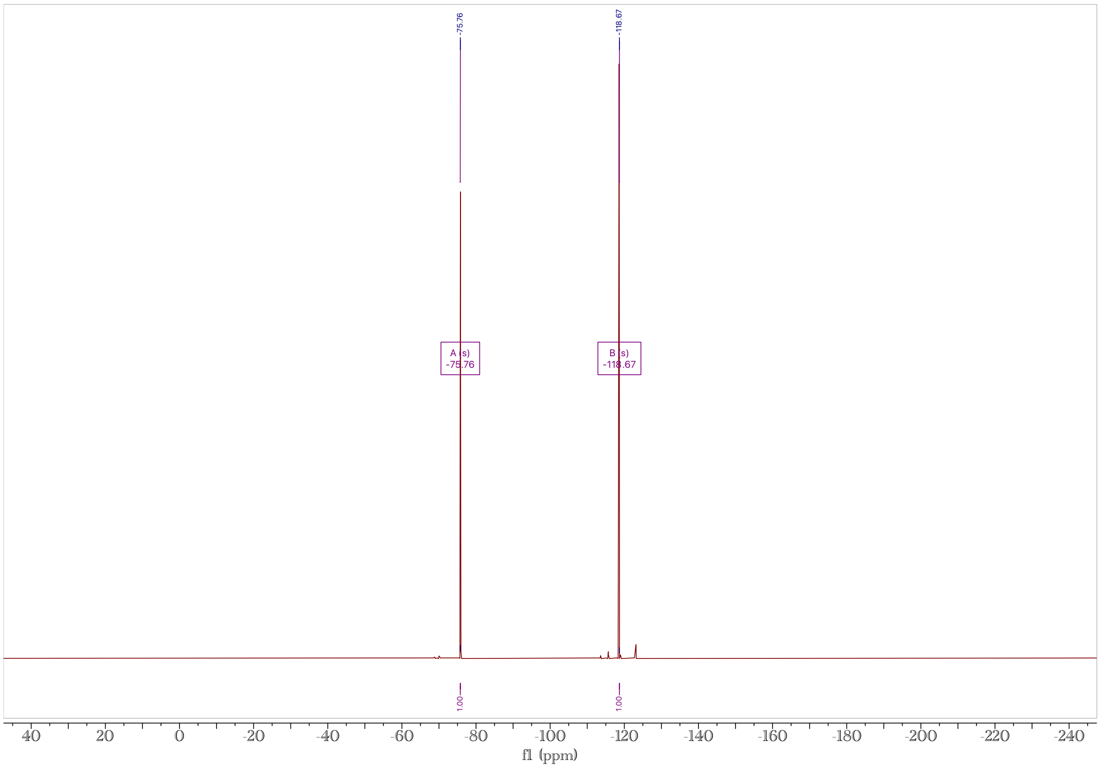
-75.76
-118.67
A (s)
-75.76
B (s)
-118.67
1.00
1.00
f1 (ppm)

2-(2-iodophenyl)-*N*-methylethan-1-amine

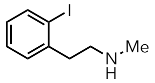

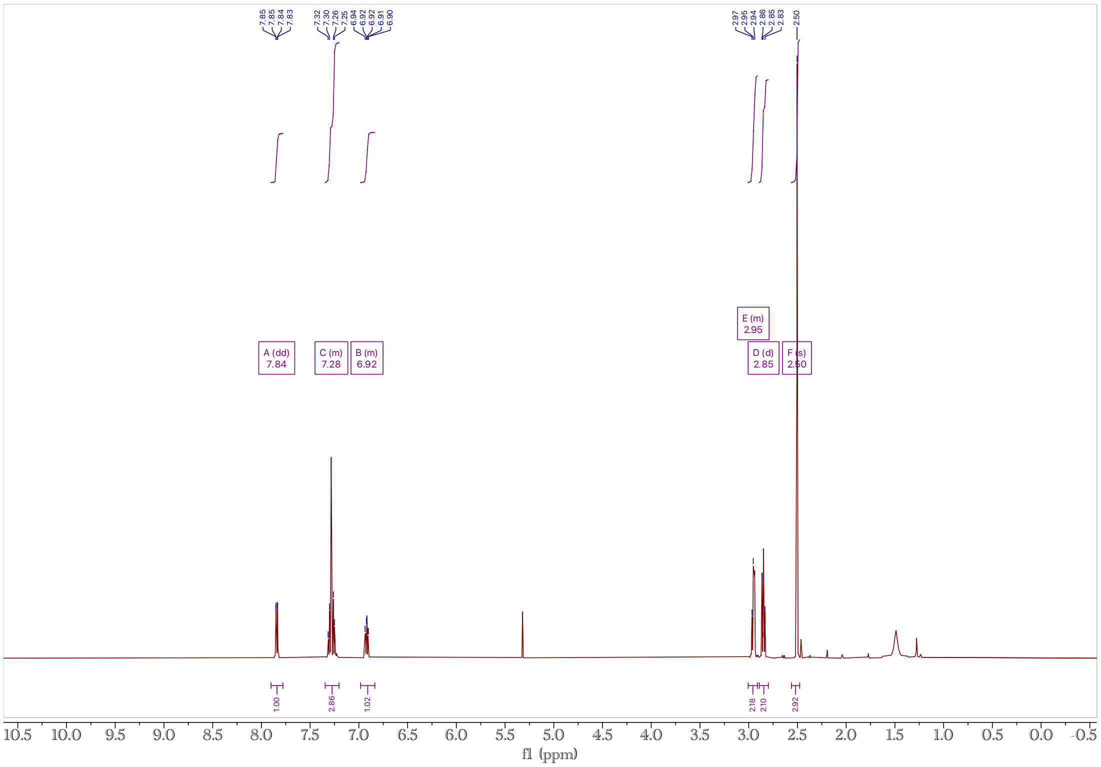

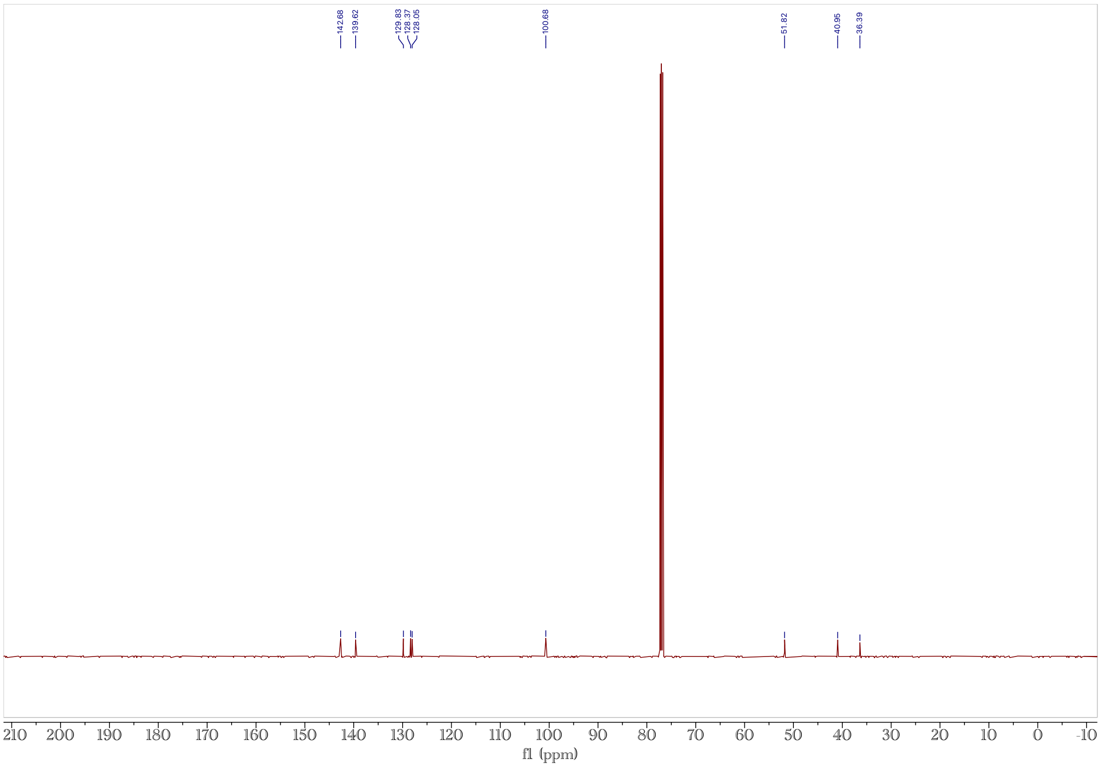

2-(2-bromo-5-chlorophenyl)-*N*-methylethan-1-amine

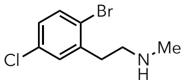

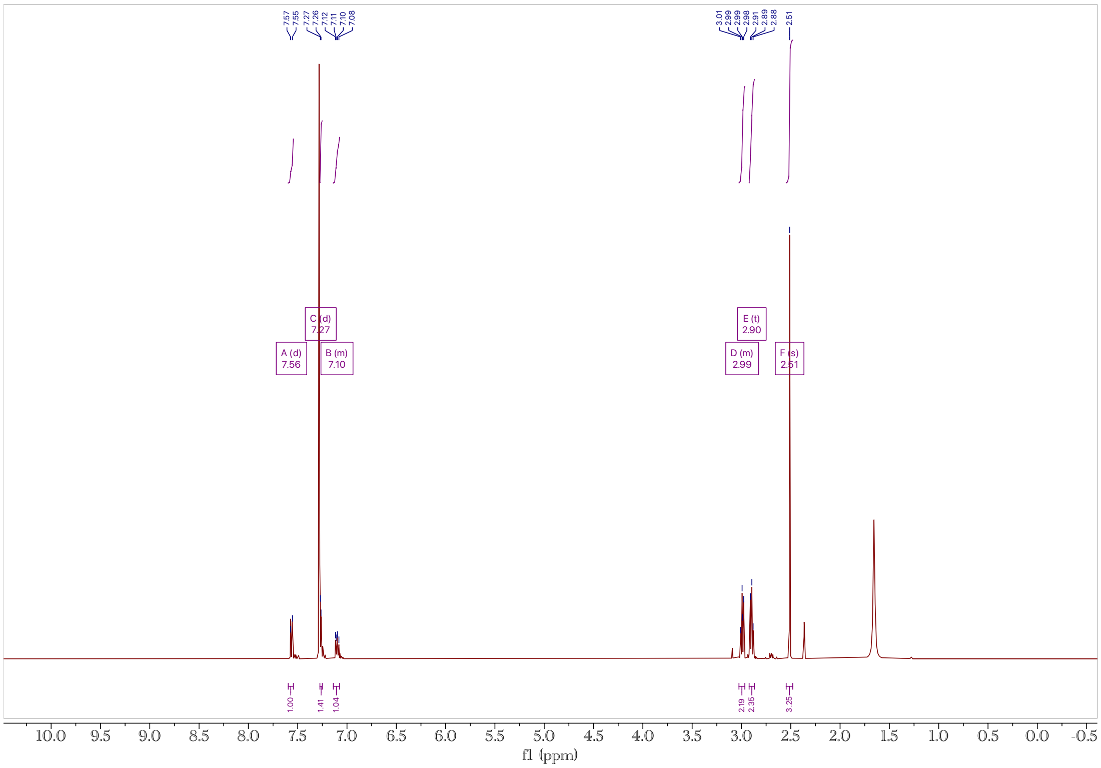

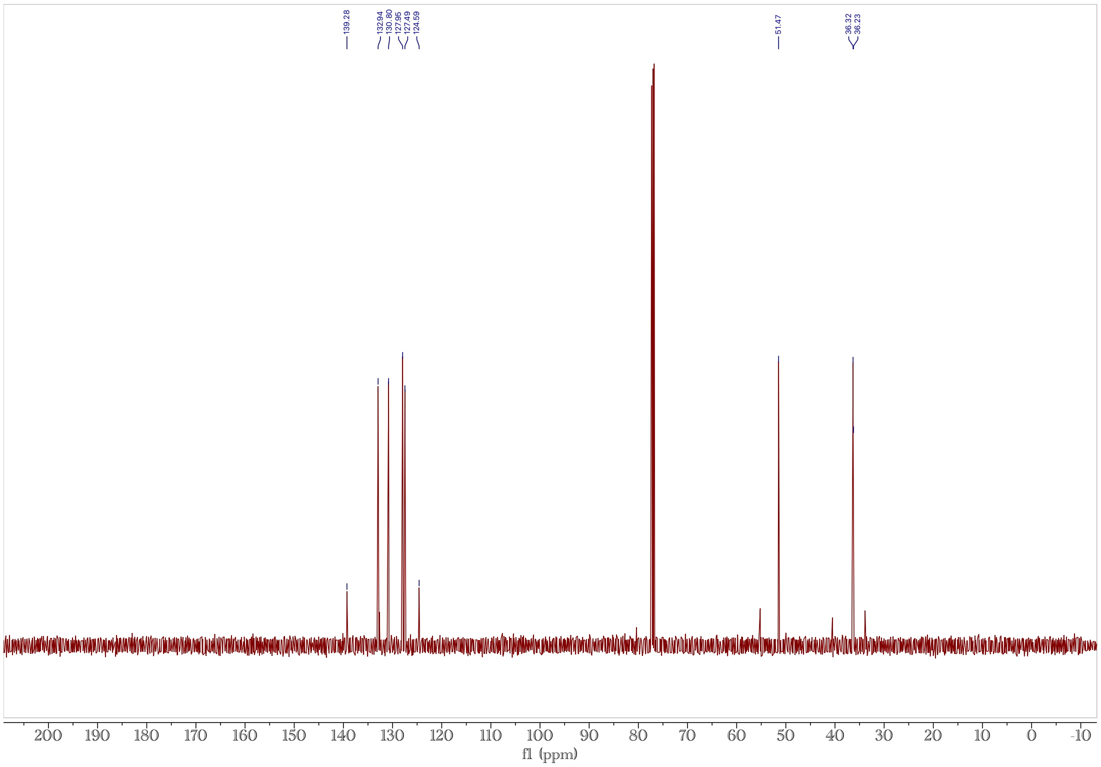

2-(2-bromo-5-fluorophenyl)-*N*-methylethan-1-amine

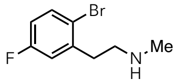

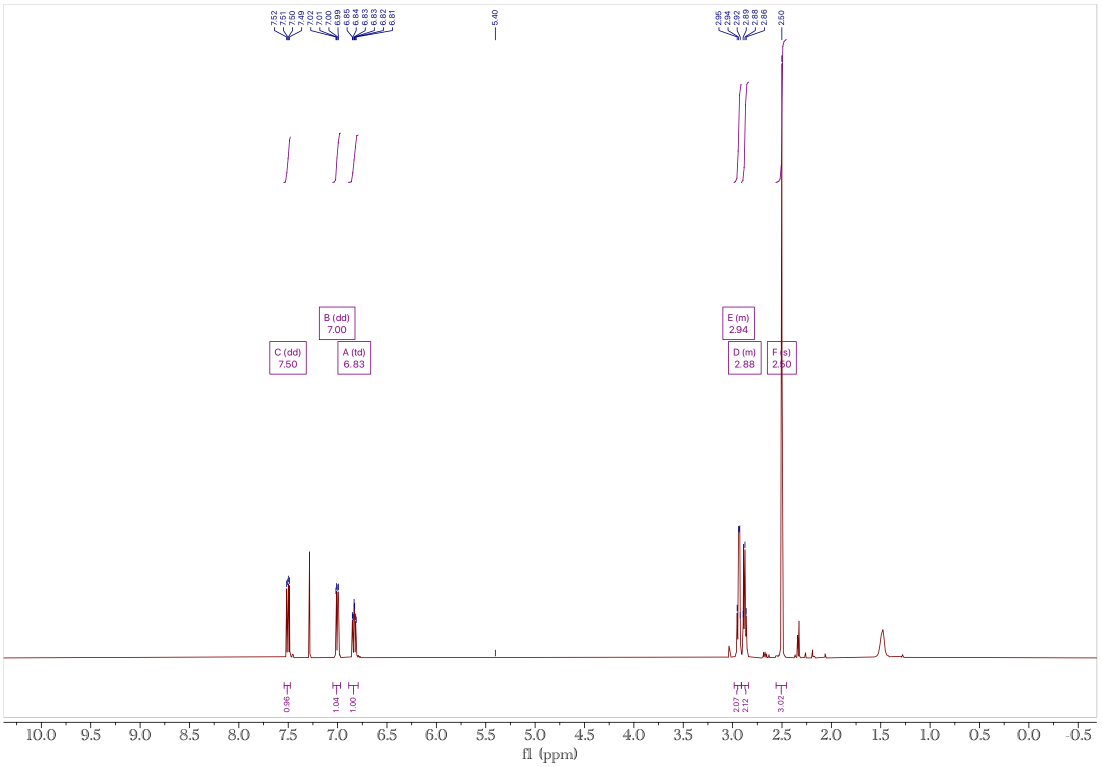
7.52
7.51
7.50
7.49
7.02
7.01
7.00
6.99
6.85
6.84
6.83
6.83
6.82
6.81
5.40
2.95
2.94
2.92
2.89
2.88
2.86
2.50
C (dd)
7.50
B (dd)
7.00
A (td)
6.83
E (m)
2.94
D (m)
2.88
F (s)
2.50
0.96
1.04
1.00
2.07
2.12
3.02
f1 (ppm)

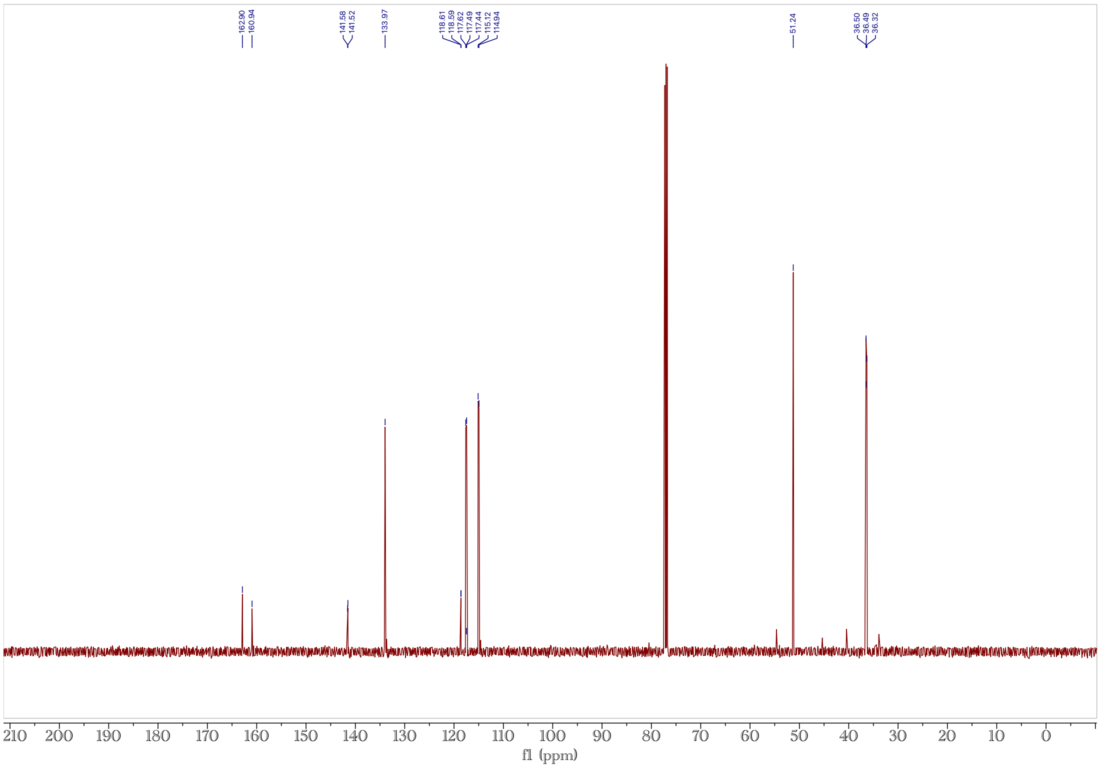
162.90
160.94
141.58
141.52
133.97
118.61
118.59
117.62
117.49
117.44
115.12
114.94
51.24
36.50
36.49
36.32
f1 (ppm)

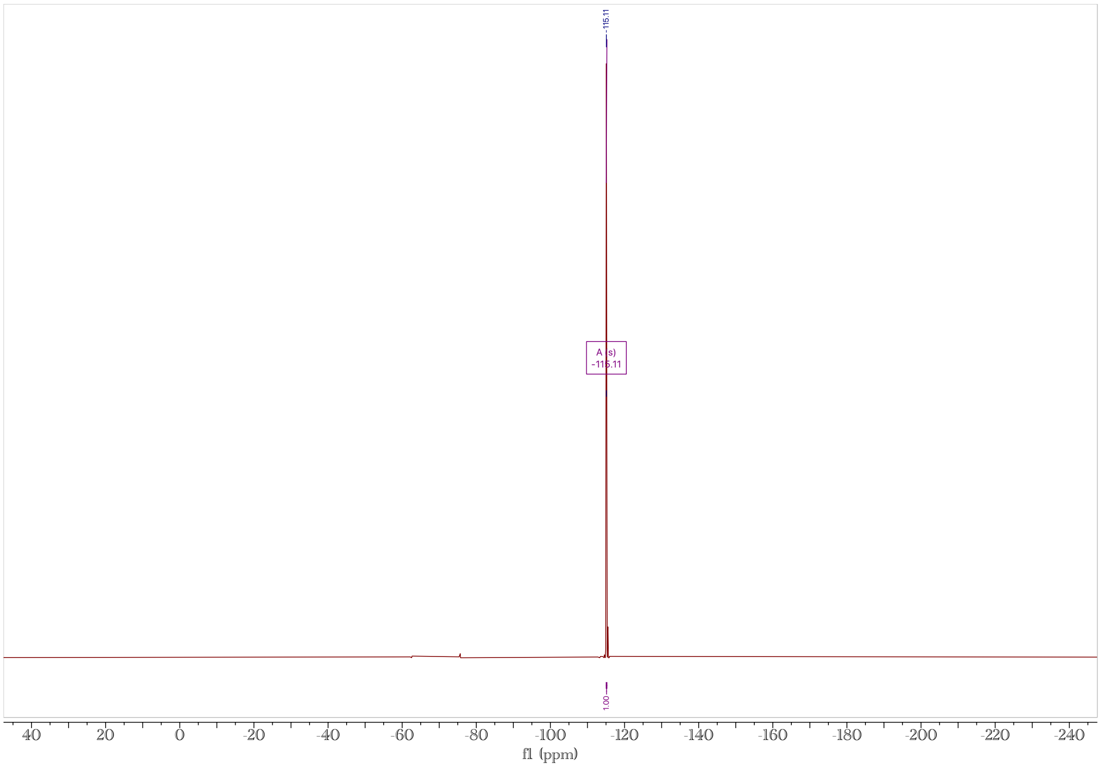
-115.11
A (s)
-115.11
1.00
40 20 0 -20 -40 -60 -80 -100 -120 -140 -160 -180 -200 -220 -240
f1 (ppm)

2-(2-bromo-5-(trifluoromethyl)phenyl)-*N*-methylethan-1-amine

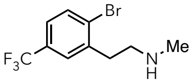

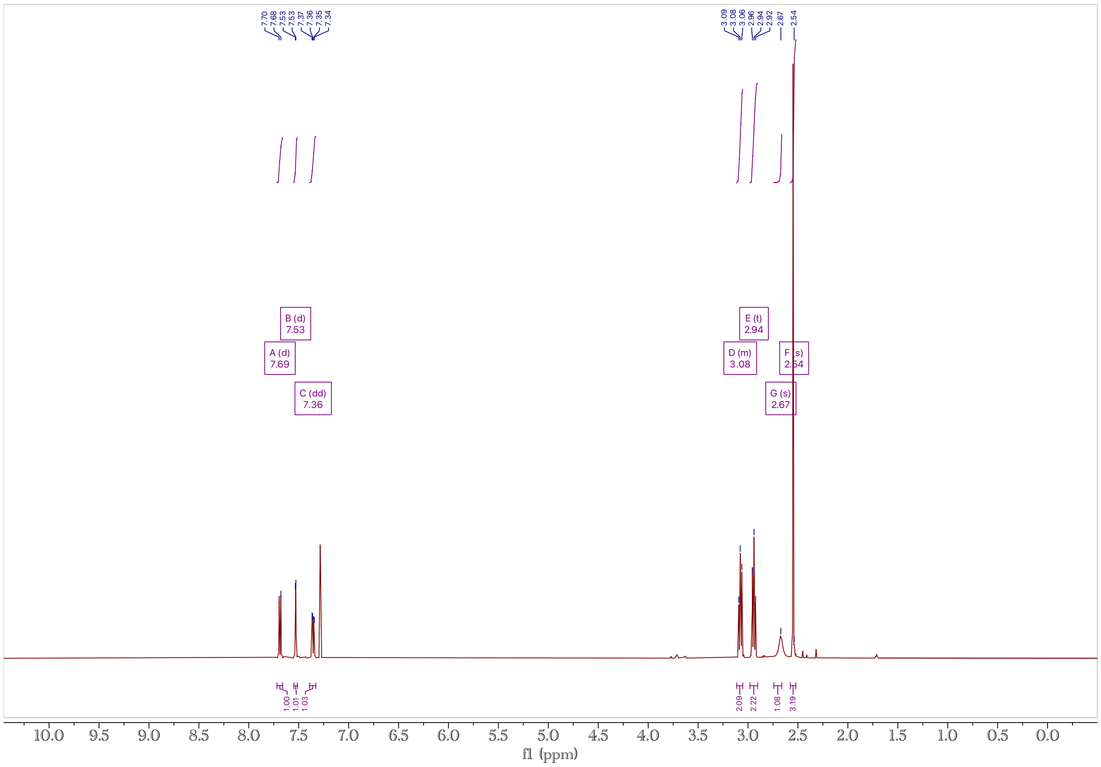

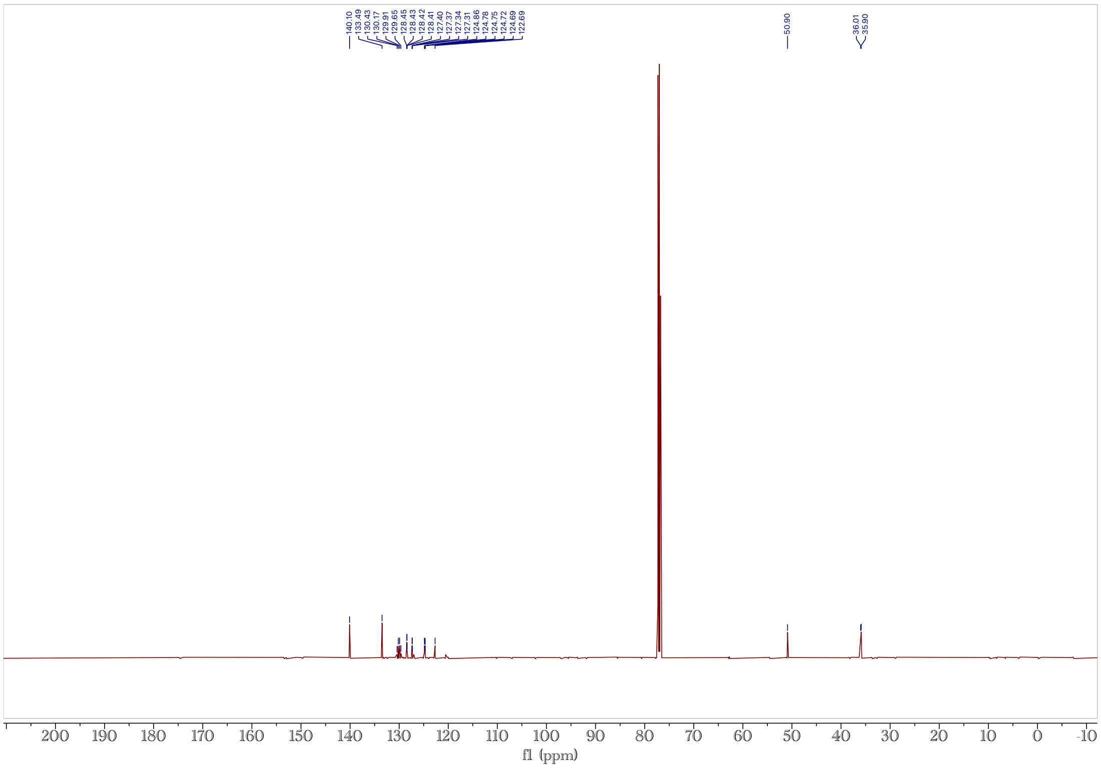

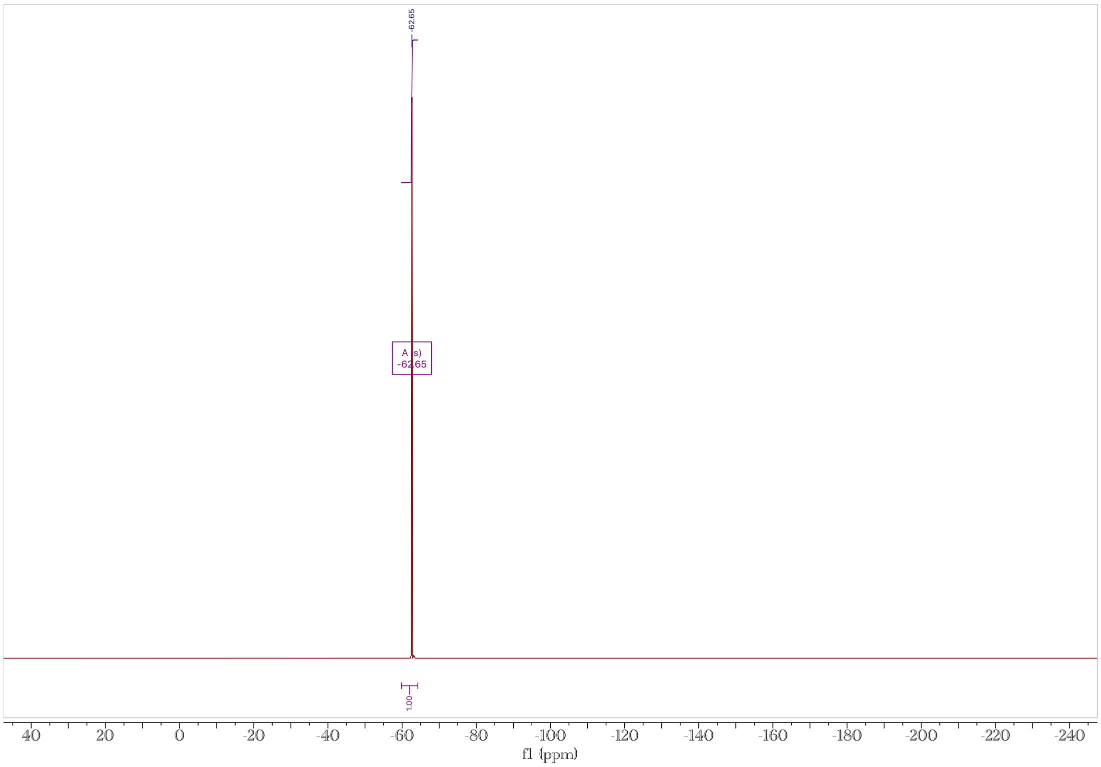
-62.65
A (s)
-62.65
1.00
40
20
0
-20
-40
-60
-80
-100
-120
-140
-160
-180
-200
-220
-240
f1 (ppm)

3-(2-bromophenyl)-*N*-methylpropan-1-amine

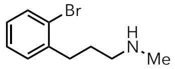

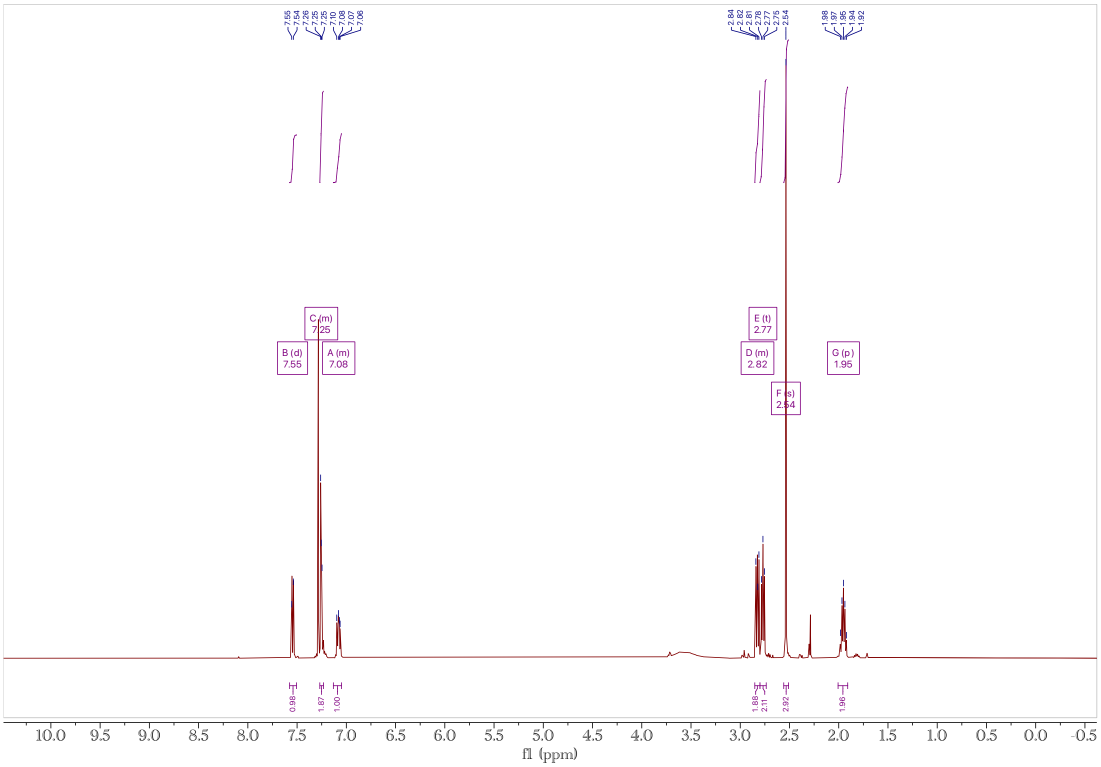

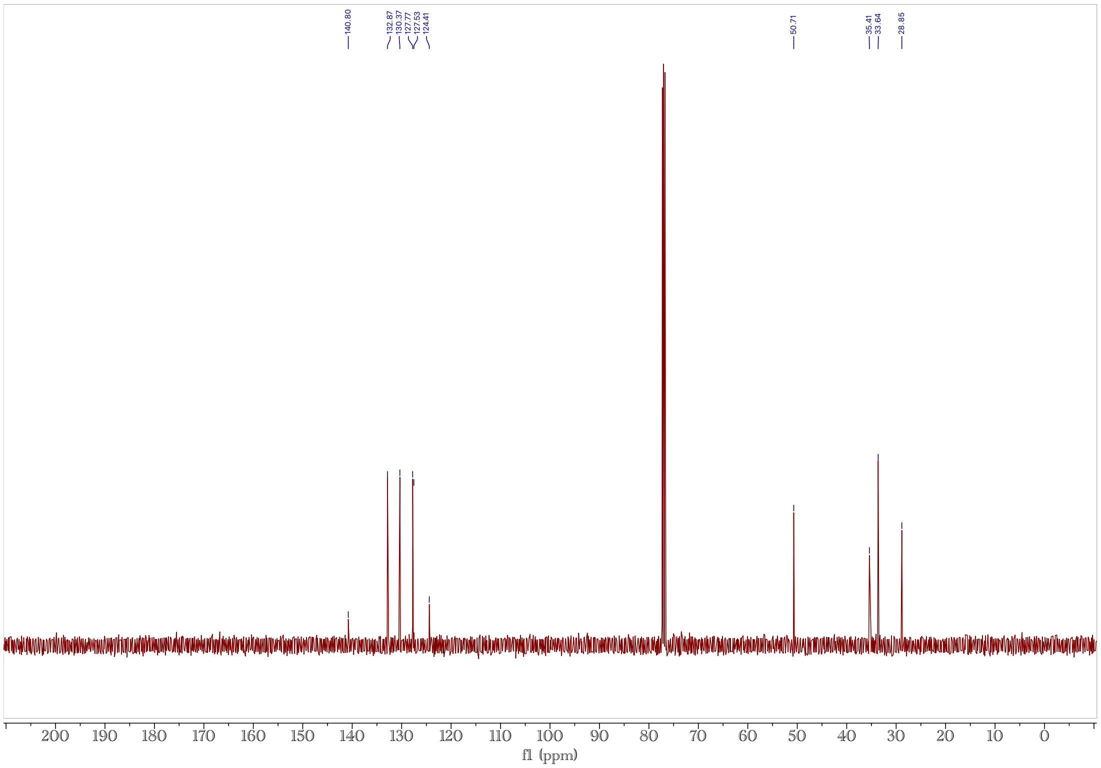

1-methylindoline (14)

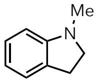

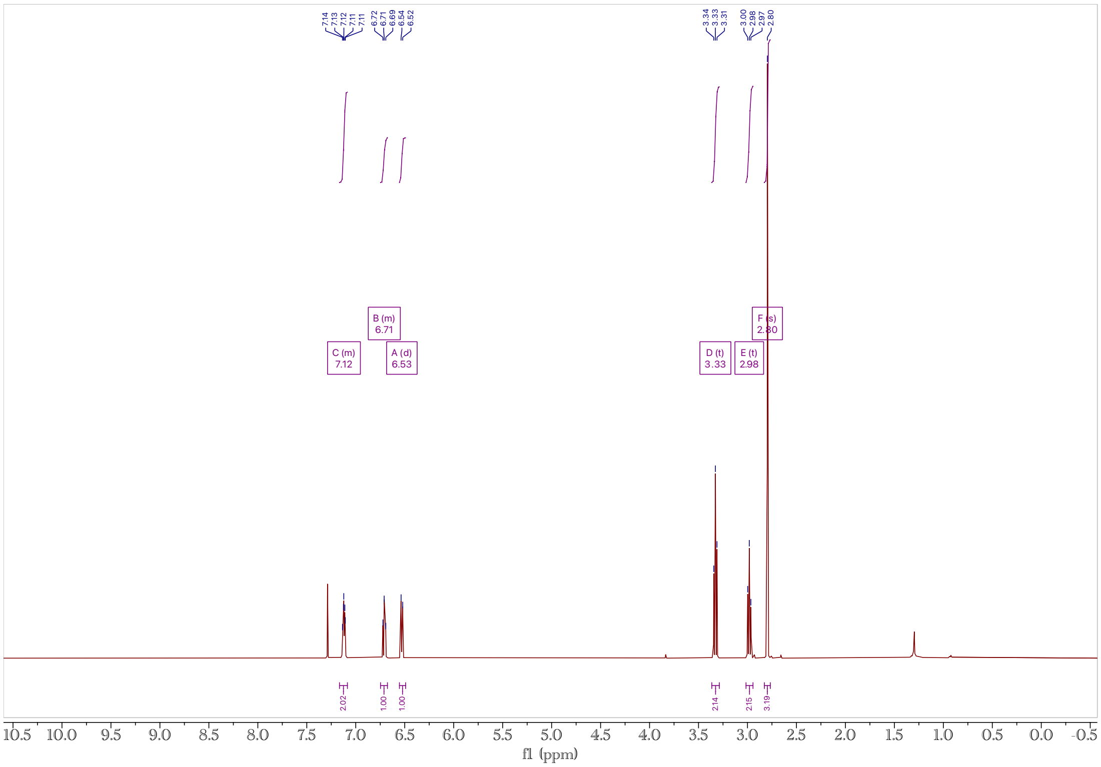

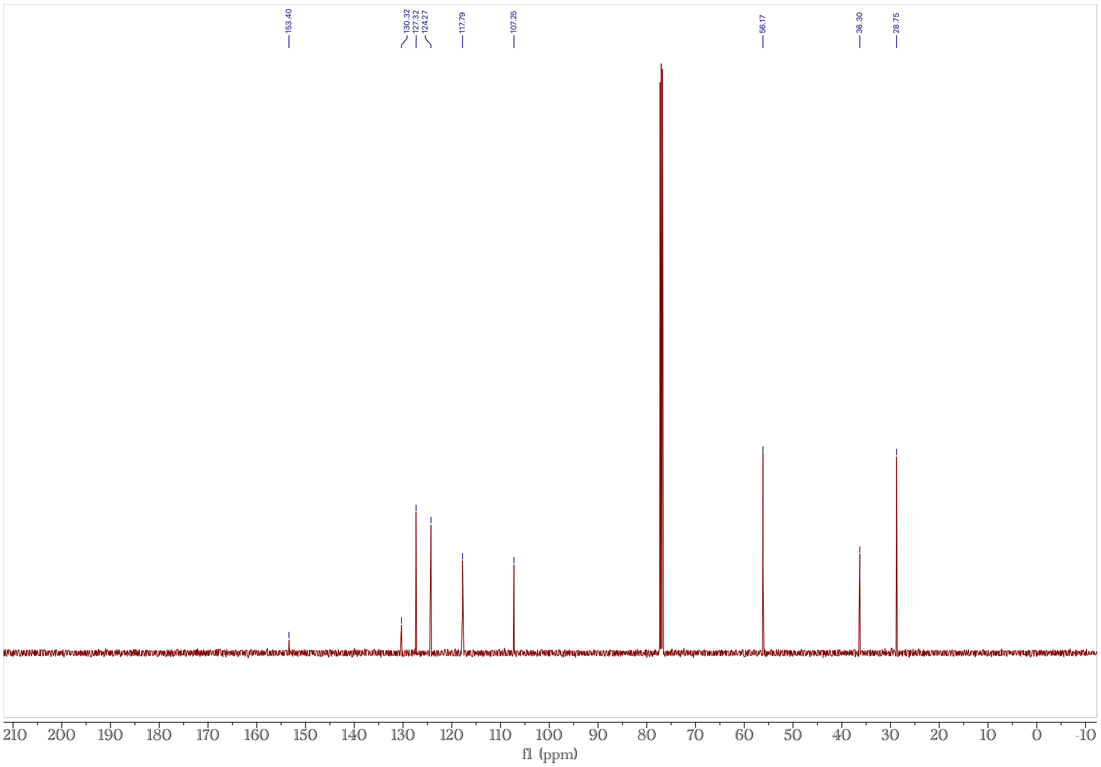
153.40
130.32
127.32
124.27
117.79
107.25
56.17
36.30
28.75
f1 (ppm)

Indoline (15)

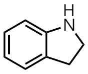

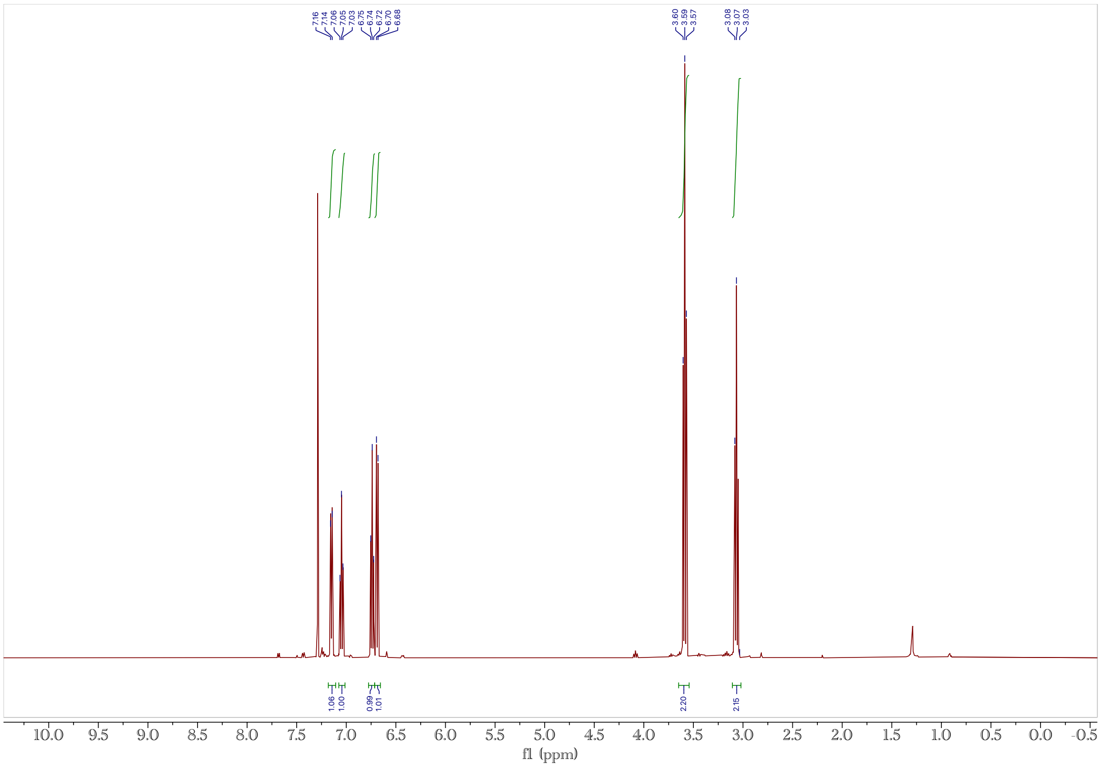
7.16
7.14
7.06
7.05
7.03
6.75
6.74
6.72
6.70
6.68
3.60
3.59
3.57
3.08
3.07
3.03
1.06
1.00
0.99
1.01
2.20
2.15
f1 (ppm)

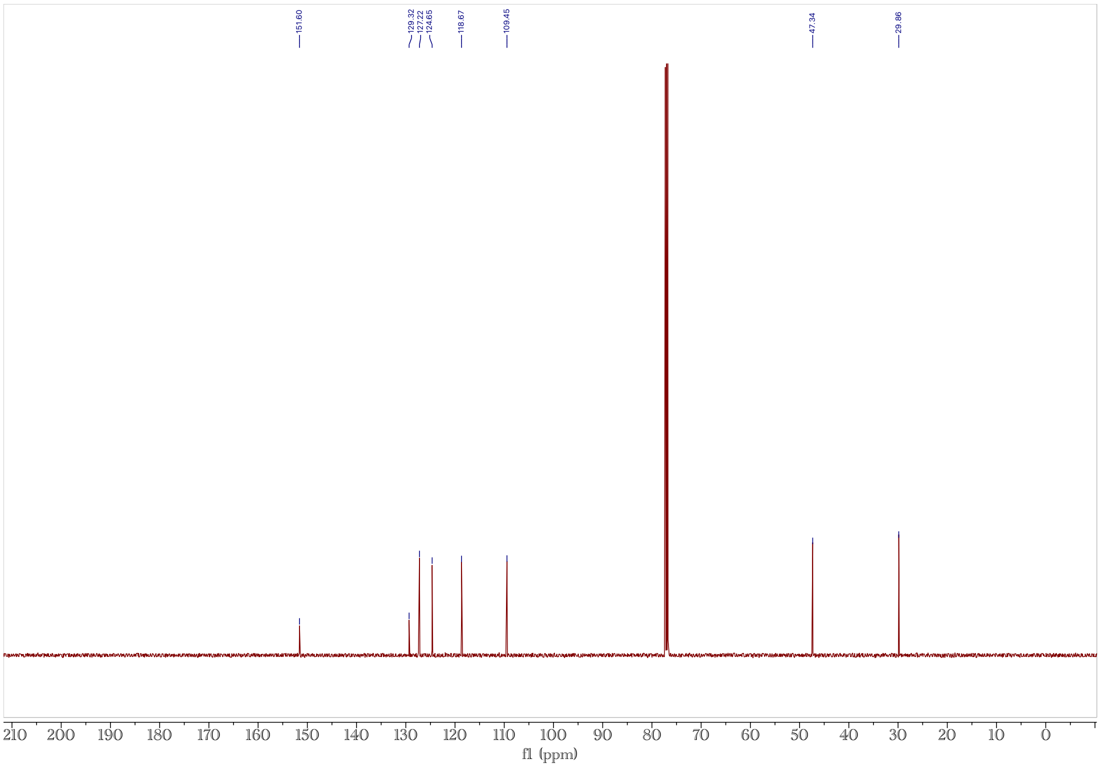
151.60
129.32
127.22
124.65
118.67
109.45
47.34
29.86
210
200
190
180
170
160
150
140
130
120
110
100
90
80
70
60
50
40
30
20
10
0
f1 (ppm)

5-chloro-1-methylindoline (16)

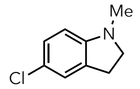

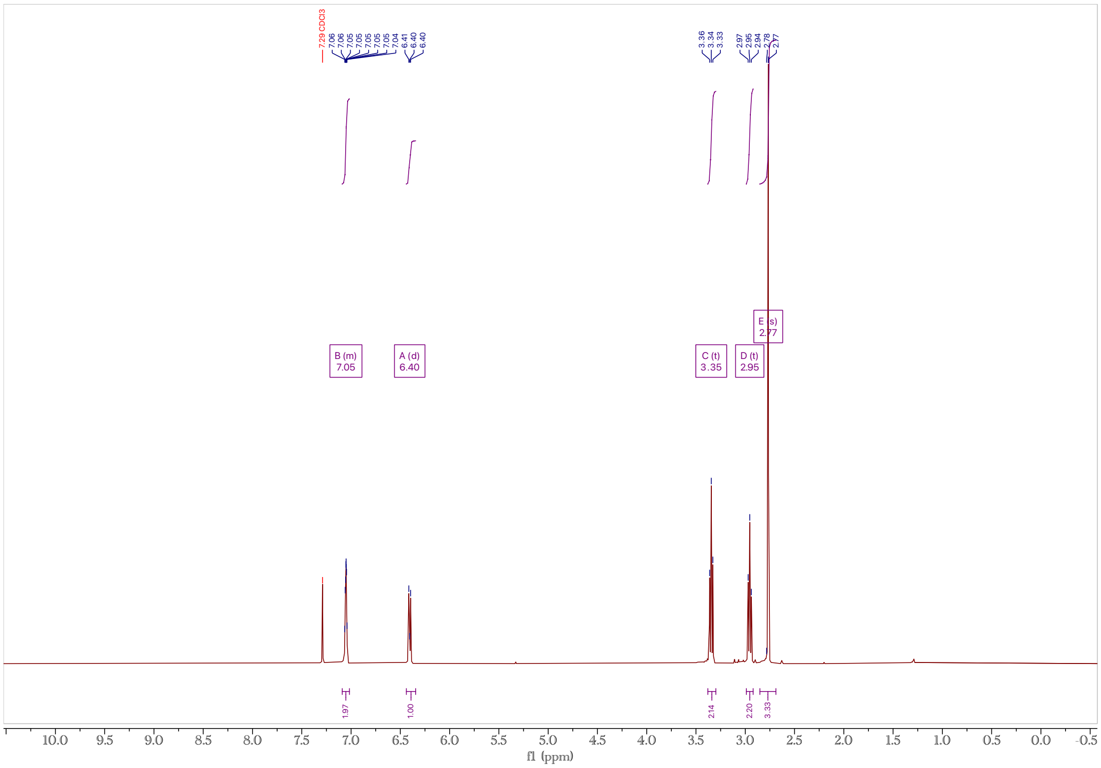

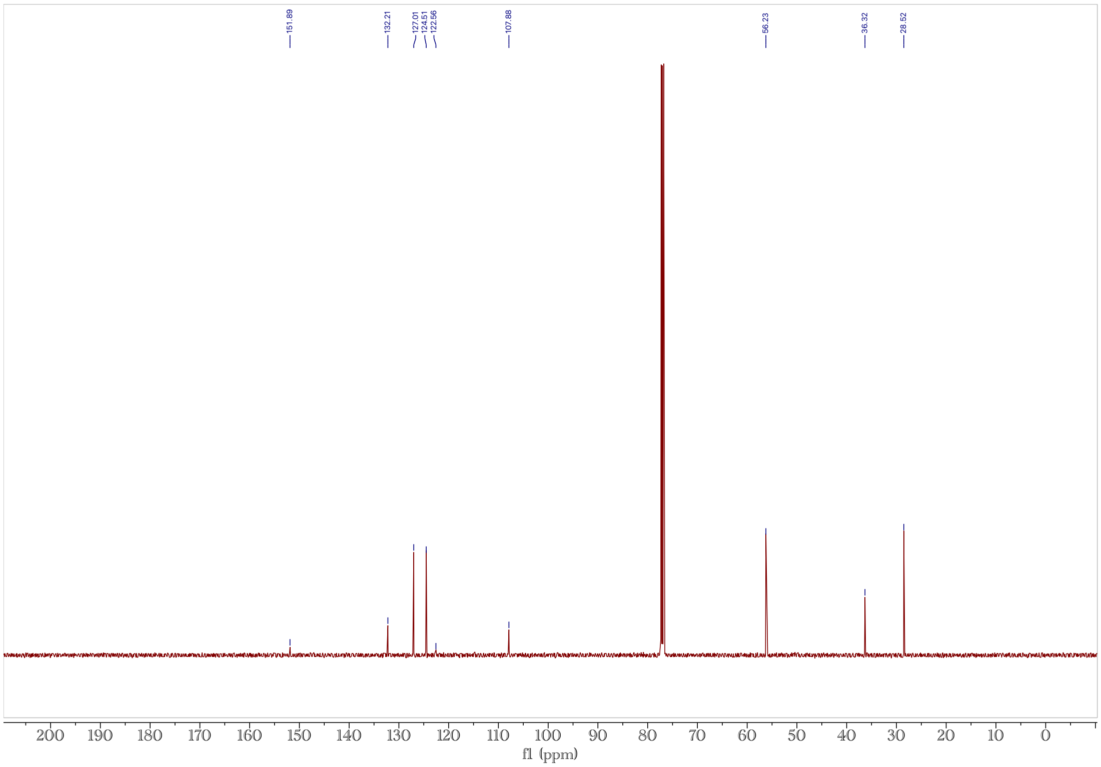

5-fluoro-1-methylindoline (17)

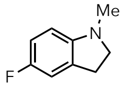

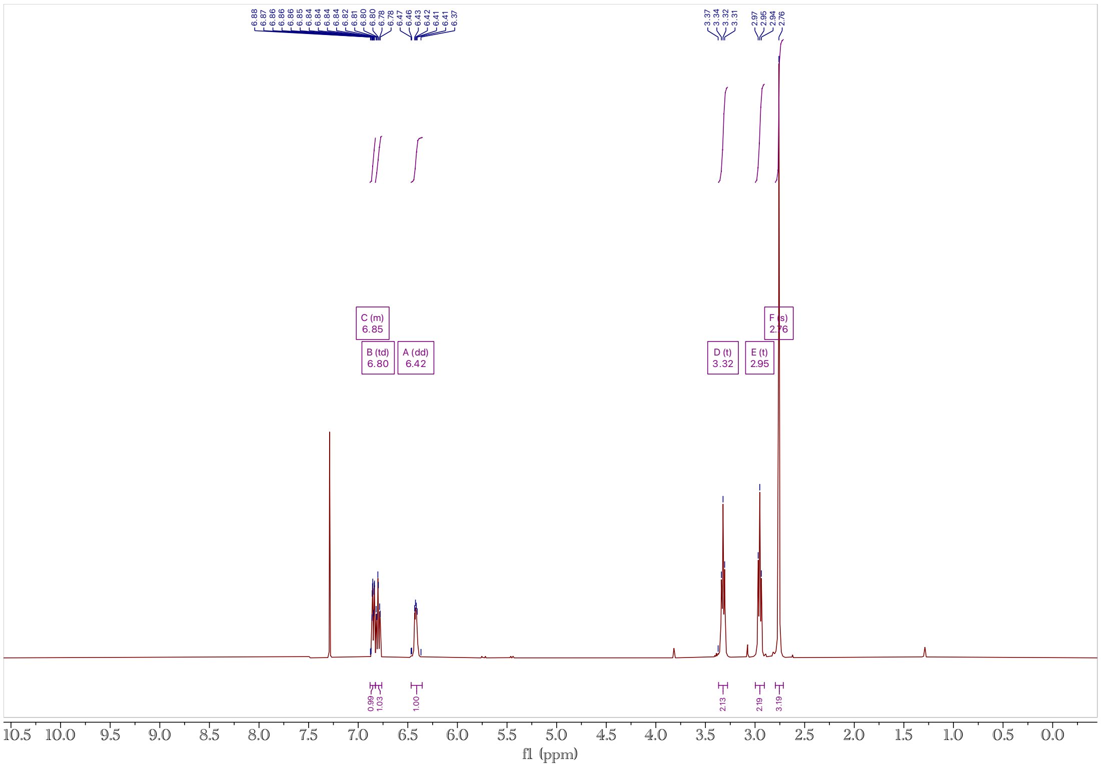

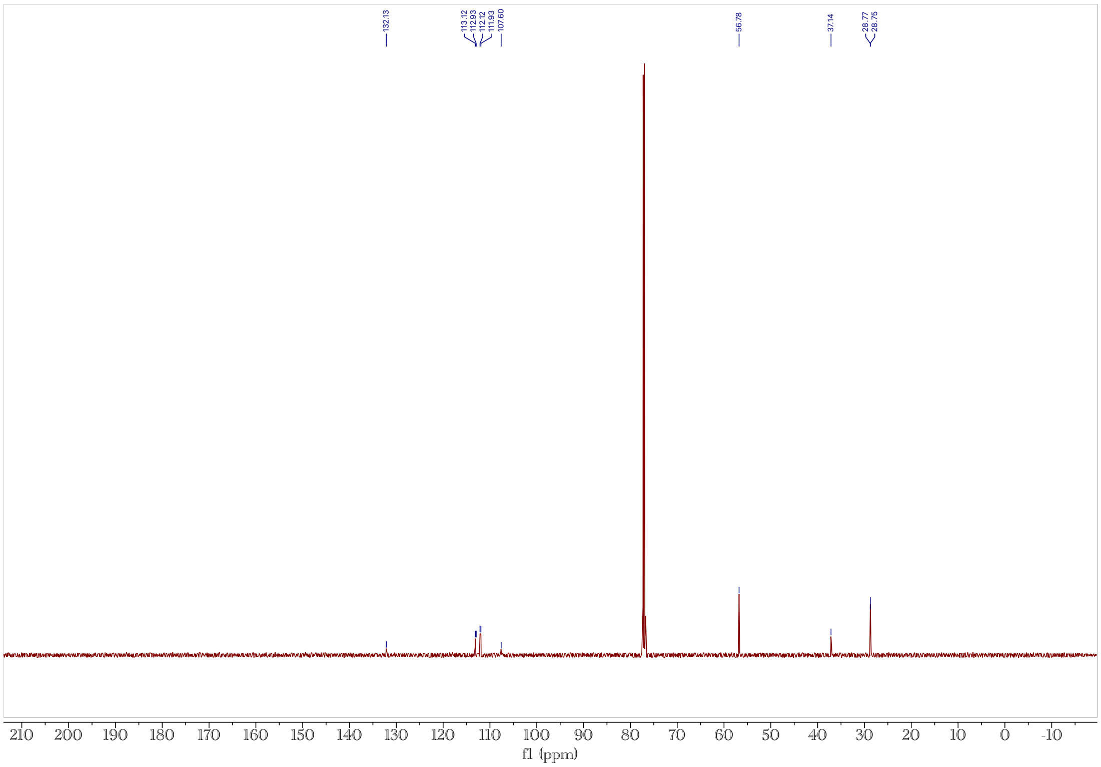

132.13
113.12
112.93
112.12
111.93
107.60
56.78
37.14
28.77
28.75
210 200 190 180 170 160 150 140 130 120 110 100 90 80 70 60 50 40 30 20 10 0 -10
f1 (ppm)

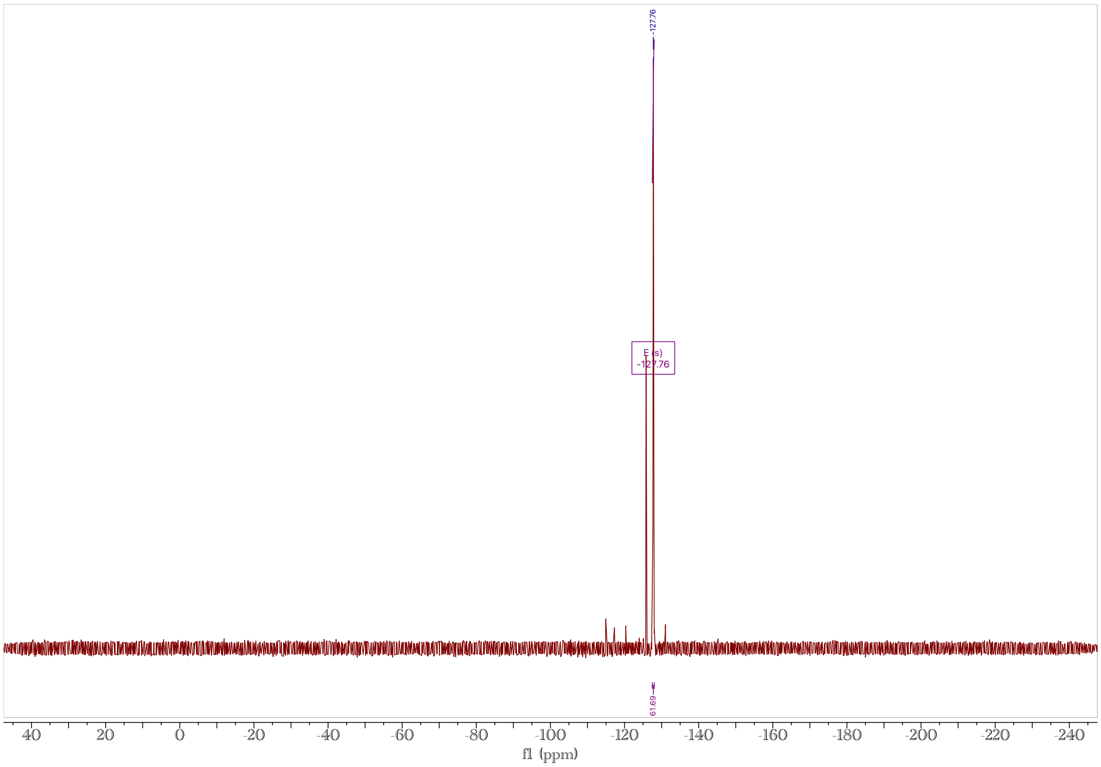

-127.76
F (s)
-127.76
61.69
40 20 0 -20 -40 -60 -80 -100 -120 -140 -160 -180 -200 -220 -240
f1 (ppm)

1-methyl-5-(trifluoromethyl)indoline (18)

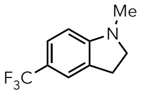

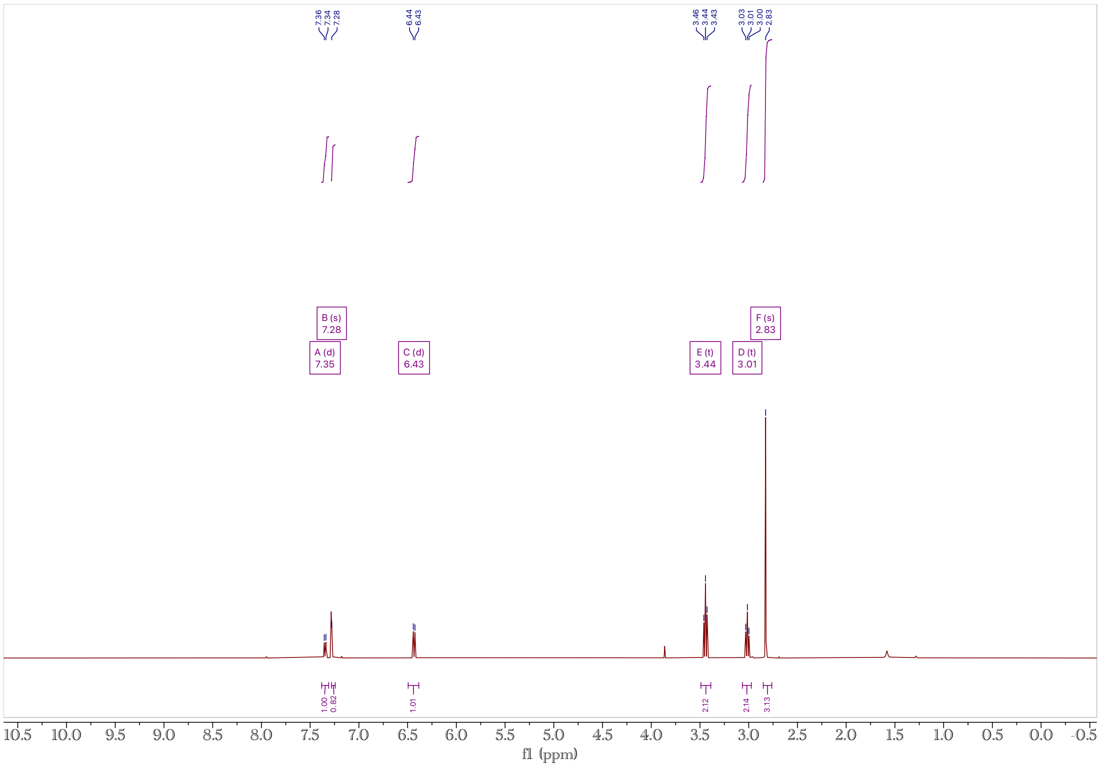

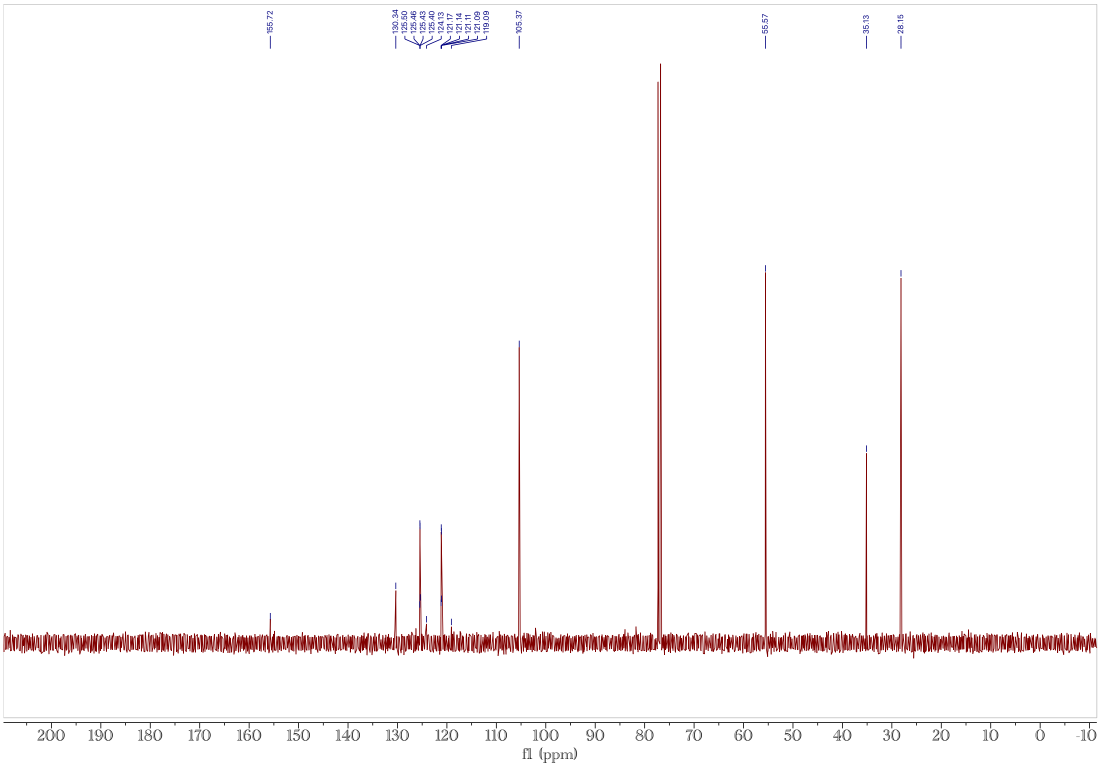
155.72
130.34
125.50
125.46
125.43
125.40
124.13
121.17
121.14
121.11
121.09
119.09
105.37
55.57
35.13
28.15
200 190 180 170 160 150 140 130 120 110 100 90 80 70 60 50 40 30 20 10 0 -10
f1 (ppm)

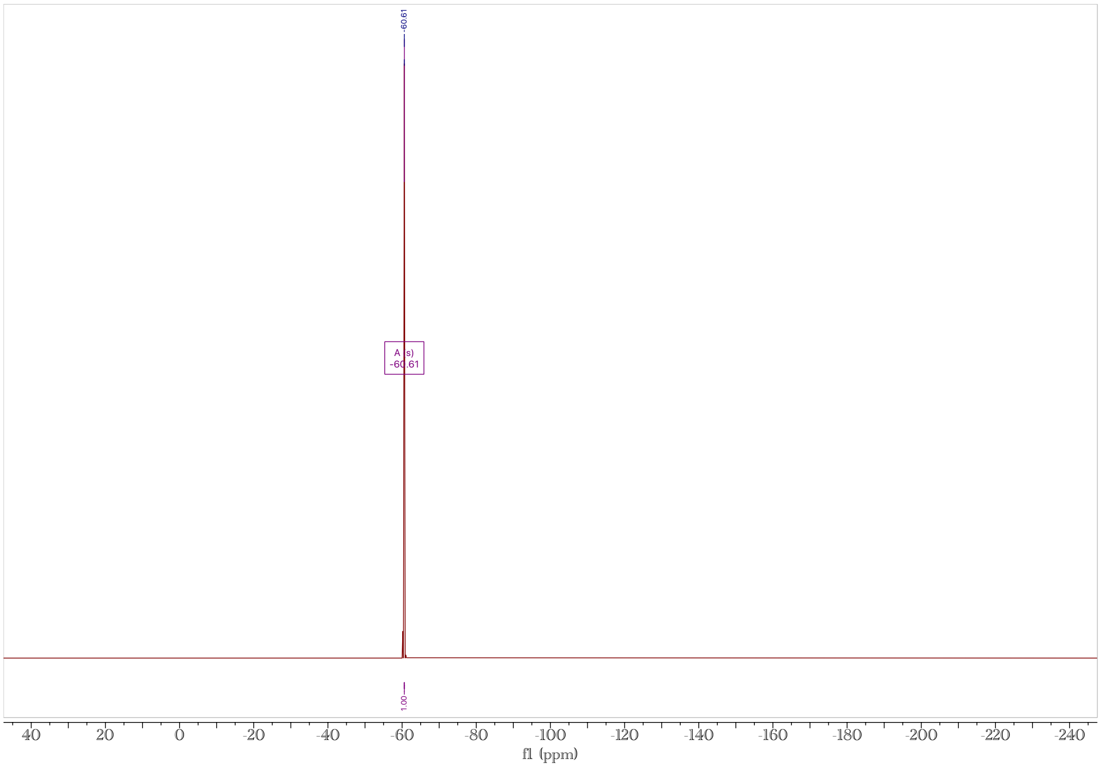
-60.61
A (s)
-60.61
1.00
40 20 0 -20 -40 -60 -80 -100 -120 -140 -160 -180 -200 -220 -240
f1 (ppm)

1-methyl-1,2,3,4-tetrahydroquinoline (19)

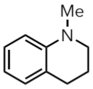

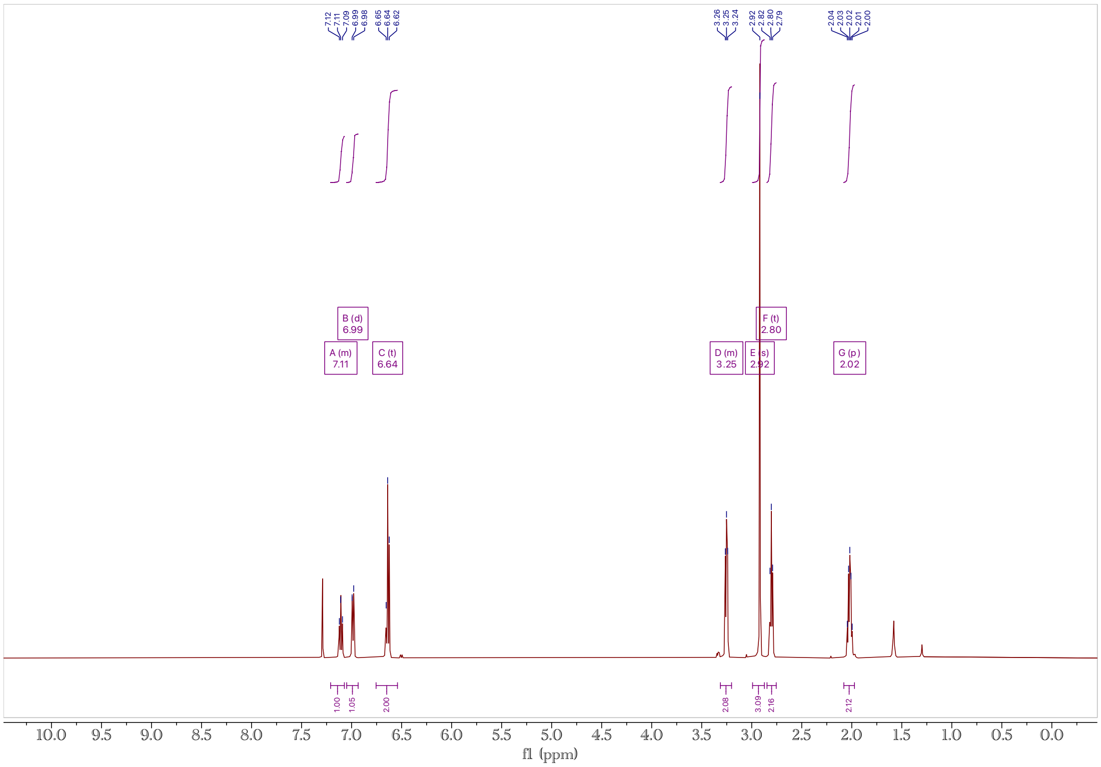

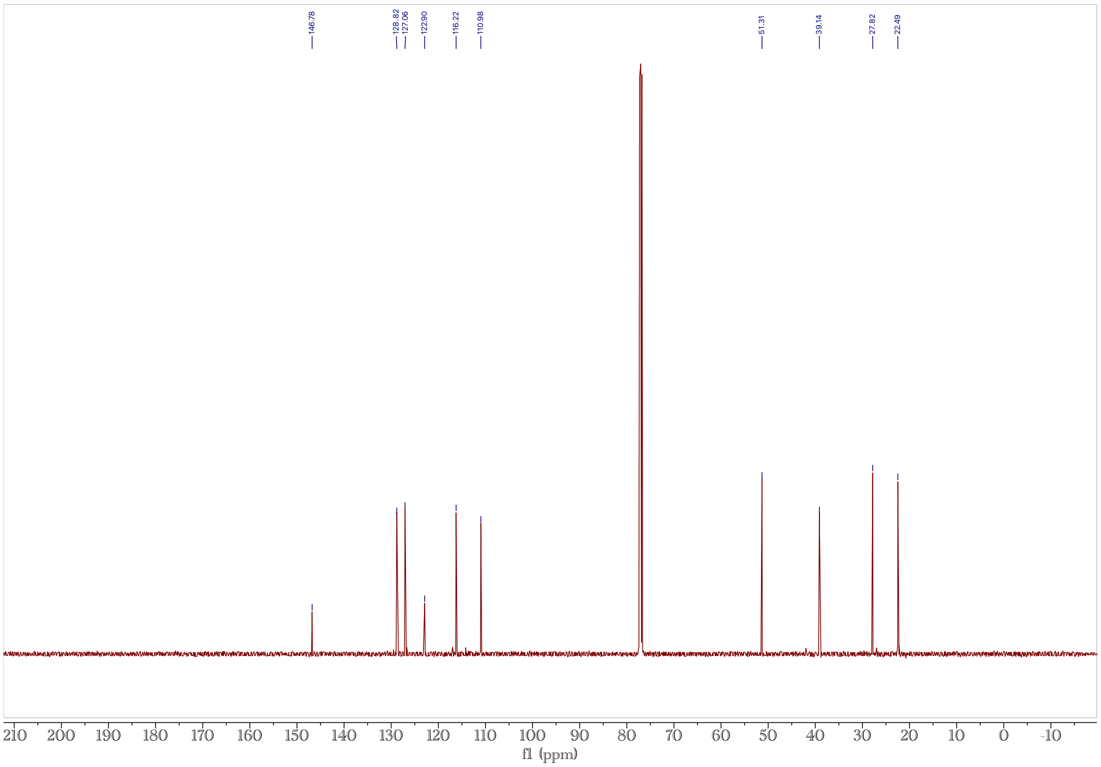

1,4-dimethyl-1,2,3,4-tetrahydropyrazino[2,3-*b*]pyrazine (21h)

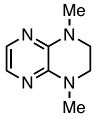

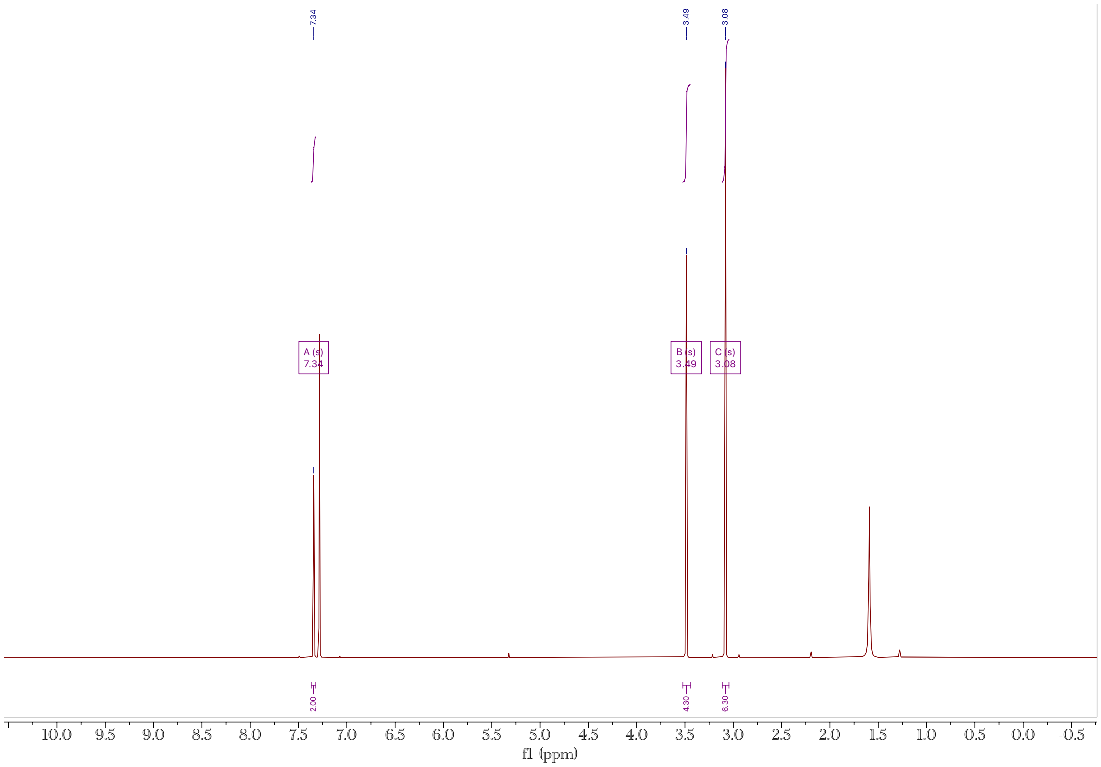

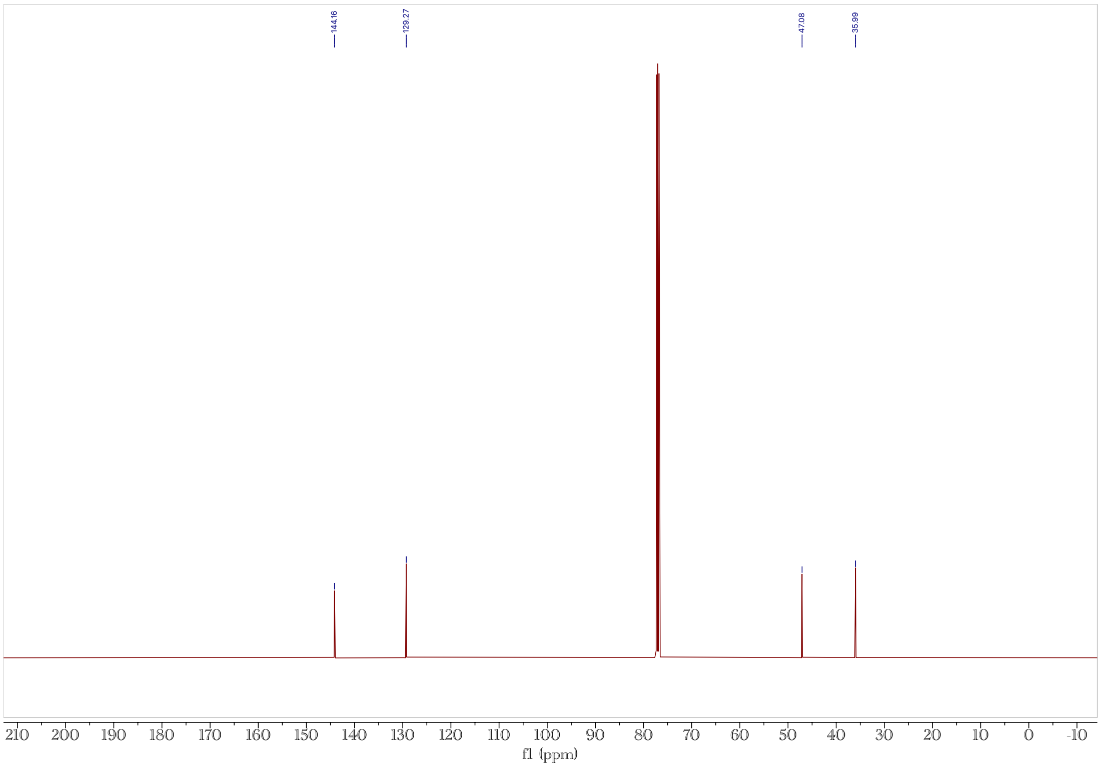

1,4-dimethyl-1,2,3,4-tetrahydropyrazino[2,3-b]quinoxaline (22h)

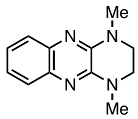
Me
N
N
N
N
Me

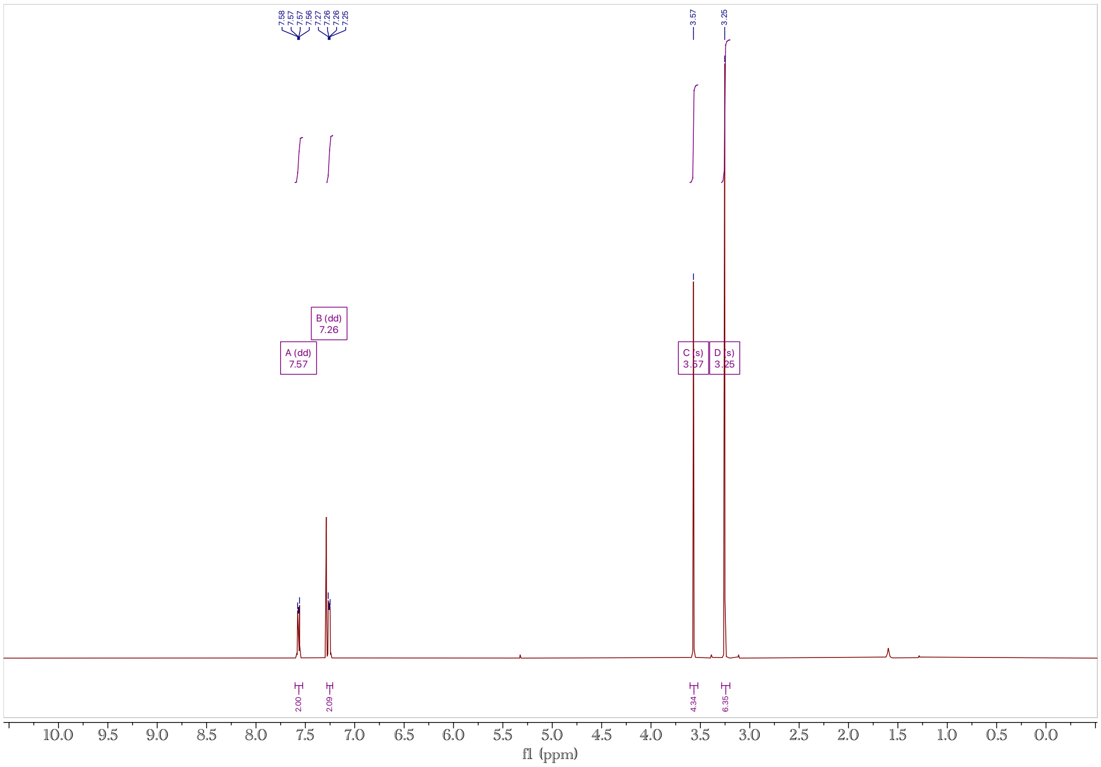
7.58
7.57
7.57
7.56
7.27
7.26
7.26
7.25
3.57
3.25
B (dd)
7.26
A (dd)
7.57
C (s)
3.57
D (s)
3.25
2.00
2.09
4.34
6.35
10.0 9.5 9.0 8.5 8.0 7.5 7.0 6.5 6.0 5.5 5.0 4.5 4.0 3.5 3.0 2.5 2.0 1.5 1.0 0.5 0.0
f1 (ppm)

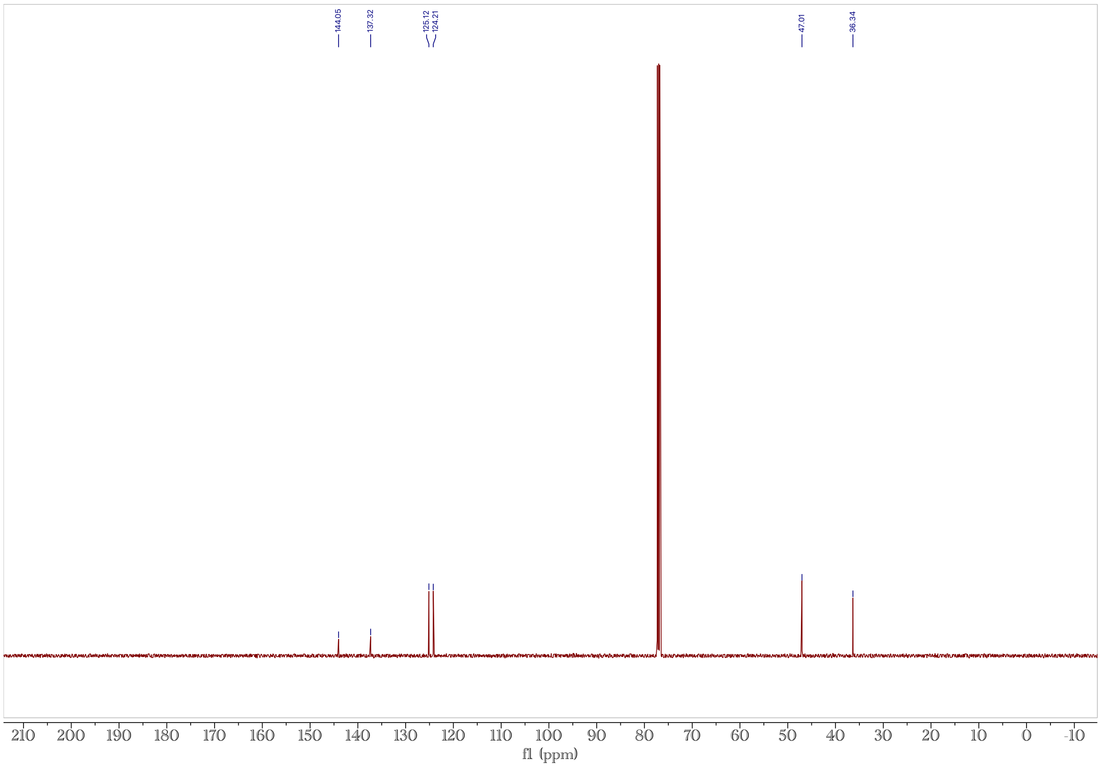
144.05
137.32
125.12
124.21
47.01
36.34
210 200 190 180 170 160 150 140 130 120 110 100 90 80 70 60 50 40 30 20 10 0 -10
f1 (ppm)

7-chloro-1,4-dimethyl-1,2,3,4-tetrahydropyrido[2,3-*b*]pyrazine (23h)

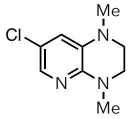

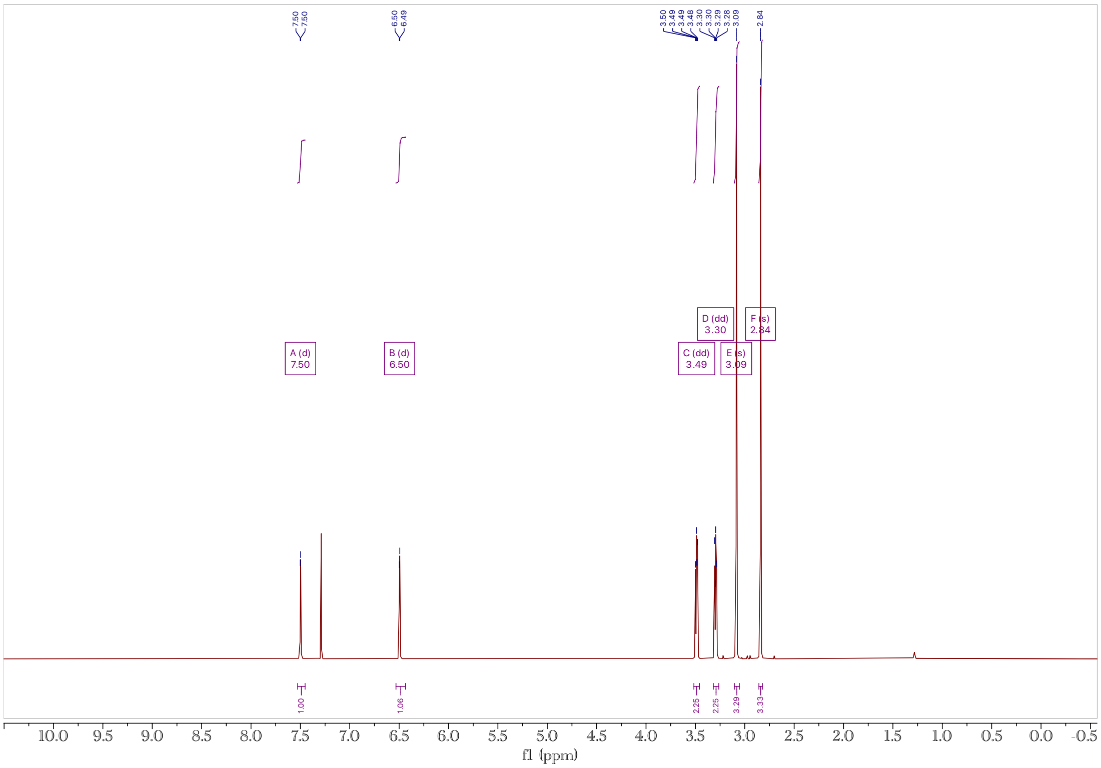

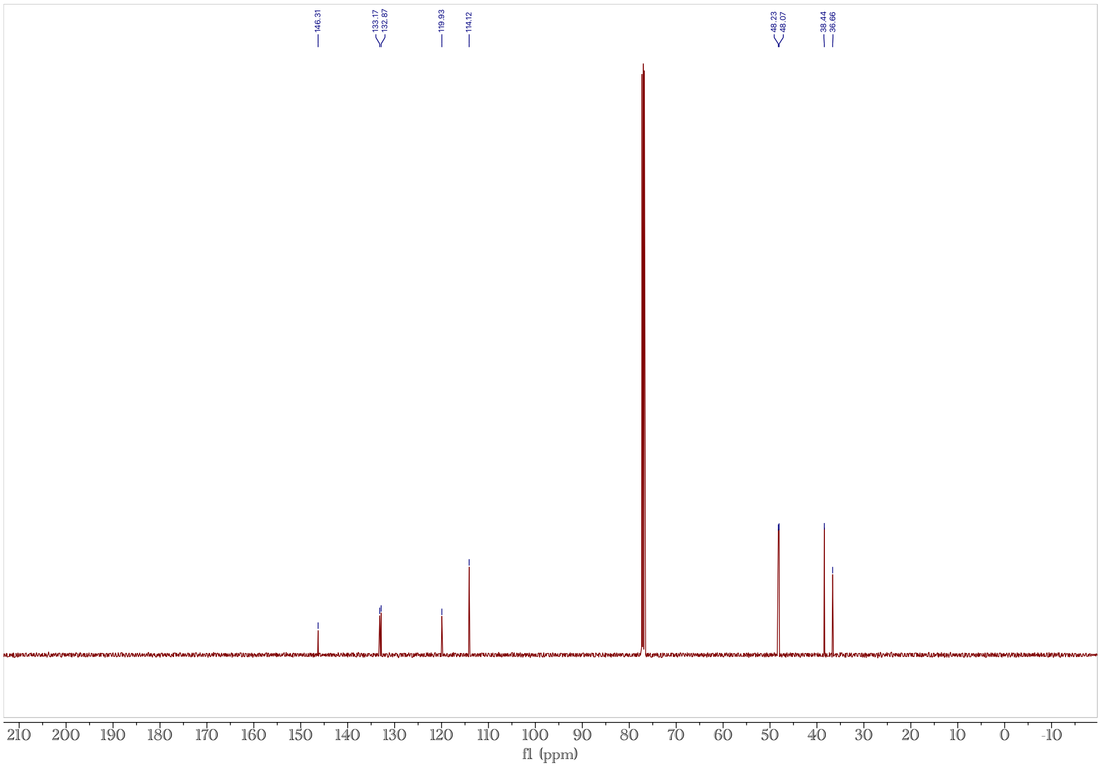

5,8-dimethyl-5,6,7,8-tetrahydropteridine (24h)

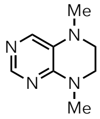

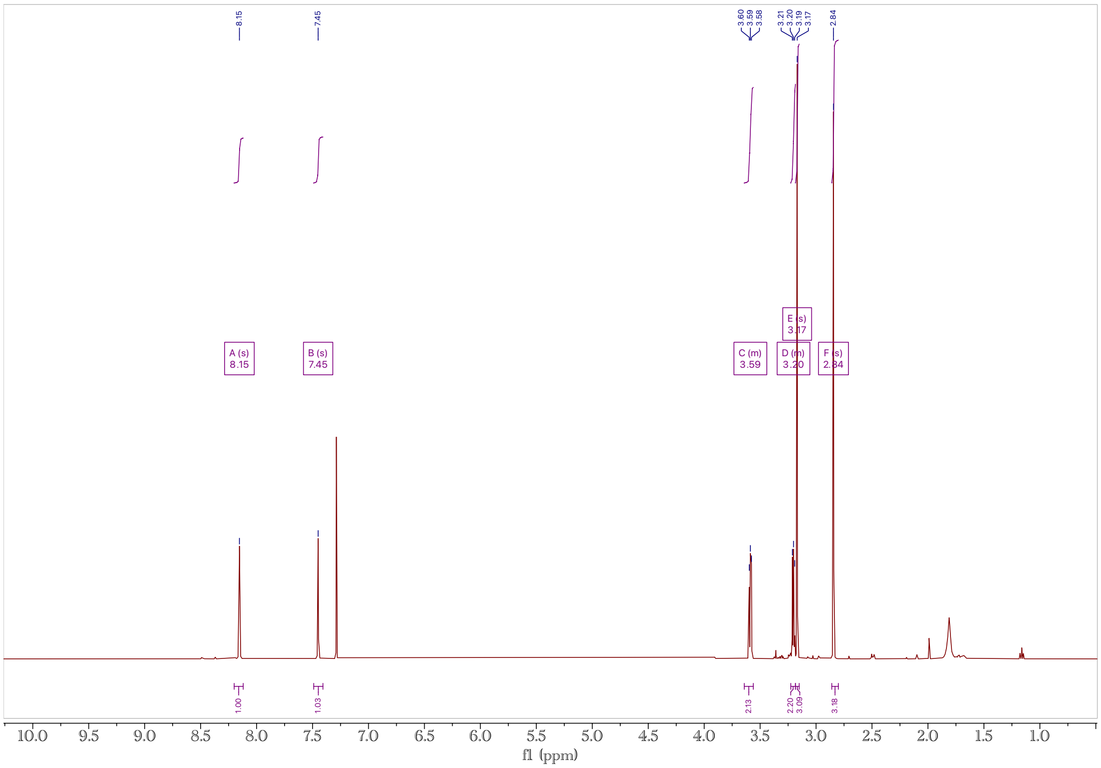

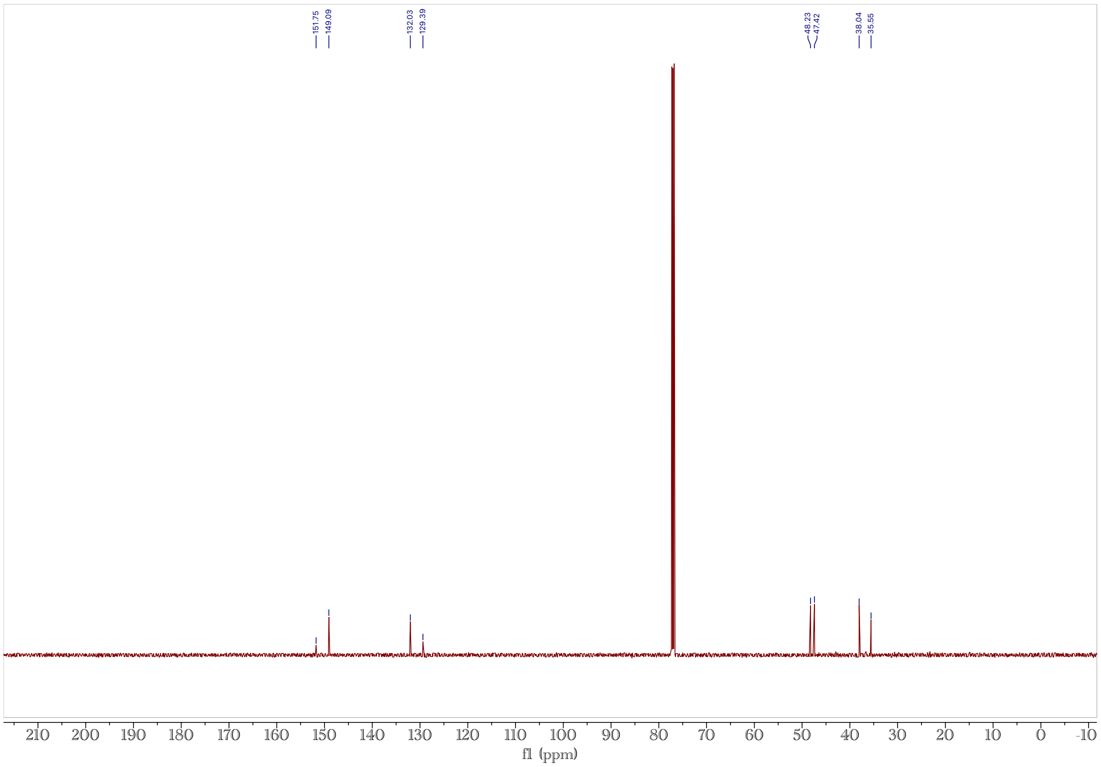

5,8-dimethyl-5,6,7,8-tetrahydropteridin-2-amine (25h)

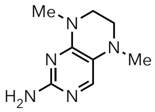

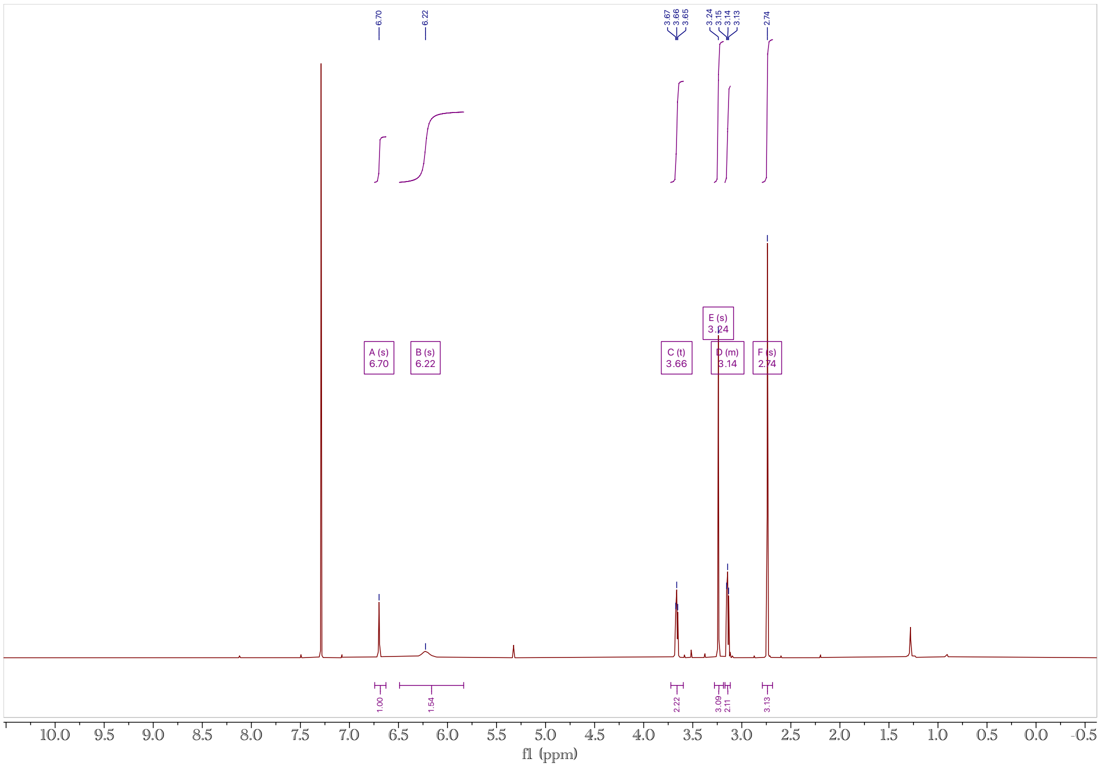

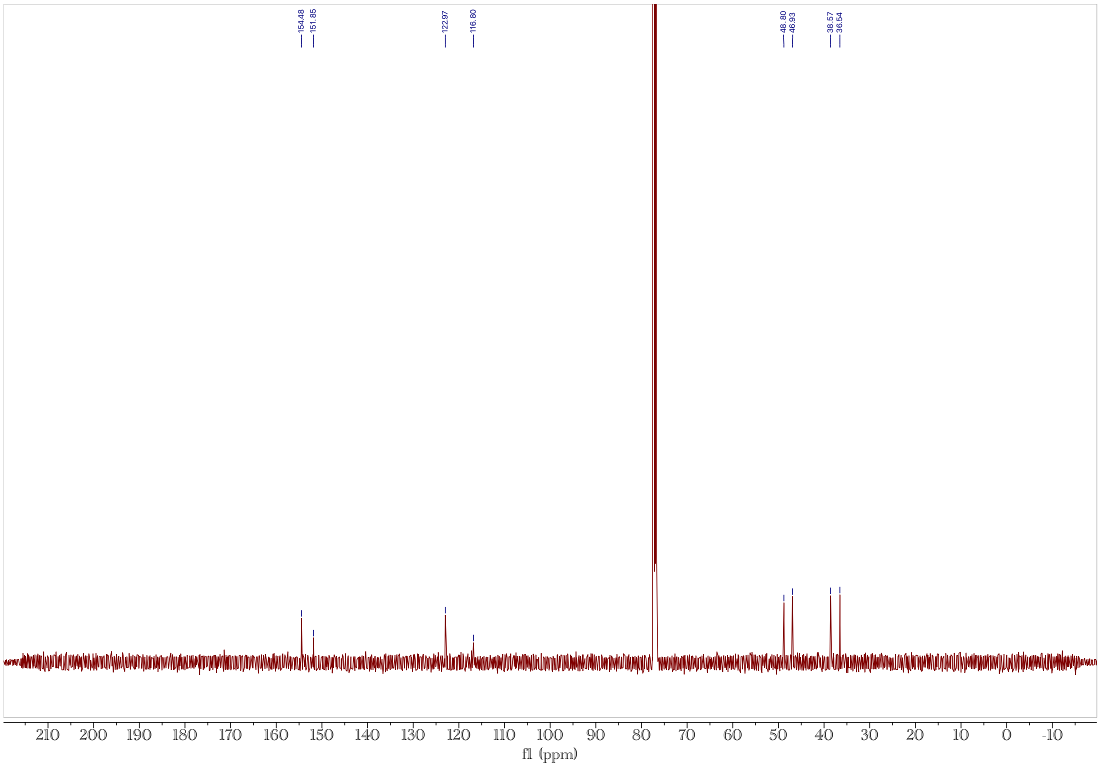

1,4-dimethyl-1,2,3,4-tetrahydropyrido[3,4-*b*]pyrazin-7-amine (26h)

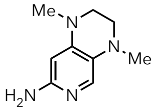

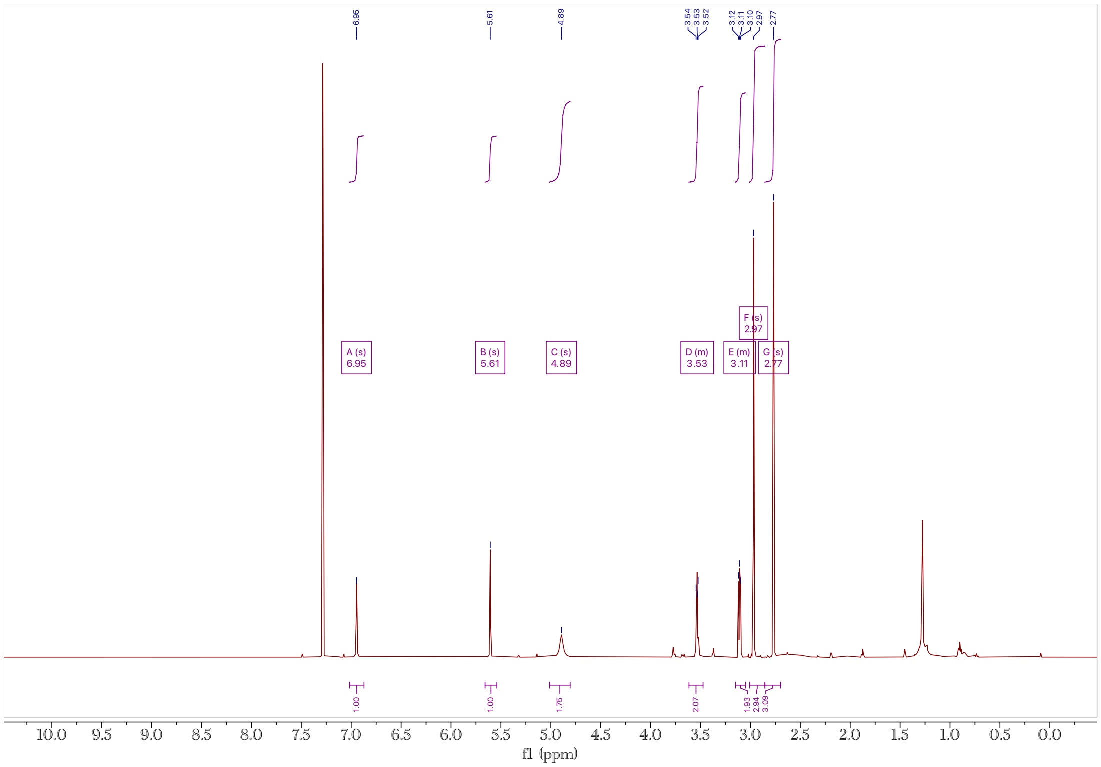

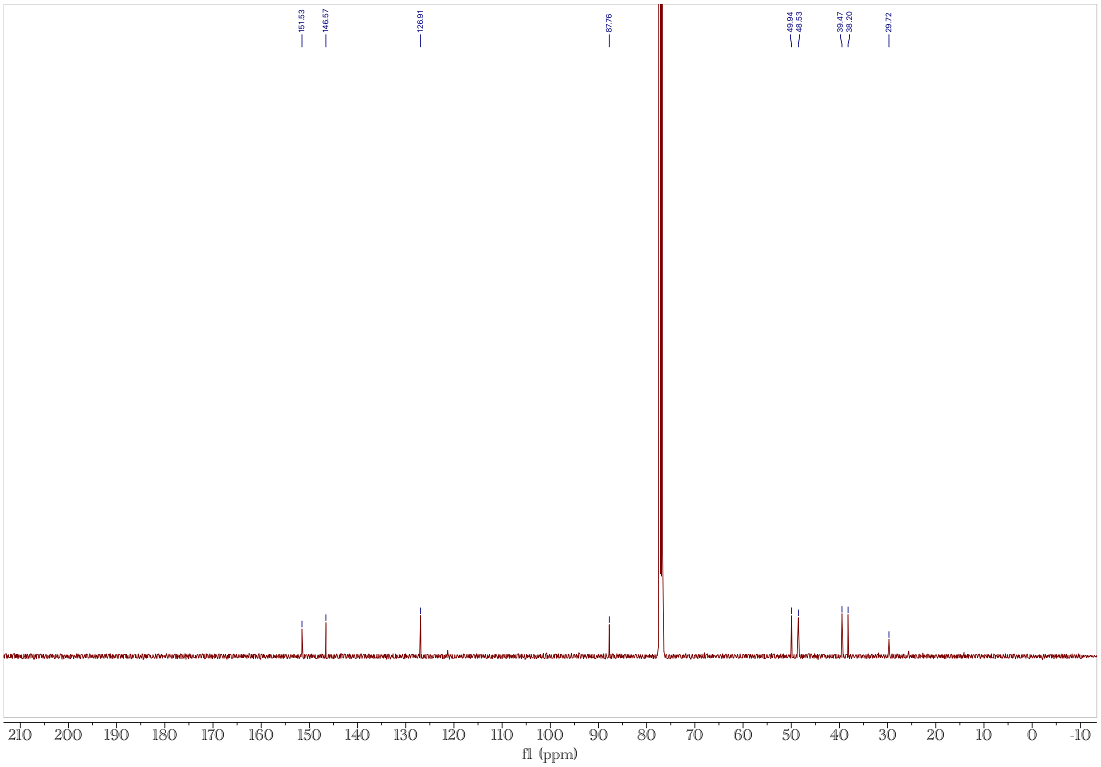

1,4-dimethyl-1,2,3,4-tetrahydropyrido[3,4-*b*]pyrazine (27h)

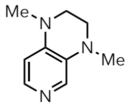

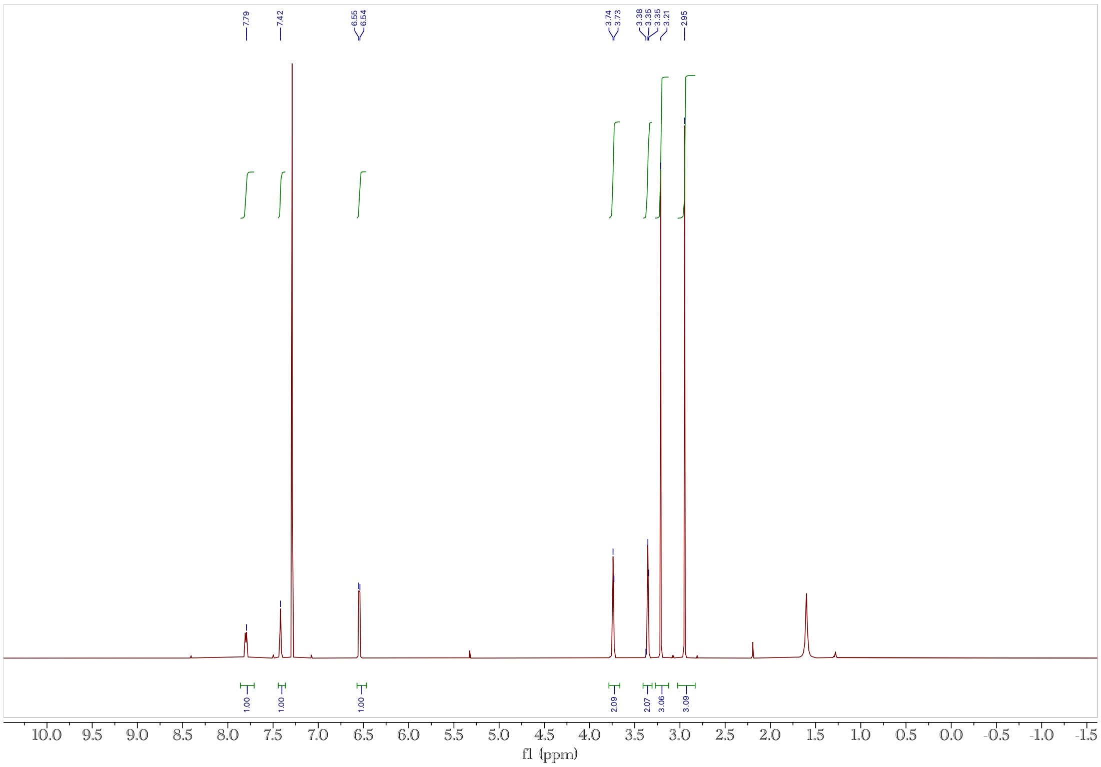

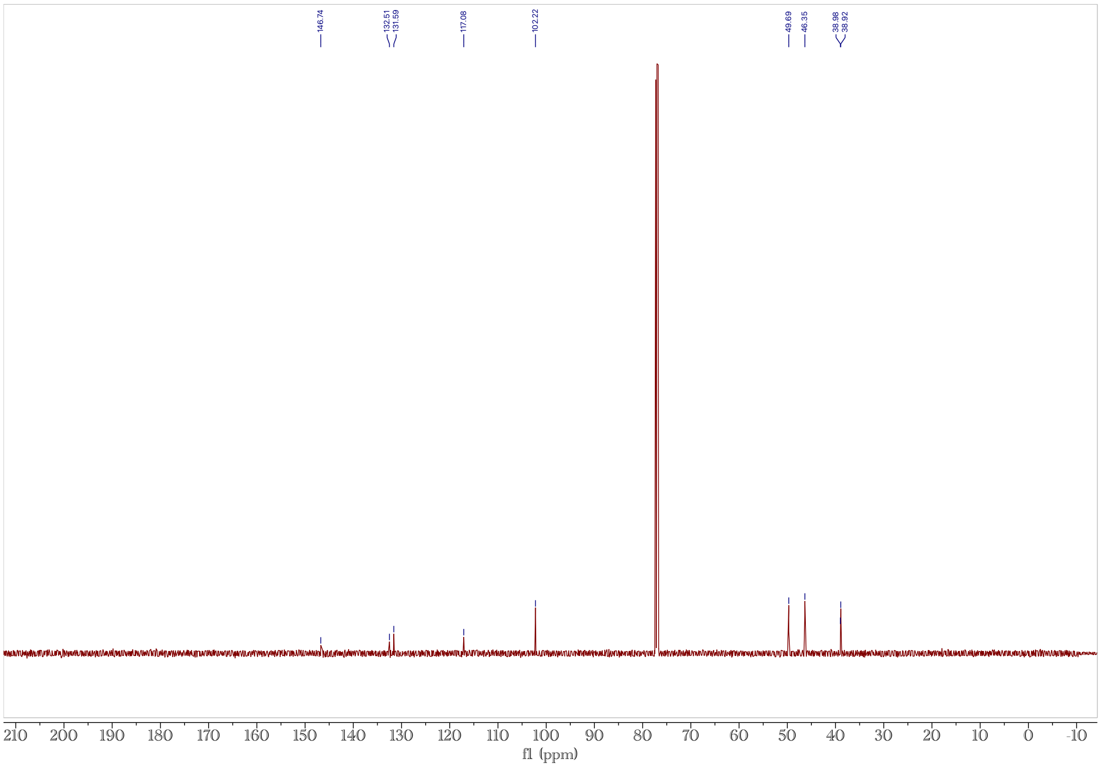

1,4-dimethyl-1,2,3,4-tetrahydropyrido[2,3-*b*]pyrazine (28h)

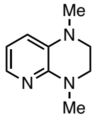

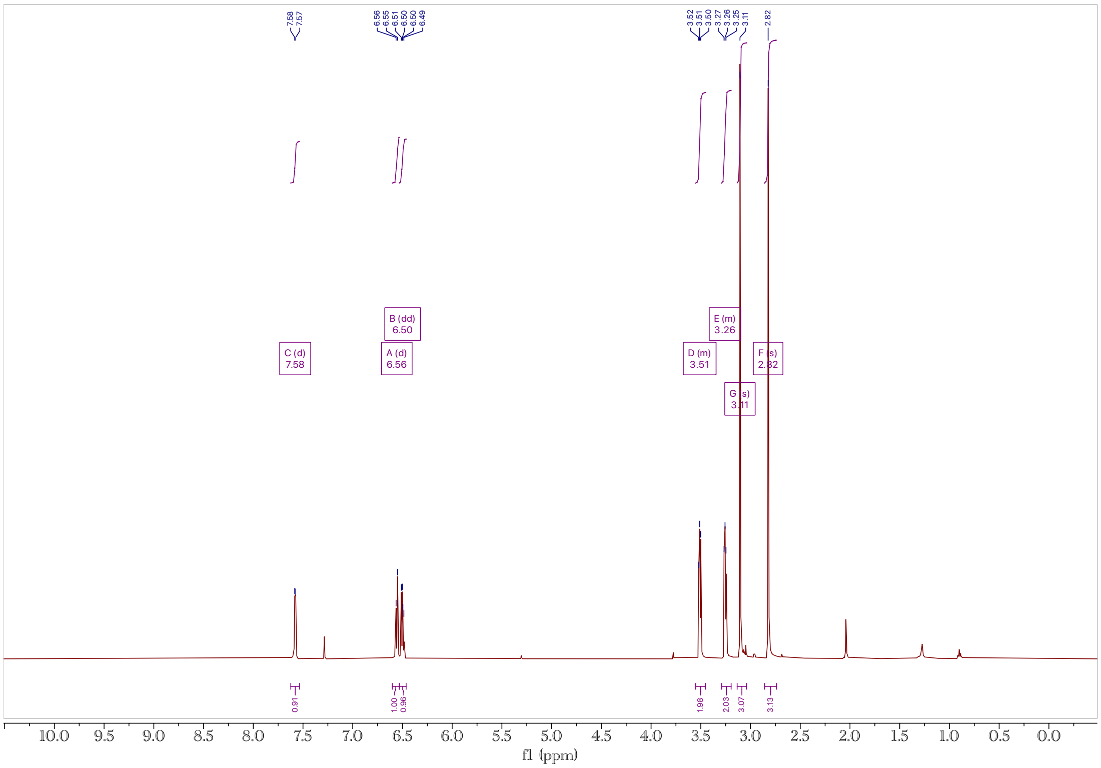

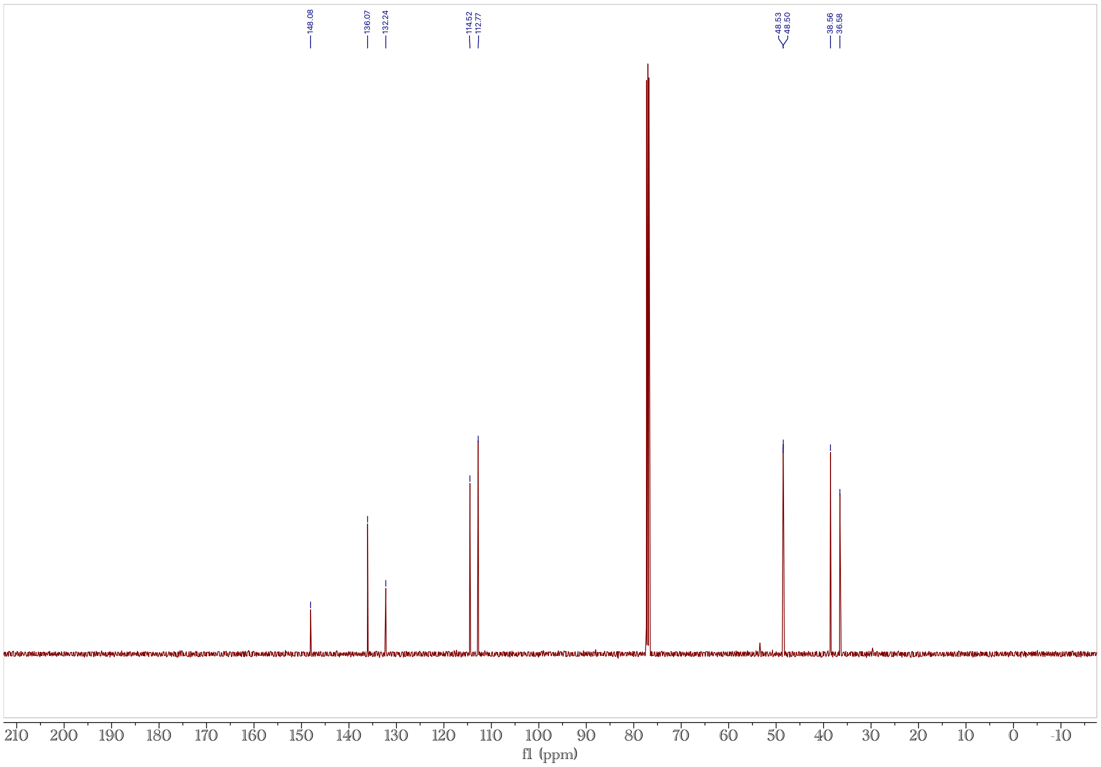

1,2,3,4-tetrahydropyrazino[2,3-*b*]pyrazine (21i)

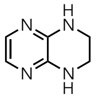

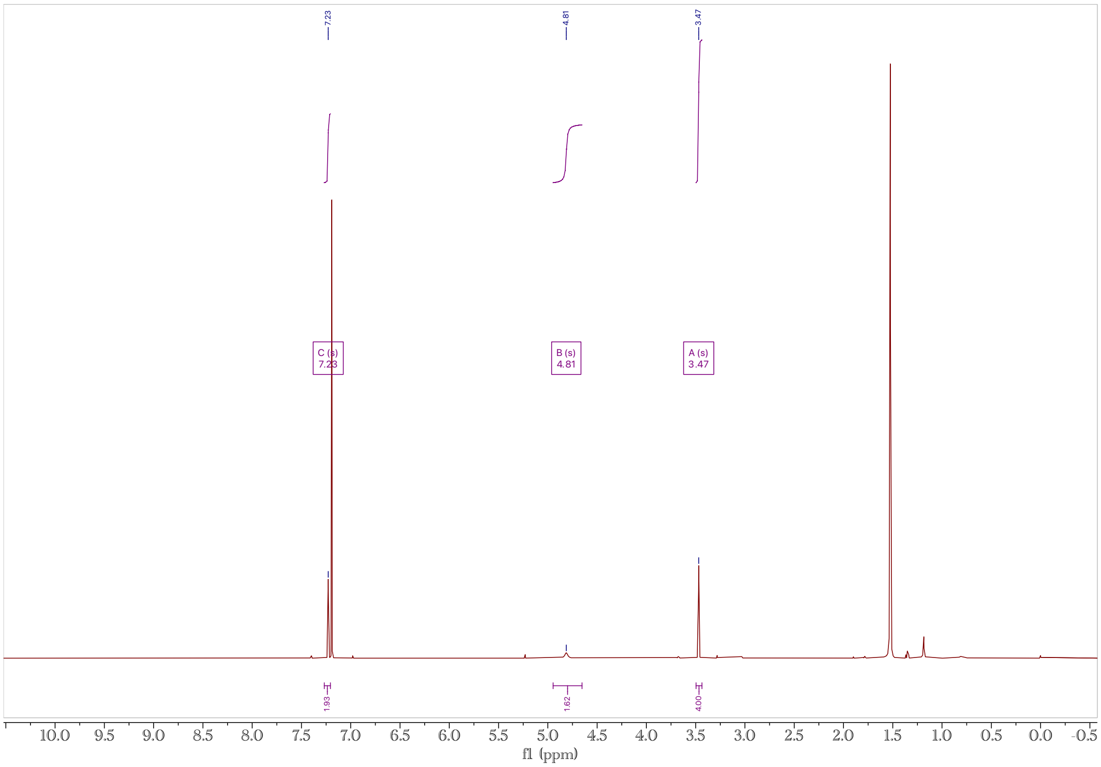

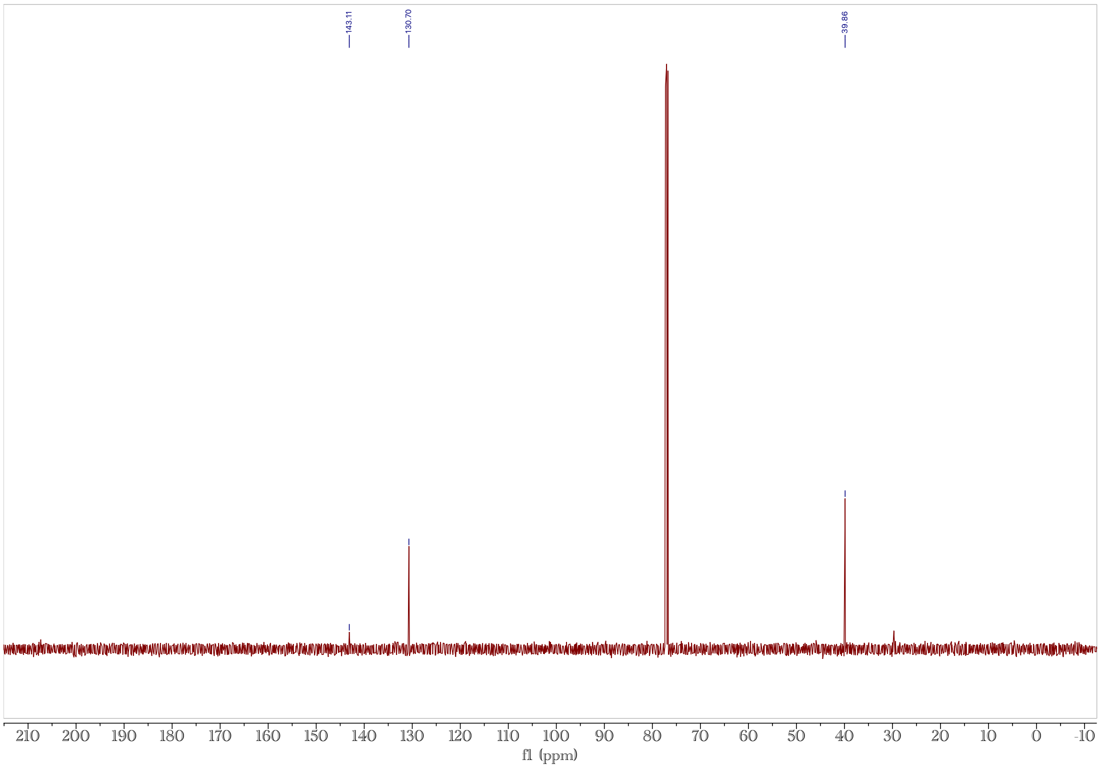

1-methyl-1,2,3,4-tetrahydropyrazino[2,3-*b*]pyrazine (21j)

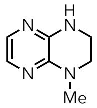

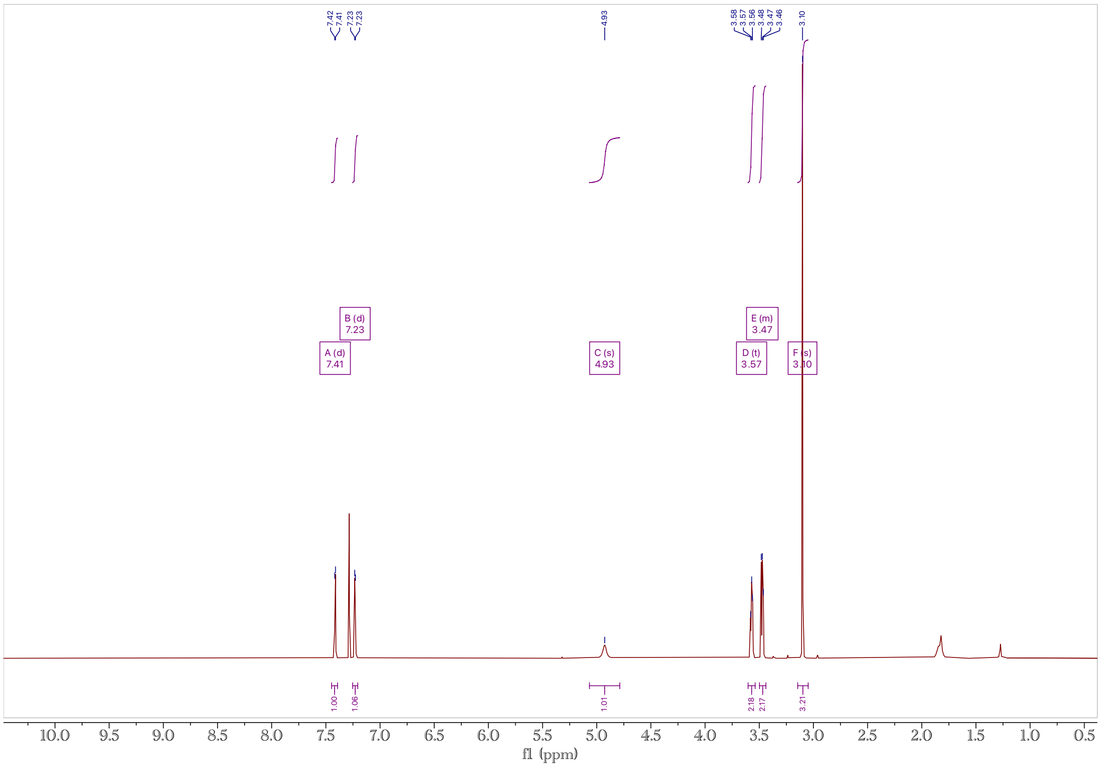

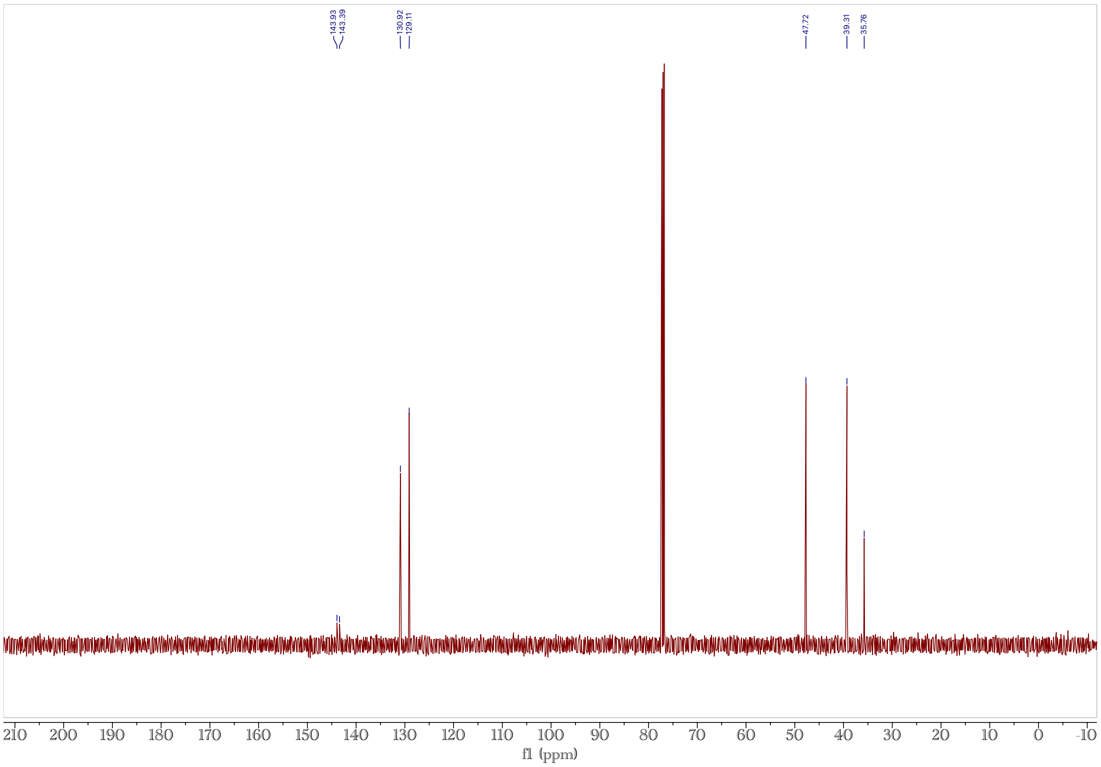

5,9-dimethyl-6,7,8,9-tetrahydro-5*H*-pyrazino[2,3-*b*]azepine (21k)

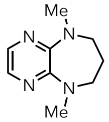

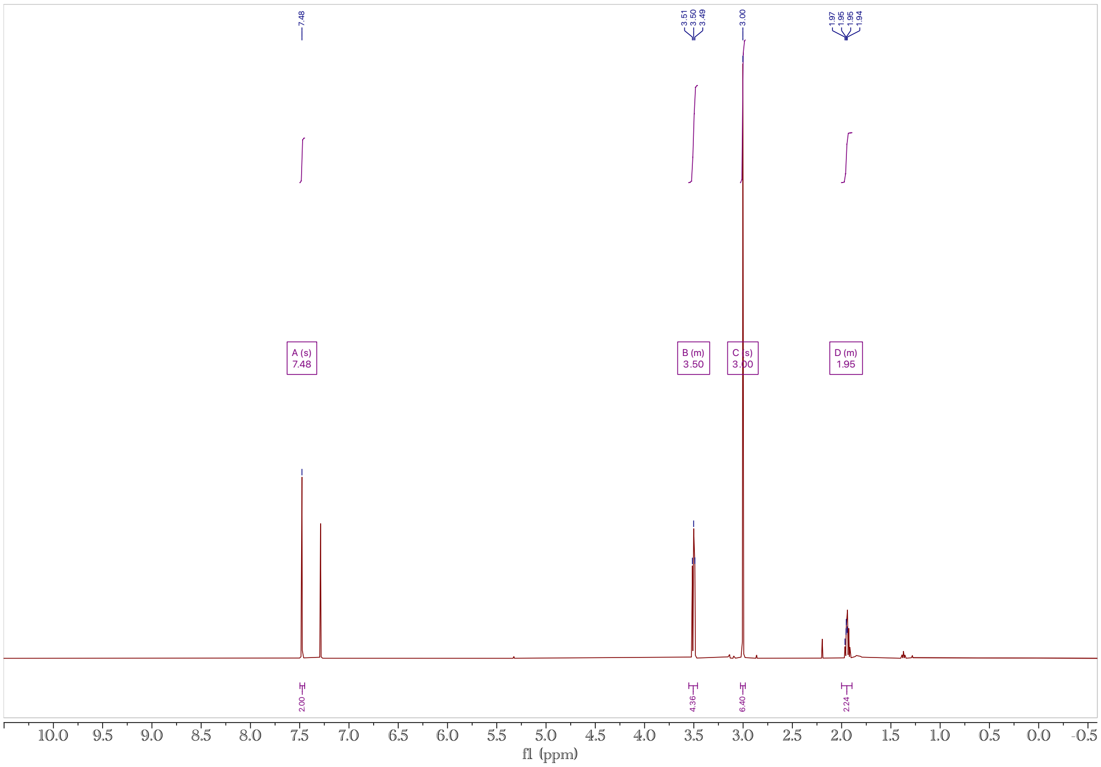

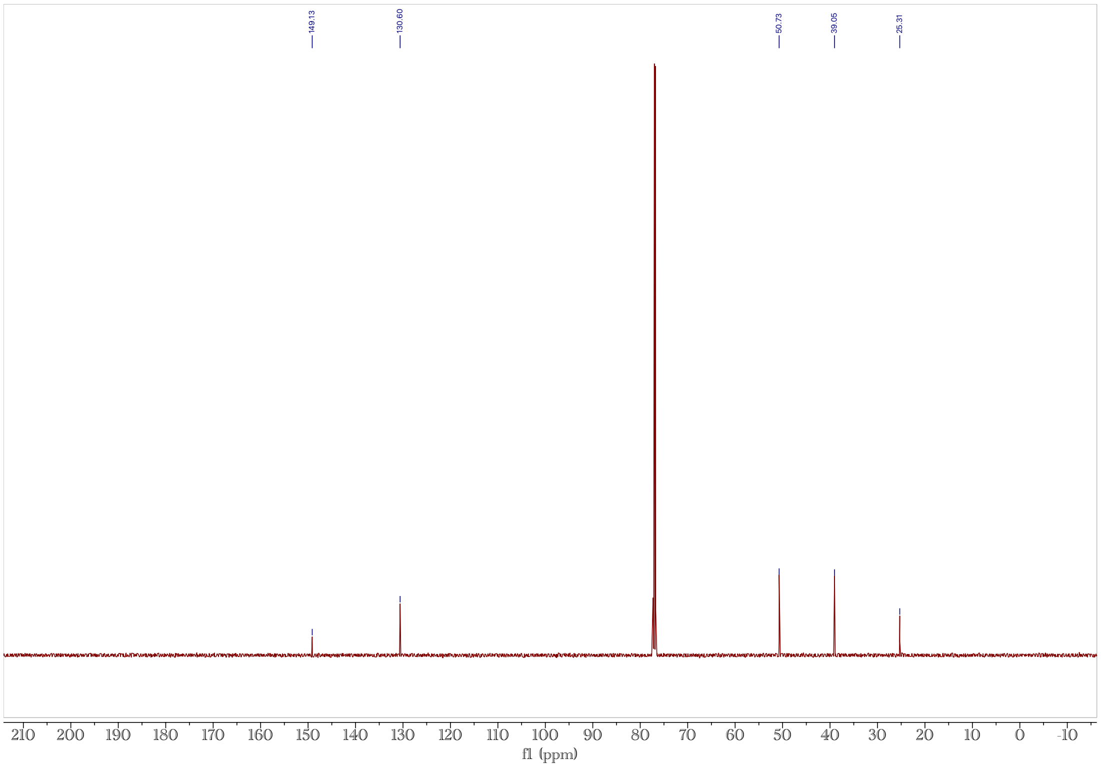

1,4-diethyl-1,2,3,4-tetrahydropyrazino[2,3-*b*]pyrazine (21l)

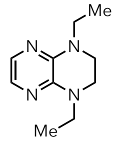

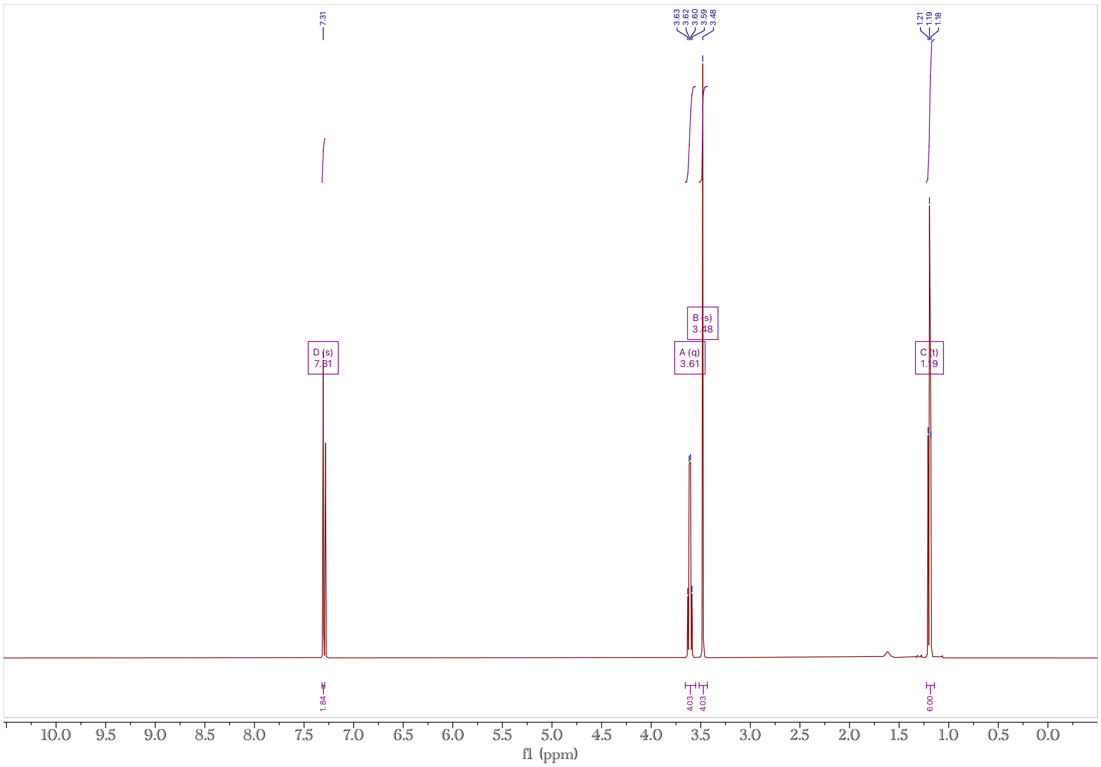

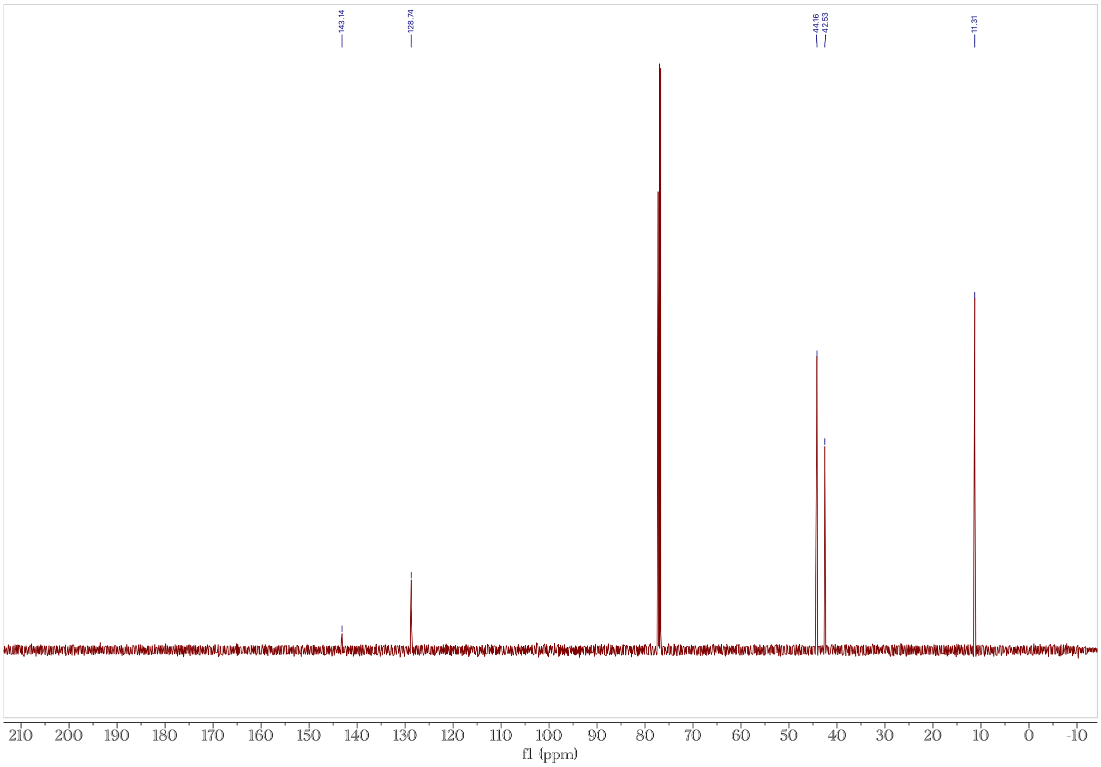

1,4-dibenzyl-1,2,3,4-tetrahydropyrazino[2,3-*b*]pyrazine (21m)

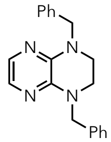

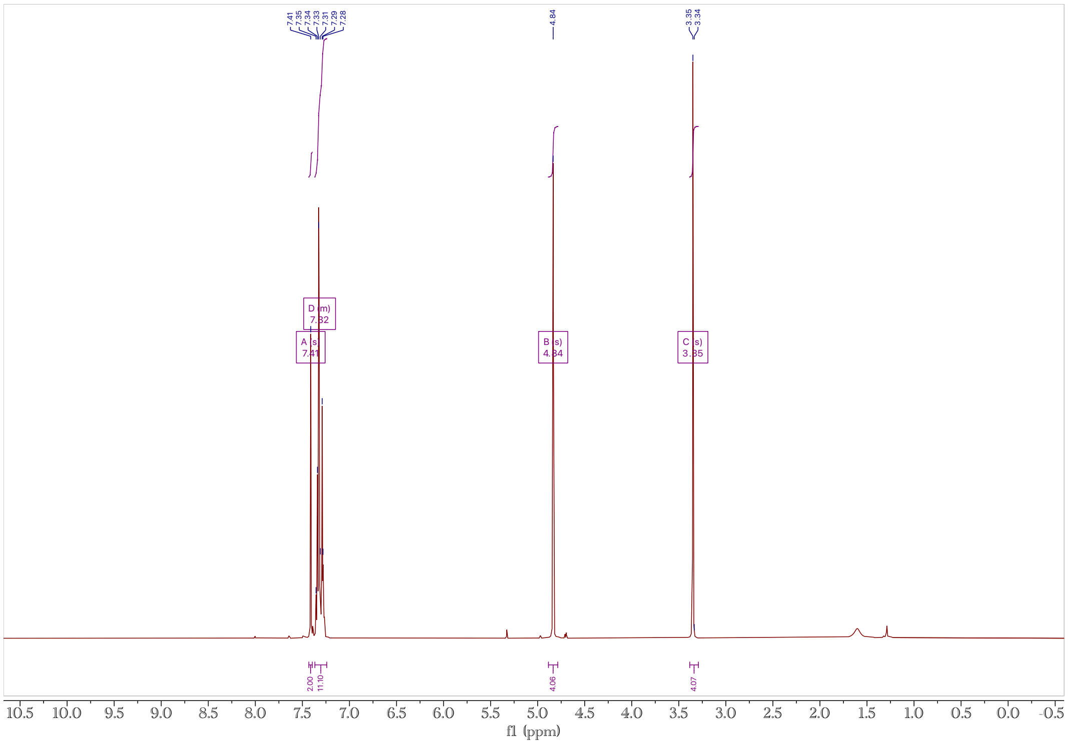

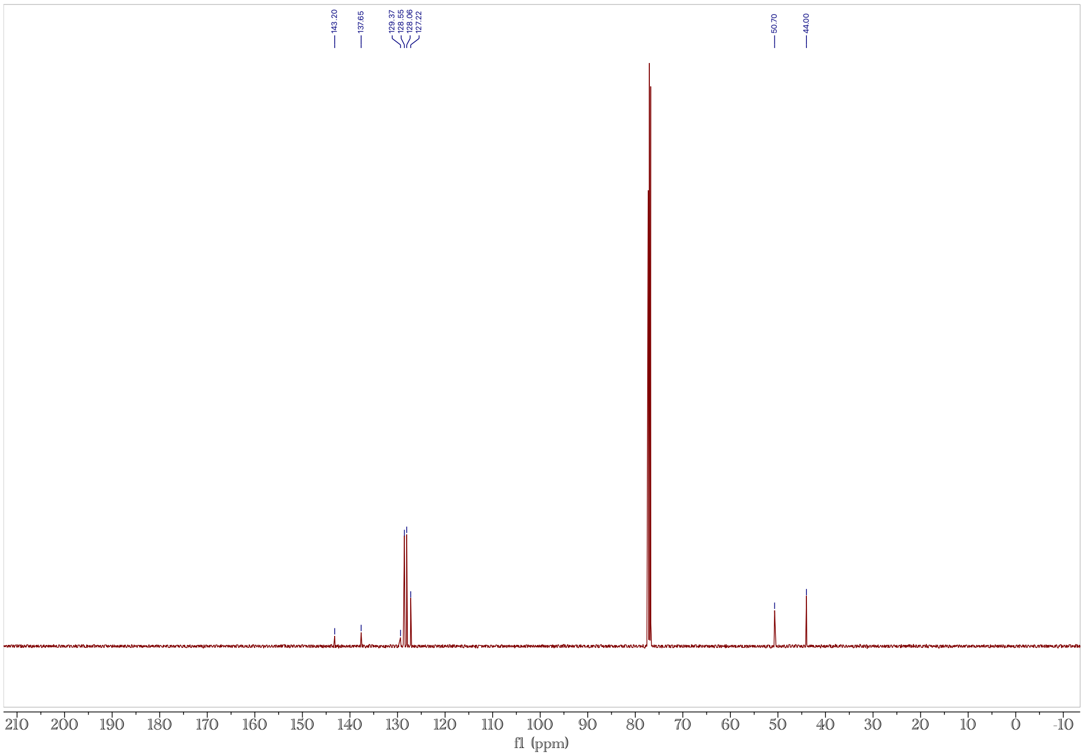

3,4-dihydro-2*H*-pyrazino[2,3-*b*][1,4]thiazine (21n)

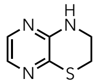

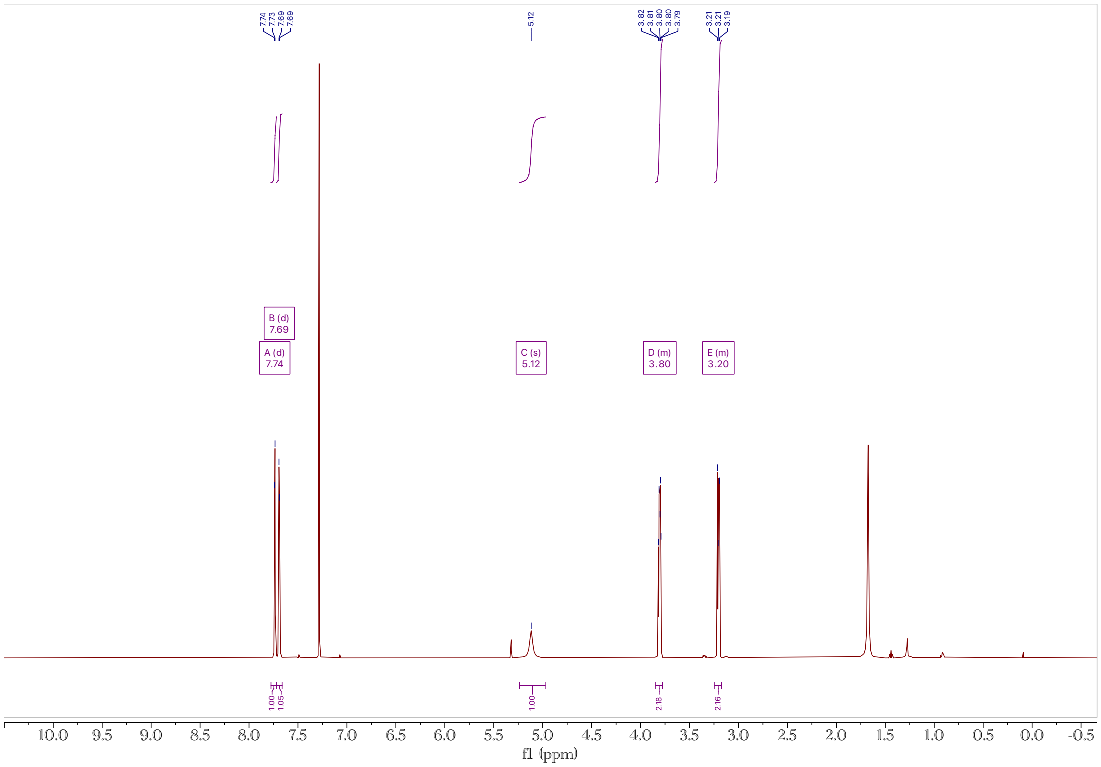

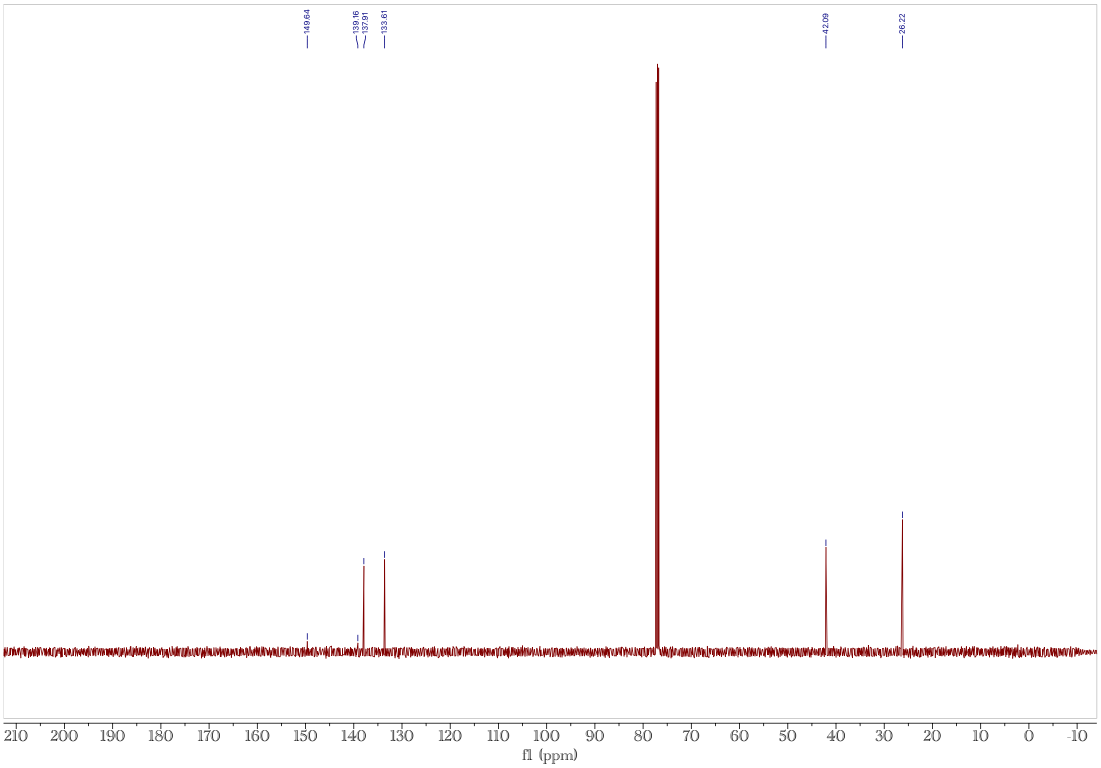

(5a*S*,9a*S*)-5,10-dimethyl-5,5a,6,7,8,9,9a,10-octahydropyrazino[2,3-*b*]quinoxaline (21o)

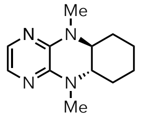

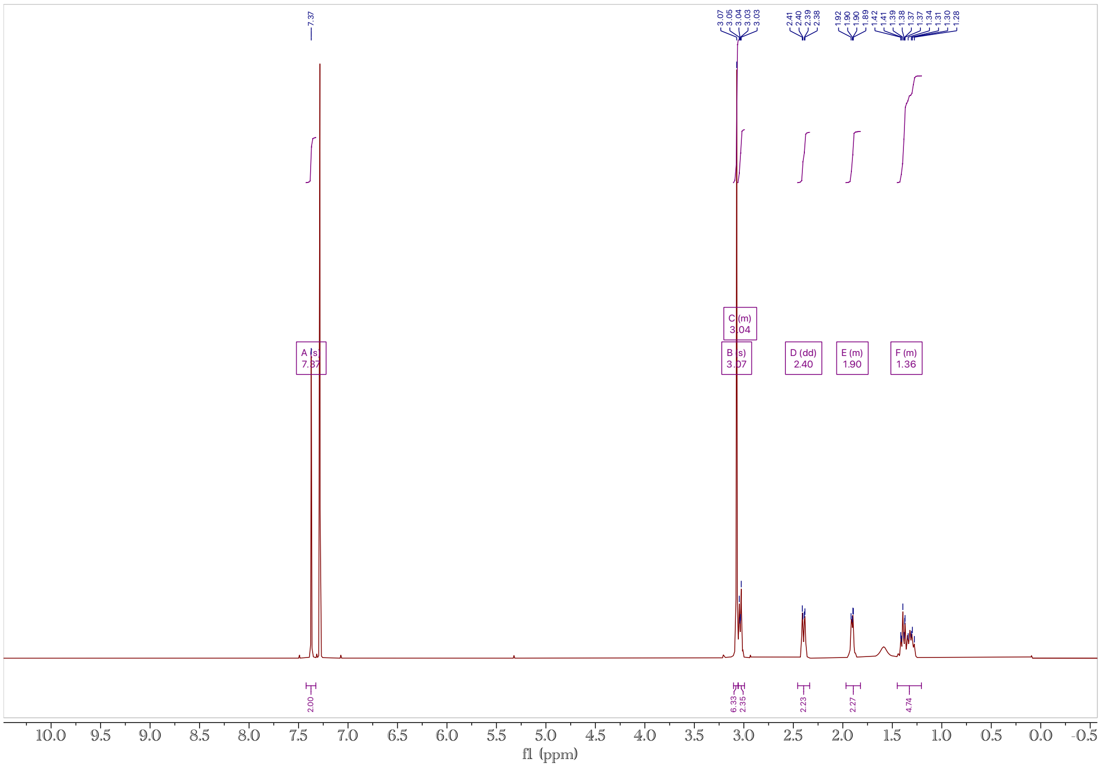

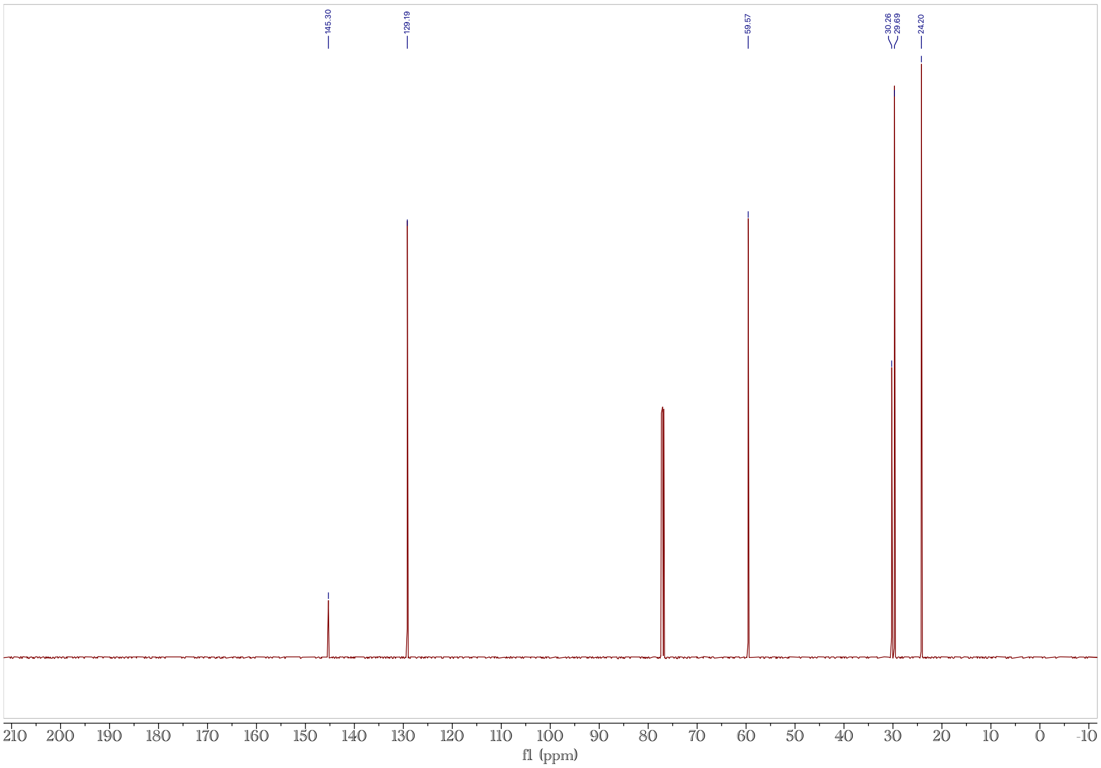

5,10-dimethyl-5,5a,6,7,8,9,9a,10-octahydropyrido[2,3-*b*]quinoxaline (28o)

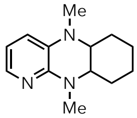

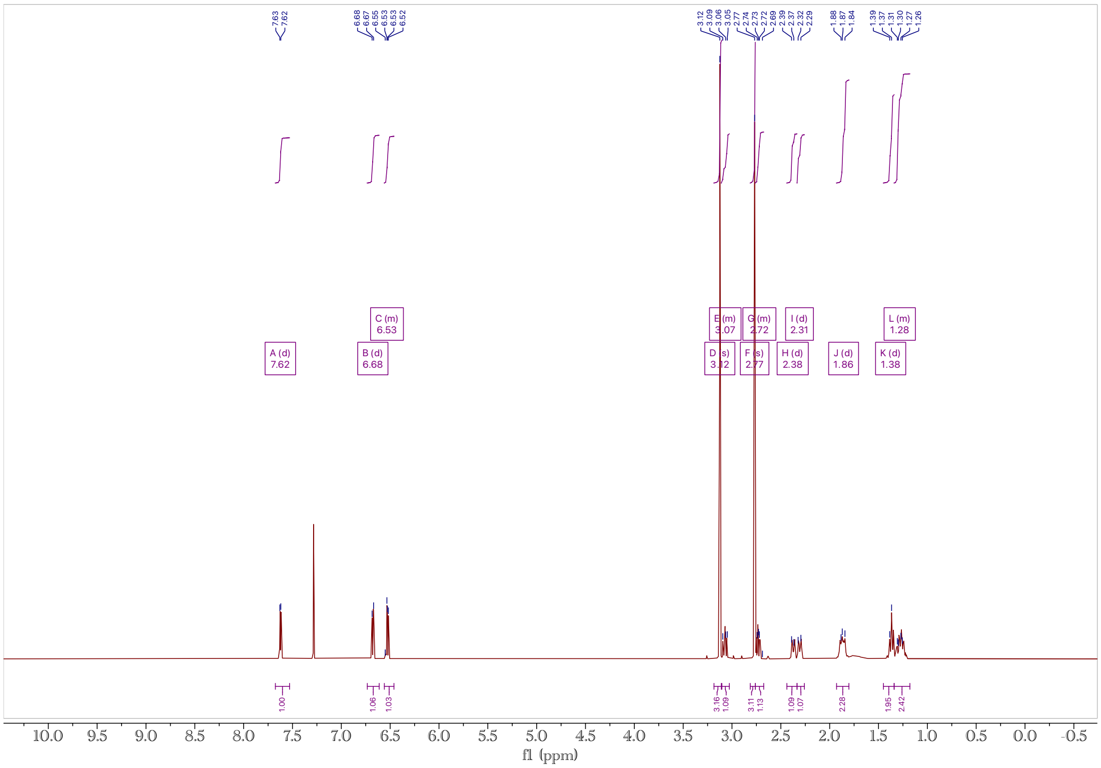

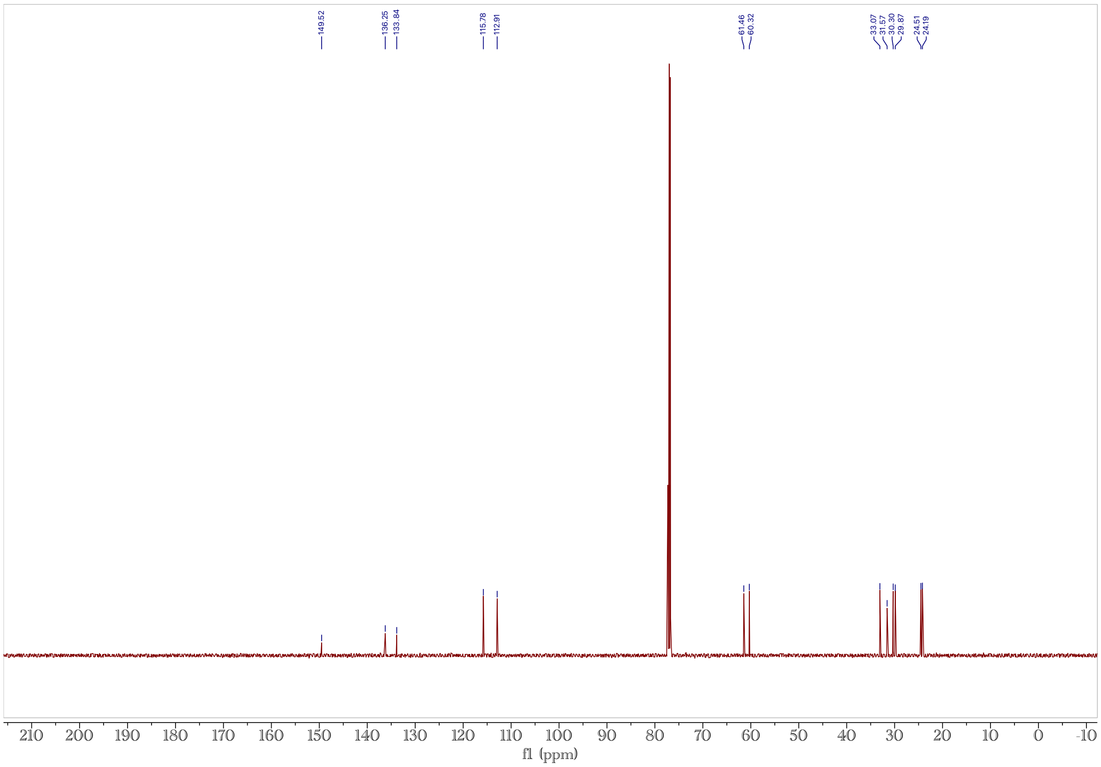

References

(1) Heinz, C.; Lutz, J. P.; Simmons, E. M.; Miller, M. M.; Ewing, W. R.; Doyle, A. G. Ni-Catalyzed Carbon–Carbon Bond-Forming Reductive Amination. *J. Am. Chem. Soc.* **2018**, *140* (6), 2292–2300. https://doi.org/10.1021/jacs.7b12212.

(2) Jarboe, S. G.; Terrazas, M. S.; Beak, P. The Endocyclic Restriction Test: The Geometries of Nucleophilic Substitutions at Sulfur(VI) and Sulfur(II). *J. Org. Chem.* **2008**, *73* (24), 9627–9632. https://doi.org/10.1021/jo8016428.

(3) Jiang, N.; Zhuo, X.; Wang, J. Active Plasmonics: Principles, Structures, and Applications. *Chem. Rev.* **2018**, *118* (6), 3054–3099. https://doi.org/10.1021/acs.chemrev.7b00252.

(4) Huang, X.; Zhang, W.; Guan, G.; Song, G.; Zou, R.; Hu, J. Design and Functionalization of the NIR-Responsive Photothermal Semiconductor Nanomaterials for Cancer Theranostics. *Acc. Chem. Res.* **2017**, *50* (10), 2529–2538. https://doi.org/10.1021/acs.accounts.7b00294.

(5) Xu, D.; Li, Z.; Li, L.; Wang, J. Insights into the Photothermal Conversion of 2D MXene Nanomaterials: Synthesis, Mechanism, and Applications. *Adv. Funct. Mater.* **2020**, *30* (47). https://doi.org/10.1002/adfm.202000712.

(6) Manrique-Bedoya, S.; Abdul-Moqueet, M.; Lopez, P.; Gray, T.; Disiena, M.; Locker, A.; Kwee, S.; Tang, L.; Hood, R. L.; Feng, Y.; Large, N.; Mayer, K. M. Multiphysics Modeling of Plasmonic Photothermal Heating Effects in Gold Nanoparticles and Nanoparticle Arrays. *J. Phys. Chem. C* **2020**, *124* (31), 17172–17182. https://doi.org/10.1021/acs.jpcc.0c02443.
